# Supplementary material for: Electroreductive alkylations of (hetero)arenes with carboxylic acids
Source: Nat Commun. 2024 Jun 11;15:4970. doi: 10.1038/s41467-024-49355-1 (PMC11166922; doi:10.1038/s41467-024-49355-1)
Supplement: Supplementary file 1 — Supplementary Information [file 41467_2024_49355_MOESM1_ESM.pdf]

Supplementary Information

Electroreductive Alkylations of (Hetero)arenes with Carboxylic Acids

Bing Wang,[1] Xianshuai Huang,[1] Huihua Bi,[1] and Jie Liu*[1,2]

[1] *College of Chemistry and Chemical Engineering, State Key Laboratory of Chemo/Biosensing and Chemometrics, Hunan University, 410082, Changsha, China*

[2] *Greater Bay Area Institute for Innovation, Hunan University, Guangzhou 511300, Guangdong Province, China*

E-mail: jieliu@hnu.edu.cn

Table of contents

1. Supplementary Notes 2

2. Supplementary Methods 2

   2.1 Optimization of reaction conditions 2

   2.2 General reaction procedures 5

3. Supplementary Discussion 9

   3.1 NMR experiments for the interactions 9

   3.2 Intermediate experiments 11

   3.3 Kinetic isotope effect (KIE) experiments 13

   3.4 Natural population analysis 13

   3.5 Cyclic voltammetry experiments 13

   3.6 Characterization of products 16

   3.7 NMR spectra of products 37

4. Supplementary References 110

1. Supplementary Notes

Unless otherwise noted, all reagents were used as received from the commercial suppliers. Flash chromatography was performed using 200-300 mesh SiliaFlash 60® silica gel (Silicycle Inc.). TLC plates were visualized with UV light (254 nm). Flash chromatography was carried out silica gel (200-300 mesh). ^{1}H, ^{13}C and ^{19}F NMR spectra were recorded on a Bruker Avance III HD NMR 400 MHz instrument, and are internally referenced to the residual proto-solvent signals (note: $CDCl_3$ referenced at 7.26 ppm and 77.0 ppm, respectively). Data for ^{1}H are reported as: chemical shift (δ ppm), integration, multiplicity (s: singlet, d: doublet, t: triplet, m: multiplet), coupling constant (Hz) and assignment. GC-MS (EI) was recorded on Agilent 8860A GC systems and 5977 Series MSD. HRMS were recorded using ESI-TOF techniques. All measurements were carried out at room temperature unless otherwise stated.

2. Supplementary Methods

2.1 Optimization of reaction conditions

Supplementary Table 1. Optimization of catalysts

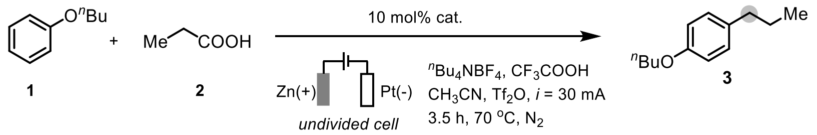

Entry	Catalyst	Yield[a]
1	$Cp^*_2TiCl_2$	54%
2	$CpTiCl_3$	66%
**3**	**Cp_2TiCl_2**	**98%**
4	Cp^*TiCl_3	50%
5	Cp_2ZrCl_2	54%
6	$Ti(O^iPr)_4$	72%
7	$TiCl_4$	68%

Reaction conditions: **1** (0.3 mmol), **2** (1.5 mmol), catalyst (10 mol%), $^nBu_4NBF_4$ (0.6 mmol), CF_3COOH (2.0 mL), CH_3CN (2.0 mL), Tf_2O (2.0 equiv.), 70 °C, N_2, 30 mA, 3.5 h, undivided cell, Zn (+) Pt (-). [a] ^{1}H NMR yield was obtained by using CH_2Br_2 as internal standard.

Supplementary Table 2. Optimization of solvents

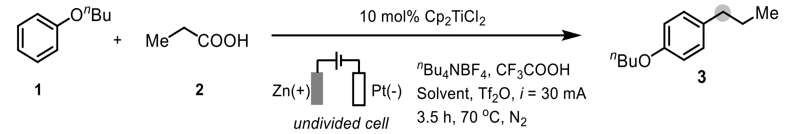

Entry	Solvent	Yield[a]
**1**	**CH_3CN**	**98%**
2	DMAc	N.D.
3	DMSO	N.D.

Reaction conditions: **1** (0.3 mmol), **2** (1.5 mmol), Cp_2TiCl_2 (10 mol%), ^{n}Bu_4NBF_4 (0.6 mmol), CF_3COOH (2.0 mL), solvent (2.0 mL), Tf_2O (2.0 equiv.), 70 °C, N_2, 30 mA, 3.5 h, undivided cell, Zn (+) Pt (-). [a] ^{1}H NMR yield was obtained by using CH_2Br_2 as internal standard.

Supplementary Table 3. Optimization of electrodes

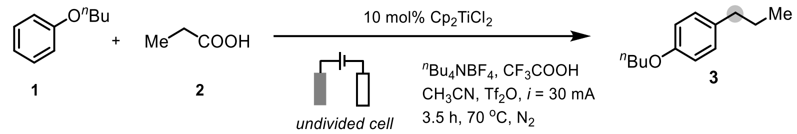

Entry	Electrode	Yield[a]
**1**	**Zn (+) Pt (-)**	**98%**
2	Zn (+) C plate (-)	63%
3	Zn (+) Ni (-)	52%
4	Al (+) Pt (-)	36%
5	Fe (+) Pt (-)	N.D.

Reaction conditions: **1** (0.3 mmol), **2** (1.5 mmol), Cp_2TiCl_2 (10 mol%), ^{n}Bu_4NBF_4 (0.6 mmol), CF_3COOH (2.0 mL), CH_3CN (2.0 mL), Tf_2O (2.0 equiv.), 70 °C, N_2, 30 mA, 3.5 h, undivided cell. [a] ^{1}H NMR yield was obtained by using CH_2Br_2 as internal standard.

Supplementary Table 4. Optimization of electrolytes

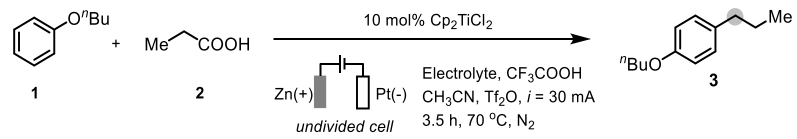

Entry	Electrolyte	Yield[a]
**1**	**^{n}Bu_4NBF_4**	**98%**
2	^{n}Bu_4NPF_6	11%
3	^{n}Bu_4NOAc	27%

Reaction conditions: **1** (0.3 mmol), **2** (1.5 mmol), Cp_2TiCl_2 (10 mol%), electrolyte (0.6 mmol), CF_3COOH (2.0 mL), CH_3CN (2.0 mL), Tf_2O (2.0 equiv.), 70 °C, N_2, 30 mA, 3.5 h, undivided cell, Zn (+) Pt (-). [a] ^{1}H NMR yield was obtained by using CH_2Br_2 as internal standard.

Supplementary Table 5. Optimization of temperature

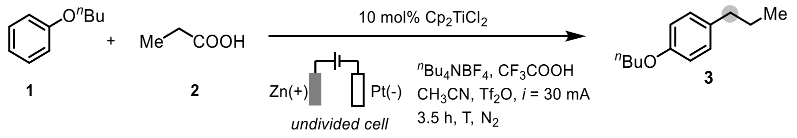

Entry	Temperature	Yield[a]
**1**	**70 ºC**	**98%**
2	60 ºC	85%

Reaction conditions: **1** (0.3 mmol), **2** (1.5 mmol), Cp_2TiCl_2 (10 mol%), ^{n}Bu_4NBF_4 (0.6 mmol), CF_3COOH (2.0 mL), CH_3CN (2.0 mL), Tf_2O (2.0 equiv.), temperature, N_2, 30 mA, 3.5 h, undivided cell, Zn (+) Pt (-). [a] ^{1}H NMR yield was obtained by using CH_2Br_2 as internal standard.

Supplementary Table 6. Optimization of activating reagent

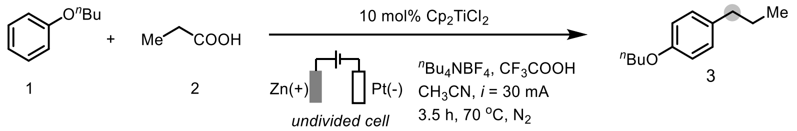

Entry	Conditions	Yield[a]
1	Tf_2O	98%
2	TFAA	0
3	$SOCl_2$	0
4	$POCl_3$	0
5	Boc_2O	0

Reaction conditions: **1** (0.3 mmol), **2** (1.5 mmol), Cp_2TiCl_2 (10 mol%), ^{n}Bu_4NBF_4 (0.6 mmol), CF_3COOH (2.0 mL), CH_3CN (2.0 mL), activating reagent (2.0 equiv.), 70 °C, N_2, 30 mA, 3.5 h, undivided cell, Zn (+) Pt (-). [a] ^{1}H NMR yield was obtained by using CH_2Br_2 as internal standard.

Supplementary Table 7. Control experiments

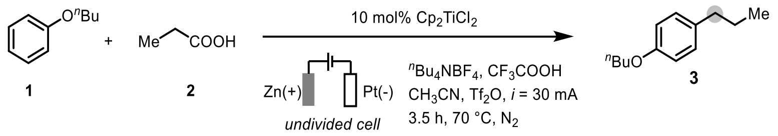

Entry	Conditions	Yield[a]
1	No Cp_2TiCl_2	67%
2	No TFA	37%
3	No Tf_2O	0
4	No electrolysis	0
5	1 bar H_2 instead of electrolysis	0

Reaction conditions: **1** (0.3 mmol), **2** (1.5 mmol), Cp_2TiCl_2 (10 mol%), ^{n}Bu_4NBF_4 (0.6 mmol), CF_3COOH (2.0 mL), CH_3CN (2.0 mL), Tf_2O (2.0 equiv.), 70 °C, N_2, 30 mA, 3.5 h, undivided cell, Zn (+) Pt (-). [a] ^{1}H NMR yield was obtained by using CH_2Br_2 as internal standard.

2.2 General reaction procedures

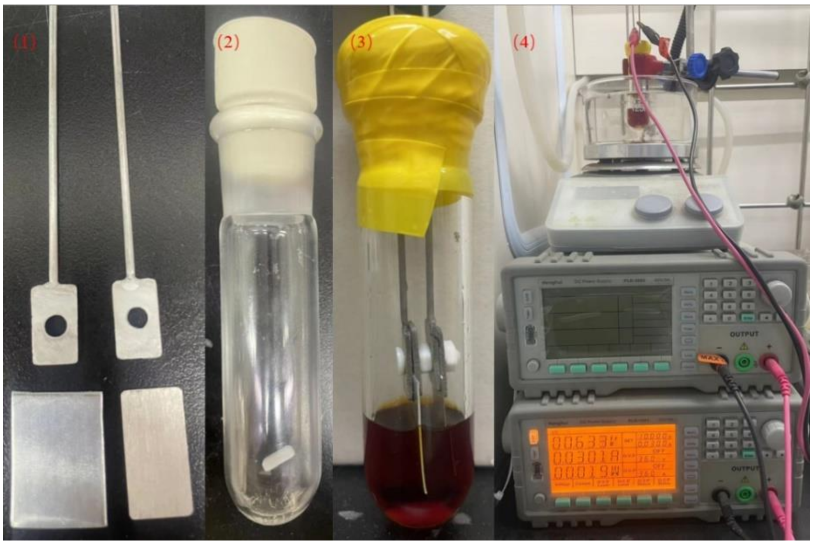

Supplementary Figure 1. Overview of reaction set-up.

(1) an Electrode holder, Zn electrode and Pt electrode.

(2) A dried 10 mL glass tube.

(3) The Zn and Pt were fitted into the tube.

(4) Conducted constant current electrolysis (I = 30.0 mA) using a potentiostat.

2.2.1 Reaction procedure of electrochemical alkylations using carboxylic acids

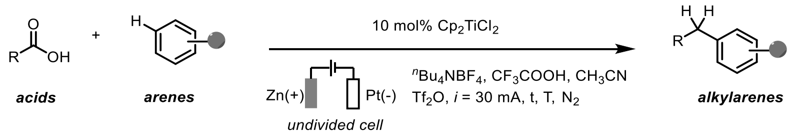

Condition A： A dried 10 mL glass tube equipped with a magnetic stirring bar was added Cp_2TiCl_2 (7.5 mg, 0.03 mmol, 0.1 equiv.), arene (0.3 mmol, 1.0 equiv.), carboxylic acid (1.5 mmol, 5.0 equiv.), $^nBu_4NBF_4$ (197.5 mg, 0.6 mmol), Tf_2O (0.6 mmol, 2.0 equiv.), CF_3COOH(D) (2.0 mL) and CH_3CN (2.0 mL). The reactor was equipped with Zn electrode (20×15×0.5 mm) as the anode and Pt electrode (20×10×0.2 mm) as the cathode. The reaction was bubbled with N_2 for five minutes. Then the mixture was electrolyzed under a constant current of 30 mA for 3.5 h at 70 °C. After reaction was completed, the reaction solvent was diluted with 40 mL ethyl acetate, washed with sat. $NaHCO_3$ aq. solution for three times, dried over Na_2SO_4 and organic layers were combined and concentrated *in vacuo*. The resulting residue was purified by silica gel flash chromatography to give the products.

Condition B：A dried 10 mL glass tube equipped with a magnetic stirring bar was added Cp_2TiCl_2 (7.5 mg, 0.03 mmol, 0.1 equiv.), arene (0.3 mmol, 1.0 equiv.), carboxylic acid (1.5 mmol, 5.0 equiv.), $^{n}Bu_4NBF_4$ (197.5 mg, 0.6 mmol), Tf_2O (0.6 mmol, 2.0 equiv.), $CF_3COOH(D)$ (2.0 mL) and CH_3CN (2.0 mL). The reactor was equipped with Zn electrode (20×10×0.5 mm) as the anode and Pt electrode (20×10×0.2 mm) as the cathode. The reaction was bubbled with N_2 for five minutes. Then the mixture was electrolyzed under a constant current of 30 mA for 5 h at 60 °C. After reaction was completed, the reaction solvent was diluted with 40 mL ethyl acetate, washed with sat. $NaHCO_3$ aq. solution for three times, dried over Na_2SO_4 and organic layers were combined and concentrated *in vacuo*. The resulting residue was purified by silica gel flash chromatography to give the products.

Condition C：A dried 10 mL glass tube equipped with a magnetic stirring bar was added Cp_2TiCl_2 (7.5 mg, 0.03 mmol, 0.1 equiv.), arene (0.3 mmol, 1.0 equiv.), carboxylic acid (1.5 mmol, 5.0 equiv.), $^{n}Bu_4NBF_4$ (197.5 mg, 0.6 mmol), Tf_2O (0.675 mmol, 2.25 equiv.), $CF_3COOH(D)$ (3.0 mL) and CH_3CN (1.0 mL). The reactor was equipped with Zn electrode (20×15×0.5 mm) as the anode and Pt electrode (20×10×0.2 mm) as the cathode. The reaction was bubbled with N_2 for five minutes. Then the mixture was electrolyzed under a constant current of 30 mA for 6 h at 50 °C. After reaction was completed, the reaction solvent was diluted with 40 mL ethyl acetate, washed with sat. $NaHCO_3$ aq. solution for three times, dried over Na_2SO_4 and organic layers were combined and concentrated *in vacuo*. The resulting residue was purified by silica gel flash chromatography to give the products.

Condition D：A dried 10 mL glass tube equipped with a magnetic stirring bar was added Cp_2TiCl_2 (7.5 mg, 0.03 mmol, 0.1 equiv.), arene (0.3 mmol, 1.0 equiv.), carboxylic acid (1.5 mmol, 5.0 equiv.), $^{n}Bu_4NBF_4$ (197.5 mg, 0.6 mmol), Tf_2O (0.675 mmol, 2.25 equiv.), $CF_3COOH(D)$ (2.0 mL) and CH_3CN (2.0 mL). The reactor was equipped with Zn electrode (20×15×0.5 mm) as the anode and Pt electrode (20×10×0.2 mm) as the cathode. The reaction was bubbled with N_2 for five minutes. Then the mixture was electrolyzed under a constant current of 30 mA for 6 h at 60 °C. After reaction was completed, the reaction solvent was diluted with 40 mL ethyl acetate, washed with sat. $NaHCO_3$ aq. solution for three times, dried over

Na_2SO_4 and organic layers were combined and concentrated *in vacuo*. The resulting residue was purified by silica gel flash chromatography to give the products.

Condition E: A dried 10 mL glass tube equipped with a magnetic stirring bar was added Cp_2TiCl_2 (7.5 mg, 0.03 mmol, 0.1 equiv.), arene (0.3 mmol, 1.0 equiv.), carboxylic acid (1.5 mmol, 5.0 equiv.), $^{n}Bu_4NBF_4$ (197.5 mg, 0.6 mmol), Tf_2O (0.3 mmol, 1.0 equiv.), CF_3COOH(D) (2.0 mL) and CH_3CN (2.0 mL). The reactor was equipped with Zn electrode (20×15×0.5 mm) as the anode and Pt electrode (20×10×0.2 mm) as the cathode. The reaction was bubbled with N_2 for five minutes. Then the mixture was electrolyzed under a constant current of 30 mA for 6 h at room temperature. After reaction was completed, the reaction solvent was diluted with 40 mL ethyl acetate, washed with sat. $NaHCO_3$ aq. solution for three times, dried over Na_2SO_4 and organic layers were combined and concentrated *in vacuo*. The resulting residue was purified by silica gel flash chromatography to give the products.

Condition F: A dried 10 mL glass tube equipped with a magnetic stirring bar was added Cp_2TiCl_2 (7.5 mg, 0.03 mmol, 0.1 equiv.), arene (0.3 mmol, 1.0 equiv.), carboxylic acid (1.5 mmol, 5.0 equiv.), $^{n}Bu_4NBF_4$ (197.5 mg, 0.6 mmol), Tf_2O (0.6 mmol, 2.0 equiv.), CF_3COOH(D) (1.0 mL) and CH_3CN (2.0 mL). The reactor was equipped with Zn electrode (20×10×0.5 mm) as the anode and Pt electrode (20×10×0.2 mm) as the cathode. The reaction was bubbled with N_2 for five minutes. Then the mixture was electrolyzed under a constant current of 30 mA for 5 h at room temperature. After reaction was completed, the reaction solvent was diluted with 40 mL ethyl acetate, washed with sat. $NaHCO_3$ aq. solution for three times, dried over Na_2SO_4 and organic layers were combined and concentrated *in vacuo*. The resulting residue was purified by silica gel flash chromatography to give the products.

2.2.2 Reaction procedure of electrochemical trifluoromethylation of indole

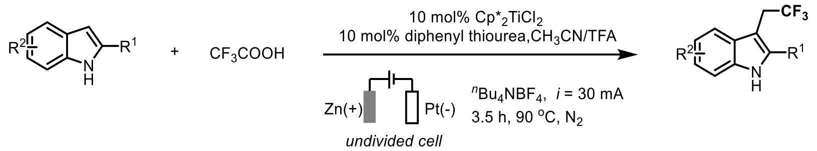

Condition G: A dried 10 mL glass tube equipped with a magnetic stirring bar was added $Cp^{*}_2TiCl_2$ (11.7 mg, 0.03 mmol, 0.1 equiv.), diphenyl thiourea (6.8 mg, 0.03 mmol, 0.1 equiv.), indole (0.3 mmol, 1.0 equiv.), $^{n}Bu_4NBF_4$ (197.5 mg, 0.6 mmol), CF_3COOH (0.9 mL) and

CH_3CN (3.0 mL). The reactor was equipped with Zn electrode (20×10×0.5 mm) as the anode and Pt electrode (20×10×0.2 mm) as the cathode. The reaction was bubbled with N_2 for five minutes. Then the mixture was electrolyzed under a constant current of 30 mA for 3.5 h at 90 °C. After reaction was completed, the reaction solvent was diluted with 40 mL ethyl acetate, washed with sat. $NaHCO_3$ aq. solution for three times, dried over Na_2SO_4 and organic layers were combined and concentrated *in vacuo*. The resulting residue was purified by silica gel flash chromatography to give the products.

2.2.3 Reaction procedure of gram-scale reaction

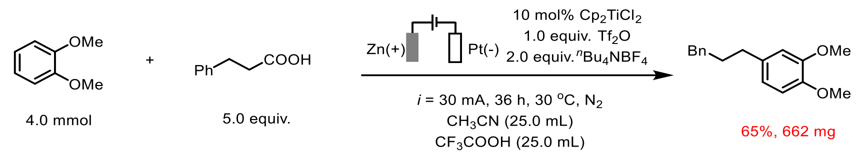

A dried 100 mL three-necked flask equipped with a magnetic stirring bar was added Cp_2TiCl_2 (99.6 mg, 0.4 mmol, 0.1 equiv.), veratrole (514 µL, 4 mmol, 1.0 equiv.), 3-Phenylpropanoic acid (3.0 g, 20 mmol, 5.0 equiv.), $^nBu_4NBF_4$ (2.6 g, 8 mmol), Tf_2O (673 µL, 4 mmol, 1.0 equiv.), CF_3COOH (25.0 mL) and CH_3CN (25.0 mL). The reactor was equipped with Zn electrode (60×40×0.5 mm) as the anode and Pt electrode (20×10×0.2 mm) as the cathode. The reaction was bubbled with N_2 for five minutes. Then the mixture was electrolyzed under a constant current of 30 mA for 36 h at 30 °C. After reaction was completed, the reaction solvent was diluted with 200 mL ethyl acetate, washed with sat. $NaHCO_3$ aq. solution for three times, dried over Na_2SO_4 and organic layers were combined and concentrated *in vacuo*. The resulting residue was purified by silica gel flash chromatography to give the products.

2.2.4 Control experiments with or without Ti catalyst

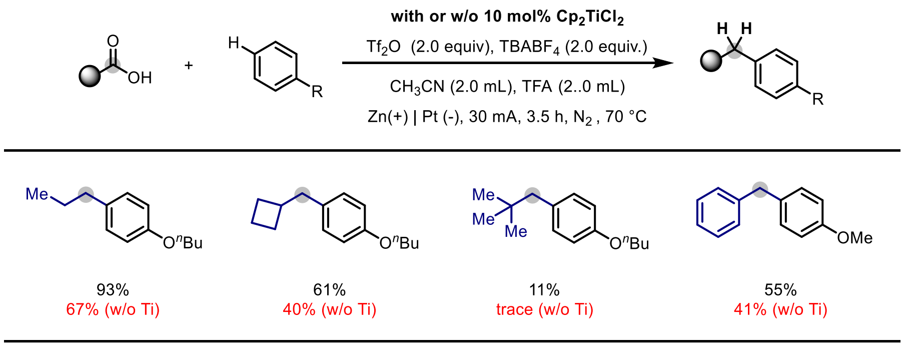

Supplementary Figure 2. Control experiments with or without Ti catalyst.

2.2.5 Unsuccessful substrates in this reaction

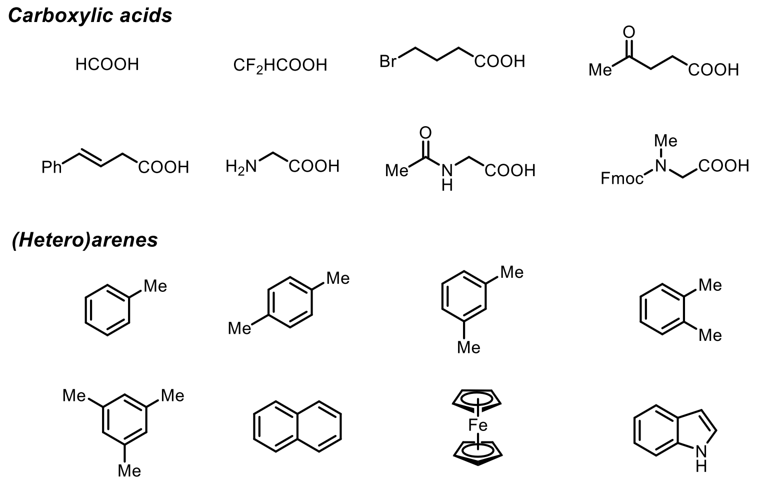

Supplementary Figure 3. Substrate limitations in this reaction.

3. Supplementary Discussion

3.1 NMR experiments for the interactions

^{13}C NMR experiments were recorded using Bruker Avance III HD NMR 400 MHz instrument. The sample was sonicated in $CDCl_3$ (400 μL) for 10 min at room temperature.

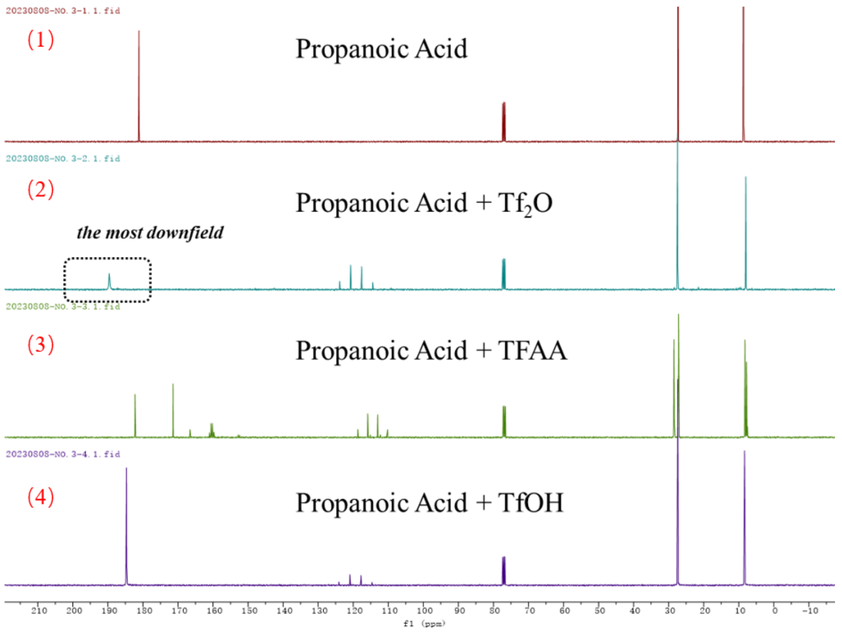

Supplementary Figure 4. ^{13}C NMR investigations.

(1) propanoic acid (0.3mmol).

(2) propanoic acid (0.3mmol) and Tf_2O (0.6 mmol).

(3) propanoic acid (0.3mmol) and TFAA (0.6 mmol).

(4) propanoic acid (0.3mmol) and TfOH (0.6 mmol).

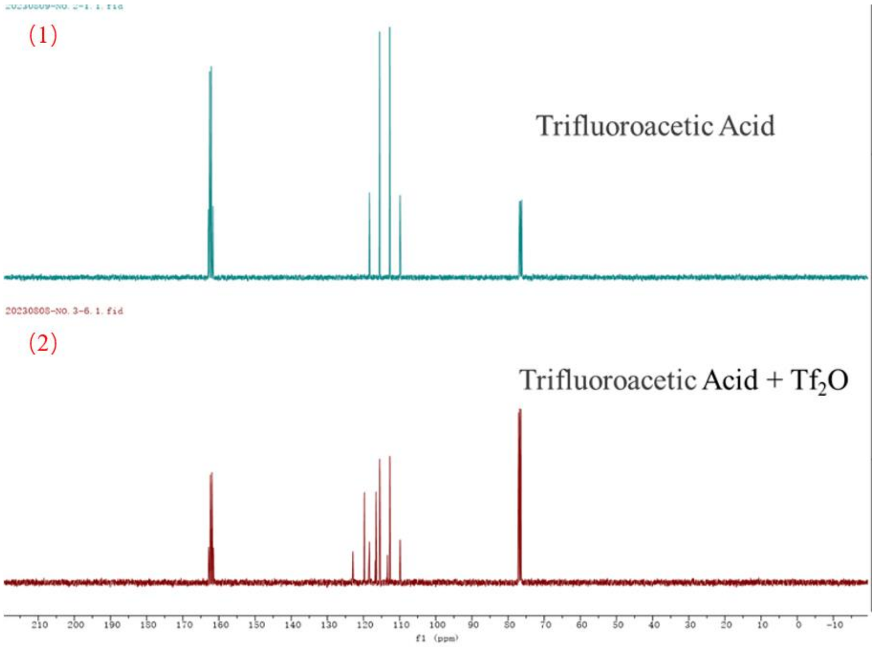

Supplementary Figure 5. ^{13}C NMR investigation of activation method of trifluoroacetic acid (TFA).

(1) TFA (0.3mmol).

(2) TFA (0.3 mmol) and Tf_2O (0.6 mmol).

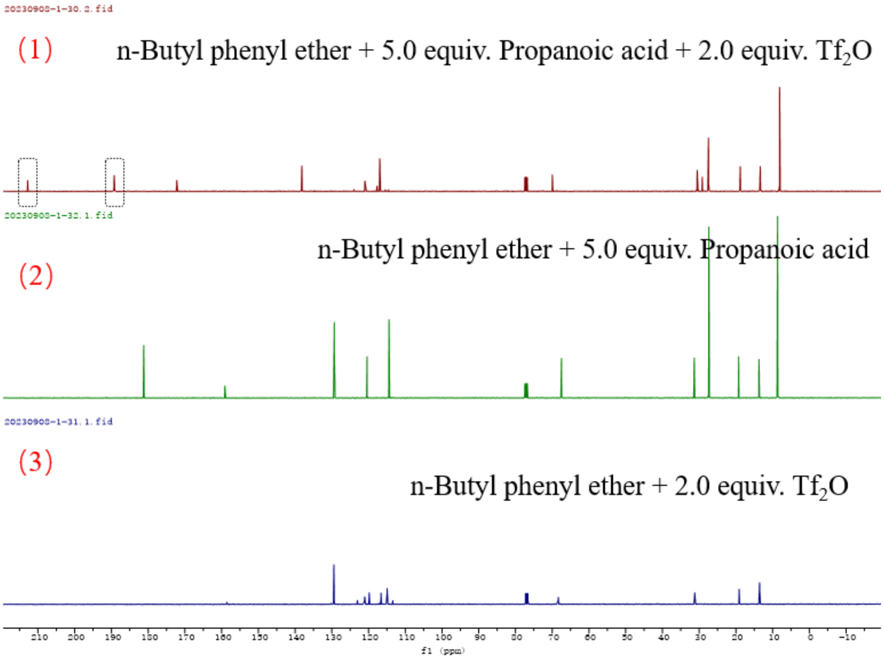

Supplementary Figure 6. ^{13}C NMR investigation of reaction system.

(1) *n*-Butyl phenyl ether (0.3mmol), propanoic acid (1.5 mmol) and Tf_2O (0.6 mmol).

(2) *n*-Butyl phenyl ether (0.3mmol) and propanoic acid (1.5 mmol).

(3) *n*-Butyl phenyl ether (0.3mmol) and Tf_2O (0.6 mmol).

3.2 Intermediate experiments

3.2.1 Reactions of possible intermediates

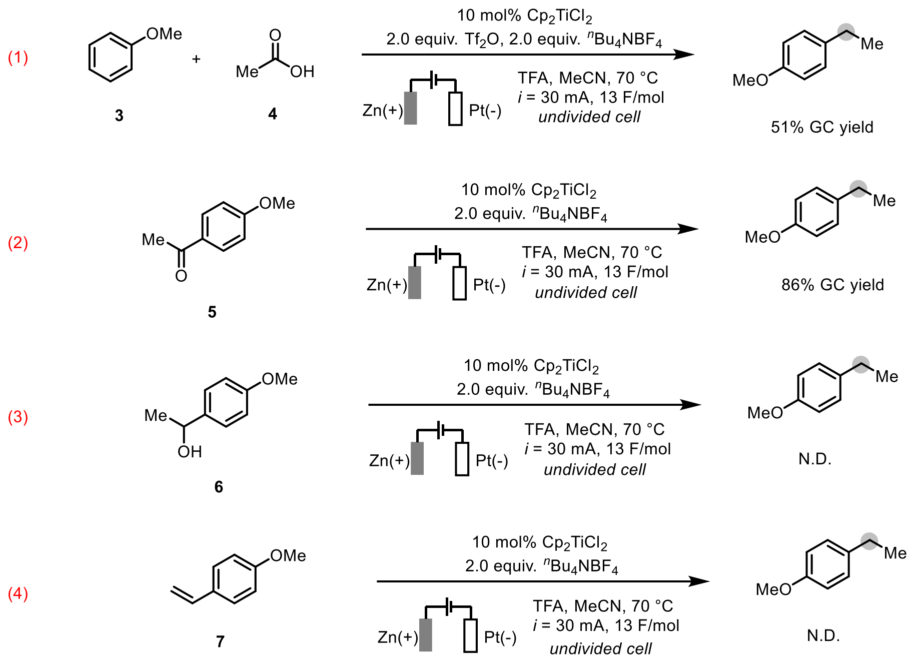

Supplementary Figure 7. Investigation of possible intermediates.

Reaction conditions: Cp_2TiCl_2 (10 mol%), $^nBu_4NBF_4$ (0.6 mmol), CF_3COOH (2.0 mL), CH_3CN (2.0 mL), Tf_2O (2.0 equiv.), 70 °C, N_2, 30 mA, 3.5 h, undivided cell, Zn (+) Pt (-), (1) **3** (0.3 mmol), **4** (1.5 mmol). (2) **5** (0.3 mmol), without Tf_2O. (3) **6** (0.3 mmol), without Tf_2O. (4) **7** (0.3 mmol), without Tf_2O. Yields are determined by GC using dodecane as an internal standard.

3.2.2 Radical capture experiments

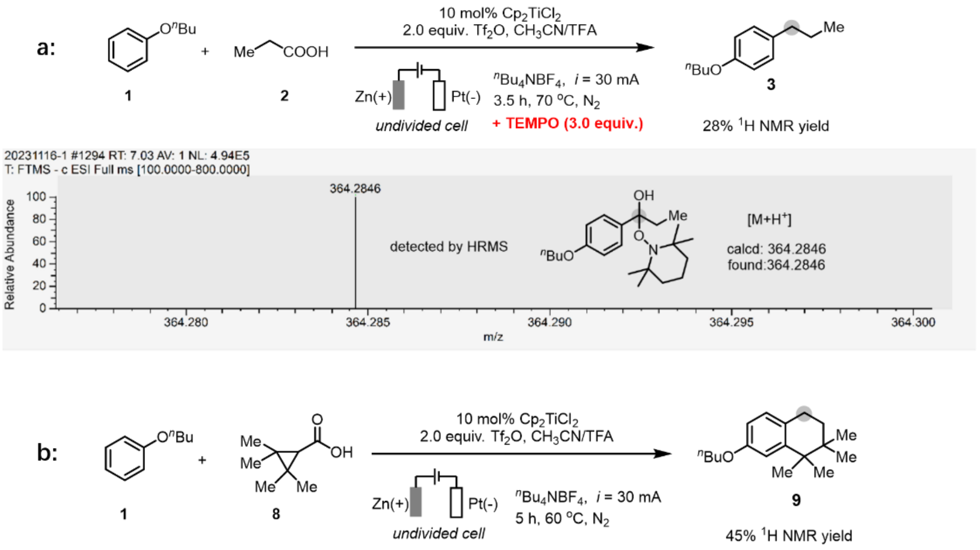

Supplementary Figure 8. Radical capture experiments.

Reaction condition a: **1** (0.3 mmol), **2** (1.5 mmol), Cp_2TiCl_2 (10 mol%), $^nBu_4NBF_4$ (0.6 mmol), CF_3COOH (2.0 mL), CH_3CN (2.0 mL), Tf_2O (2.0 equiv.), TEMPO (3.0 equiv.) 70 °C, N_2, 30 mA, 3.5 h,

undivided cell, Zn (+) Pt (-). Reaction condition b: **1** (0.3 mmol), **8** (1.5 mmol), Cp_2TiCl_2 (10 mol%), $^nBu_4NBF_4$ (0.6 mmol), CF_3COOH (2.0 mL), CH_3CN (2.0 mL), Tf_2O (2.0 equiv.), 60 °C, N_2, 30 mA, 5 h, undivided cell, Zn (+) Pt (-).

3.2.3 HRMS of catalytic species

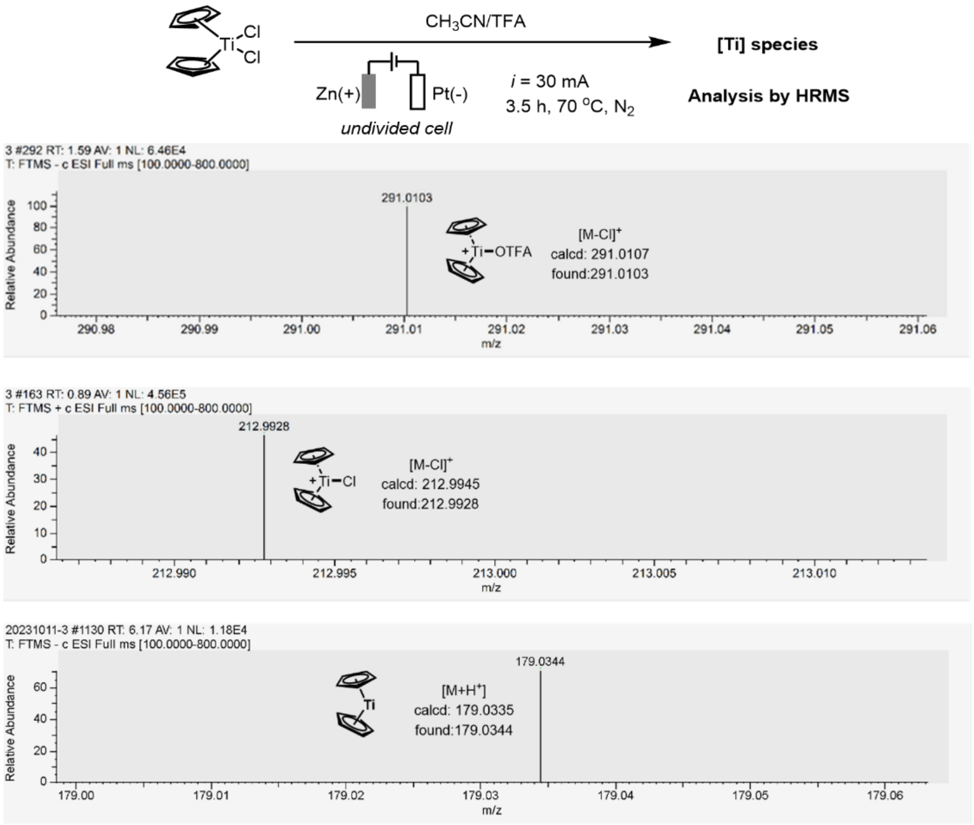

Supplementary Figure 9. HRMS of Ti species after electrolysis.

Reaction conditions: Cp_2TiCl_2 (0.03 mmol), CF_3COOH (2.0 mL), CH_3CN (2.0 mL), 70 °C, N_2, 30 mA, 3.5 h, undivided cell, Zn (+) Pt (-).

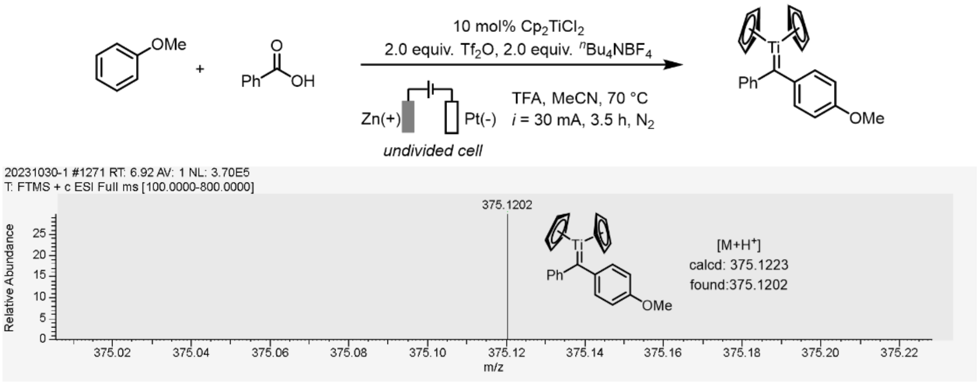

Supplementary Figure 10. Observation of titanium carbene species.

Reaction conditions: anisole (0.3 mmol), benzoic acid (1.5 mmol), Cp_2TiCl_2 (10 mol%), $^{n}Bu_4NBF_4$ (0.6 mmol), CF_3COOH (2.0 mL), CH_3CN (2.0 mL), Tf_2O (2.0 equiv.), 70 °C, N_2, 30 mA, 3.5 h, undivided cell, Zn (+) Pt (-).

3.3 Kinetic isotope effect (KIE) experiments

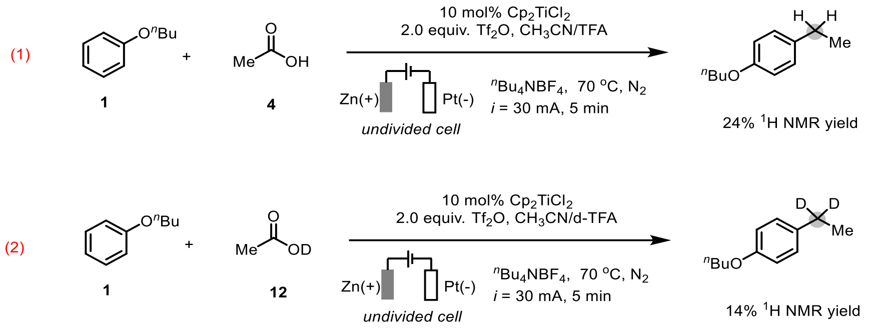

Supplementary Figure 11. Kinetic isotope effect experiments.

Reaction conditions: (1) **1** (0.3 mmol), **4** (1.5 mmol), Cp_2TiCl_2 (10 mol%), $^{n}Bu_4NBF_4$ (0.6 mmol), CF_3COOH (2.0 mL), CH_3CN (2.0 mL), Tf_2O (2.0 equiv.), 70 °C, N_2, 30 mA, 3.5 h, undivided cell, Zn (+) Pt (-). (2) **1** (0.3 mmol), **12** (1.5 mmol), Cp_2TiCl_2 (10 mol%), $^{n}Bu_4NBF_4$ (0.6 mmol), CF_3COOD (2.0 mL), CH_3CN (2.0 mL), Tf_2O (2.0 equiv.), 70 °C, N_2, 30 mA, 3.5 h, undivided cell, Zn (+) Pt (-).

3.4 Natural population analysis

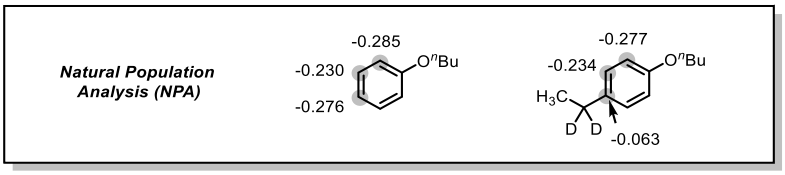

Supplementary Figure 12. Natural population analysis.

There is more charge distribution at the ortho-position of ether functionality than the meta- and para-positions. Therefore, for some substrate in Fig. 3a, we observed partly deuterated at the ortho-position of ether group.

3.5 Cyclic voltammetry experiments

Cyclic voltammetry was recorded using a CHI660E potentiostat at room temperature. A glassy carbon electrode (3 mm-diameter), Pt wire, and Ag/AgCl (in saturated potassium chloride) were used as the working, counter, and reference electrodes, respectively. CH_3CN was used as the solvent in the presence of 100 mM $^{n}Bu_4NBF_4$ as supporting electrolyte. All experiments were performed under nitrogen.

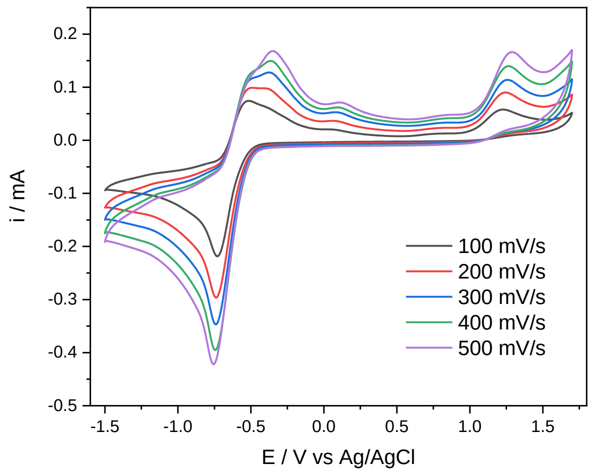

Supplementary Figure 13. Cyclic voltammetry of Cp_2TiCl_2 in CH_3CN with 0.1 M $^{n}Bu_4NBF_4$ at varied sweep rates.

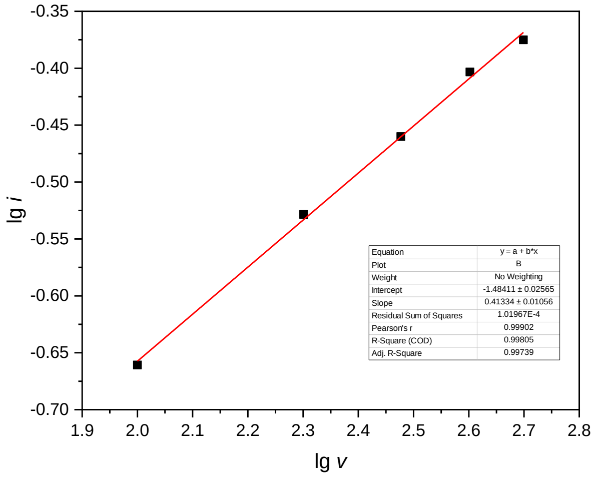

Supplementary Figure 14. Linear relation between logarithm of reduction peak current and logarithm of scan rate.

A straight line with a slope of 0.41 close to the theoretical value of 0.5 for a purely diffusion-controlled process.

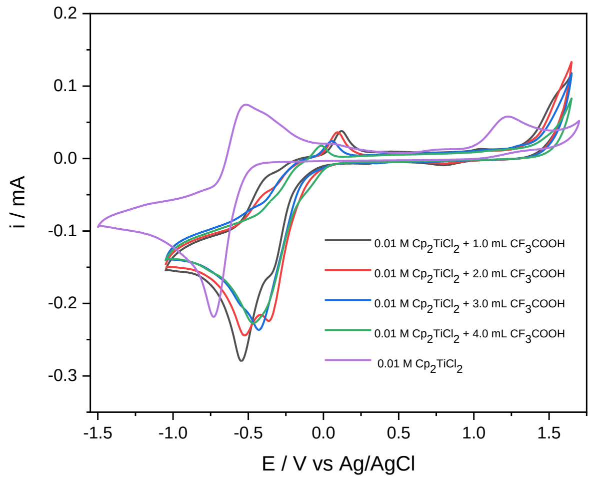

Supplementary Figure 15. Cyclic voltammetry of Cp_2TiCl_2 in CH_3CN in the presence of CF_3COOH with 0.1 M $^{n}Bu_4NBF_4$, acquired at a scan rate of 100 mV/s.

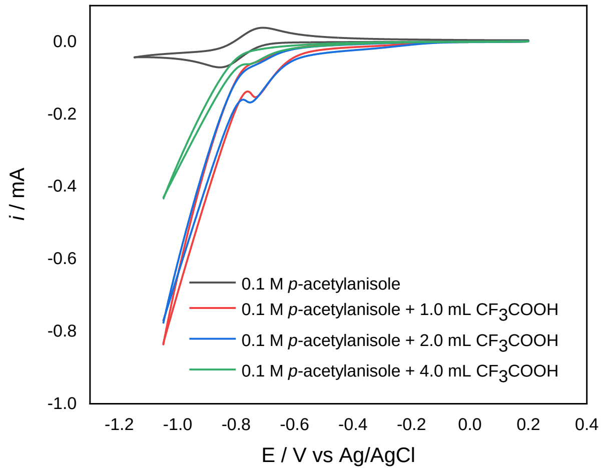

Supplementary Figure 16. Cyclic voltammetry of *p*-acetylanisole in CH_3CN in the presence of CF_3COOH with 0.1 M $^{n}Bu_4NBF_4$, acquired at a scan rate of 100 mV/s.

3.6 Characterization of products

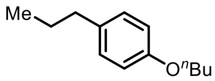

1-butoxy-4-propylbenzene (3)

Following the representative **Condition A**, colorless oil, yield: 93% (53.4 mg). Flash silica gel chromatography (petroleum ether), *p*:*o* > 50:1.

^{1}H NMR (400 MHz, $CDCl_3$) δ 7.12 (d, *J* = 7.5 Hz, 2H), 6.87 (d, *J* = 7.5 Hz, 2H), 3.98 (t, *J* = 6.2 Hz, 2H), 2.57 (t, *J* = 7.5 Hz, 2H), 1.85-1.75 (m, 2H), 1.70-1.62 (m, 2H), 1.58-1.50 (m, 2H), 1.05-0.95 (m, 6H).

^{13}C NMR (101 MHz, $CDCl_3$) δ 157.2, 134.6, 129.2, 114.2, 67.6, 37.1, 31.4, 24.8, 19.3, 13.9, 13.8.

ESI HRMS for [$C_{13}H_{20}O+H^+$] calculated: 193.1587, found: 193.1585.

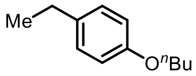

1-butoxy-4-ethylbenzene (4) [1-2]

Following the representative **Condition A**, colorless oil, yield: 81% (43.4 mg). Flash silica gel chromatography (petroleum ether), *p*:*o* > 50:1.

^{1}H NMR (400 MHz, $CDCl_3$) δ 7.15 (d, *J* = 8.4 Hz, 2H), 6.88 (d, *J* = 8.4 Hz, 2H), 3.98 (t, *J* = 6.5 Hz, 2H), 2.64 (q, *J* = 7.6 Hz, 2H), 1.85-1.75 (m, 2H), 1.58-1.50 (m, 2H), 1.26 (t, *J* = 7.6 Hz, 3H), 1.02 (t, *J* = 7.4 Hz, 3H).

^{13}C NMR (101 MHz, $CDCl_3$) δ 157.2, 136.1, 128.6, 114.3, 67.6, 31.4, 28.0, 19.3, 15.9, 13.8.

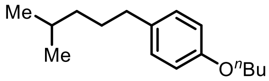

1-butoxy-4-(4-methylpentyl)benzene (5)

Following the representative **Condition A**, colorless oil, yield: 72% (50.5 mg). Flash silica gel chromatography (petroleum ether), *p*:*o* > 50:1.

^{1}H NMR (400 MHz, $CDCl_3$) δ 7.10 (d, *J* = 8.1 Hz, 2H), 6.84 (d, *J* = 8.1 Hz, 2H), 3.96 (t, *J* = 6.5 Hz, 2H), 2.54 (t, *J* = 7.7 Hz, 2H), 1.82-1.73 (m, 2H), 1.65-1.56 (m, 3H), 1.55-1.48 (m, 2H), 1.28-1.19 (m, 2H), 1.00 (t, *J* = 7.4 Hz, 3H), 0.90 (d, *J* = 6.6 Hz, 6H).

^{13}C NMR (101 MHz, $CDCl_3$) δ 157.2, 134.8, 129.2, 114.3, 67.7, 38.6, 35.3, 31.4, 29.6, 27.9, 22.6, 19.3, 13.9.

ESI HRMS for [$C_{16}H_{26}O+H^+$] calculated: 235.2056, found: 235.2057.

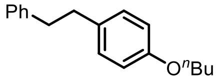

1-butoxy-4-phenethylbenzene (6)

Following the representative **Condition A**, colorless oil, yield: 76% (58.1 mg). Flash silica gel chromatography (petroleum ether), *p*:*o* > 50:1.

^{1}H NMR (400 MHz, $CDCl_3$) δ 7.42-7.35 (m, 2H), 7.33-7.24 (m, 3H), 7.18 (d, *J* = 7.7 Hz, 2H), 6.92 (d, *J* = 7.7 Hz, 2H), 4.04 (t, *J* = 6.3 Hz, 2H), 2.98 (s, 4H), 1.91-1.81 (m, 2H), 1.66-1.56 (m, 2H), 1.08 (t, *J* = 7.2 Hz, 3H).

^{13}C NMR (101 MHz, $CDCl_3$) δ 157.4, 141.8, 133.6, 129.2, 128.4, 128.2, 125.8, 114.3, 67.6, 38.2, 37.0, 31.4, 19.2, 13.9.

ESI HRMS for [$C_{18}H_{22}O+H^+$] calculated: 255.1743, found: 255.1747.

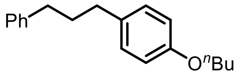

1-butoxy-4-(3-phenylpropyl)benzene (7)

Following the representative **Condition A**, colorless oil, yield: 88% (71.0 mg). Flash silica gel chromatography (petroleum ether), *p*:*o* > 50:1.

^{1}H NMR (400 MHz, $CDCl_3$) δ 7.34-7.26 (m, 2H), 7.25-7.17 (m, 3H), 7.11 (d, *J* = 8.3 Hz, 2H), 6.85 (d, *J* = 8.3 Hz, 2H), 3.96 (t, *J* = 6.5 Hz, 2H), 2.64 (m, 4H), 2.00-1.90 (m, 2H), 1.84-1.73 (m, 2H), 1.56-1.47 (m, 2H), 1.00 (t, *J* = 7.4 Hz, 3H).

^{13}C NMR (101 MHz, $CDCl_3$) δ 157.3, 142.4, 134.1, 129.2, 128.4, 128.2, 125.7, 114.3, 67.6, 35.4, 34.5, 33.2, 31.4, 19.3, 13.9.

ESI HRMS for [$C_{19}H_{24}O+H^+$] calculated: 269.1900, found: 269.1900.

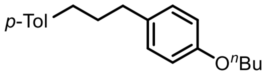

1-butoxy-4-(3-(p-tolyl)propyl)benzene (8)

Following the representative **Condition A**, colorless oil, yield: 65% (54.8 mg). Flash silica gel chromatography (petroleum ether), *p*:*o* > 50:1.

^{1}H NMR (400 MHz, $CDCl_3$) δ 7.12 (s, 6H), 6.86 (d, *J* = 8.0 Hz, 2H), 3.97 (t, *J* = 6.5 Hz, 2H), 2.67-2.59 (m, 4H), 2.36 (s, 3H), 1.99-1.89 (m, 2H), 1.84-1.75 (m, 2H), 1.59-1.49 (m, 2H), 1.01 (t, *J* = 7.4 Hz, 3H).

^{13}C NMR (101 MHz, $CDCl_3$) δ 157.2, 139.3, 135.0, 134.2, 129.2, 128.9, 128.3, 114.3, 67.6, 34.9, 34.5, 33.3, 31.4, 21.0, 19.3, 13.9.

ESI HRMS for [$C_{20}H_{26}O+H^+$] calculated: 283.2056, found: 283.2055.

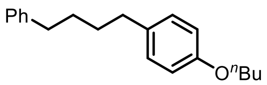

1-butoxy-4-(4-phenylbutyl)benzene (9)

Following the representative **Condition A**, colorless oil, yield: 49% (41.6 mg). Flash silica gel chromatography (petroleum ether), *p*:*o* > 50:1.

^{1}H NMR (400 MHz, $CDCl_3$) δ 7.34-7.27 (m, 2H), 7.24-7.17 (m, 3H), 7.10 (d, *J* = 8.0 Hz, 2H), 6.85 (d, *J* = 8.0 Hz, 2H), 3.97 (t, *J* = 6.4 Hz, 2H), 2.66 (t, *J* = 6.6 Hz, 2H), 2.61 (t, *J* = 6.4 Hz, 2H), 1.83-1.75 (m, 2H), 1.73-1.62 (m, 4H), 1.57-1.48 (m, 2H), 1.01 (t, *J* = 7.3 Hz, 3H).

^{13}C NMR (101 MHz, $CDCl_3$) δ 157.2, 142.6, 134.4, 129.2, 128.4, 128.2, 125.6, 114.3, 67.6, 35.8, 34.9, 31.4, 31.3, 31.0, 19.3, 13.9.

ESI HRMS for [$C_{20}H_{26}O+H^+$] calculated: 283.2056, found: 283.2055.

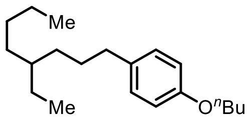

1-butoxy-4-(4-ethyloctyl)benzene (10)

Following the representative **Condition A**, colorless oil, yield: 57% (49.5 mg). Flash silica gel chromatography (petroleum ether), *p*:*o* > 50:1.

^{1}H NMR (400 MHz, $CDCl_3$) δ 7.09 (d, *J* = 8.3 Hz, 2H), 6.82 (d, *J* = 8.3 Hz, 2H), 3.94 (t, *J* = 6.5 Hz, 2H), 2.52 (t, *J* = 7.7 Hz, 2H), 1.80-1.72 (m, 2H), 1.59-1.48 (m, 4H), 1.34 -1.18 (m, 11H), 0.98 (t, *J* = 7.4 Hz, 3H), 0.89 (t, *J* = 6.8 Hz, 3H), 0.83 (t, J = 6.9 Hz, 3H).

^{13}C NMR (101 MHz, $CDCl_3$) δ 157.4, 135.1, 129.4, 114.5, 67.9, 38.9, 35.7, 33.1, 33.0, 31.6, 29.2, 29.1, 26.1, 23.3, 19.5, 14.3, 14.1, 11.1.

ESI HRMS for [$C_{20}H_{34}O+H^+$] calculated: 291.2682, found: 291.2686.

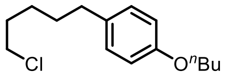

1-butoxy-4-(5-chloropentyl)benzene (11)

Following the representative **Condition A**, colorless oil, yield: 75% (57.4 mg). Flash silica gel chromatography (petroleum ether), *p*:*o* > 50:1.

^{1}H NMR (400 MHz, $CDCl_3$) δ 7.09 (d, *J* = 8.0 Hz, 2H), 6.84 (d, *J* = 8.0 Hz, 2H), 3.95 (t, *J* = 6.5 Hz, 2H), 3.54 (t, *J* = 6.7 Hz, 2H), 2.58 (t, *J* = 7.6 Hz, 2H), 1.86-1.73 (m, 4H), 1.66-1.59 (m, 2H), 1.55-1.44 (m, 4H), 0.99 (t, *J* = 7.3 Hz, 3H).

^{13}C NMR (101 MHz, $CDCl_3$) δ 157.2, 134.1, 129.1, 114.3, 67.6, 45.0, 34.8, 32.5, 31.4, 31.0, 26.4, 19.2, 13.8.

ESI HRMS for [$C_{15}H_{23}ClO+H^+$] calculated: 255.1510, found: 255.1511.

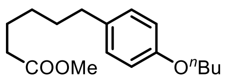

Methyl 6-(4-butoxyphenyl)hexanoate (12)

Following the representative **Condition A**, colorless oil, yield: 68% (56.6 mg). Flash silica gel chromatography (petroleum ether/ethyl acetate = 100/1), *p*:*o* > 50:1.

^{1}H NMR (400 MHz, $CDCl_3$) δ 7.07 (d, *J* = 8.3 Hz, 2H), 6.81 (d, *J* = 8.3 Hz, 2H), 3.94 (t, *J* = 6.5 Hz, 2H), 3.66 (s, 3H), 2.55 (t, *J* = 7.7 Hz, 2H), 2.30 (t, *J* = 7.5 Hz, 2H), 1.80-1.72 (m, 2H), 1.70-1.59 (m, 4H), 1.54-1.45 (m, 2H), 1.39-1.32 (m, 2H), 0.97 (t, *J* = 7.4 Hz, 3H).

^{13}C NMR (101 MHz, $CDCl_3$) δ 174.2, 157.2, 134.3, 129.1, 114.2, 67.6, 51.4, 34.7, 34.0, 31.4, 31.3, 28.6, 24.8, 19.2, 13.8.

ESI HRMS for [$C_{17}H_{26}O_3+H^+$] calculated: 279.1955, found: 279.1955.

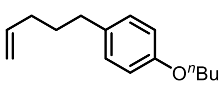

1-butoxy-4-(pent-4-en-1-yl)benzene (13)

Following the representative **Condition A**, colorless oil, yield: 27% (18.0 mg). Flash silica gel chromatography (petroleum ether), *p*:*o* > 50:1.

^{1}H NMR (400 MHz, $CDCl_3$) δ 7.09 (d, *J* = 8.1 Hz, 2H), 6.83 (d, *J* = 8.1 Hz, 2H), 5.90-5.79 (m, 1H), 5.01 (dd, *J* = 20.4, 13.7 Hz, 2H), 3.95 (t, *J* = 6.5 Hz, 2H), 2.57 (t, *J* = 7.7 Hz, 2H), 2.09 (q, *J* = 7.1 Hz, 2H), 1.81-1.65 (m, 4H), 1.56-1.45 (m, 2H), 0.98 (t, *J* = 7.4 Hz, 3H).

^{13}C NMR (101 MHz, $CDCl_3$) δ 157.2, 138.7, 134.3, 129.2, 114.6, 114.3, 67.6, 34.4, 33.2, 31.4, 30.8, 19.3, 13.9.

ESI HRMS for [$C_{15}H_{22}O+H^+$] calculated: 219.1743, found: 219.1745.

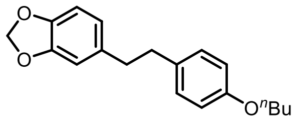

5-(4-butoxyphenethyl)benzo[*d*][1,3]dioxole (14)

Following the representative **Condition A**, white solid, yield: 27% (24.0 mg). Flash silica gel chromatography (petroleum ether/ether acetate = 70:1), *p*:*o* > 50:1.

^{1}H NMR (400 MHz, $CDCl_3$) δ 7.08 (d, *J* = 8.2 Hz, 2H), 6.83 (d, *J* = 8.2 Hz, 2H), 6.73 (d, *J* = 7.9 Hz, 1H), 6.69 (s, 1H), 6.62 (d, *J* = 7.9 Hz, 1H), 5.93 (s, 2H), 3.95 (t, *J* = 6.4 Hz, 2H), 2.82 (s, 4H), 1.82-1.72 (m, 2H), 1.54-1.47 (m, 2H), 0.99 (t, *J* = 7.3 Hz, 3H).

^{13}C NMR (101 MHz, $CDCl_3$) δ 157.4, 147.4, 145.6, 135.7, 133.5, 129.3, 121.2, 114.3, 108.9, 108.0, 100.7, 67.6, 37.9, 37.3, 31.4, 19.2, 13.9.

ESI HRMS for [$C_{19}H_{22}O_3+H^+$] calculated: 299.1642, found: 299.1644.

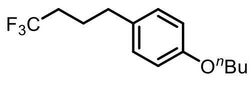

1-butoxy-4-(4,4,4-trifluorobutyl)benzene (15)

Following the representative **Condition A**, colorless oil, yield: 17% (13.3 mg). Flash silica gel chromatography (petroleum ether), *p*:*o* = 12:1.

^{1}H NMR (400 MHz, $CDCl_3$) δ 7.07 (d, *J* = 7.9 Hz, 2H), 6.84 (d, *J* = 7.9 Hz, 2H), 3.94 (t, *J* = 6.3 Hz, 2H), 2.63 (t, *J* = 7.4 Hz, 2H), 2.15-1.99 (m, 2H), 1.91-1.82 (m, 2H), 1.81-1.70 (m, 2H), 1.54-1.43 (m, 2H), 0.98 (t, *J* = 7.3 Hz, 3H).

^{13}C NMR (101 MHz, $CDCl_3$) δ 157.6, 132.5, 129.2, 127.2 (q, *J* = 277.4 Hz,), 114.5, 67.7, 33.7, 33.0 (q, *J* = 28.6 Hz) 31.4, 23.7 (q, *J* = 2.4 Hz), 19.3, 13.8.

^{19}F NMR (376 MHz, $CDCl_3$) δ -66.2.

ESI HRMS for [$C_{14}H_{19}F_3O+H^+$] calculated: 261.1461, found: 261.1461.

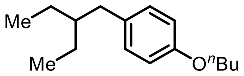

1-butoxy-4-(2-ethylbutyl)benzene (16)

Following the representative **Condition B**, colorless oil, yield: 40% (28.0 mg). Flash silica gel chromatography (petroleum ether), *p*:*o* > 50:1.

^{1}H NMR (400 MHz, $CDCl_3$) δ 7.05 (d, *J* = 8.1 Hz, 2H), 6.81 (d, *J* = 8.1 Hz, 2H), 3.94 (t, *J* = 6.5 Hz, 2H), 2.46 (d, *J* = 7.0 Hz, 2H), 1.80-1.70 (m, 2H), 1.53-1.43 (m, 3H), 1.33-1.23 (m, 4H), 0.97 (t, *J* = 7.4 Hz, 3H), 0.87 (t, *J* = 7.4 Hz, 6H).

^{13}C NMR (101 MHz, $CDCl_3$) δ 157.1, 133.7, 130.0, 114.1, 67.6, 42.7, 38.7, 31.4, 24.9, 19.3, 13.9, 10.8.

ESI HRMS for [$C_{16}H_{26}O+H^+$] calculated: 235.2056, found: 235.2056.

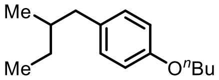

1-butoxy-4-(2-methylbutyl)benzene (17)

Following the representative **Condition B**, colorless oil, yield: 42% (27.6 mg). Flash silica gel chromatography (petroleum ether), *p*:*o* > 50:1.

^{1}H NMR (400 MHz, $CDCl_3$) δ 7.05 (d, *J* = 7.9 Hz, 2H), 6.82 (d, *J* = 7.9 Hz, 2H), 3.95 (t, *J* = 6.5 Hz, 2H), 2.57 (dd, *J* = 13.5, 8.0 Hz, 1H), 2.31 (dd, *J* = 13.5, 8.0 Hz, 1H), 1.81-1.72 (m, 2H), 1.60 (d, *J* = 5.3 Hz, 1H), 1.54-1.46 (m, 2H), 1.39 (d, *J* = 5.3 Hz, 1H), 1.20-1.10 (m, 1H), 0.99 (t, *J* = 7.4 Hz, 3H), 0.91 (t, *J* = 7.4 Hz, 3H), 0.85 (d, *J* = 6.6 Hz, 3H).

^{13}C NMR (101 MHz, $CDCl_3$) δ 157.2, 133.5, 130.0, 114.0, 67.6, 42.4, 36.8, 31.4, 29.1, 19.3, 18.9, 13.9, 11.5.

ESI HRMS for [$C_{15}H_{24}O+H^+$] calculated: 221.1900, found: 221.1900.

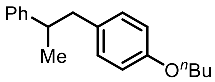

1-butoxy-4-(2-phenylpropyl)benzene (18) [3]

Following the representative **Condition B**, colorless oil, yield: 46% (37.1 mg). Flash silica gel chromatography (petroleum ether), *p*:*o* > 50:1.

^{1}H NMR (400 MHz, $CDCl_3$) δ 7.36-7.29 (m, 2H), 7.25-7.18 (m, 3H), 7.01 (d, *J* = 7.1 Hz, 2H), 6.81 (d, *J* = 7.1 Hz, 2H), 3.96 (t, *J* = 6.5 Hz, 2H), 3.04-2.86 (m, 2H), 2.79-2.70 (m, 1H), 1.84-1.74 (m, 2H), 1.57-1.46 (m, 2H), 1.27 (d, *J* = 6.7 Hz, 3H), 1.01 (t, *J* = 6.7 Hz, 3H).

^{13}C NMR (101 MHz, $CDCl_3$) δ 157.3, 147.1, 132.7, 130.0, 128.2, 127.1, 125.9, 114.1, 67.6, 44.1, 42.0, 31.4, 21.1, 19.3, 13.9.

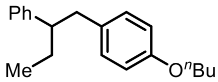

1-butoxy-4-(2-phenylbutyl)benzene (19)

Following the representative **Condition B**, colorless oil, yield: 33% (28.0 mg). Flash silica gel chromatography (petroleum ether), *p*:*o* > 50:1.

^{1}H NMR (400 MHz, $CDCl_3$) δ 7.27 (t, *J* = 7.4 Hz, 2H), 7.18 (t, *J* = 7.2 Hz, 1H), 7.12 (d, *J* = 7.7 Hz, 2H), 6.94 (d, *J* = 8.2 Hz, 2H), 6.75 (d, *J* = 8.2 Hz, 2H), 3.92 (t, *J* = 6.5 Hz, 2H), 2.83 (d, *J* = 7.3 Hz, 2H), 2.72-2.62 (m, 1H), 1.81-1.57 (m, 4H), 1.53-1.43 (m, 2H), 0.98 (t, *J* = 7.4 Hz, 3H), 0.77 (t, *J* = 7.3 Hz, 3H).

^{13}C NMR (101 MHz, $CDCl_3$) δ 157.2, 145.2, 132.7, 130.0, 128.1, 127.8, 125.9, 114.0, 67.6, 50.0, 42.6, 31.4, 28.2, 19.3, 13.9, 12.1.

ESI HRMS for [$C_{20}H_{26}O+H^+$] calculated: 283.2056, found: 283.2057.

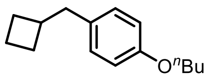

1-butoxy-4-(cyclobutylmethyl)benzene (20)

Following the representative **Condition B**, colorless oil, yield: 61% (39.8 mg). Flash silica gel chromatography (petroleum ether), *p*:*o* > 50:1.

^{1}H NMR (400 MHz, $CDCl_3$) δ 7.05 (d, *J* = 8.0 Hz, 2H), 6.82 (d, *J* = 8.0 Hz, 2H), 3.95 (t, *J* = 6.5 Hz, 2H), 2.64 (d, *J* = 7.5 Hz, 2H), 2.59-2.49 (m, 1H), 2.08-2.00 (m, 2H), 1.89-1.79 (m, 2H), 1.80-1.67 (m, 4H), 1.55-1.45 (m, 2H), 0.99 (t, *J* = 7.4 Hz, 3H).

^{13}C NMR (101 MHz, $CDCl_3$) δ 157.2, 133.2, 129.3, 114.2, 67.6, 42.1, 37.4, 31.4, 28.1, 19.3, 18.3, 13.9.

ESI HRMS for [$C_{15}H_{22}O+H^+$] calculated: 219.1743, found: 219.1742.

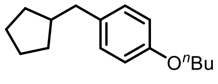

1-butoxy-4-(cyclopentylmethyl)benzene (21)

Following the representative **Condition B**, colorless oil, yield: 54% (37.7 mg). Flash silica gel chromatography (petroleum ether), *p*:*o* > 50:1.

^{1}H NMR (400 MHz, $CDCl_3$) δ 7.08 (d, *J* = 7.7 Hz, 2H), 6.82 (d, *J* = 7.7 Hz, 2H), 3.94 (t, *J* = 6.4 Hz, 2H), 2.55 (d, *J* = 7.4 Hz, 2H), 2.10-2.00 (m, 1H), 1.81-1.73 (m, 2H), 1.73-1.61 (m, 4H), 1.57-1.46 (m, 4H), 1.24-1.14 (m, 2H), 0.98 (t, *J* = 7.3 Hz, 3H).

^{13}C NMR (101 MHz, $CDCl_3$) δ 157.1, 134.3, 129.6, 114.1, 67.6, 42.2, 41.2, 32.4, 31.4, 24.9, 19.3, 13.9.

ESI HRMS for [$C_{16}H_{24}O+H^+$] calculated: 233.1900, found: 233.1900.

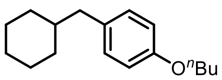

1-butoxy-4-(cyclohexylmethyl)benzene (22)

Following the representative **Condition B**, colorless oil, yield: 47% (34.7 mg). Flash silica gel chromatography (petroleum ether), *p*:*o* > 50:1.

^{1}H NMR (400 MHz, $CDCl_3$) δ 7.04 (d, *J* = 7.9 Hz, 2H), 6.82 (d, *J* = 7.9 Hz, 2H), 3.95 (t, *J* = 6.5 Hz, 2H), 2.42 (d, *J* = 7.1 Hz, 2H), 1.81-1.73 (m, 2H), 1.73-1.62 (m, 5H), 1.55-1.45 (m, 3H), 1.23-1.13 (m, 3H), 0.98 (t, *J* = 7.4 Hz, 3H), 0.95-0.87 (m, 2H).

^{13}C NMR (101 MHz, $CDCl_3$) δ 157.2, 133.2, 129.9, 114.0, 67.6, 43.2, 39.9, 33.1, 31.4, 26.6, 26.3, 19.3, 13.9.

ESI HRMS for [$C_{17}H_{26}O+H^+$] calculated: 247.2056, found: 247.2057.

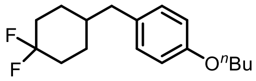

1-butoxy-4-((4,4-difluorocyclohexyl)methyl)benzene (23)

Following the representative **Condition B**, colorless oil, yield: 53% (45.0 mg). Flash silica gel chromatography (petroleum ether), *p*:*o* > 50:1.

^{1}H NMR (400 MHz, $CDCl_3$) δ 7.05 (d, *J* = 7.6 Hz, 2H), 6.84 (d, *J* = 7.6 Hz, 2H), 3.95 (t, *J* = 6.4 Hz, 2H), 2.49 (d, *J* = 7.1 Hz, 2H), 2.12-2.00 (m, 2H), 1.84-1.65 (m, 6H), 1.64-1.61 (m, 1H), 1.61-1.43 (m, 4H), 0.99 (t, *J* = 7.4 Hz, 3H).

^{13}C NMR (101 MHz, $CDCl_3$) δ 157.5, 132.3, 129.8, 123.8 (t, *J* = 240.4 Hz), 114.2, 67.6, 41.4, 38.0, 33.5 (t, *J* = 22.2 Hz), 31.4, 28.7, 19.2, 13.8.

^{19}F NMR (376 MHz, $CDCl_3$) δ -91.5 (d, *J* = 234.8 Hz), -101.83 (d, *J* = 234.8 Hz).

ESI HRMS for [$C_{17}H_{24}F_2O+H^+$] calculated: 283.1868, found: 283.1858.

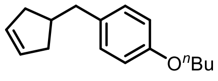

1-butoxy-4-(cyclopent-3-en-1-ylmethyl)benzene (24)

Following the representative **Condition B**, colorless oil, yield: 48% (33.2 mg). Flash silica gel chromatography (petroleum ether), *p*:*o* > 50:1.

^{1}H NMR (400 MHz, $CDCl_3$) δ 7.09 (d, *J* = 8.2 Hz, 2H), 6.82 (d, *J* = 8.2 Hz, 2H), 5.68 (s, 2H), 3.94 (t, *J* = 6.4 Hz, 2H), 2.62 (d, *J* = 7.4 Hz, 2H), 2.58-2.46 (m, 1H), 2.42 (dd, *J* = 14.6, 7.6 Hz, 2H), 2.06 (dd, *J* = 14.6, 4.3 Hz, 2H), 1.82-1.72 (m, 2H), 1.54-1.44 (m, 2H), 0.98 (t, *J* = 7.3 Hz, 3H).

^{13}C NMR (101 MHz, $CDCl_3$) δ 157.3, 133.7, 129.8, 129.6, 114.2, 67.6, 41.4, 39.1, 38.5, 31.4, 19.3, 13.9.

ESI HRMS for [$C_{16}H_{22}O+H^+$] calculated: 231.1743, found: 231.1743.

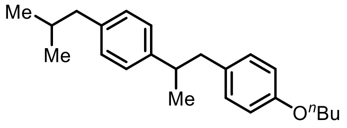

1-butoxy-4-(2-(4-isobutylphenyl)propyl)benzene (25)

Following the representative **Condition B**, colorless oil, yield: 40% (38.8 mg). Flash silica gel chromatography (petroleum ether), *p*:*o* > 50:1.

^{1}H NMR (400 MHz, $CDCl_3$) δ 7.16-7.06 (m, 4H), 7.01 (d, *J* = 8.1 Hz, 2H), 6.81 (d, *J* = 8.1 Hz, 2H), 3.96 (t, *J* = 6.5 Hz, 2H), 3.03-2.85 (m, 2H), 2.78-2.66 (m, 1H), 2.48 (d, *J* = 7.1 Hz, 2H), 1.93-1.85 (m, 1H), 1.83-1.75 (m, 2H), 1.57-1.47 (m, 2H), 1.25 (d, *J* = 6.5 Hz, 3H), 1.01 (t, *J* = 7.4 Hz, 3H), 0.94 (d, *J* = 6.6 Hz, 6H).

^{13}C NMR (101 MHz, $CDCl_3$) δ 157.3, 144.3, 139.2, 132.9, 130.0, 128.9, 126.7, 114.0, 67.6, 45.0, 44.3, 41.6, 31.4, 30.2, 22.4, 21.0, 19.3, 13.9.

ESI HRMS for [$C_{23}H_{32}O+H^+$] calculated: 325.2526, found:325.2509.

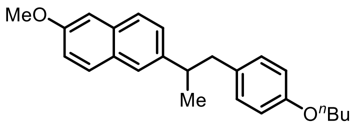

2-(1-(4-butoxyphenyl)propan-2-yl)-6-methoxynaphthalene (26)

Following the representative **Condition B**, white solid, yield: 37% (38.4 mg). Flash silica gel chromatography (petroleum ether/ethyl acetate = 100/1), *p*:*o* > 50:1.

^{1}H NMR (400 MHz, $CDCl_3$) δ 7.68 (d, *J* = 7.8 Hz, 2H), 7.53 (s, 1H), 7.33 (d, *J* = 8.5 Hz, 1H), 7.13 (d, *J* = 7.3 Hz, 2H), 7.00 (d, *J* = 8.2 Hz, 2H), 6.77 (d, *J* = 8.2 Hz, 2H), 3.97-3.87 (m, 5H), 3.15-3.05 (m, 1H), 2.98 (dd, *J* = 13.5, 8.1 Hz, 1H), 2.79 (dd, *J* = 13.4, 8.1 Hz, 1H), 1.80-1.70 (m 2H), 1.53-1.45 (m, 2H), 1.32 (d, 3H), 0.98 (t, *J* = 7.4 Hz, 3H).

^{13}C NMR (101 MHz, $CDCl_3$) δ 157.3, 157.2, 142.3, 133.1, 132.7, 130.0, 129.1, 129.0, 126.6, 126.5, 125.0, 118.5, 114.1, 105.6, 67.6, 55.3, 44.1, 41.9, 31.4, 21.2, 19.2, 13.9.

ESI HRMS for [$C_{24}H_{28}O_2+H^+$] calculated: 349.2162, found: 349.2161.

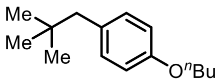

1-butoxy-4-neopentylbenzene (27)

Following the representative **Condition C**, colorless oil, yield: 11% (7.5 mg). Flash silica gel chromatography (petroleum ether), *p*:*o* > 50:1.

^{1}H NMR (400 MHz, $CDCl_3$) δ 7.02 (d, *J* = 8.3 Hz, 2H), 6.80 (d, *J* = 8.3 Hz, 2H), 3.94 (t, *J* = 6.5 Hz, 2H), 2.42 (s, 2H), 1.81-1.71 (m, 2H), 1.54-1.44 (m, 2H), 0.97 (t, *J* = 7.3 Hz, 3H), 0.88 (s, 9H).

^{13}C NMR (101 MHz, $CDCl_3$) δ 157.3, 131.7, 131.3, 113.6, 67.6, 49.3, 31.7, 31.4, 29.3, 19.3, 13.9.

ESI HRMS for [$C_{15}H_{24}O+H^+$] calculated: 221.1900, found: 221.1901.

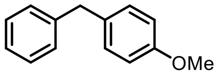

1-benzyl-4-methoxybenzene (28) [4]

Following the representative **Condition C**, colorless oil, yield: 55% (32.7 mg). Flash silica gel chromatography (petroleum ether), *p*:*o* > 50:1.

^{1}H NMR (400 MHz, $CDCl_3$) δ 7.31-7.25 (m, 2H), 7.22-7.15 (m, 3H), 7.11 (d, *J* = 8.0 Hz, 2H), 6.84 (d, *J* = 8.0 Hz, 2H), 3.93 (s, 2H), 3.79 (s, 3H).

^{13}C NMR (101 MHz, $CDCl_3$) δ 157.9, 141.6, 133.2, 129.8, 128.8, 128.4, 126.0, 113.8, 55.2, 41.0.

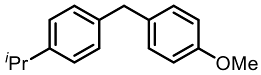

1-isopropyl-4-(4-methoxybenzyl)benzene (29) [5]

Following the representative **Condition C**, colorless oil, yield: 53% (38.0 mg). Flash silica gel chromatography (petroleum ether), *p*:*o* > 50:1.

^{1}H NMR (400 MHz, $CDCl_3$) δ 7.20-7.08 (m, 6H), 6.86 (d, *J* = 8.1 Hz, 2H), 3.90 (s, 2H), 3.79 (s, 3H), 2.95-2.85 (m, 1H), 1.24 (d, *J* = 6.8 Hz, 6H).

^{13}C NMR (101 MHz, $CDCl_3$) δ 157.9, 146.5, 138.9, 133.5, 129.8, 128.6, 126.4, 113.8, 55.2, 40.6, 33.7, 24.0.

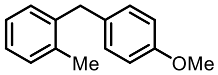

1-(4-methoxybenzyl)-2-methylbenzene (30) [6]

Following the representative **Condition C**, colorless oil, yield: 78% (49.8 mg). Flash silica gel chromatography (petroleum ether), *p*:*o* > 50:1.

¹H NMR (400 MHz, $CDCl_3$) δ 7.20-7.14 (m, 3H), 7.13-7.09 (m, 1H), 7.06 (d, *J* = 8.2 Hz, 2H), 6.84 (d, *J* = 8.2 Hz, 2H), 3.94 (s, 2H), 3.79 (s, 3H), 2.26 (s, 3H).

¹³C NMR (101 MHz, $CDCl_3$) δ 157.8, 139.3, 136.5, 132.4, 130.2, 129.7, 129.6, 126.3, 125.9, 113.8, 55.2, 38.5, 19.6.

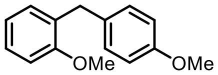

1-methoxy-2-(4-methoxybenzyl)benzene (31) [7]

Following the representative **Condition C**, colorless oil, yield: 59% (40.3 mg). Flash silica gel chromatography (petroleum ether/ethyl acetate = 80/1), *p*:*o* > 50:1.

¹H NMR (400 MHz, $CDCl_3$) δ 7.30-7.23 (m, 1H), 7.21 (d, *J* = 8.1 Hz, 2H), 7.13 (d, *J* = 7.5 Hz, 1H), 6.98-6.91 (m 2H), 6.89 (d, *J* = 8.1 Hz, 2H), 3.99 (s, 2H), 3.88 (s, 3H), 3.84 (s, 3H).

¹³C NMR (101 MHz, $CDCl_3$) δ 157.7, 157.2, 133.0, 130.1, 130.0, 129.8, 127.2, 120.4, 113.6, 110.3, 55.3, 55.2, 34.9.

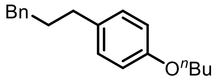

1-butoxy-4-(3-phenylpropyl)benzene (32)

Following the representative **Condition A**, colorless oil, yield: 88% (71.0 mg). Flash silica gel chromatography (petroleum ether), *p*:*o* > 50:1.

¹H NMR (400 MHz, $CDCl_3$) δ 7.34-7.26 (m, 2H), 7.21 (m, 3H), 7.11 (d, *J* = 8.3 Hz, 2H), 6.85 (d, *J* = 8.3 Hz, 2H), 3.96 (t, *J* = 6.5 Hz, 2H), 2.64 (m, 4H), 2.00-1.90 (m, 2H), 1.84-1.73 (m, 2H), 1.56-1.47 (m, 2H), 1.00 (t, *J* = 7.4 Hz, 3H).

¹³C NMR (101 MHz, $CDCl_3$) δ 157.3, 142.4, 134.1, 129.2, 128.4, 128.2, 125.7, 114.3, 67.6, 35.4, 34.5, 33.2, 31.4, 19.3, 13.9.

ESI HRMS for [$C_{19}H_{24}O+H^+$] calculated: 269.1900, found: 269.1900.

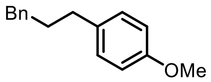

1-methoxy-4-(3-phenylpropyl)benzene (33) [8]

Following the representative **Condition A**, colorless oil, yield: 62% (42.2 mg). Flash silica gel chromatography (petroleum ether), *p*:*o* > 50:1.

¹H NMR (400 MHz, $CDCl_3$) δ 7.32-7.25 (m, 2H), 7.21-7.16 (m, 3H), 7.11 (d, *J* = 8.0 Hz, 2H), 6.84 (d, *J* = 8.0 Hz, 2H), 3.79 (s, 3H), 2.67-2.57 (m, 4H), 1.97-1.87 (m 2H).

¹³C NMR (101 MHz, $CDCl_3$) δ 157.7, 142.3, 134.3, 129.3, 128.4, 128.3, 125.7, 113.7, 55.2, 35.3, 34.5, 33.2.

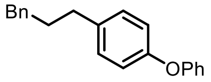

1-phenoxy-4-(3-phenylpropyl)benzene (34) [9]

Following the representative **Condition D**, colorless oil, yield: 36% (30.9 mg). Flash silica gel chromatography (petroleum ether), *p*:*o* > 50:1.

^{1}H NMR (400 MHz, $CDCl_3$) δ 7.39-7.31 (m, 3H), 7.31-7.27 (m 1H), 7.25-7.21 (m 3H), 7.18 (d, *J* = 8.1 Hz, 2H), 7.14-7.08 (m, 1H), 7.02 (d, *J* = 8.1 Hz, 2H), 6.97 (d, *J* = 8.1 Hz, 2H), 2.74-2.64 (m, 4H), 2.04-1.94 (m, 2H).

^{13}C NMR (101 MHz, $CDCl_3$) δ 157.6, 155.0, 142.2, 137.3, 129.7, 129.6, 128.4, 128.3, 125.7, 121.9, 119.0, 118.5, 35.4, 34.7, 33.1.

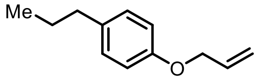

1-(allyloxy)-4-propylbenzene (35) [10]

Following the representative **Condition D**, colorless oil, yield: 57% (30.3 mg). Flash silica gel chromatography (petroleum ether), *p*:*o* > 50:1.

^{1}H NMR (400 MHz, $CDCl_3$) δ 7.08 (d, *J* = 8.3 Hz, 2H), 6.84 (d, *J* = 8.3 Hz, 2H), 6.14-5.99 (m, 1H), 5.41 (d, *J* = 17.3 Hz, 1H), 5.28 (d, *J* = 10.5 Hz, 1H), 4.52 (d, *J* = 5.2 Hz, 2H), 2.53 (t, *J* = 7.6 Hz, 2H), 1.65-1.58 (m, 2H), 0.93 (t, *J* = 7.3 Hz, 3H).

^{13}C NMR (101 MHz, $CDCl_3$) δ 156.6, 135.0, 133.5, 129.3, 117.5, 114.5, 68.9, 37.1, 24.8, 13.8.

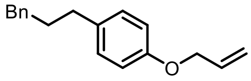

1-(allyloxy)-4-(3-phenylpropyl)benzene (36)

Following the representative **Condition D**, colorless oil, yield: 67% (50.8 mg). Flash silica gel chromatography (petroleum ether/ethyl acetate = 100/1), *p*:*o* > 50:1.

^{1}H NMR (400 MHz, $CDCl_3$) δ 7.35-7.27 (m, 2H), 7.25-7.19 (m, 3H), 7.13 (d, *J* = 8.0 Hz, 2H), 6.88 (d, *J* = 8.0 Hz, 2H), 6.15-6.05 (m, 1H), 5.45 (d, *J* = 17.2 Hz, 1H), 5.32 (d, *J* = 10.5 Hz, 1H), 4.55 (d, *J* = 5.3 Hz, 2H), 2.72-2.59 (m, 4H), 2.02-1.92 (m, 2H).

^{13}C NMR (101 MHz, $CDCl_3$) δ 156.7, 142.3, 134.5, 133.5, 129.3, 128.4, 128.3, 125.7, 117.5, 114.5, 68.8, 35.4, 34.5, 33.1.

ESI HRMS for [$C_{18}H_{20}O+H^+$] calculated: 253.1587, found: 253.1588.

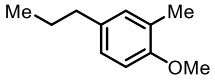

1-methoxy-2-methyl-4-propylbenzene (37) [11]

Following the representative **Condition D**, colorless oil, yield: 56% (27.6 mg). Flash silica gel chromatography (petroleum ether), *p*:*o* > 50:1.

^{1}H NMR (400 MHz, $CDCl_3$) δ 6.98 (d, *J* = 6.3 Hz, 2H), 6.76 (d, *J* = 8.9 Hz, 1H), 3.82 (s, 3H), 2.51 (t, *J* = 7.6 Hz, 2H), 2.22 (s, 3H), 1.66-1.59 (m, 2H), 0.95 (t, *J* = 7.3 Hz, 3H).

^{13}C NMR (101 MHz, $CDCl_3$) δ 155.8, 134.4, 130.8, 126.3, 126.2, 109.8, 55.3, 37.2, 24.8, 16.2, 13.8.

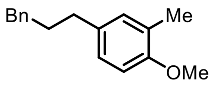

1-methoxy-2-methyl-4-(3-phenylpropyl)benzene (38)

Following the representative **Condition D**, colorless oil, yield: 51% (36.6 mg). Flash silica gel chromatography (petroleum ether), *p*:*o* > 50:1.

^{1}H NMR (400 MHz, $CDCl_3$) δ 7.33-7.27 (m, 2H), 7.24-7.17 (m, 3H), 6.99 (d, *J* = 6.7 Hz, 2H), 6.77 (d, *J* = 8.6 Hz, 1H), 3.83 (s, 3H), 2.67 (t, *J* = 7.7 Hz, 2H), 2.59 (t, *J* = 7.7 Hz, 2H), 2.23 (s, 3H), 2.00-1.90 (m 2H).

^{13}C NMR (101 MHz, $CDCl_3$) δ 155.8, 142.4, 133.9, 130.8 128.4, 128.2, 126.3, 126.3, 125.6, 109.8, 55.3, 35.4, 34.5, 33.2, 16.2.

ESI HRMS for [$C_{17}H_{20}O+H^+$] calculated: 241.1587, found:241.1586.

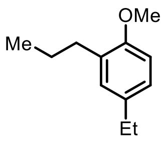

4-ethyl-1-methoxy-2-propylbenzene (39)

Following the representative **Condition D**, colorless oil, yield: 38% (20.2 mg). Flash silica gel chromatography (petroleum ether), *p*:*o* > 50:1.

^{1}H NMR (400 MHz, $CDCl_3$) δ 6.99 (d, *J* = 10.4 Hz, 2H), 6.77 (d, *J* = 8.1 Hz, 1H), 3.80 (s, 3H), 2.63-2.53 (m, 4H), 1.65-1.58 (m, 2H), 1.22 (t, *J* = 7.6 Hz, 3H), 0.96 (t, *J* = 7.3 Hz, 3H).

^{13}C NMR (101 MHz, $CDCl_3$) δ 155.6, 136.0, 130.9, 129.5, 125.7, 110.2, 55.4, 32.4, 28.0, 23.1, 15.9, 14.2.

ESI HRMS for [$C_{12}H_{18}O+H^+$] calculated: 179.1430, found:179.1431.

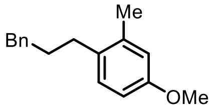

4-methoxy-2-methyl-1-(3-phenylpropyl)benzene (40)

Following the representative **Condition E**, colorless oil, yield: 40% (28.5 mg). Flash silica gel chromatography (petroleum ether), *p*:*o* = 2.2:1.

^{1}H NMR (400 MHz, $CDCl_3$) δ 7.31-7.24 (m, 2H), 7.23-7.14 (m, 3H), 7.01 (d, *J* = 7.4 Hz, 1H), 6.74-6.63 (m, 2H), 3.80 (s, 3H), 2.70-2.60 (m, 4H), 2.34 (s, 3H), 1.96-1.86 (m, 2H).

^{13}C NMR (101 MHz, $CDCl_3$) δ 157.3, 142.7, 136.7, 129.5, 128.4, 128.2, 127.6, 125.5, 120.8, 111.2, 55.2, 35.7, 31.4, 29.6, 21.4.

ESI HRMS for [$C_{17}H_{20}O+H^+$] calculated: 241.1587, found: 241.1588.

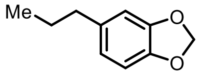

5-propylbenzo[*d*][1,3]dioxole (41) [12]

Following the representative **Condition D**, colorless oil, yield: 65% (31.8 mg). Flash silica gel chromatography (petroleum ether), *p*:*o* > 50:1.

^{1}H NMR (400 MHz, $CDCl_3$) δ 6.73 (d, *J* = 7.9 Hz, 1H), 6.68 (s, 1H), 6.62 (d, *J* = 7.9 Hz, 1H), 5.91 (s, 2H), 2.51 (t, *J* = 7.6 Hz, 2H), 1.62-1.56 (m, 2H), 0.93 (t, *J* = 7.3 Hz, 3H).

^{13}C NMR (101 MHz, $CDCl_3$) δ 147.4, 145.4, 136.6, 121.1, 108.9, 108.0, 100.7, 37.8, 24.8, 13.7.

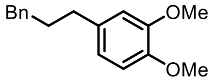

1,2-dimethoxy-4-(3-phenylpropyl)benzene (42) [13]

Following the representative **Condition E**, colorless oil, yield: 59% (45.5 mg). Flash silica gel chromatography (petroleum ether/ethyl acetate =60/1), *p*:*o* > 50:1.

^{1}H NMR (400 MHz, $CDCl_3$) δ 7.32-7.25 (m, 2H), 7.22-7.17 (m, 3H), 6.80 (d, *J* = 8.0 Hz, 1H), 6.76-6.69 (m, 2H), 3.87 (d, 6H), 2.69-2.59 (m, 4H), 2.00-1.90 (m, 2H).

^{13}C NMR (101 MHz, $CDCl_3$) δ 148.7, 147.0, 142.3, 134.9, 128.4, 128.3, 125.7, 120.2, 111.7, 111.1, 55.9, 55.8, 35.4, 35.0, 33.1.

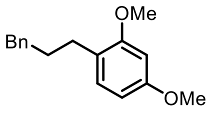

2,4-dimethoxy-1-(3-phenylpropyl)benzene (43) [14]

Following the representative **Condition E**, colorless oil, yield: 55% (42.0 mg). Flash silica gel chromatography (petroleum ether).

^{1}H NMR (400 MHz, $CDCl_3$) δ 7.32-7.25 (m, 2H), 7.24-7.16 (m, 3H), 7.04 (d, *J* = 8.1 Hz, 1H), 6.48-6.40 (m, 2H), 3.80 (s, 6H), 2.67 (t, *J* = 7.8 Hz, 2H), 2.61 (t, *J* = 7.8 Hz, 2H), 1.96-1.86 (m 2H).

^{13}C NMR (101 MHz, $CDCl_3$) δ 159.0, 158.3, 142.7, 129.9, 128.4, 128.2, 125.5, 123.1, 103.6, 98.4, 55.3, 55.2, 35.7, 31.5, 29.3.

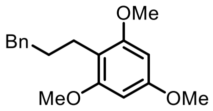

1,3,5-trimethoxy-2-(3-phenylpropyl)benzene (44) [15]

Following the representative **Condition E**, colorless oil, yield: 59% (50.3 mg). Flash silica gel chromatography (petroleum ether/ethyl acetate = 70/1).

^{1}H NMR (400 MHz, $CDCl_3$) δ 7.30-7.25 (m, 2H), 7.24-7.13 (m, 3H), 6.15 (s, 2H), 3.82 (s, 3H), 3.79 (s, 6H), 2.70-2.60 (m, 4H), 1.86-1.76 (m, 2H).

^{13}C NMR (101 MHz, $CDCl_3$) δ 159.1, 158.8, 143.2, 128.4, 128.0, 125.3, 111.5, 90.6, 55.6, 55.3, 35.9, 30.9, 22.5.

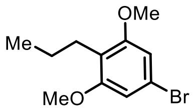

5-bromo-1,3-dimethoxy-2-propylbenzene (45)

Following the representative **Condition D**, white solid, yield: 19% (15.1 mg). Flash silica gel chromatography (petroleum ether), *p*:*o* > 50:1.

^{1}H NMR (400 MHz, $CDCl_3$) δ 6.67 (s, 2H), 3.78 (s, 6H), 2.54 (t, *J* = 7.6 Hz, 2H), 1.50-1.40 (m, 2H), 0.90 (t, *J* = 7.3 Hz, 3H).

^{13}C NMR (101 MHz, $CDCl_3$) δ 158.8, 119.4, 118.5, 107.5, 55.9, 24.7, 22.2, 14.1.

ESI HRMS for [$C_{11}H_{15}BrO_2$+H^+] calculated: 259.0328, found: 259.0328.

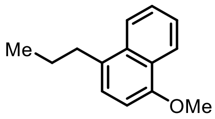

1-methoxy-4-propylnaphthalene (46)

Following the representative **Condition D**, colorless oil, yield: 38% (22.9 mg). Flash silica gel chromatography (petroleum ether), *p*:*o* > 50:1.

^{1}H NMR (400 MHz, $CDCl_3$) δ 8.36 (d, *J* = 8.3 Hz, 1H), 8.03 (d, *J* = 8.3 Hz, 1H), 7.60-7.50 (m 2H), 7.26 (d, *J* = 7.8 Hz, 1H), 6.79 (d, *J* = 7.8 Hz, 1H), 4.03 (s, 3H), 2.97 (t, *J* = 8.0 Hz, 2H), 1.86-1.76 (m, 2H), 1.07 (t, *J* = 7.3 Hz, 3H).

^{13}C NMR (101 MHz, $CDCl_3$) δ 154.0, 132.7, 130.6, 126.1, 125.9, 125.5, 124.7, 123.8, 122.5, 103.4, 55.4, 34.7, 24.0, 14.2.

ESI HRMS for [$C_{14}H_{16}O$+H^+] calculated: 201.1274, found: 201.1275.

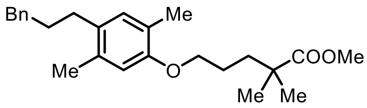

Methyl 5-(2,5-dimethyl-4-(3-phenylpropyl)phenoxy)-2,2-dimethylpentanoate (47)

Following the representative **Condition E**, colorless oil, yield: 34% (39.4 mg). Flash silica gel chromatography (petroleum ether/ethyl acetate = 90/1), *p*:*o* > 50:1.

^{1}H NMR (400 MHz, $CDCl_3$) δ 7.36-7.27 (m, 2H), 7.26-7.20 (m, 3H), 6.93 (s, 1H), 6.62 (s, 1H), 3.94 (t, 2H), 3.71 (s, 3H), 2.73 (t, J = 7.7 Hz, 2H), 2.63-2.54 (m, 2H), 2.26 (s, 3H), 2.21 (s, 3H), 1.97-1.86 (m, 2H), 1.80-1.70 (m, 4H), 1.26 (s, 6H).

^{13}C NMR (101 MHz, $CDCl_3$) δ 178.3, 155.1, 142.4, 133.8, 132.0, 131.2, 128.4, 128.2, 125.7, 123.8, 113.2, 68.1, 51.7, 42.1, 37.1, 35.8, 32.2, 32.1, 25.3, 25.2, 19.2, 15.6.

ESI HRMS for [$C_{25}H_{34}O_3$+Na^+] calculated: 405.2400, found: 405.2396.

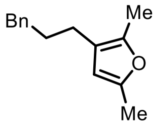

2,5-dimethyl-3-(3-phenylpropyl)furan (48)

Following the representative **Condition F**, colorless oil, yield: 32% (20.5 mg). Flash silica gel chromatography (petroleum ether).

^{1}H NMR (400 MHz, $CDCl_3$) δ 7.34-7.27 (m, 2H), 7.25-7.15 (m, 3H), 5.81 (s, 1H), 2.65 (t, J = 7.4 Hz, 2H), 2.33 (t, J = 7.4 Hz, 2H), 2.25 (s, 3H), 2.17 (s, 3H), 1.90-1.80 (m, 2H).

^{13}C NMR (101 MHz, $CDCl_3$) δ 149.1, 145.1, 142.4, 128.4, 128.2, 125.7, 119.2, 107.3, 35.3, 31.9, 24.4, 13.5, 11.4.

ESI HRMS for [$C_{15}H_{18}O$+H^+] calculated: 215.1430, found: 215.1431.

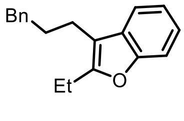

2-ethyl-3-(3-phenylpropyl)benzofuran (49)

Following the representative **Condition A**, colorless oil, yield: 41% (32.7 mg). Flash silica gel chromatography (petroleum ether).

^{1}H NMR (400 MHz, $CDCl_3$) δ 7.50-7.37 (m, 2H), 7.33-7.29 (m, 2H), 7.27-7.17 (m, 5H), 2.82-2.62 (m, 6H), 2.08-1.98 (m 2H), 1.33 (t, J = 7.6 Hz, 3H).

^{13}C NMR (101 MHz, $CDCl_3$) δ 155.7, 154.0, 142.1, 129.7, 128.4, 128.3, 125.8, 122.9, 121.9, 118.9, 113.2, 110.6, 35.6, 31.4, 23.1, 19.8, 13.0.

ESI HRMS for [$C_{19}H_{20}O$+H^+] calculated: 265.1587, found: 265.1585.

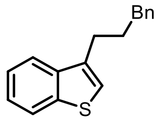

3-(3-phenylpropyl)benzo[b]thiophene (50) [16]

Following the representative **Condition A**, colorless oil, yield: 28% (20.9 mg). Flash silica gel chromatography (petroleum ether).

^{1}H NMR (400 MHz, $CDCl_3$) δ 7.87 (d, *J* = 7.7 Hz, 1H), 7.71 (d, *J* = 7.7 Hz, 1H), 7.41-7.27 (m, 4H), 7.25-7.20 (m, 3H), 7.10 (s, 1H), 2.89 (t, *J* = 7.7 Hz, 2H), 2.76 (t, *J* = 7.7 Hz, 2H), 2.16-2.06 (m, 2H).

^{13}C NMR (101 MHz, $CDCl_3$) δ 142.0, 140.5, 139.0, 136.6, 128.5, 128.4, 125.8, 124.1, 123.8, 122.9, 121.7, 121.0, 35.6, 30.7, 28.0.

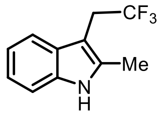

2-methyl-3-(2,2,2-trifluoroethyl)-1H-indole (51) [17]

Following the representative **Condition G**, white solid, yield: 81% (51.9 mg). Flash silica gel chromatography (petroleum ether/ethyl acetate = 10/1).

^{1}H NMR (400 MHz, $CDCl_3$) δ 7.93 (s, 1H), 7.52 (d, *J* = 7.1 Hz, 1H), 7.30 (d, *J* = 8.1 Hz, 1H), 7.19-7.09 (m, 2H), 3.47 (q, *J* = 10.9 Hz, 2H), 2.42 (s, 3H).

^{13}C NMR (101 MHz, $CDCl_3$) δ 135.0, 134.1, 128.7, 126.6 (q, *J* = 277.8 Hz), 121.5, 119.9, 117.9, 110.2, 100.9, 29.5 (q, *J* = 31.3 Hz), 11.6.

^{19}F NMR (376 MHz, $CDCl_3$) δ -66.0.

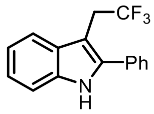

2-phenyl-3-(2,2,2-trifluoroethyl)-1H-indole (52)

Following the representative **Condition G**, yellow oil, yield: 86% (71.3 mg). Flash silica gel chromatography (petroleum ether/ethyl acetate = 15/1).

^{1}H NMR (400 MHz, $CDCl_3$) δ 8.17 (s, 1H), 7.69 (d, *J* = 7.7 Hz, 1H), 7.56 (d, *J* = 7.3 Hz, 2H), 7.50 (t, *J* = 7.3 Hz, 2H), 7.45 (d, *J* = 7.3 Hz, 1H), 7.38 (d, *J* = 7.7 Hz, 1H), 7.30-7.15 (m, 2H), 3.61 (q, *J* = 10.6 Hz, 2H).

^{13}C NMR (101 MHz, $CDCl_3$) δ 137.8, 135.6, 132.0, 129.0, 128.8, 128.5, 126.6 (q, *J* = 279.0 Hz), 122.8, 120.4, 119.5, 119.4, 110.9, 101.8, 30.2 (q, *J* = 31.3 Hz).

^{19}**F NMR** (376 MHz, $CDCl_3$) δ -64.3.

ESI HRMS for [$C_{16}H_{12}F_3N$ +H^+] calculated: 276.0995, found: 276.0996.

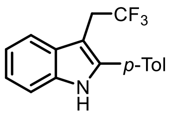

2-(p-tolyl)-3-(2,2,2-trifluoroethyl)-1H-indole (53)

Following the representative **Condition G**, white solid, yield: 76% (66.1 mg). Flash silica gel chromatography (petroleum ether/ethyl acetate = 15/1).

^{1}**H NMR** (400 MHz, $CDCl_3$) δ 8.16 (s, 1H), 7.67 (d, *J* = 7.8 Hz, 1H), 7.46 (d, *J* = 7.8 Hz, 2H), 7.39 (d, *J* = 7.9 Hz, 1H), 7.31 (d, *J* = 7.8 Hz, 2H), 7.28-7.16 (m, 2H), 3.60 (q, *J* = 10.7 Hz, 2H), 2.43 (s, 3H).

^{13}**C NMR** (101 MHz, $CDCl_3$) δ 138.5, 138.0, 135.5, 129.7, 129.1, 128.9, 128.4, 126.7 (q, *J* = 278.7 Hz), 122.6, 120.3, 119.4, 119.3, 110.8, 30.2 (q, *J* = 31.3 Hz)., 21.3.

^{19}**F NMR** (376 MHz, $CDCl_3$) δ -64.4.

ESI HRMS for [$C_{17}H_{14}F_3N$ +H^+] calculated: 290.1151, found: 290.1154.

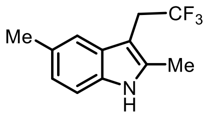

2,5-dimethyl-3-(2,2,2-trifluoroethyl)-1H-indole (54)

Following the representative **Condition G**, white solid, yield: 41% (27.8 mg). Flash silica gel chromatography (petroleum ether/ethyl acetate = 10/1).

^{1}**H NMR** (400 MHz, $CDCl_3$) δ 7.80 (s, 1H), 7.31 (s, 1H), 7.18 (d, *J* = 8.2 Hz, 1H), 6.99 (d, *J* = 8.2 Hz, 1H), 3.45 (q, *J* = 10.9 Hz, 2H), 2.47 (s, 3H), 2.39 (s, 3H).

^{13}**C NMR** (101 MHz, $CDCl_3$) δ 134.2, 133.3, 129.2, 128.9, 126.6 (q, *J* = 277.8 Hz), 123.0, 117.6, 110.0, 100.4, 29.4 (q, *J* = 31.3 Hz), 21.5, 11.6.

^{19}**F NMR** (376 MHz, $CDCl_3$) δ -66.0.

ESI HRMS for [$C_{12}H_{12}F_3N$ +H^+] calculated: 228.0995, found: 228.0994.

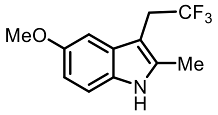

5-methoxy-2-methyl-3-(2,2,2-trifluoroethyl)-1H-indole (55) [17]

Following the representative **Condition G**, white solid, yield: 43% (31.2 mg). Flash silica gel chromatography (petroleum ether/ethyl acetate = 10/1).

^{1}H NMR (400 MHz, $CDCl_3$) δ 7.85 (s, 1H), 7.17 (d, J = 8.7 Hz, 1H), 6.98 (s, 1H), 6.86 -6.79 (m, 1H), 3.88 (s, 3H), 3.45 (q, J = 10.9 Hz, 2H), 2.38 (s, 3H).

^{13}C NMR (101 MHz, $CDCl_3$) δ 154.3, 135.0, 130.0, 129.2, 126.6 (q, J = 278.5 Hz), 111.2, 111.0, 100.7 (q, J = 277.4 Hz,), 100.3 (q, J = 4.0 Hz,), 55.9, 29.5 (q, J = 31.4 Hz), 11.7.

^{19}F NMR (376 MHz, $CDCl_3$) δ -65.9.

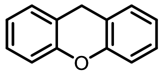

3-(3-phenylpropyl)benzo[b]thiophene (56) [18]

Following the representative **Condition C**, white solid, yield: 31% (16.8 mg). Flash silica gel chromatography (petroleum ether).

^{1}H NMR (400 MHz, $CDCl_3$) δ 7.25-7.15 (m, 4H), 7.10-6.99 (m, 4H), 4.06 (s, 2H).

^{13}C NMR (101 MHz, $CDCl_3$) δ 151.9, 128.9, 127.6, 122.9, 120.5, 116.4, 27.8.

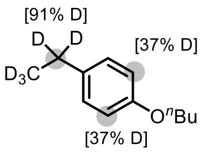

1-butoxy-4-(ethyl-d$_5$)benzene (57)

Following the representative **Condition A**, colorless oil, yield: 78% (42.7 mg). Flash silica gel chromatography (petroleum ether), *p*:*o* > 50:1.

^{1}H NMR (400 MHz, $CDCl_3$) δ 7.11 (d, J = 7.6 Hz, 2H), 3.95 (t, J = 6.2 Hz, 2H), 181-1.71 (m, 2H), 1.56-1.46 (m, 2H), 0.98 (t, J = 7.3 Hz, 3H).

^{13}C NMR (101 MHz, $CDCl_3$) δ 157.2, 136.1, 128.6, 114.3, 67.7, 31.4, 19.3, 13.9.

ESI HRMS for [$C_{12}H_{13}D_5O+H^+$] calculated: 184.1744, found: 184.1743.

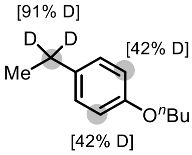

1-butoxy-4-(ethyl-1,1-d$_2$)benzene (58)

Following the representative **Condition A**, colorless oil, yield: 57% (30.9 mg). Flash silica gel chromatography (petroleum ether), *p*:*o* > 50:1.

^{1}H NMR (400 MHz, $CDCl_3$) δ 7.11 (d, J = 8.6 Hz, 2H), 3.95 (t, J = 6.5 Hz, 2H), 1.81-1.69 (m, 2H), 1.56-1.46 (m, 2H), 0.98 (t, J = 7.4 Hz, 3H).

^{13}C NMR (101 MHz, $CDCl_3$) δ 157.1, 136.1, 128.6, 114.4, 67.7, 31.4, 19.3, 15.7, 13.9.

ESI HRMS for [$C_{12}H_{16}D_2O+H^+$] calculated: 181.1556, found: 181.1544.

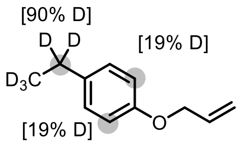

1-(allyloxy)-4-(ethyl-d$_5$)benzene (59)

Following the representative **Condition D**, colorless oil, yield: 25% (12.4 mg), 90% D. Flash silica gel chromatography (petroleum ether), *p*:*o* > 50:1.

^{1}H NMR (400 MHz, $CDCl_3$) δ 7.11 (d, J = 7.6 Hz, 2H), 6.85 (d, J = 8.1 Hz, 2H), 6.14-5.99 (m, 1H), 5.41 (d, J = 17.2 Hz, 1H), 5.28 (d, J = 10.5 Hz, 1H), 4.52 (d, J = 4.4 Hz, 2H).

^{13}C NMR (101 MHz, $CDCl_3$) δ 156.6, 136.5, 133.5, 128.7, 117.5, 114.6, 68.9.

ESI HRMS for [$C_{11}H_9D_5O+H^+$] calculated: 168.1431, found: 168.1431.

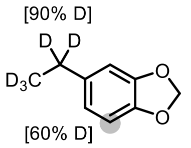

5-(ethyl-d$_5$)benzo[*d*][1,3]dioxole (60)

Following the representative **Condition D**, colorless oil, yield: 23% (10.6 mg), 90% D. Flash silica gel chromatography (petroleum ether).

^{1}H NMR (400 MHz, $CDCl_3$) δ 6.76-6.68 (m, 2H), 5.92 (s, 2H).

^{13}C NMR (101 MHz, $CDCl_3$) δ 147.5, 145.3, 120.4, 108.4, 108.1, 108.0, 100.7.

ESI HRMS for [$C_{13}H_9D_5O+H^+$] calculated: 156.1067, found: 156.1068.

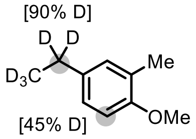

4-(ethyl-d$_5$)-1-methoxy-2-methylbenzene (61)

Following the representative **Condition D**, colorless oil, yield: 42% (19.5 mg), 90% D. Flash silica gel chromatography (petroleum ether), *p*:*o* > 50:1.

^{1}H NMR (400 MHz, $CDCl_3$) δ 6.99 (s, 2H), 6.76 (d, J = 8.0 Hz, 1H), 3.82 (s, 3H), 2.22 (s 3H).

^{13}C NMR (101 MHz, $CDCl_3$) δ 155.7, 135.9, 130.3, 126.2, 125.7, 109.9, 55.4, 16.2.

ESI HRMS for [$C_{10}H_9D_5O+H^+$] calculated: 156.1431, found: 156.1430.

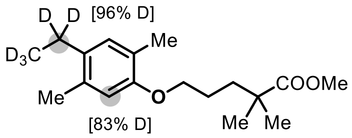

methyl 5-(4-(ethyl-d$_5$)-2,5-dimethylphenoxy)-2,2-dimethylpentanoate (62)

Following the representative **Condition E**, colorless oil, yield: 66% (58.6 mg), 96% D. Flash silica gel chromatography (petroleum ether/ethyl acetate = 90/1), *p*:*o* > 50:1.

^{1}H NMR (400 MHz, $CDCl_3$) δ 6.95 (d, *J* = 40.0 Hz,1H), 3.91 (t, 2H), 3.67 (s, 3H), 2.28 (d, *J* = 24.0 Hz, 3H), 2.17 (s, 3H), 1.79-1.66 (m, 4H), 1.22 (s, 6H).

^{13}C NMR (101 MHz, $CDCl_3$) δ 178.3, 156.9, 154.9, 136.3, 123.9, 123.5, 113.1, 68.1, 67.9, 51.7, 42.1, 37.1, 25.2, 20.2 (d, *J* = 223.2 Hz), 15.7.

ESI HRMS for [$C_{18}H_{23}D_5O_3$+H^+] calculated: 298.2425, found: 298.2425.

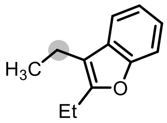

2,3-diethylbenzofuran (63)

Following the representative **Condition A**, colorless oil, yield: 56% (29.1 mg), 90% D. Flash silica gel chromatography (petroleum ether).

^{1}H NMR (400 MHz, $CDCl_3$) δ 7.48 (d, *J* = 6.5 Hz, 1H), 7.39 (d, *J* = 7.5 Hz, 1H), 7.23-7.17 (m, 2H), 2.76 (q, *J* = 7.5 Hz, 2H), 2.65 (q, *J* = 7.6 Hz, 2H), 1.30 (t, *J* = 7.6 Hz, 3H), 1.25 (t, *J* = 7.6 Hz, 3H).

^{13}C NMR (101 MHz, $CDCl_3$) δ 155.1, 153.9, 129.6, 122.9, 121.8, 118.8, 115.2, 110.6, 19.7, 16.8, 14.8, 13.1.

ESI HRMS for [$C_{12}H_{14}O$+H^+] calculated: 175.1117, found: 175.1117.

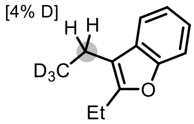

2-ethyl-3-(ethyl-2,2,2-d$_3$)benzofuran (64)

Following the representative **Condition A**, colorless oil, yield: 38% (20.3 mg). Flash silica gel chromatography (petroleum ether).

^{1}H NMR (400 MHz, $CDCl_3$) δ 7.49 (d, *J* = 6.5 Hz, 1H), 7.41 (d, *J* = 7.2 Hz, 1H), 7.25-7.14 (m, 2H), 2.77 (q, *J* = 7.5 Hz, 2H), 2.64 (s, 2H), 1.32 (t, *J* = 7.6 Hz, 3H).

^{13}C NMR (101 MHz, $CDCl_3$) δ 155.1, 153.9, 129.6, 122.9, 121.8, 118.8, 115.1, 110.6, 19.7, 16.6, 13.1.

ESI HRMS for [$C_{12}H_{11}D_3O$+H^+] calculated: 178.1306, found: 178.1306.

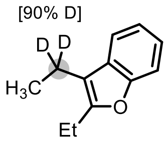

2-ethyl-3-(ethyl-1,1-d_2)benzofuran (65)

Following the representative **Condition A**, colorless oil, yield: 51% (27.0 mg), 90% D. Flash silica gel chromatography (petroleum ether).

^{1}H NMR (400 MHz, $CDCl_3$) δ 7.48 (d, J = 6.5 Hz, 1H), 7.40 (d, J = 7.5 Hz, 1H), 7.24-7.18 (m, 2H), 2.76 (q, J = 7.5 Hz, 2H), 1.31 (t, J = 7.5 Hz, 3H), 1.24 (s, 3H).

^{13}C NMR (101 MHz, $CDCl_3$) δ 155.1, 153.9, 129.6, 122.9, 121.8, 118.8, 115.1, 110.6, 19.7, 14.6, 13.1.

ESI HRMS for [$C_{12}H_{12}D_2O+H^+$] calculated: 177.1243, found: 177.1244.

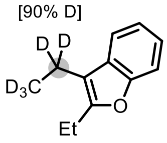

2-ethyl-3-(ethyl-d_5)benzofuran (66)

Following the representative **Condition A**, colorless oil, yield: 47% (25.2 mg), 90% D. Flash silica gel chromatography (petroleum ether).

^{1}H NMR (400 MHz, $CDCl_3$) δ 7.50 (d, J = 6.5 Hz, 1H), 7.42 (d, J = 7.2 Hz, 1H), 7.27-7.15 (m, 2H), 2.78 (q, J = 7.5 Hz, 2H), 1.33 (t, J = 7.4 Hz, 3H).

^{13}C NMR (101 MHz, $CDCl_3$) δ 155.1, 153.9, 129.6, 122.9, 121.8, 118.8, 115.1, 110.6, 19.7, 13.1.

ESI HRMS for [$C_{12}H_9D_5O+H^+$] calculated: 180.1431, found: 180.1435.

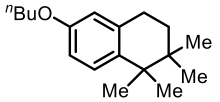

6-butoxy-1,1,2,2-tetramethyl-1,2,3,4-tetrahydronaphthalene (71)

Following the representative **Condition B**, colorless oil. Flash silica gel chromatography (petroleum ether).

^{1}H NMR (400 MHz, $CDCl_3$) δ 6.95 (d, J = 8.3 Hz, 1H), 6.91 (s, 1H), 6.67 (d, J = 8.3 Hz, 1H), 3.94 (t, J = 6.5 Hz, 2H), 2.74 (t, J = 6.8 Hz, 2H), 1.81-1.71 (m, 2H), 1.65 (t, J = 6.8 Hz, 2H), 1.55-1.45 (m, 2H), 1.22 (s, 6H), 0.98 (t, J = 7.4 Hz, 3H), 0.93 (s, 6H).

^{13}C NMR (101 MHz, $CDCl_3$) δ 157.3, 147.9, 129.4, 127.1, 113.2, 111.1, 67.6, 40.0, 34.4, 33.3, 31.5, 26.3, 25.6, 24.5, 19.3, 13.9.

ESI HRMS for [$C_{18}H_{28}O+H^+$] calculated: 261.2213, found: 261.2214.

3.7 NMR spectra of products

^{1}H NMR of Compound 3 (400 MHz, $CDCl_3$)

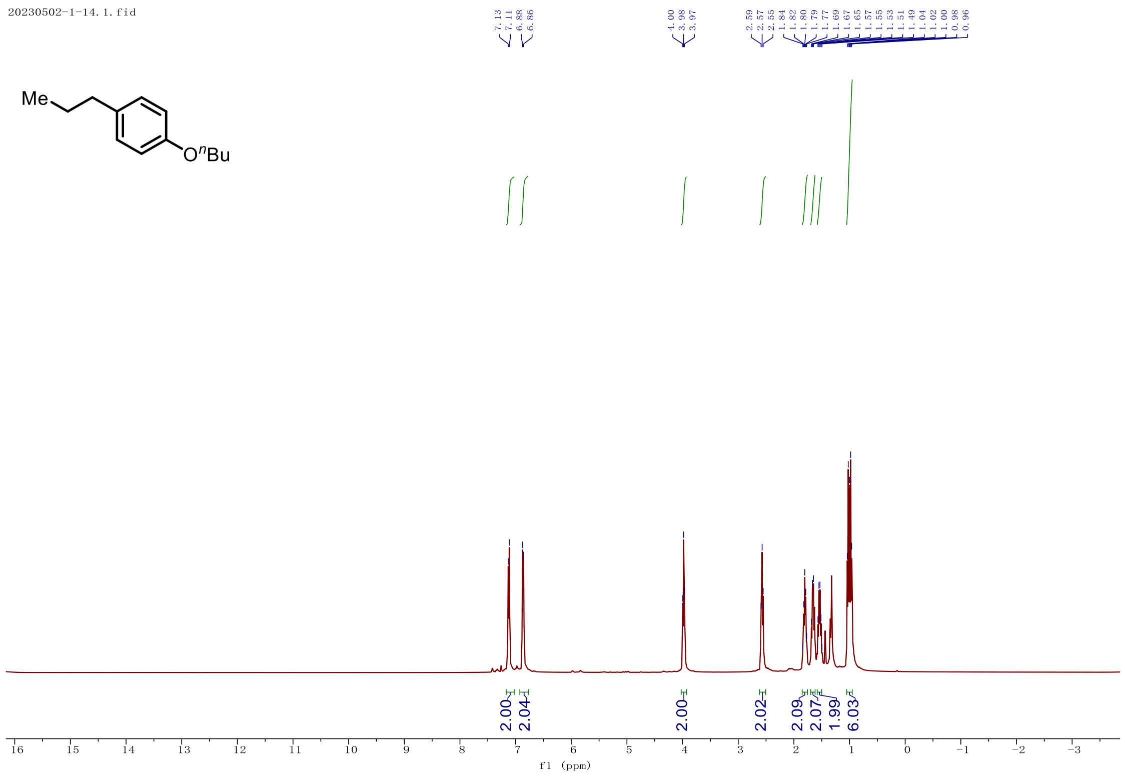

^{13}C NMR of Compound 3 (101 MHz, $CDCl_3$)

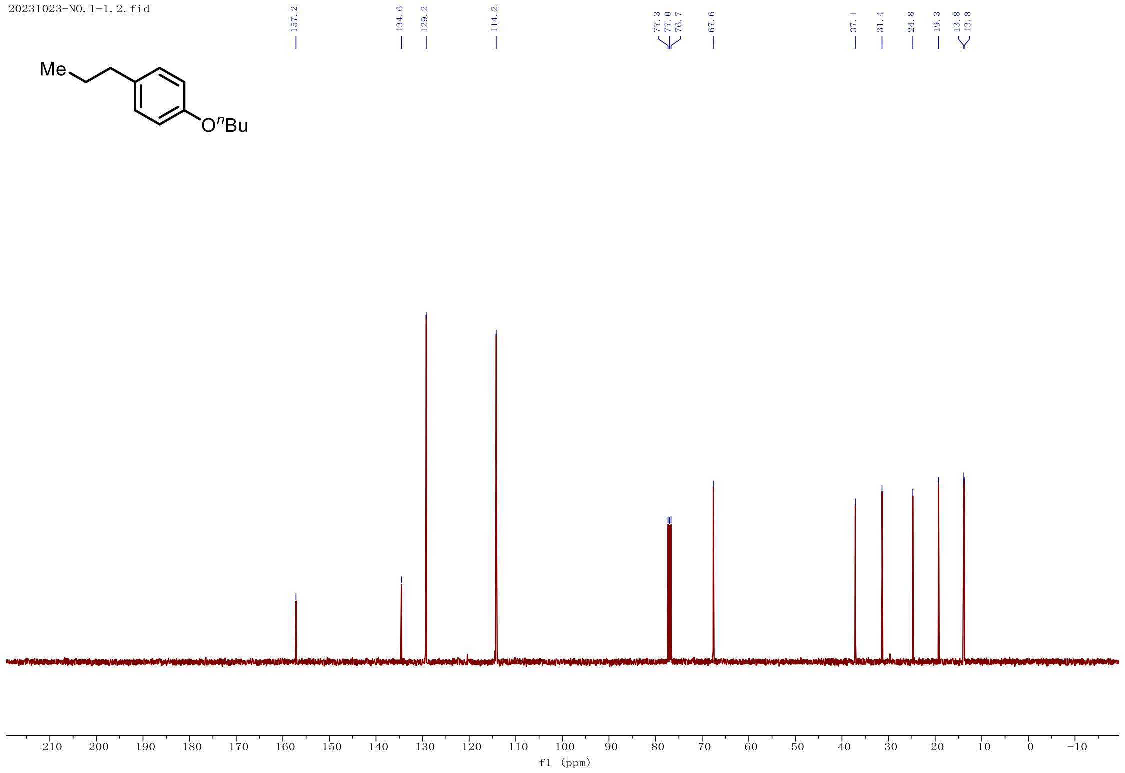

^{1}H NMR of Compound 4 (400 MHz, $CDCl_3$)

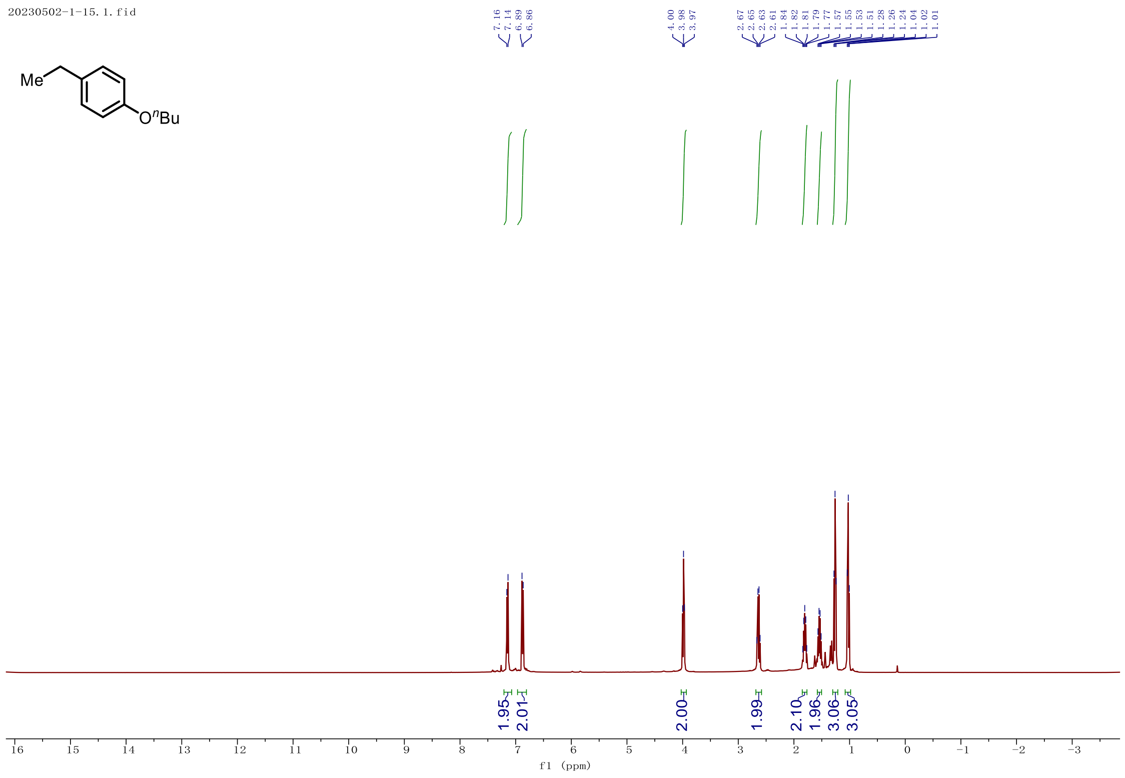

^{13}C NMR of Compound 4 (101 MHz, $CDCl_3$)

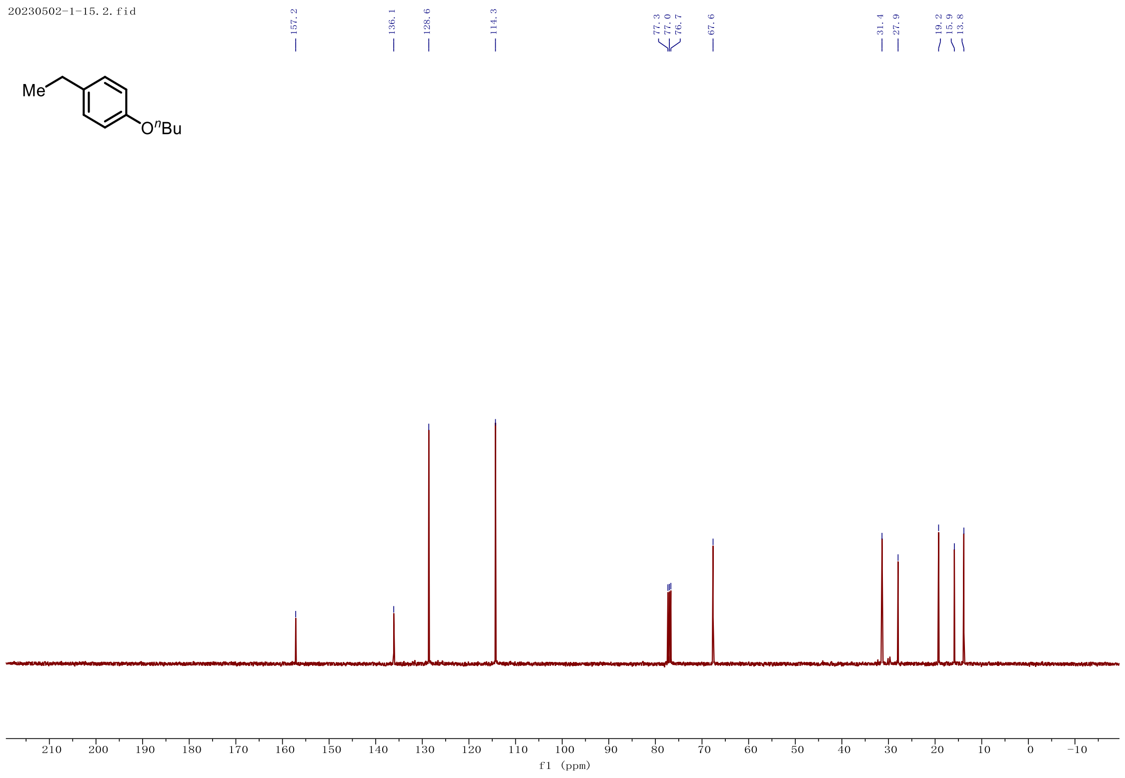

^{1}H NMR of Compound 5 (400 MHz, $CDCl_3$)

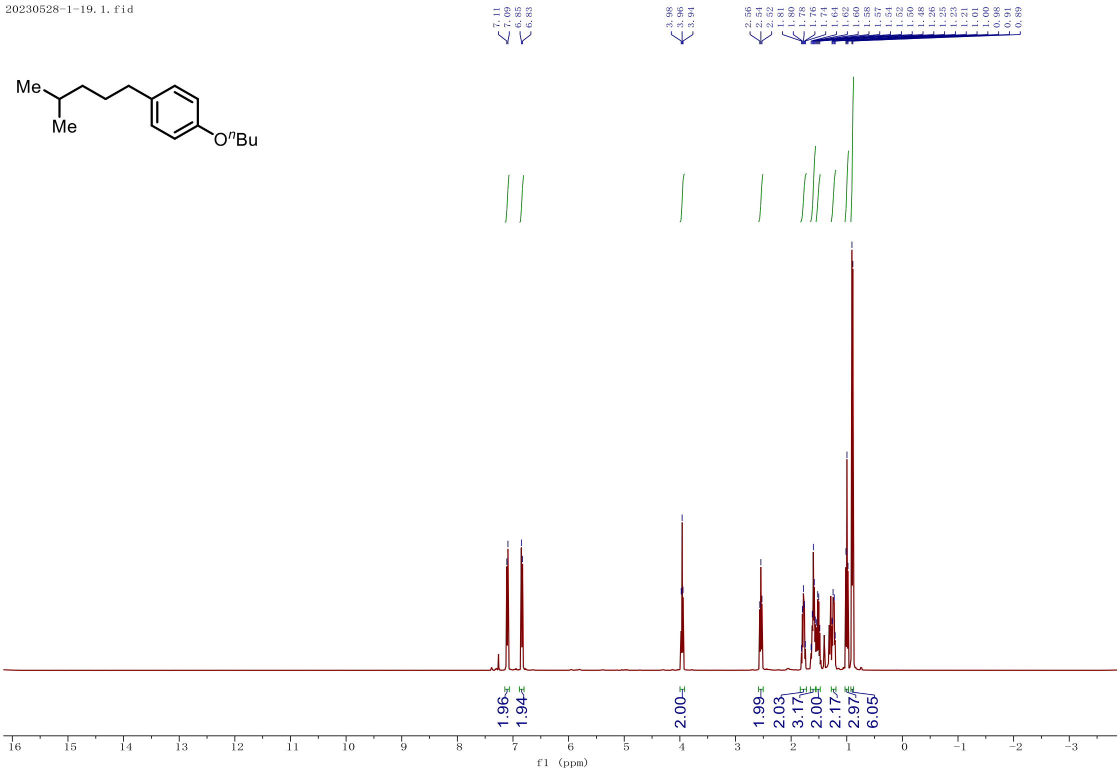

^{13}C NMR of Compound 5 (101 MHz, $CDCl_3$)

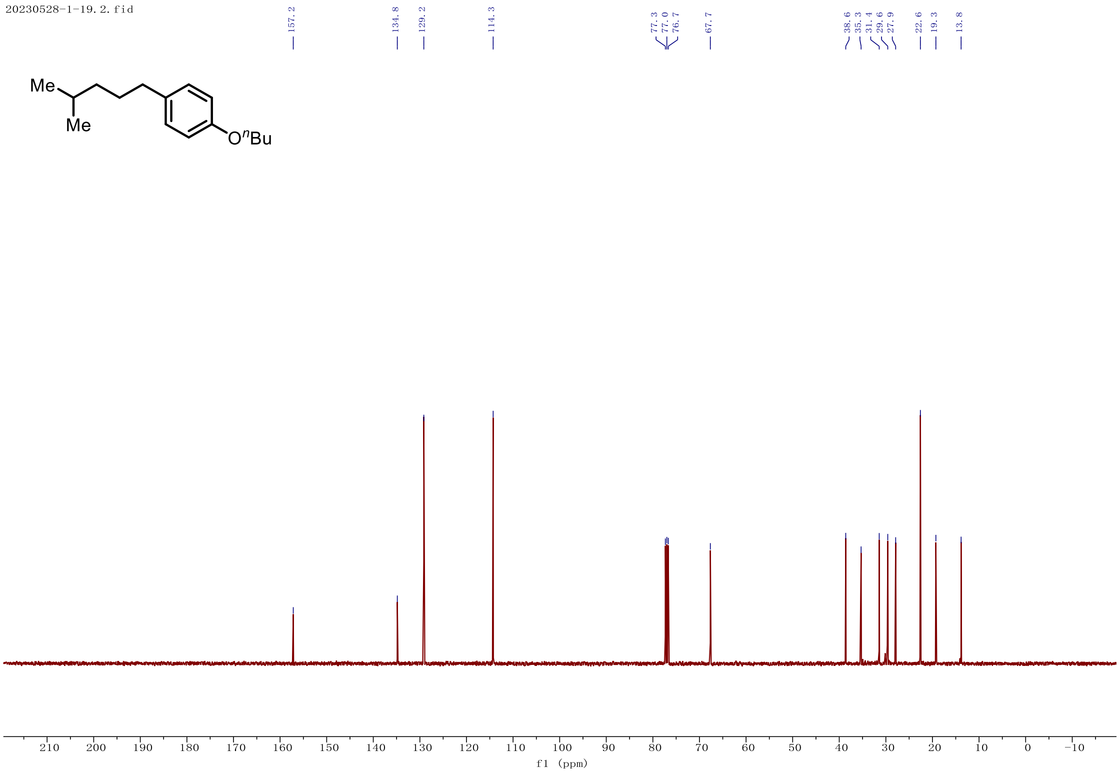

^{1}H NMR of Compound 6 (400 MHz, $CDCl_3$)

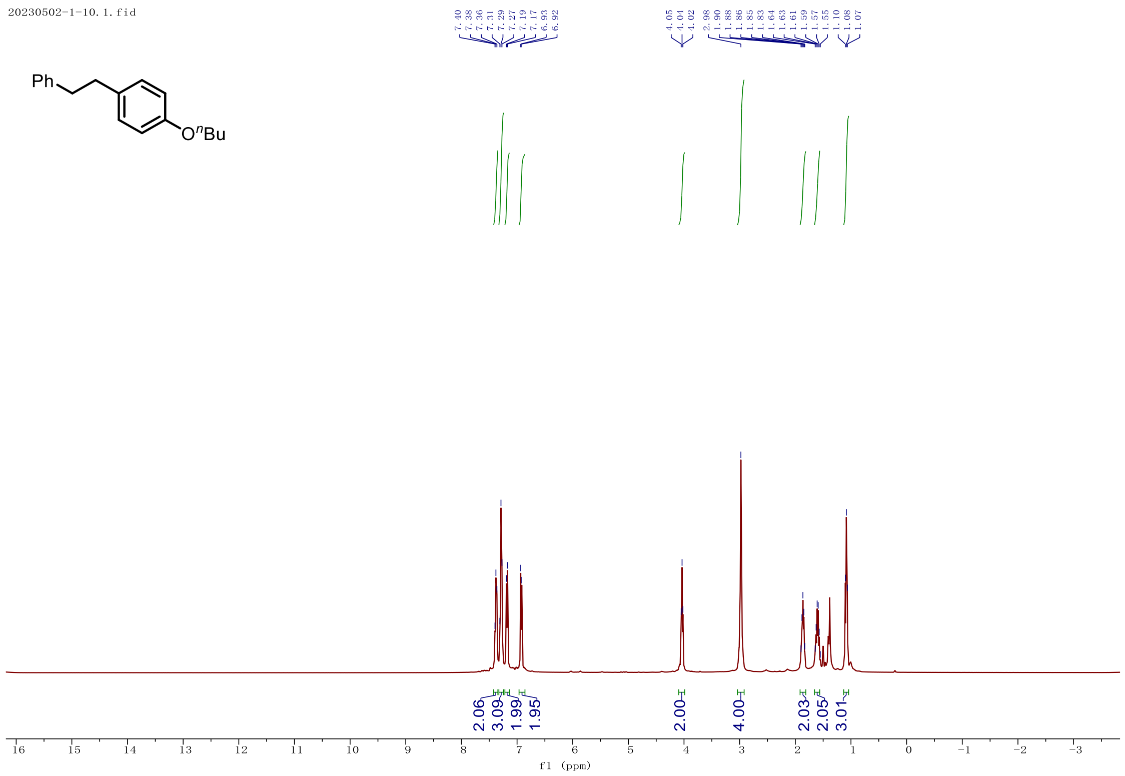

^{13}C NMR of Compound 6 (101 MHz, $CDCl_3$)

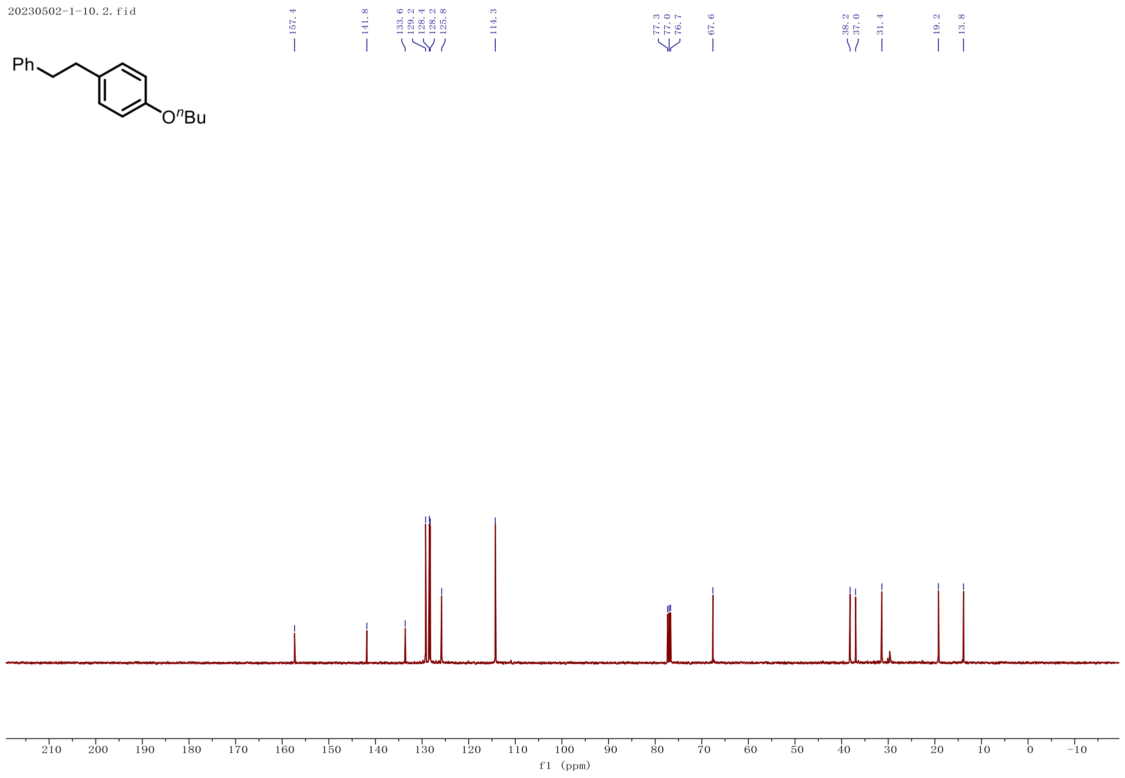

^{1}H NMR of Compound 7 (400 MHz, $CDCl_3$)

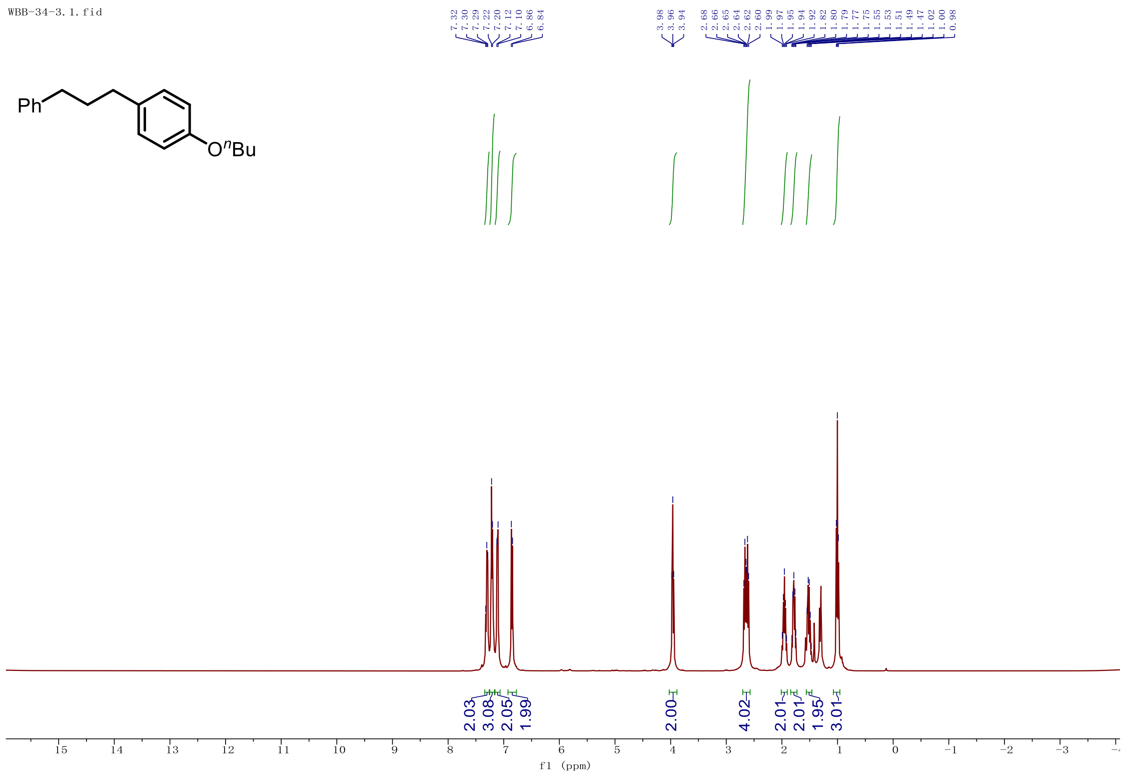

^{13}C NMR of Compound 7 (101 MHz, $CDCl_3$)

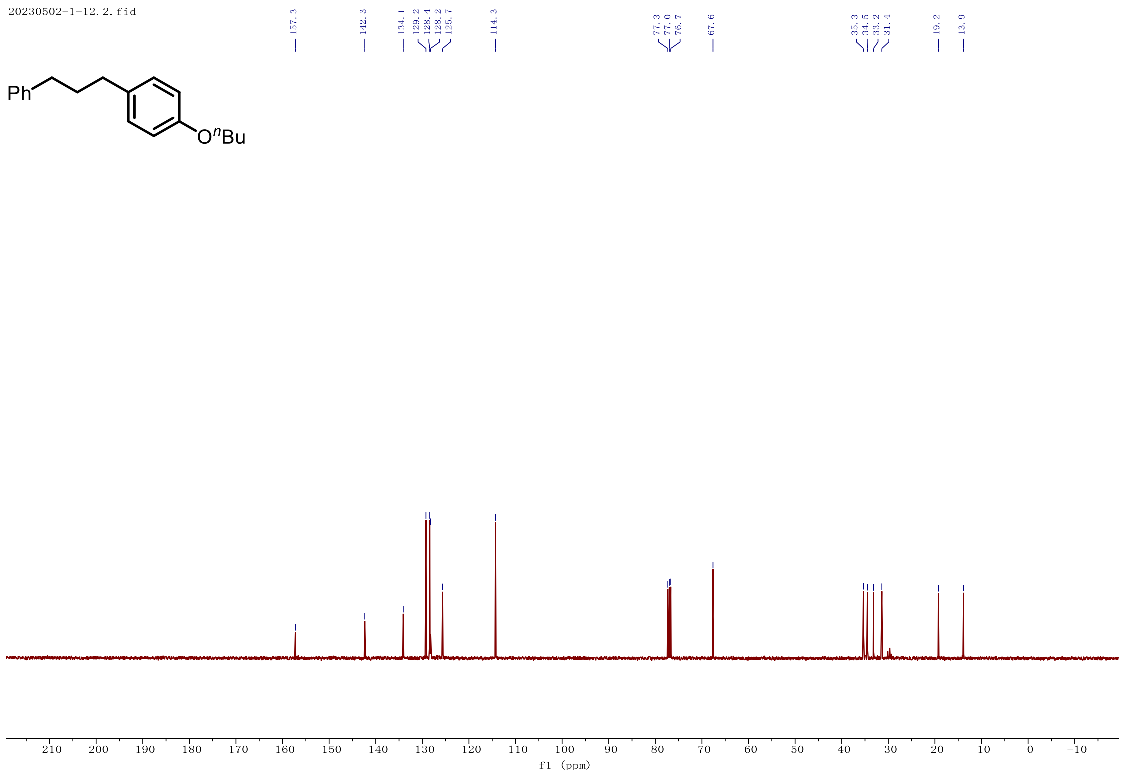

^{1}H NMR of Compound 8 (400 MHz, $CDCl_3$)

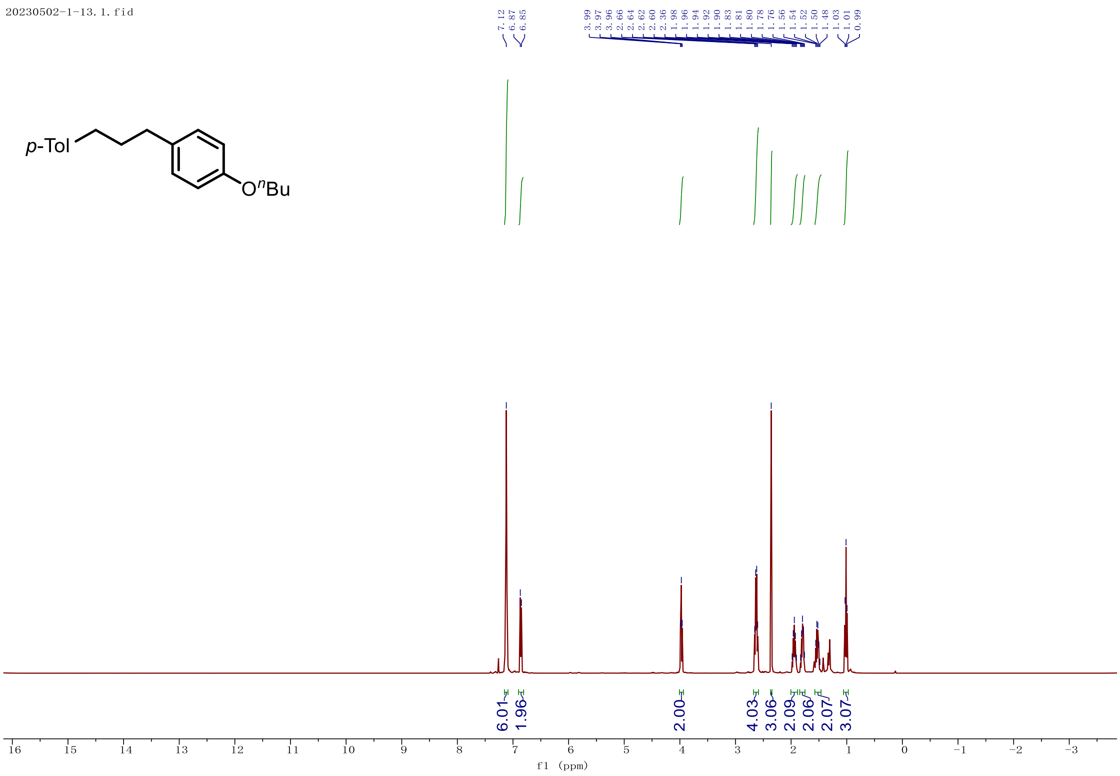

^{13}C NMR of Compound 8 (101 MHz, $CDCl_3$)

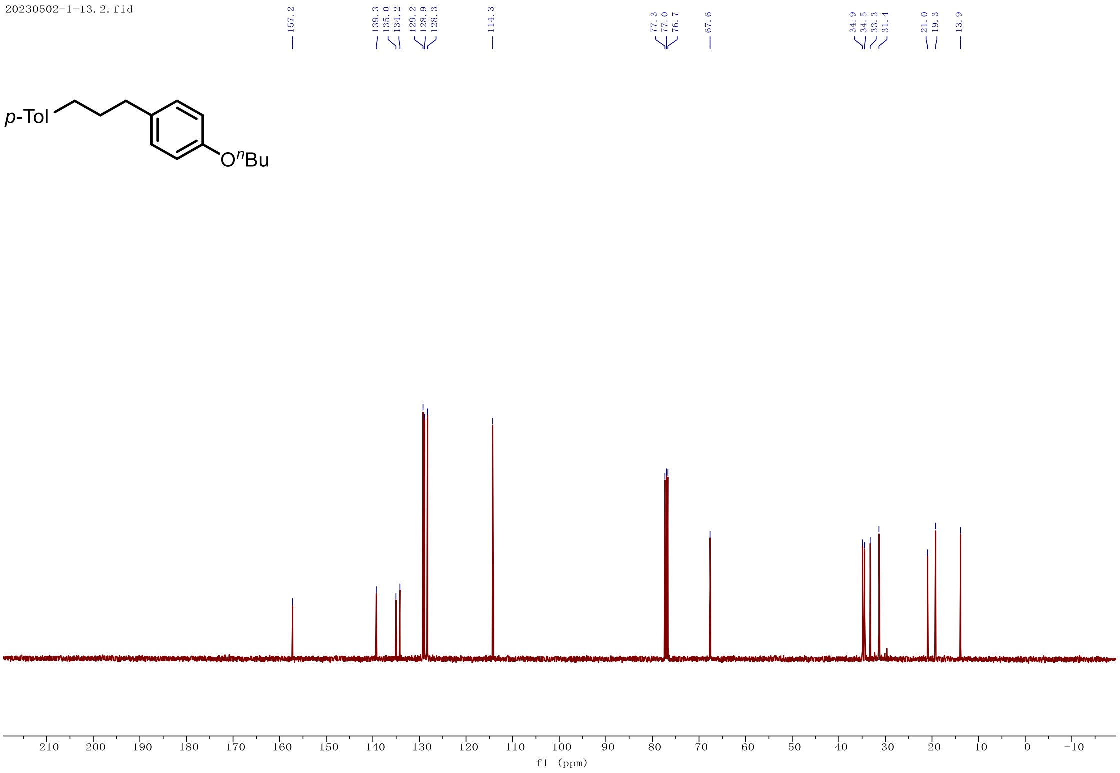

^{1}H NMR of Compound 9 (400 MHz, $CDCl_3$)

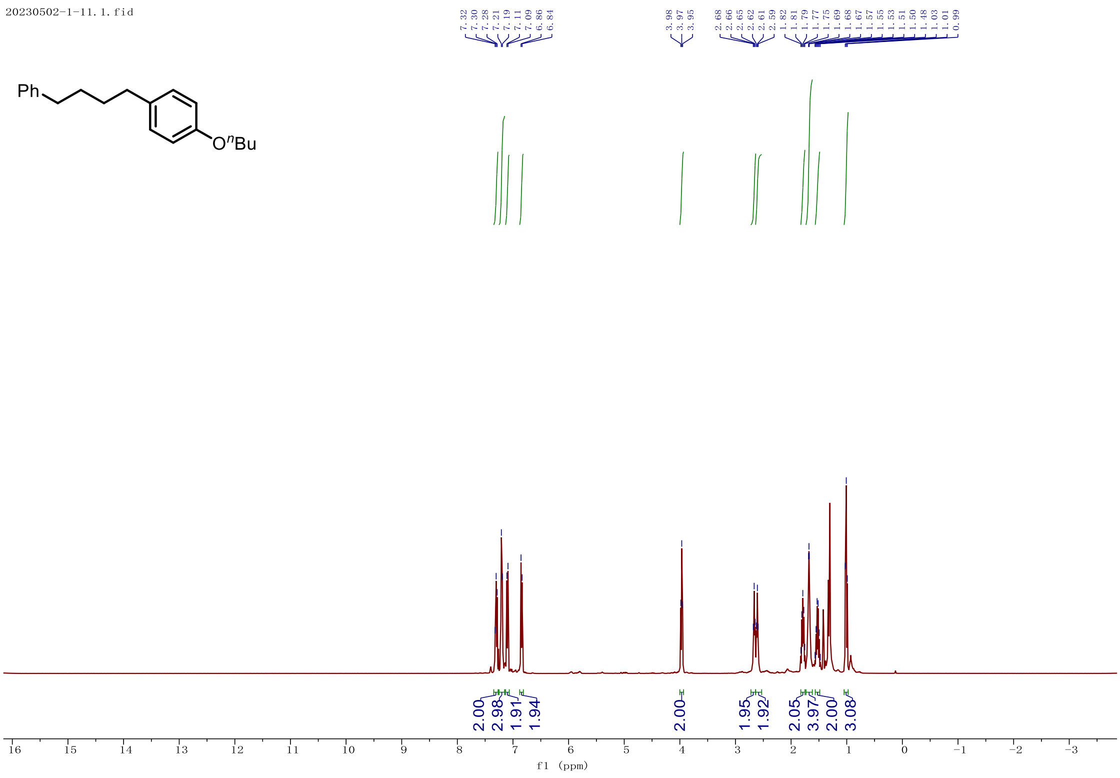

^{13}C NMR of Compound 9 (101 MHz, $CDCl_3$)

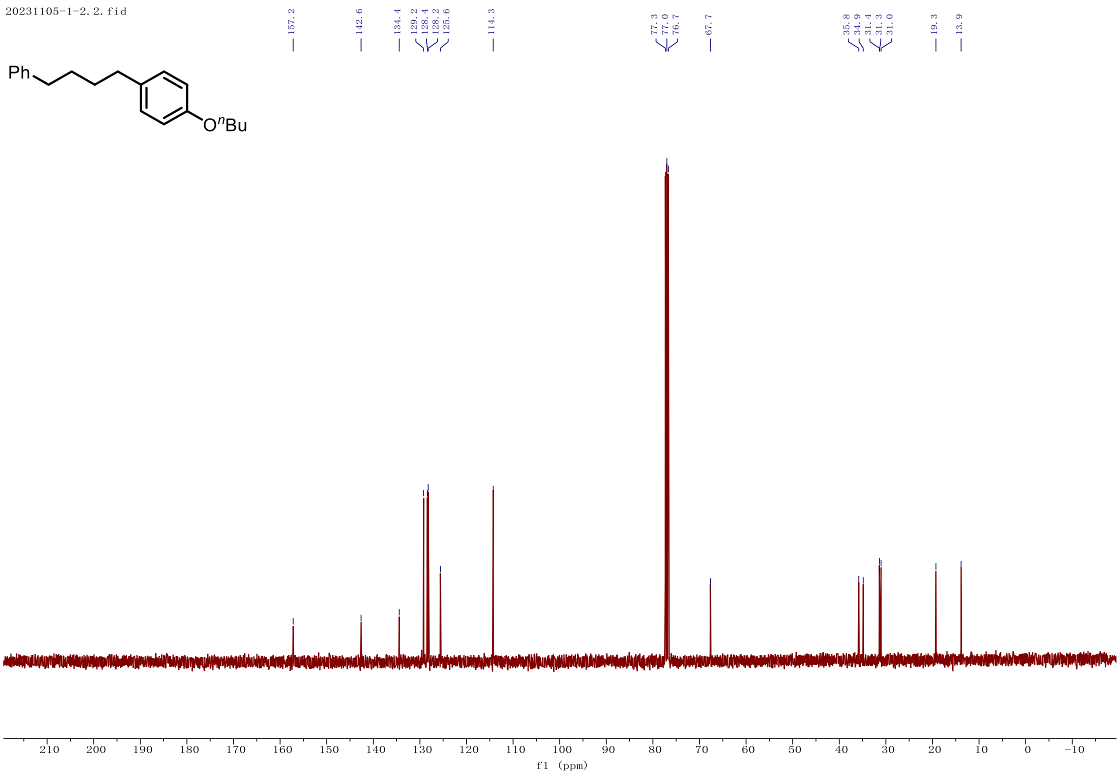

^{1}H NMR of Compound 10 (400 MHz, $CDCl_3$)

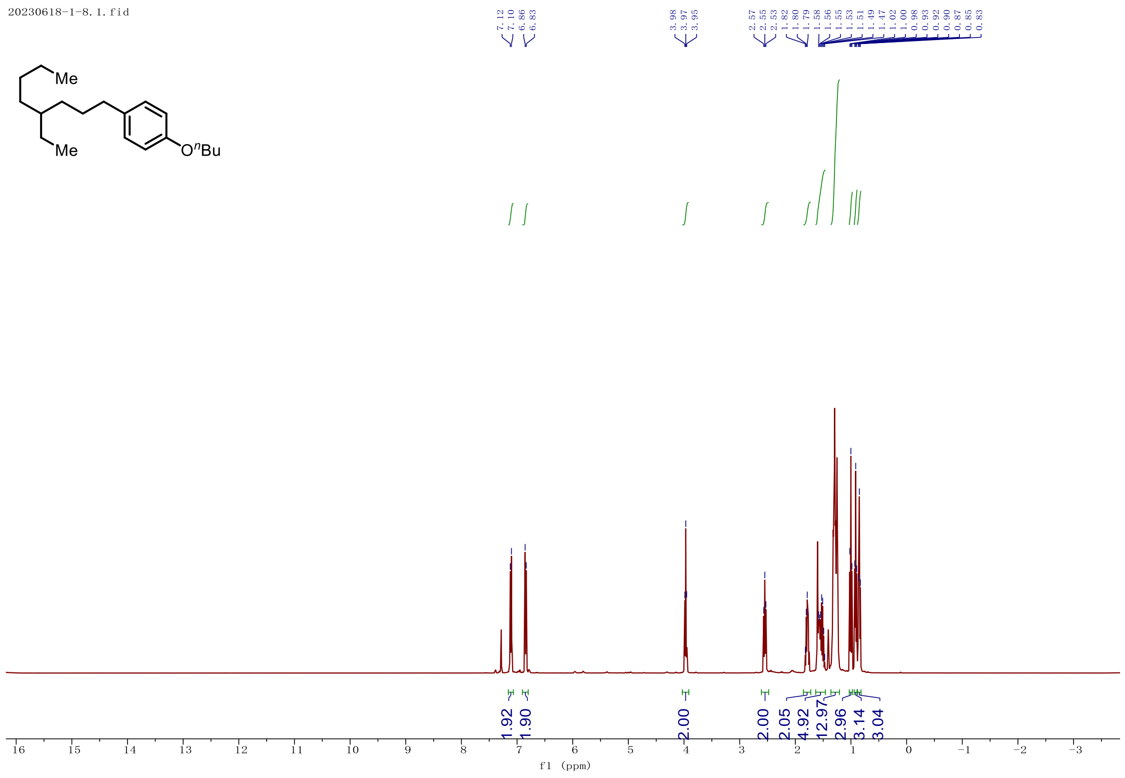

^{13}C NMR of Compound 10 (101 MHz, $CDCl_3$)

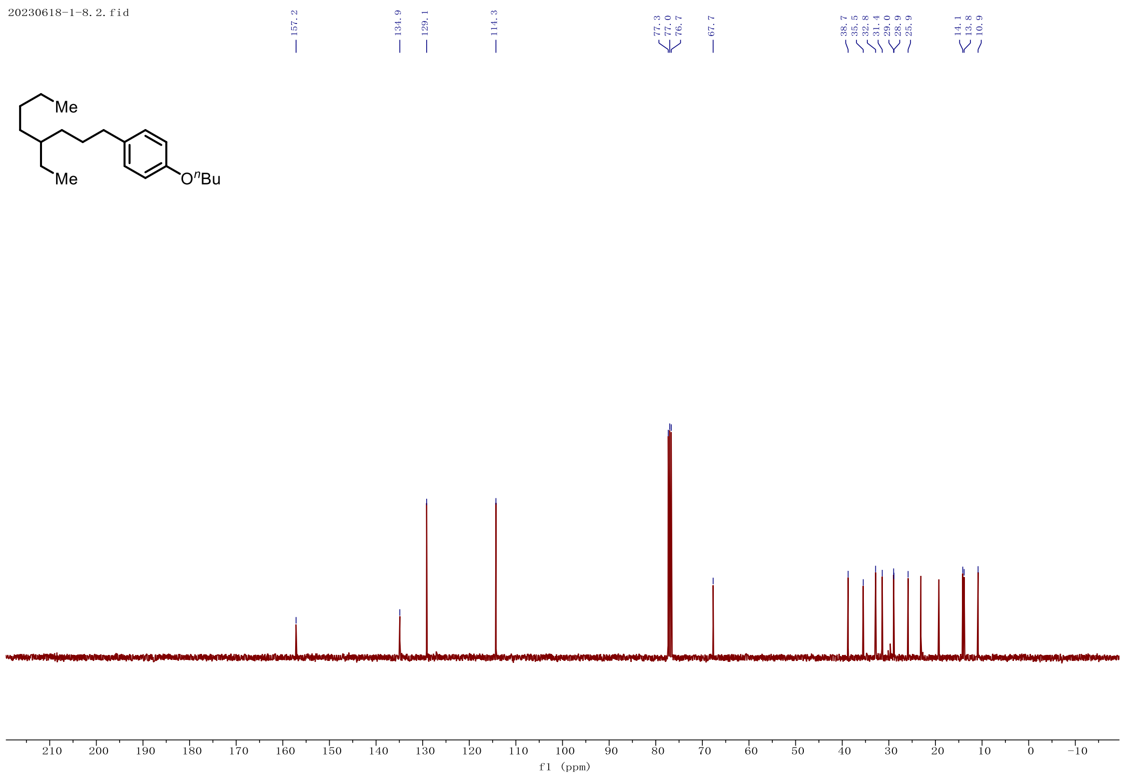

^{1}H NMR of Compound 11 (400 MHz, CDCl$_3$)

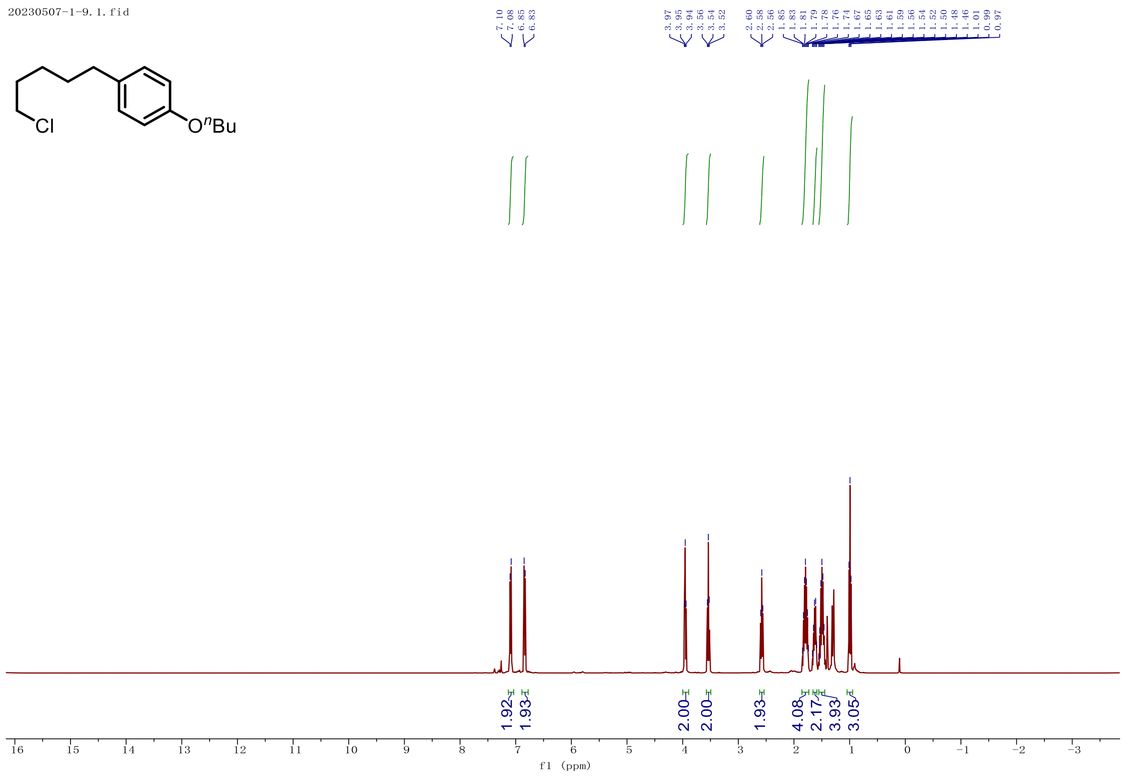

^{13}C NMR of Compound 11 (101 MHz, CDCl$_3$)

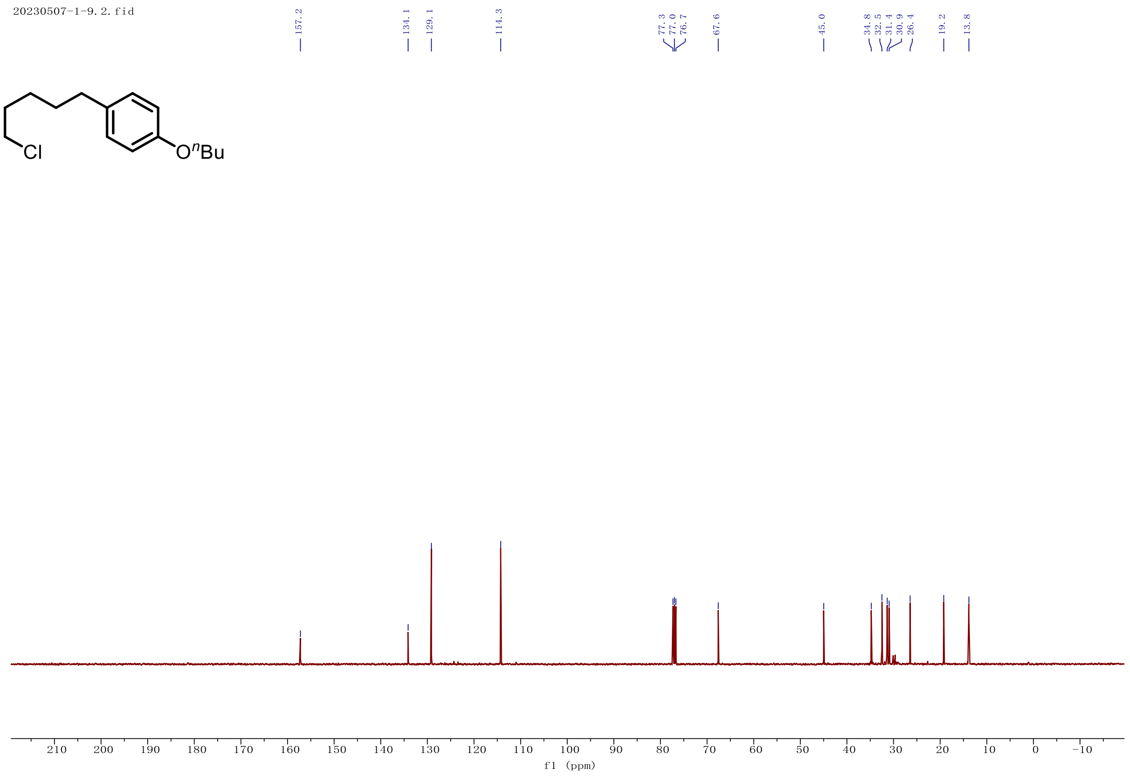

^{1}H NMR of Compound 12 (400 MHz, $CDCl_3$)

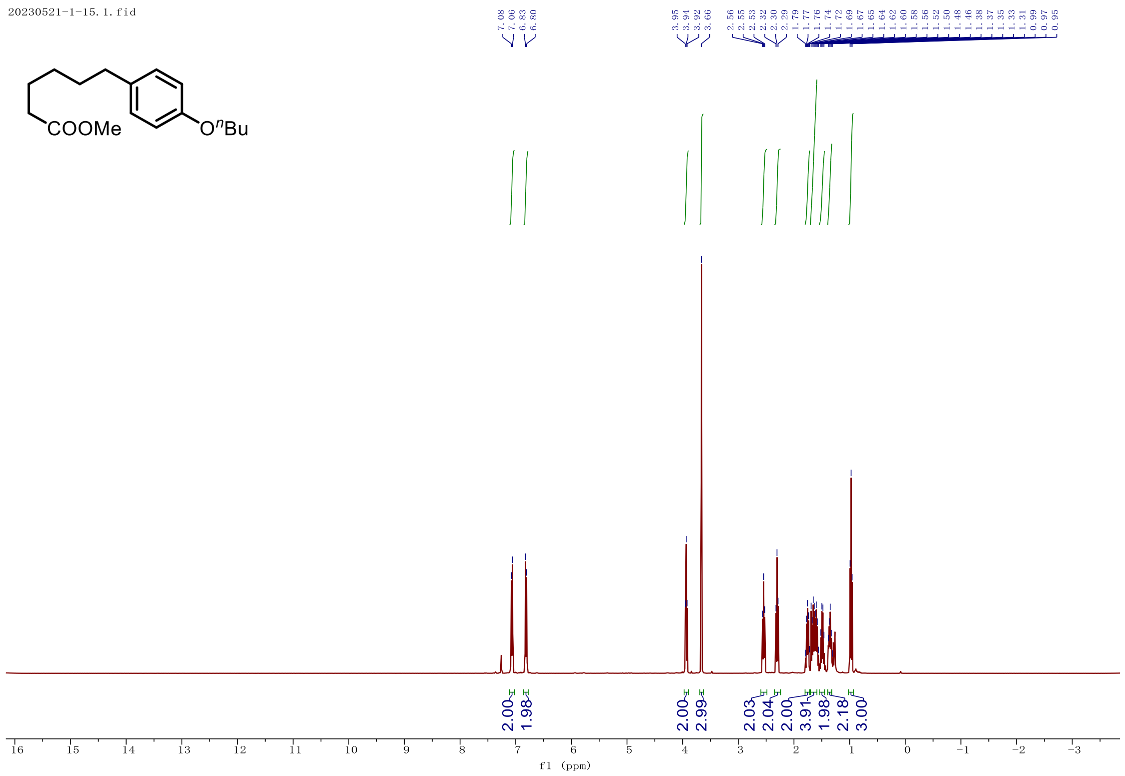

^{13}C NMR of Compound 12 (101 MHz, $CDCl_3$)

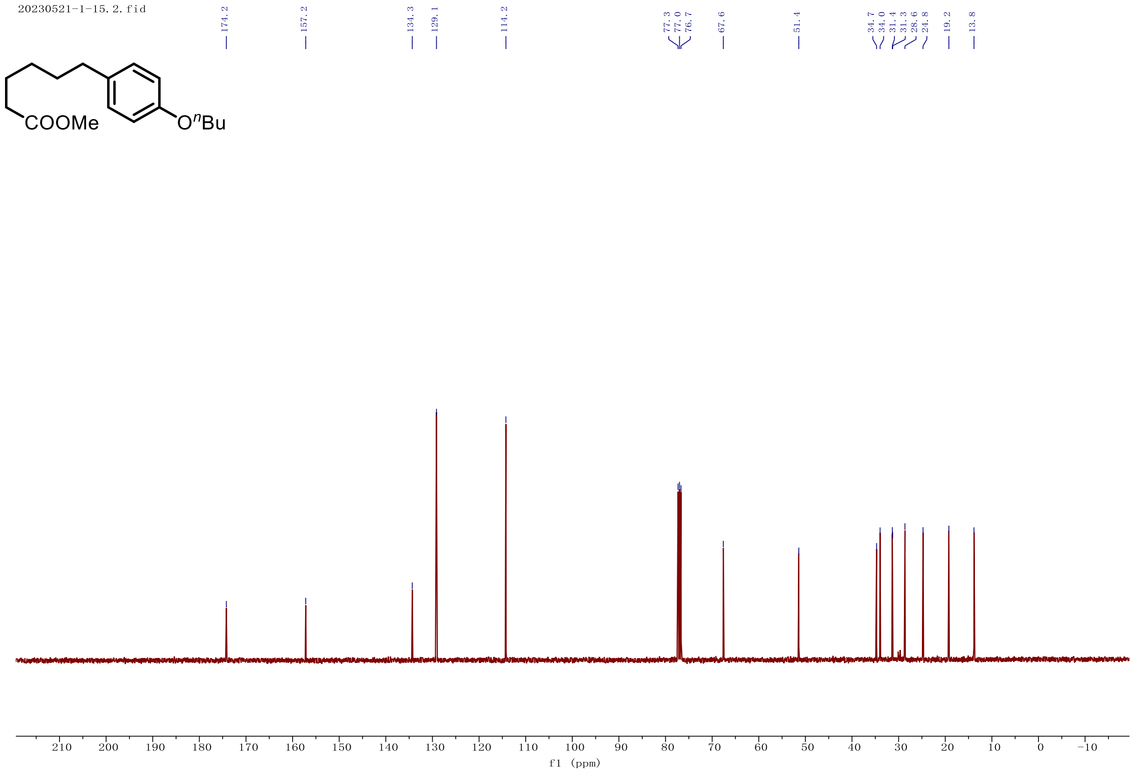

^{1}H NMR of Compound 13 (400 MHz, $CDCl_3$)

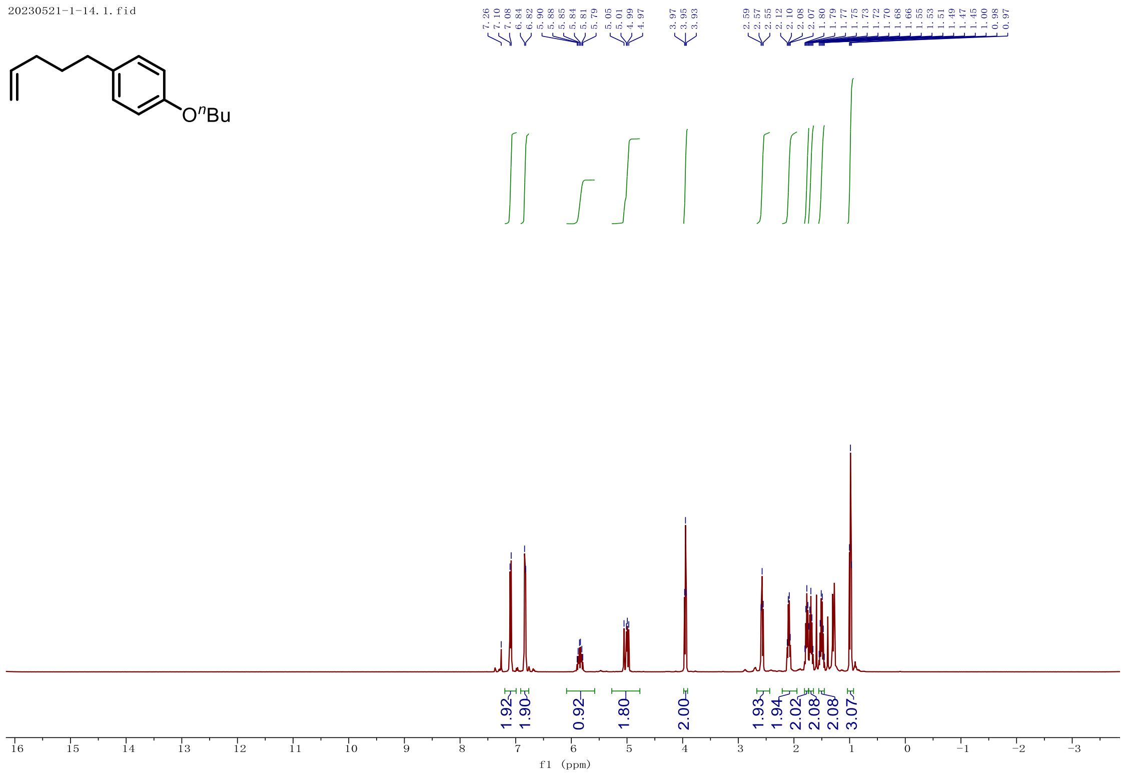

^{13}C NMR of Compound 13 (101 MHz, $CDCl_3$)

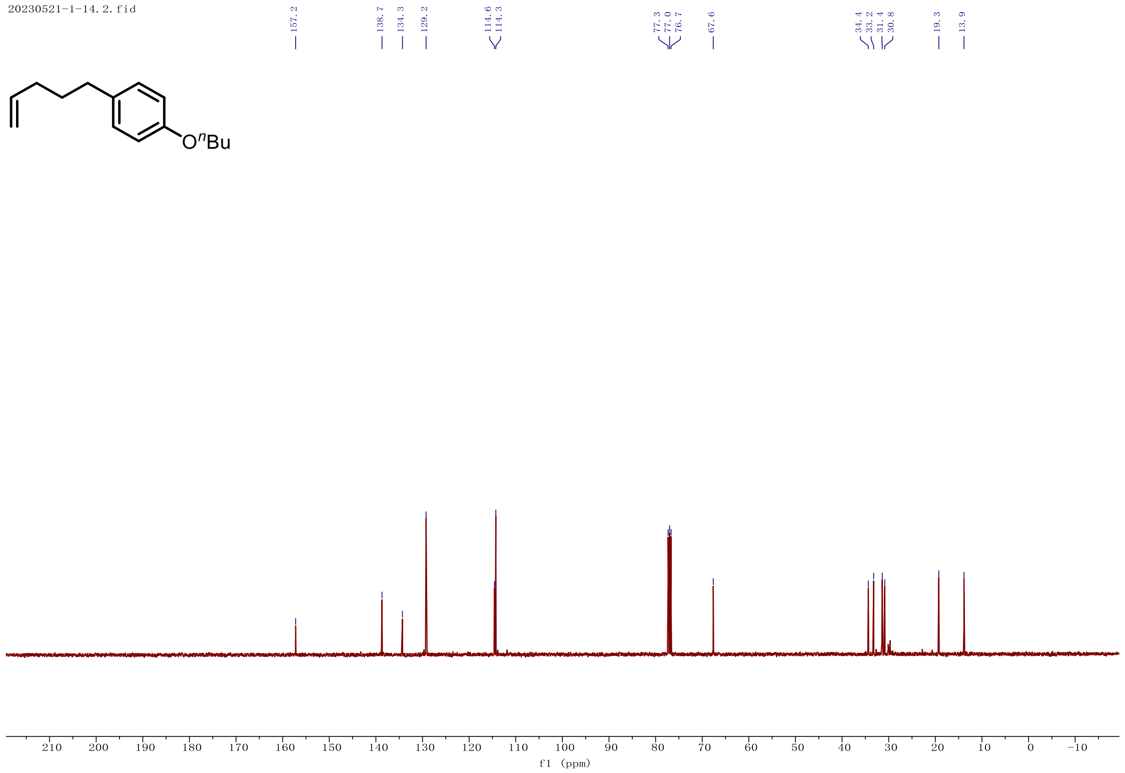

^{1}H NMR of Compound 14 (400 MHz, $CDCl_3$)

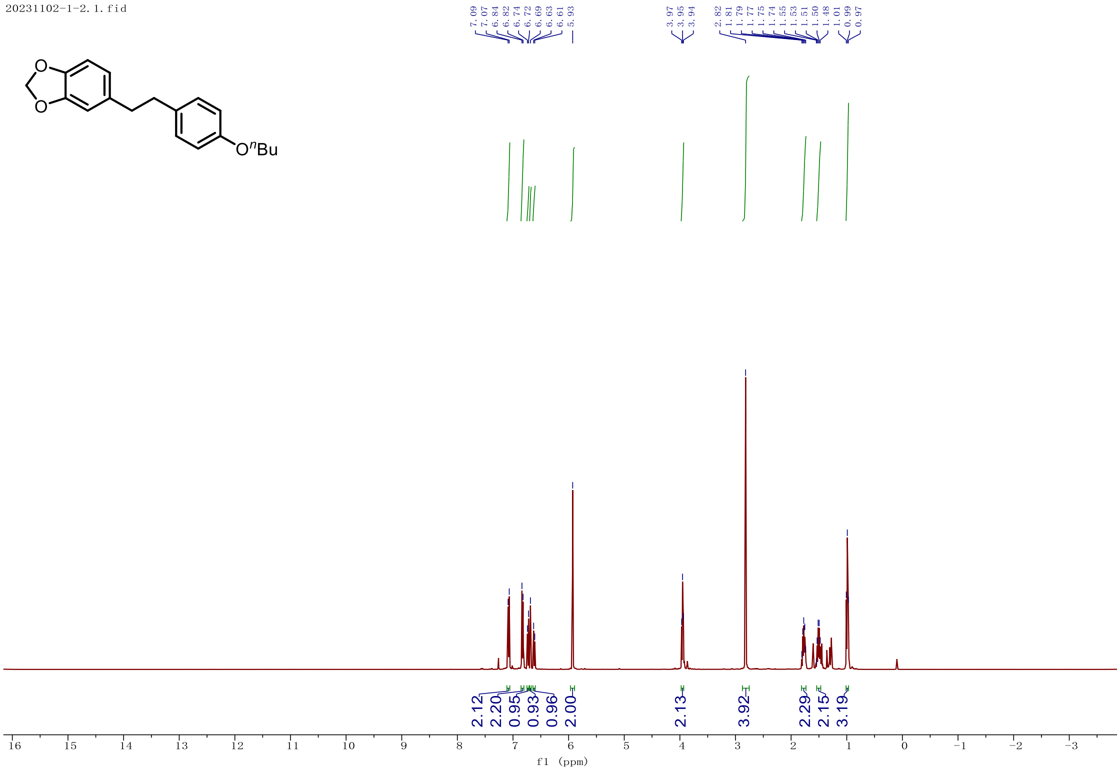

^{13}C NMR of Compound 14 (101 MHz, $CDCl_3$)

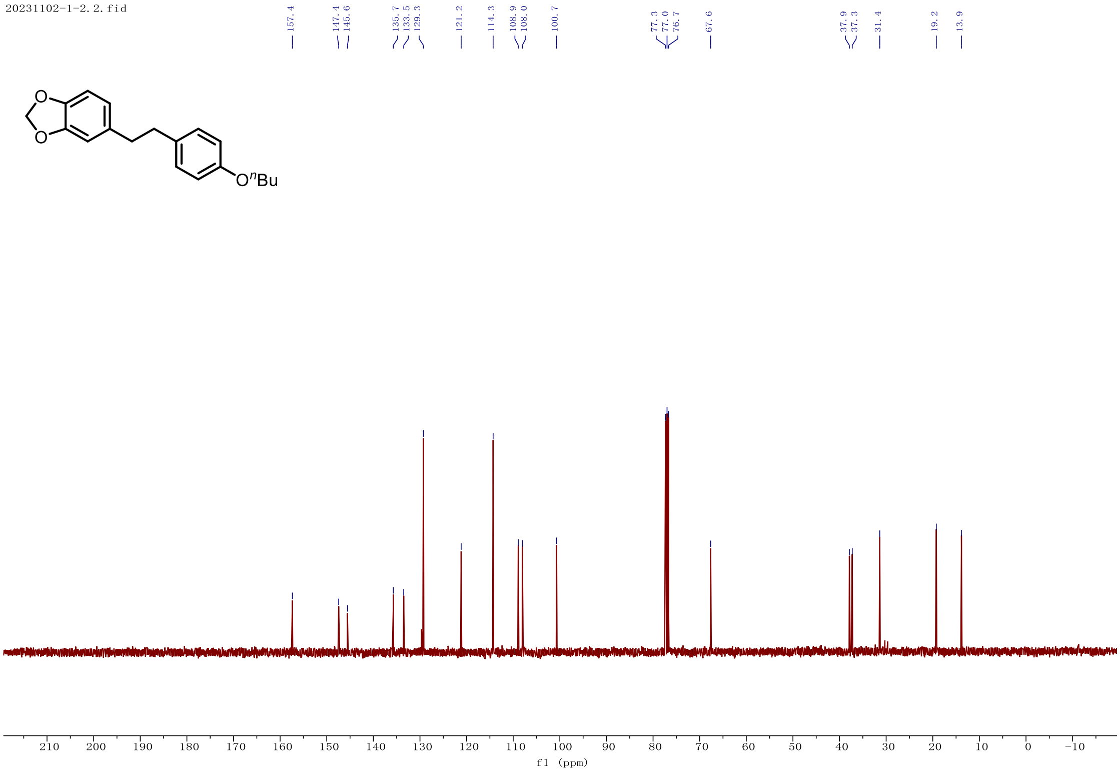

^{1}H NMR of Compound 15 (400 MHz, $CDCl_3$)

20231017-NO.2-10.1.fid

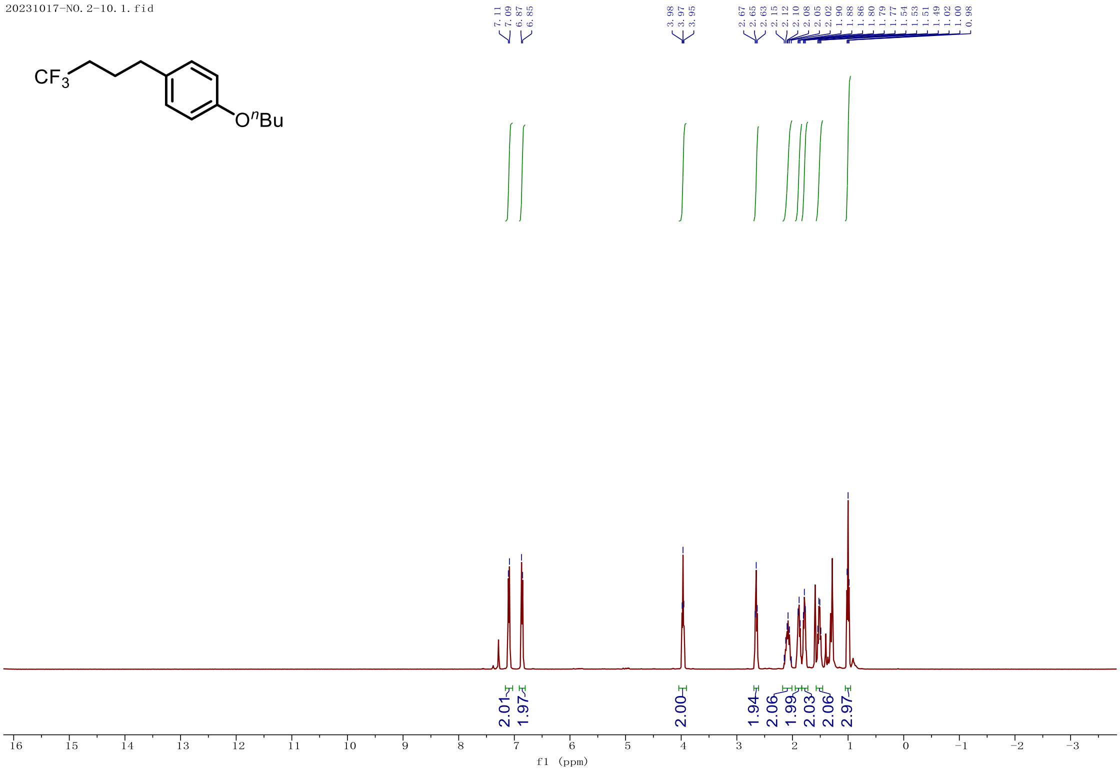

^{13}C NMR of Compound 15 (101 MHz, $CDCl_3$)

20231117-HXS-WBB-153-1.2.fid

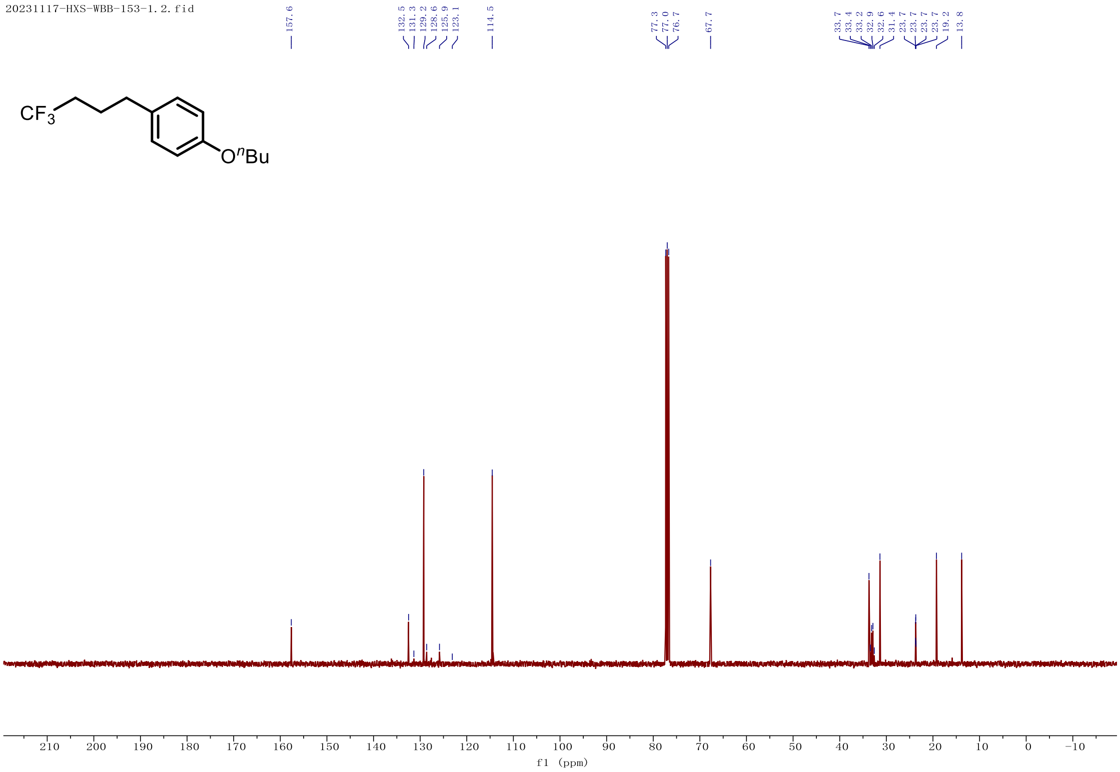

^{19}F NMR of Compound 15 (376 MHz, $CDCl_3$)

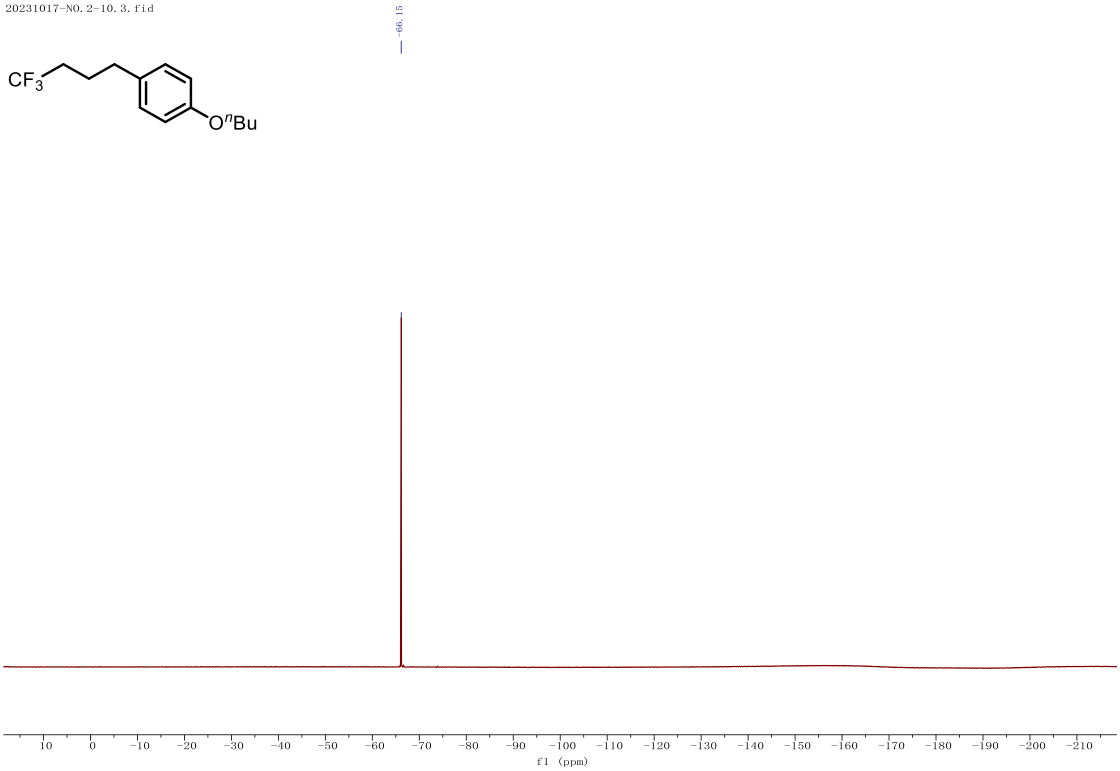

^{1}H NMR of Compound 16 (400 MHz, $CDCl_3$)

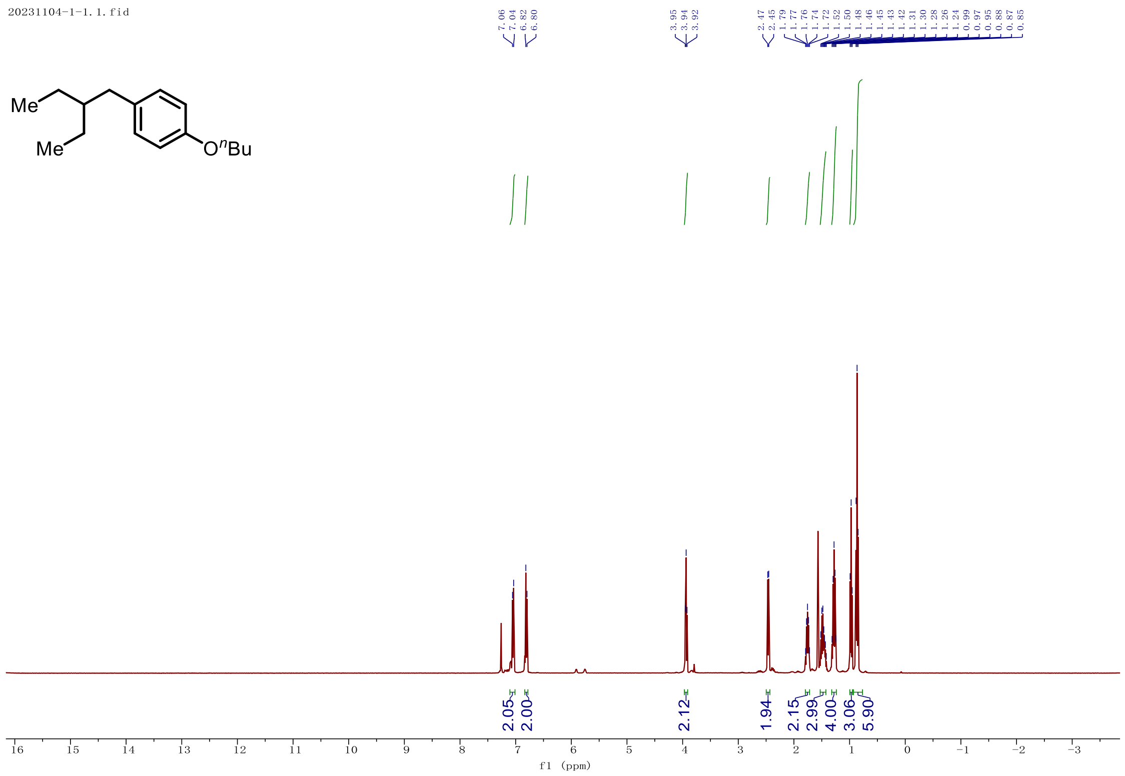

^{13}C NMR of Compound 16 (101 MHz, $CDCl_3$)

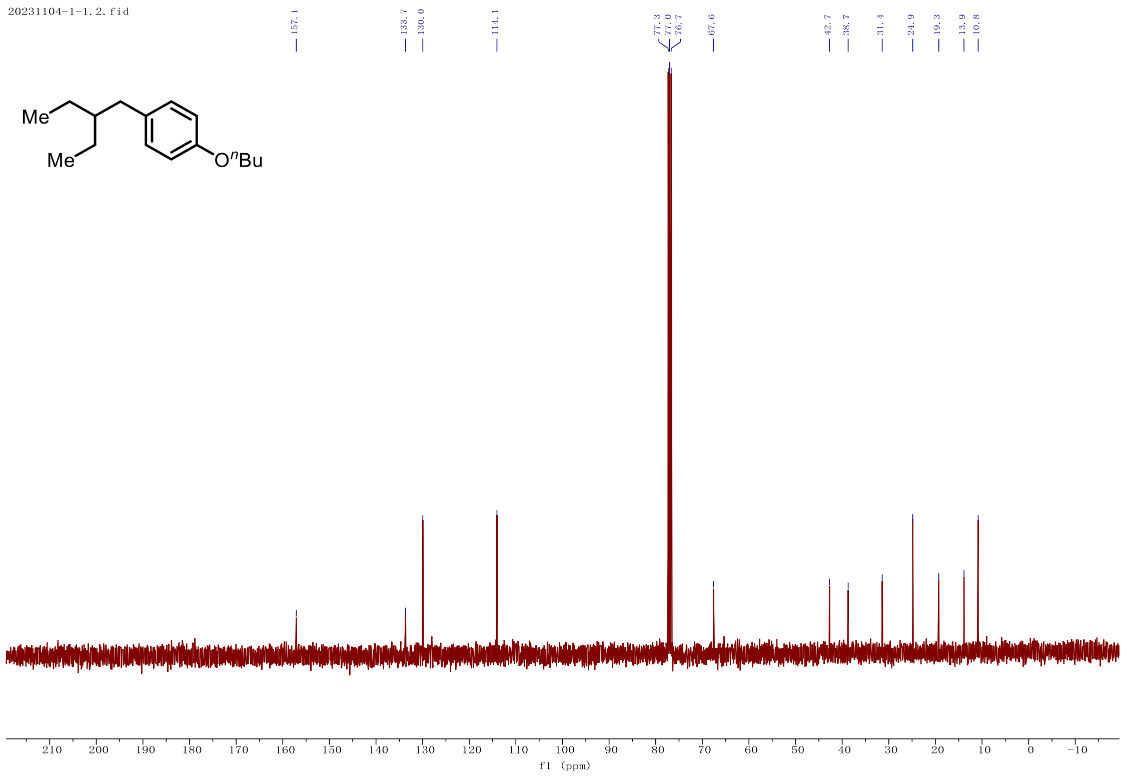

^{1}H NMR of Compound 17 (400 MHz, $CDCl_3$)

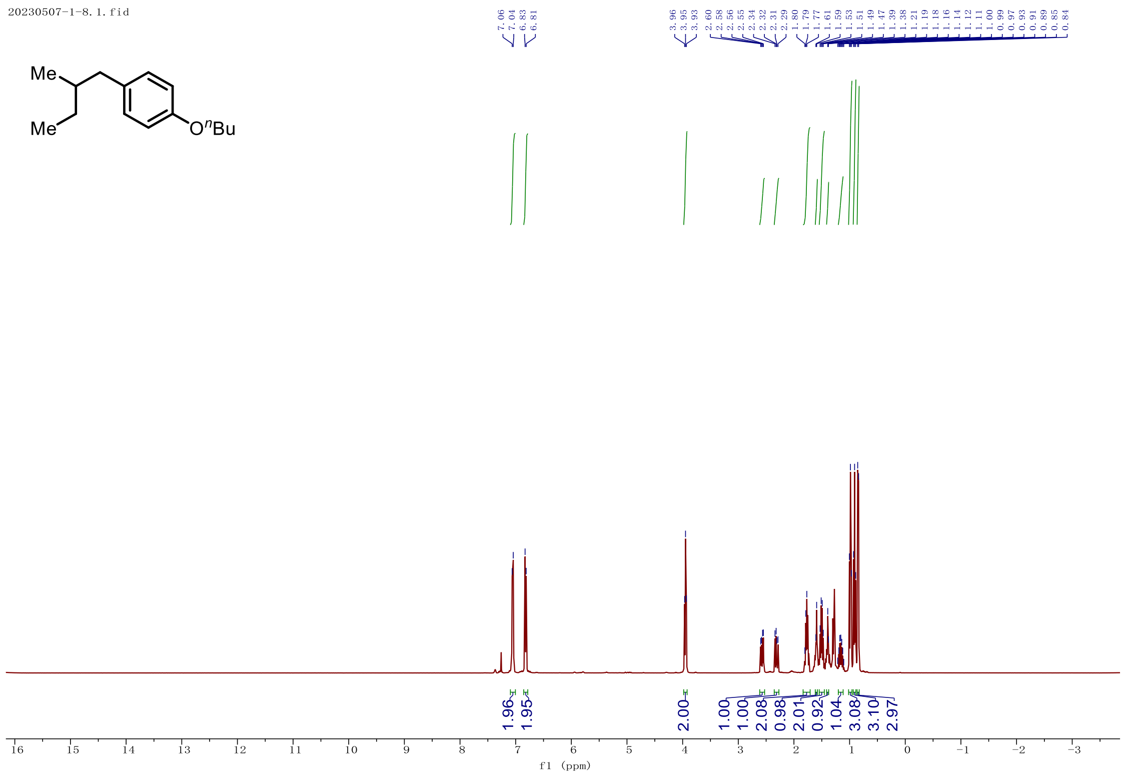

^{13}C NMR of Compound 17 (101 MHz, $CDCl_3$)

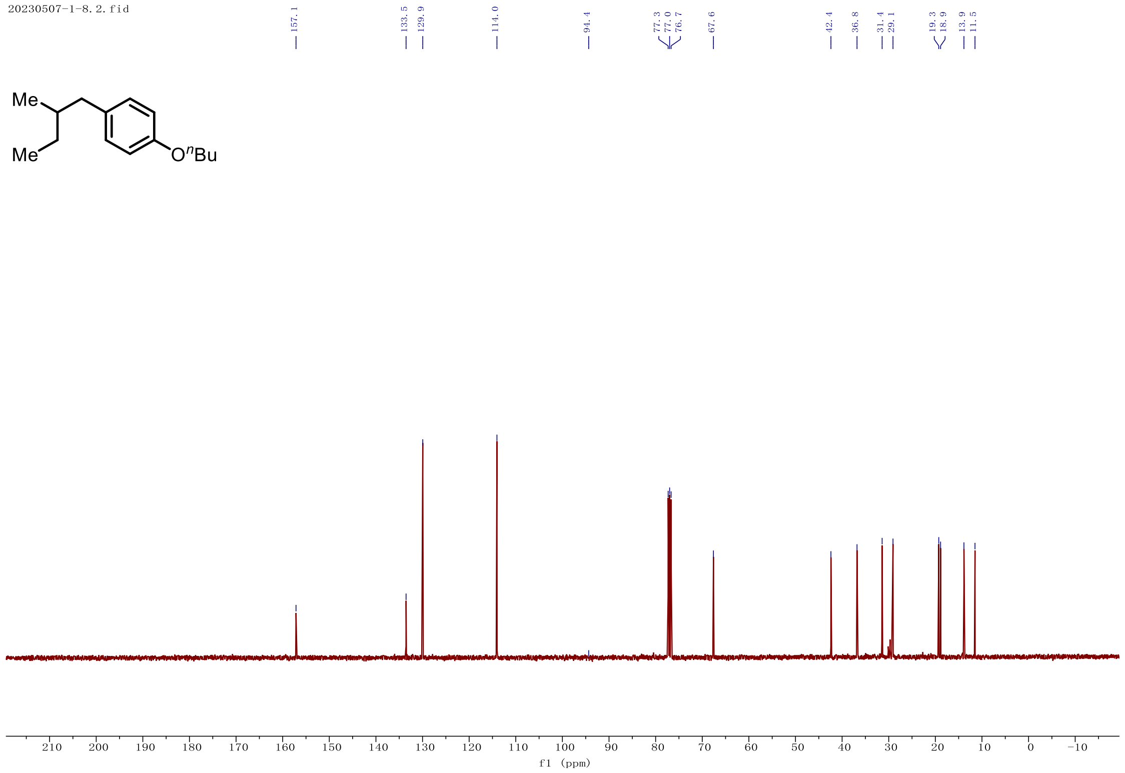

^{1}H NMR of Compound 18 (400 MHz, $CDCl_3$)

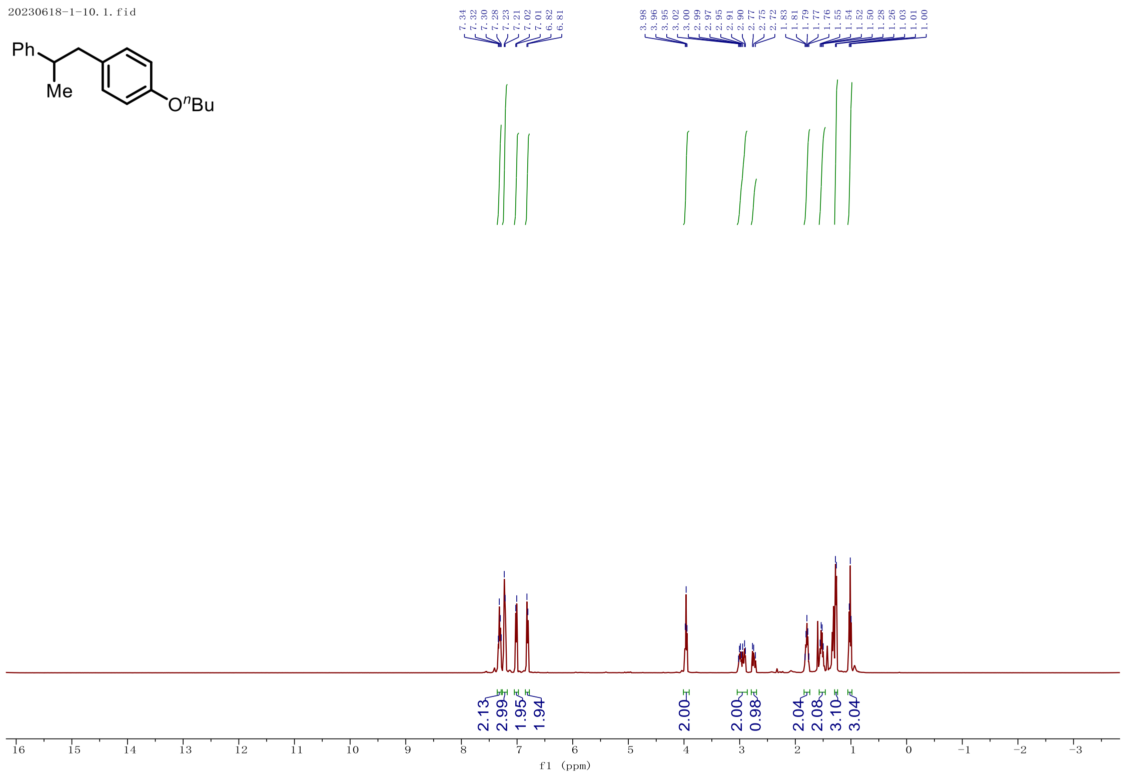

^{13}C NMR of Compound 18 (101 MHz, $CDCl_3$)

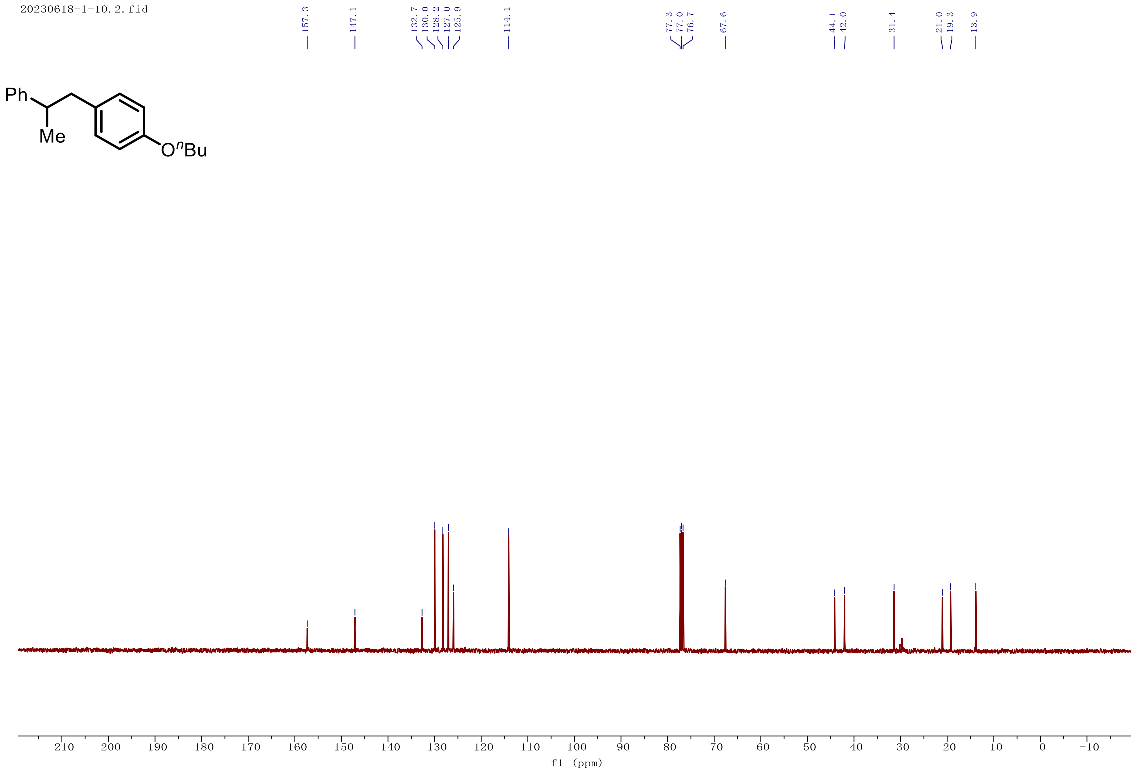

^{1}H NMR of Compound 19 (400 MHz, $CDCl_3$)

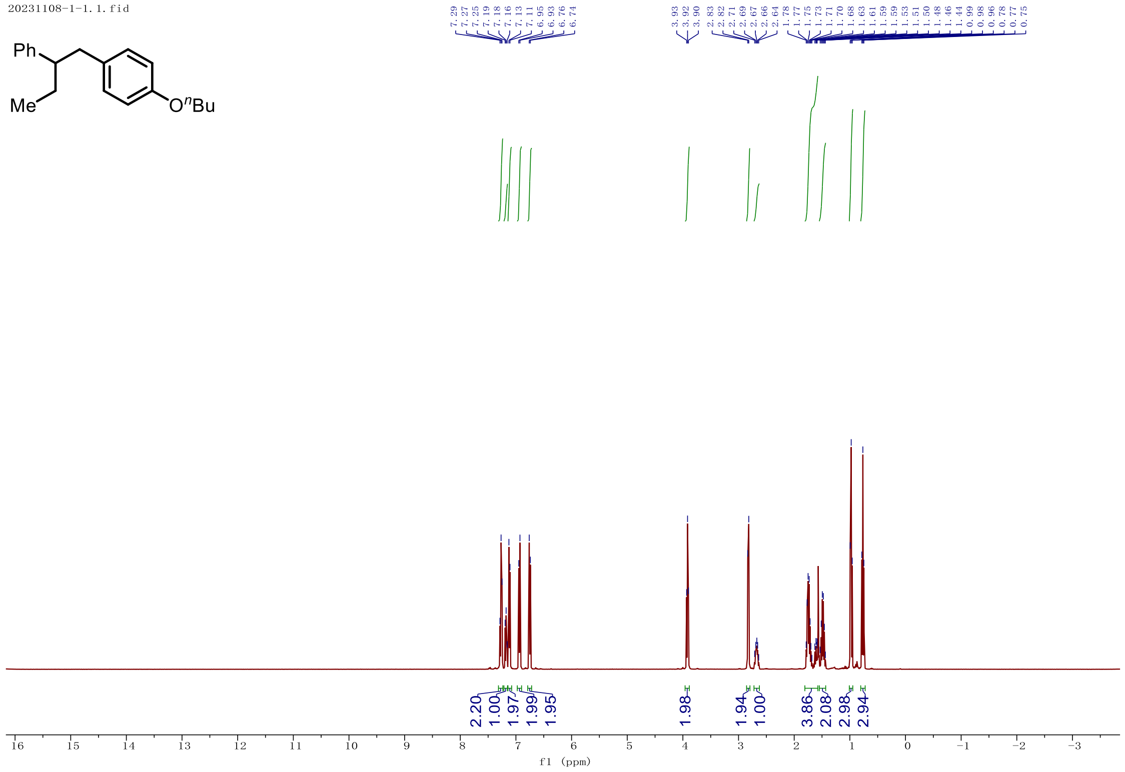

^{13}C NMR of Compound 19 (101 MHz, $CDCl_3$)

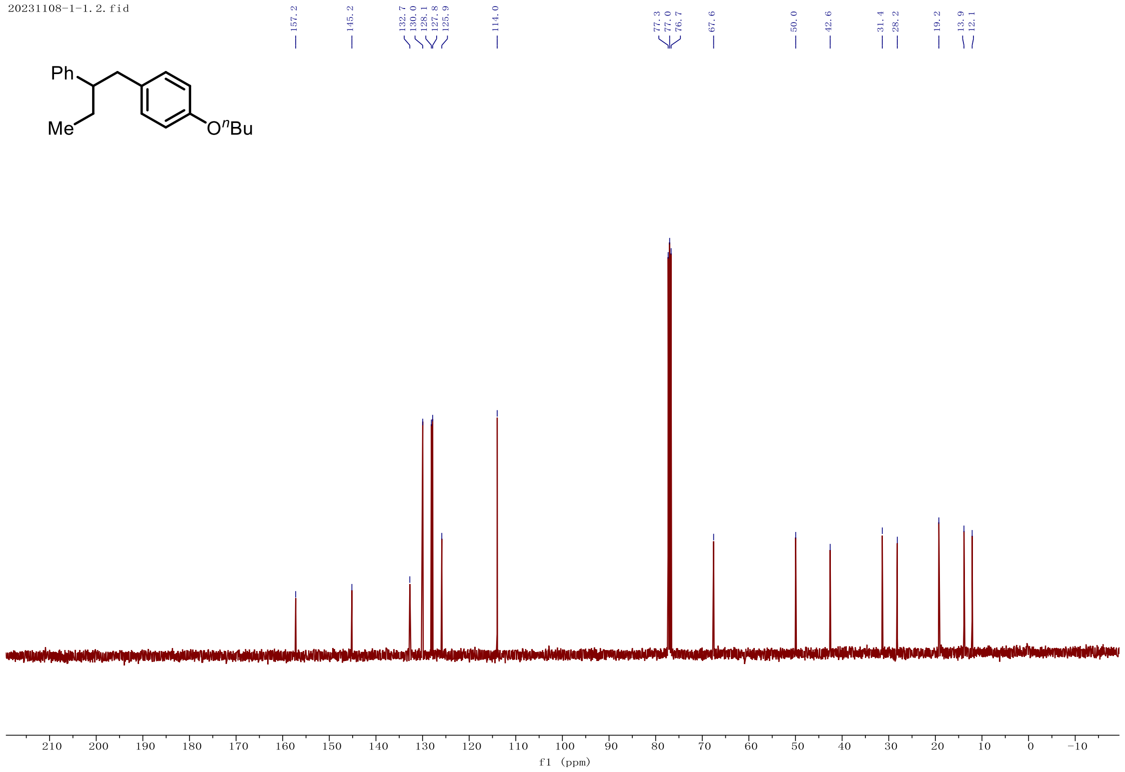

^{1}H NMR of Compound 20 (400 MHz, $CDCl_3$)

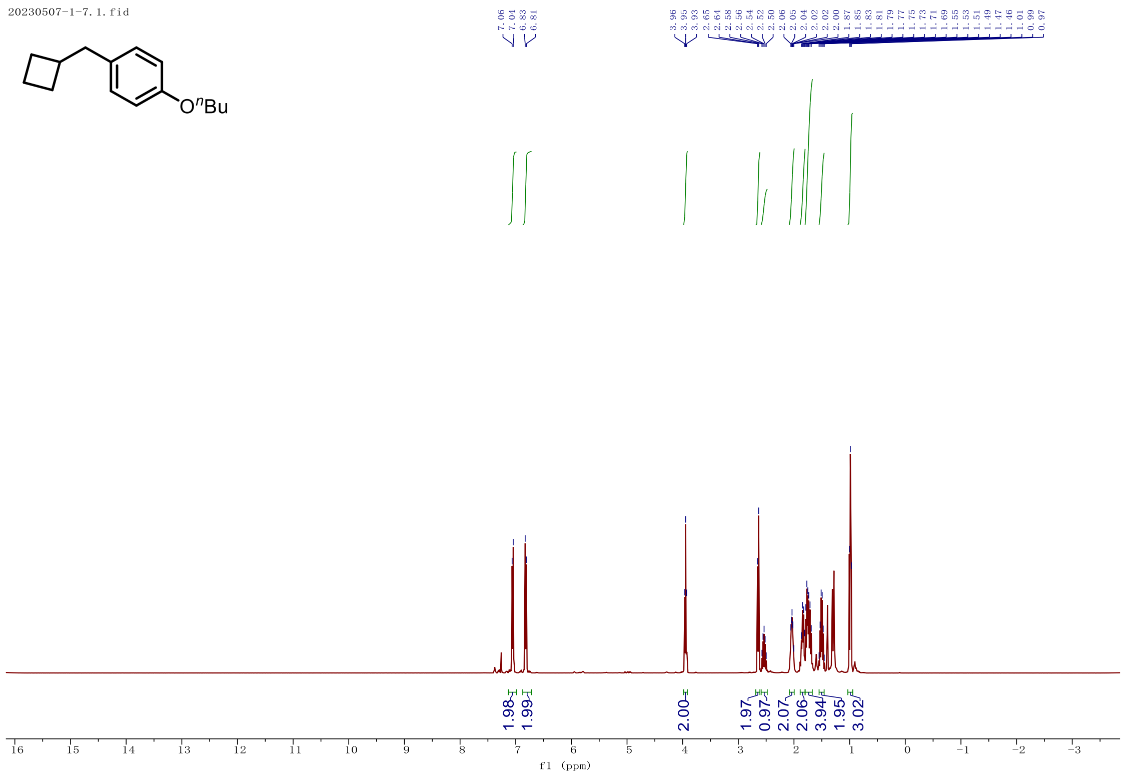

^{13}C NMR of Compound 20 (101 MHz, $CDCl_3$)

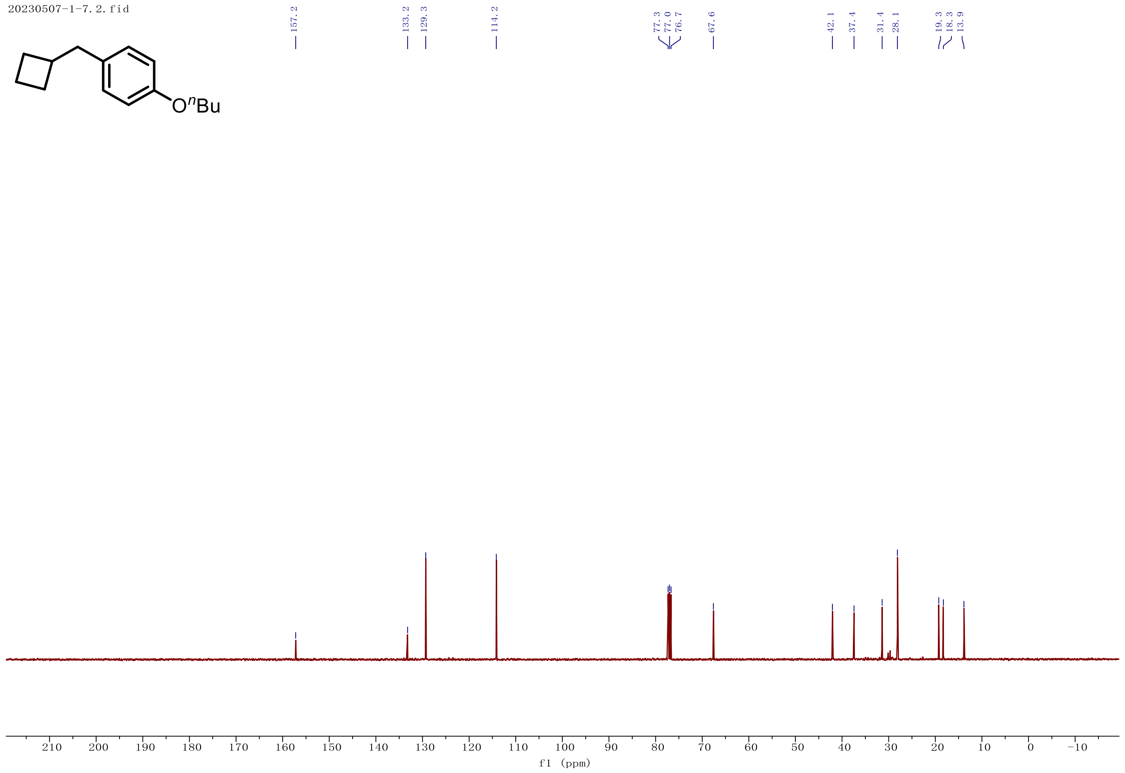

^{1}H NMR of Compound 21 (400 MHz, $CDCl_3$)

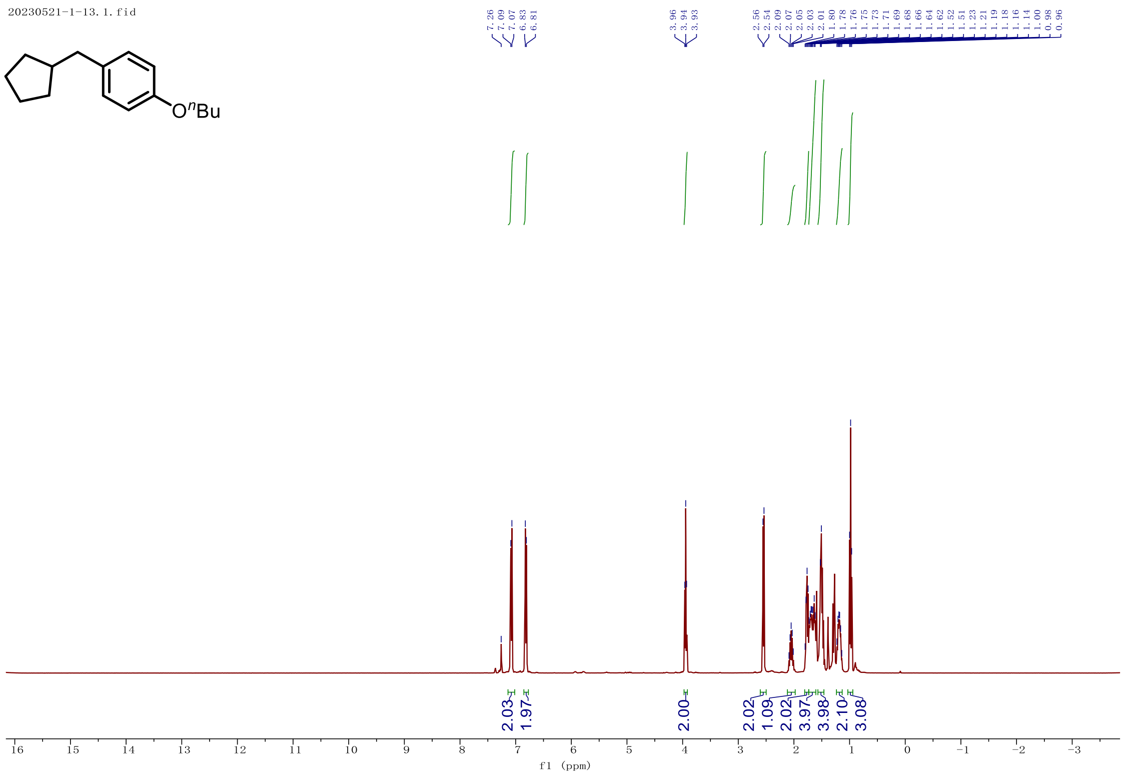

^{13}C NMR of Compound 21 (101 MHz, $CDCl_3$)

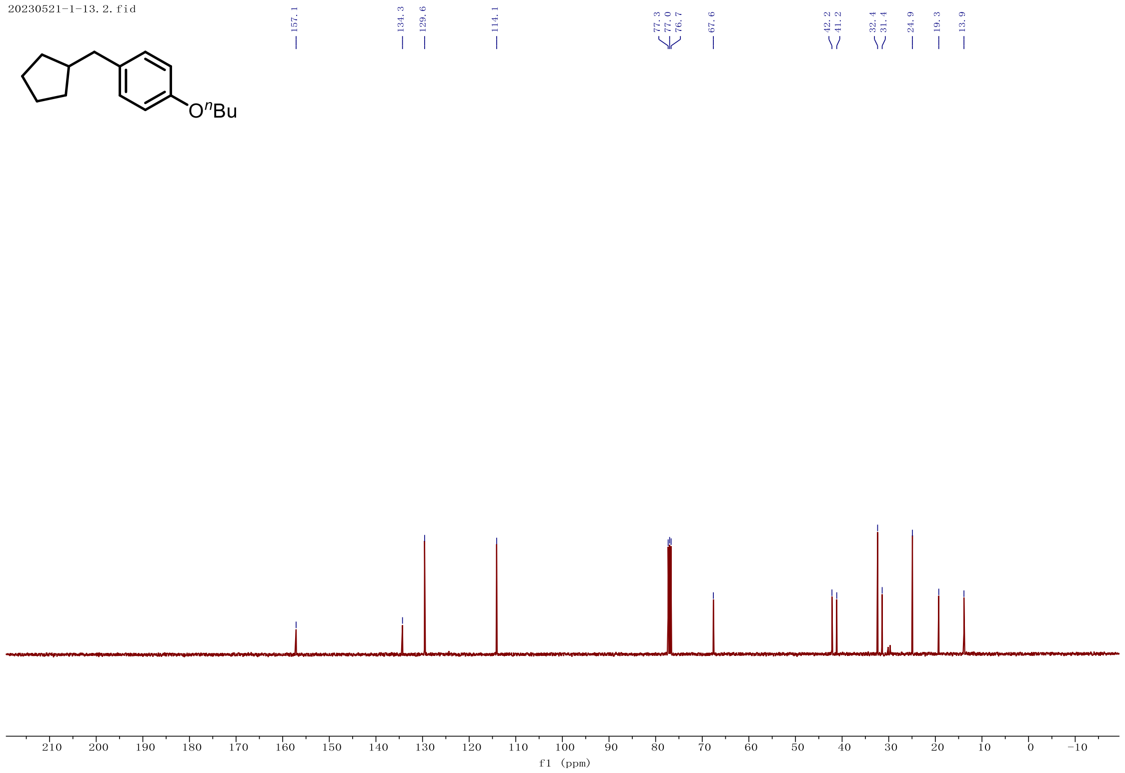

^{1}H NMR of Compound 22 (400 MHz, $CDCl_3$)

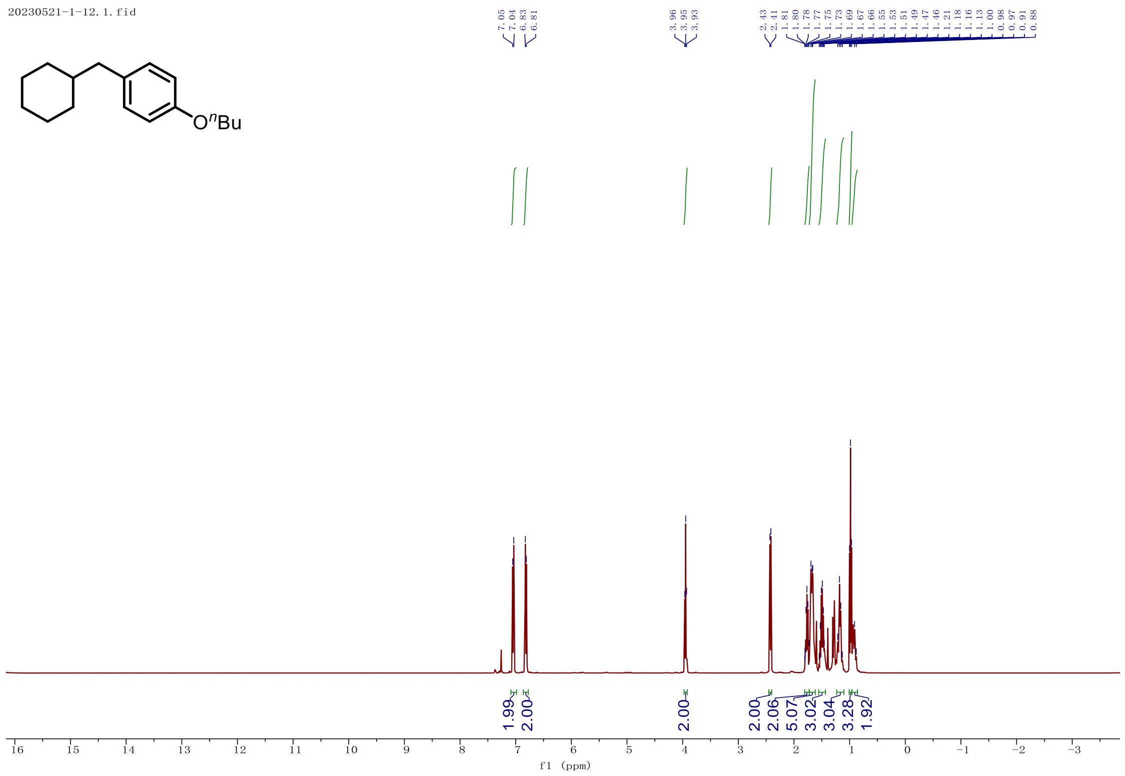

^{13}C NMR of Compound 22 (101 MHz, $CDCl_3$)

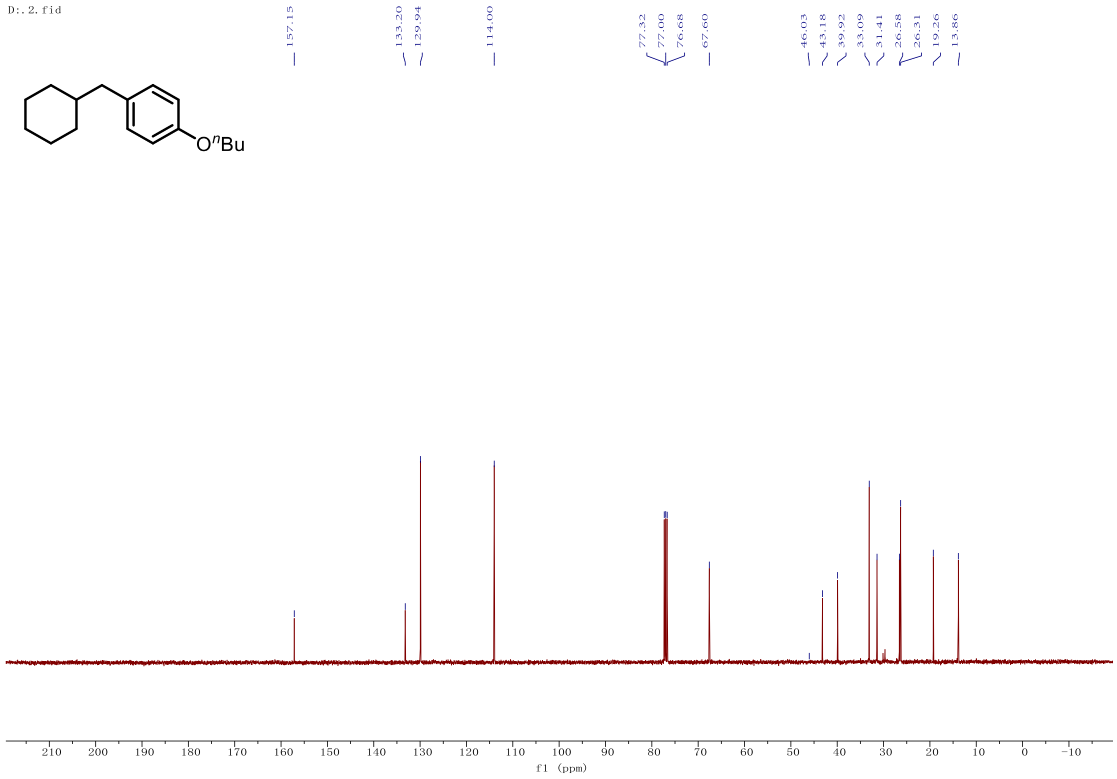

^{1}H NMR of Compound 23 (400 MHz, $CDCl_3$)

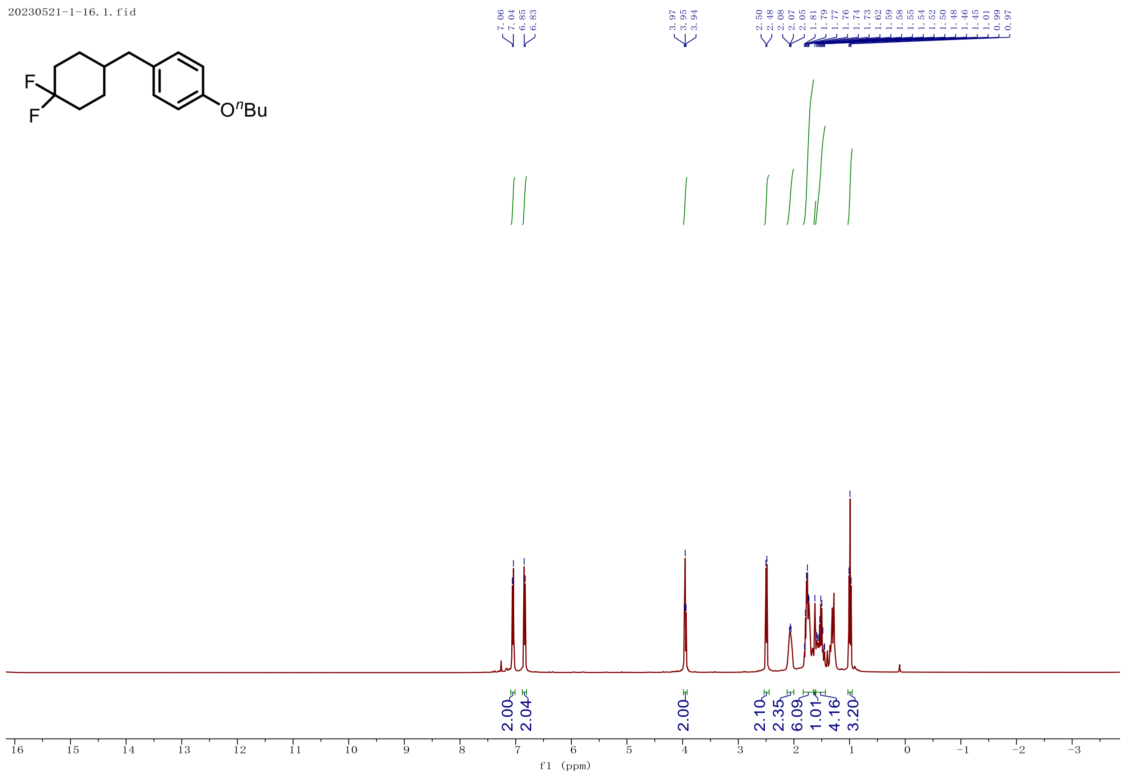

^{13}C NMR of Compound 23 (101 MHz, $CDCl_3$)

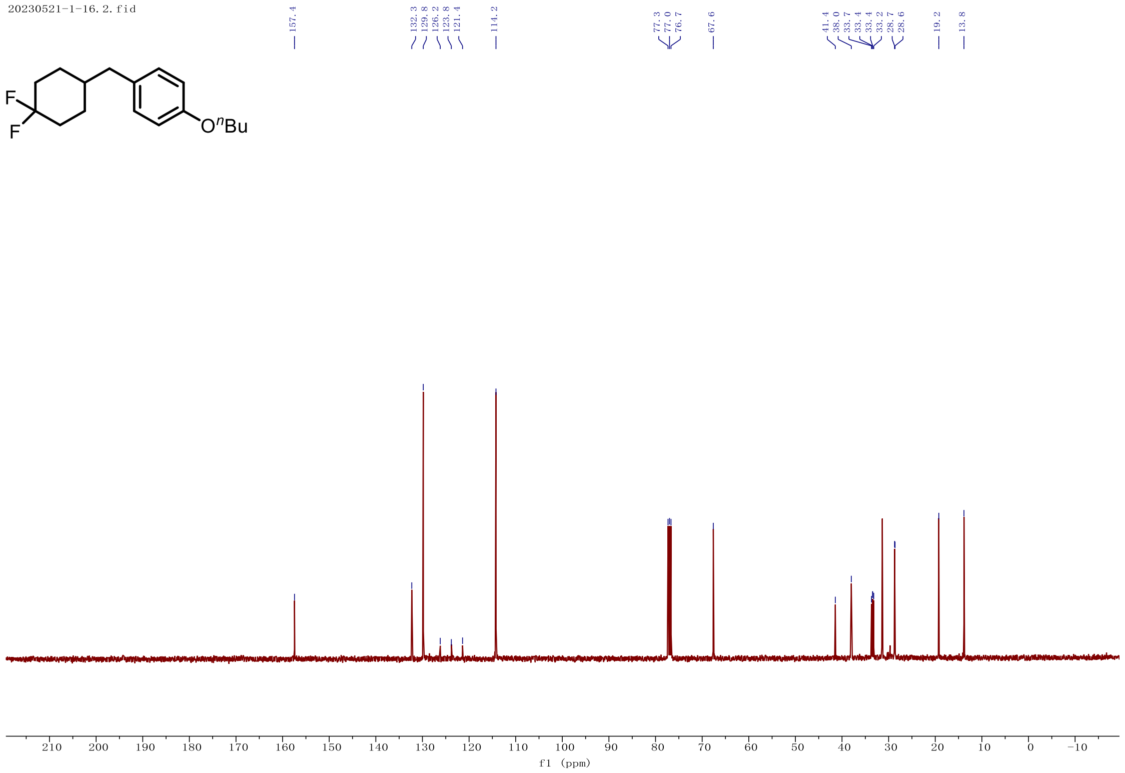

^{19}F NMR of Compound 23 (376 MHz, $CDCl_3$)

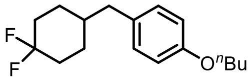

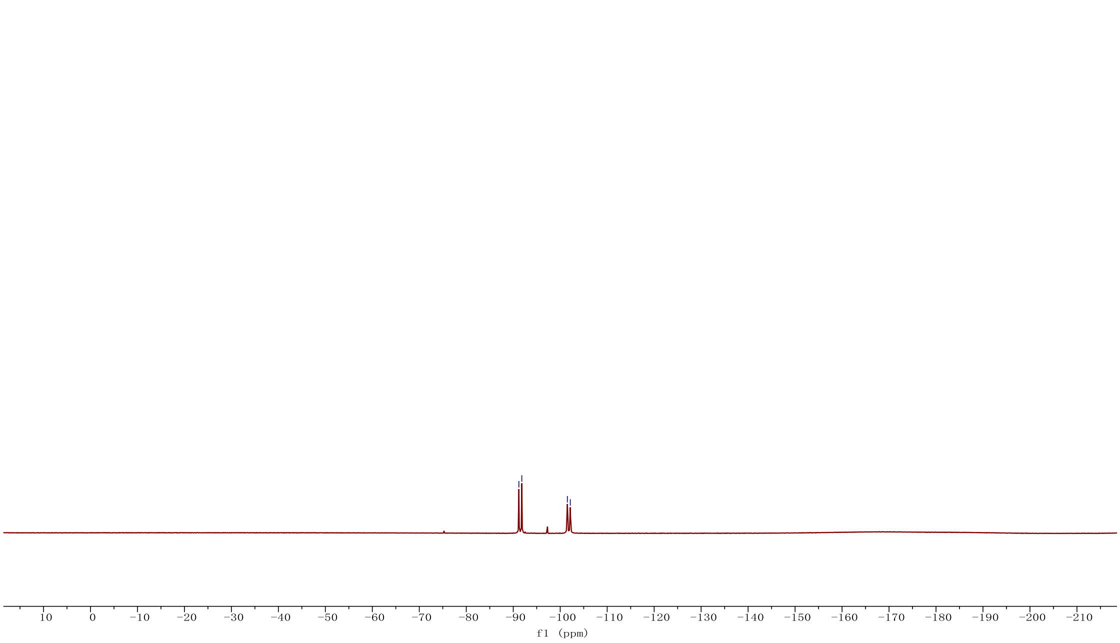

^{1}H NMR of Compound 24 (400 MHz, $CDCl_3$)

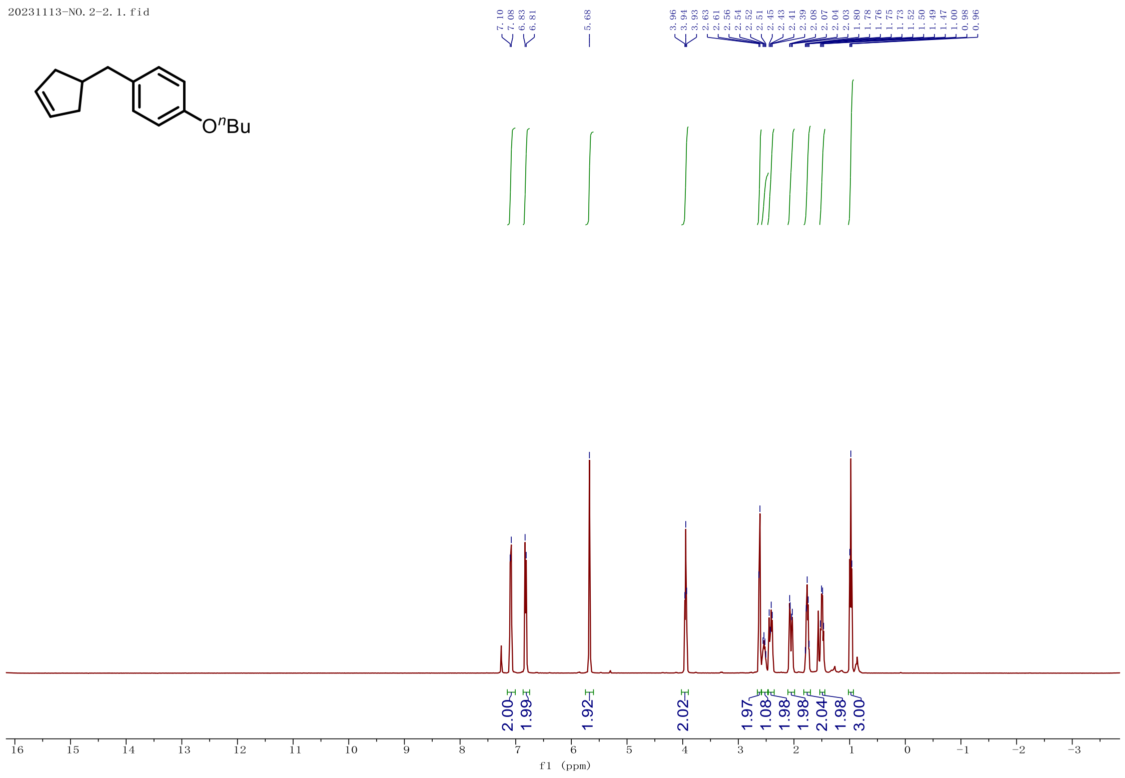

^{13}C NMR of Compound 24 (101 MHz, $CDCl_3$)

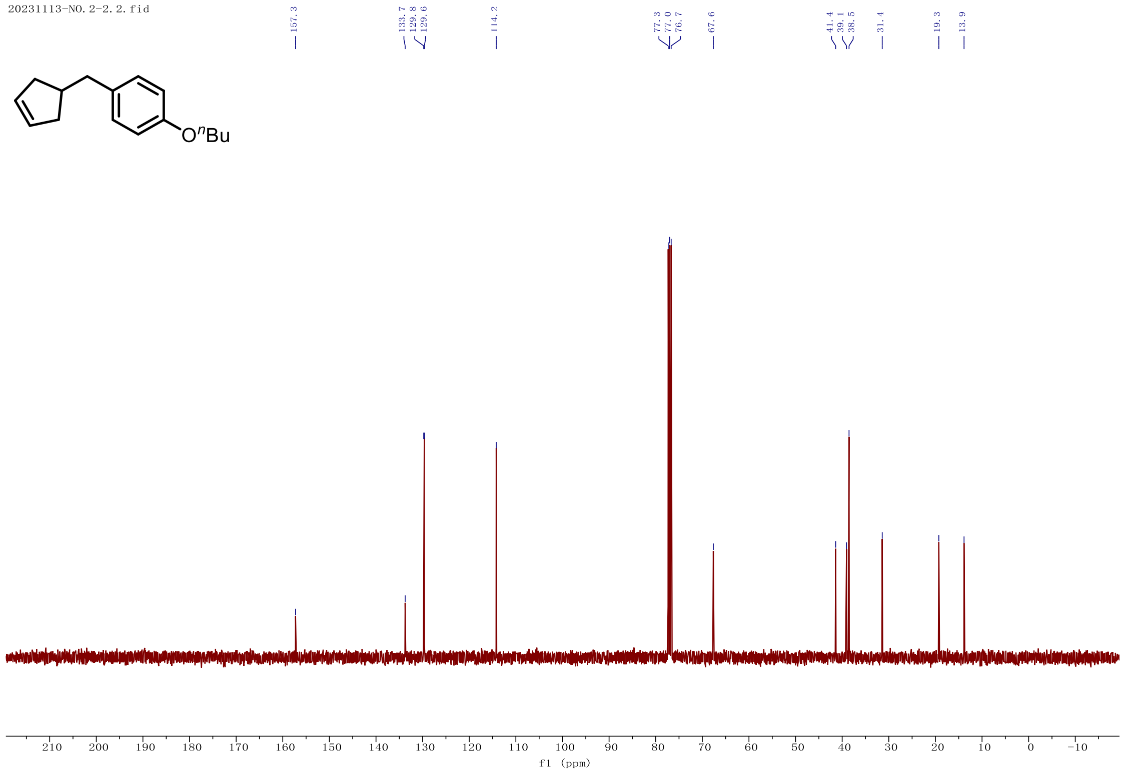

^{1}H NMR of Compound 25 (400 MHz, $CDCl_3$)

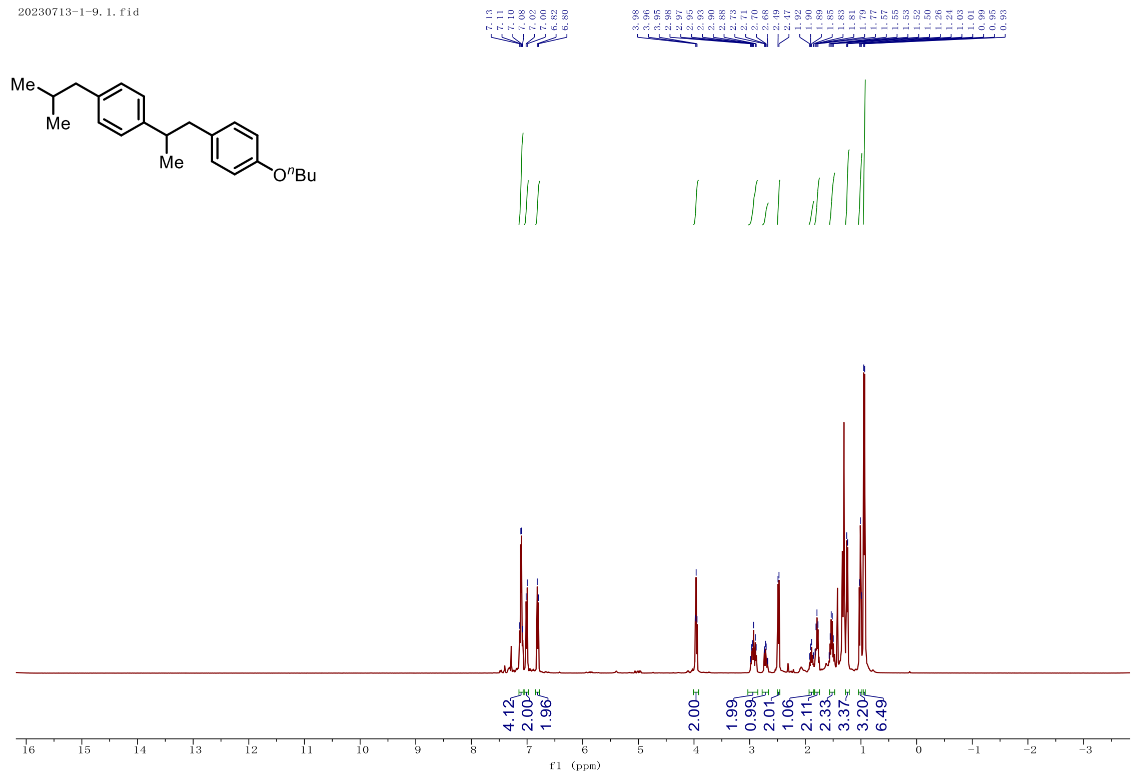

^{13}C NMR of Compound 25 (101 MHz, $CDCl_3$)

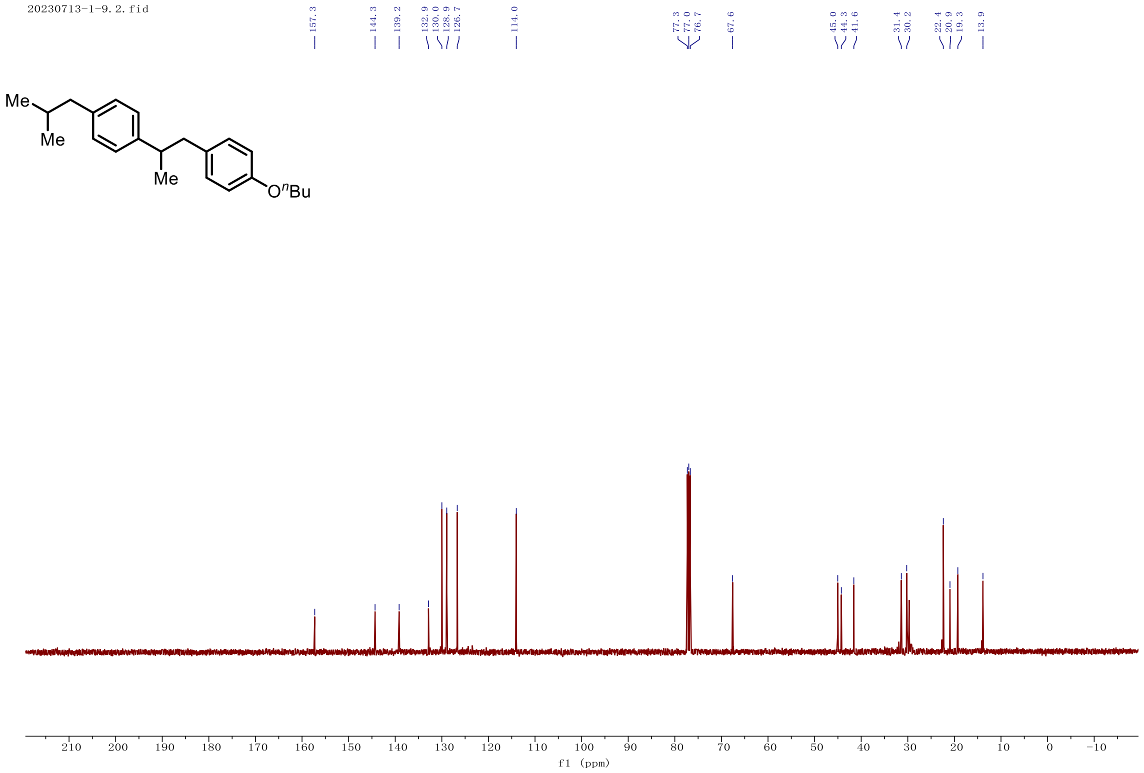

^{1}H NMR of Compound 26 (400 MHz, $CDCl_3$)

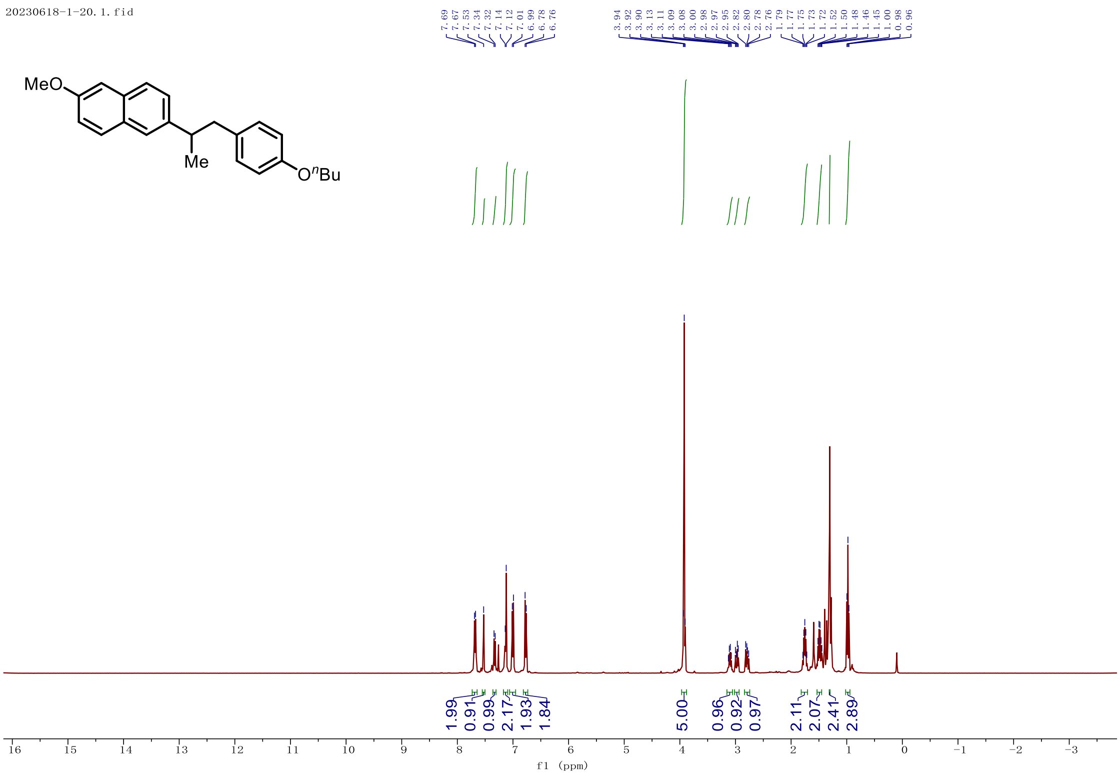

^{13}C NMR of Compound 26 (101 MHz, $CDCl_3$)

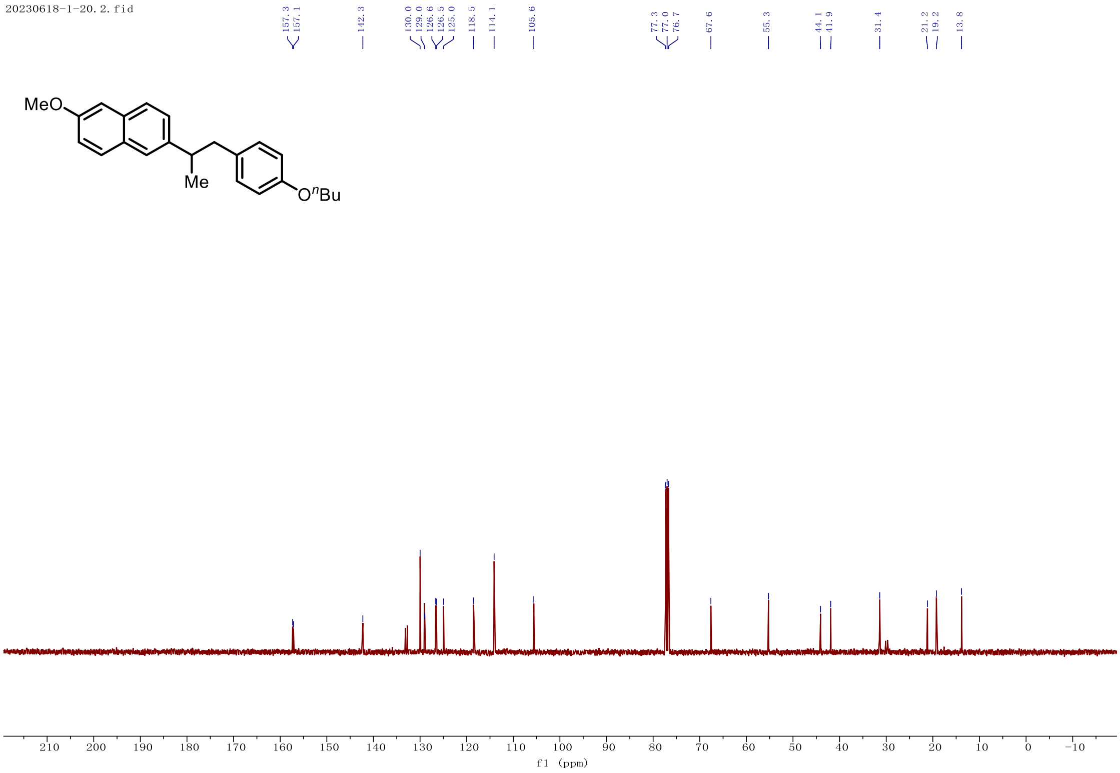

^{1}H NMR of Compound 27 (400 MHz, $CDCl_3$)

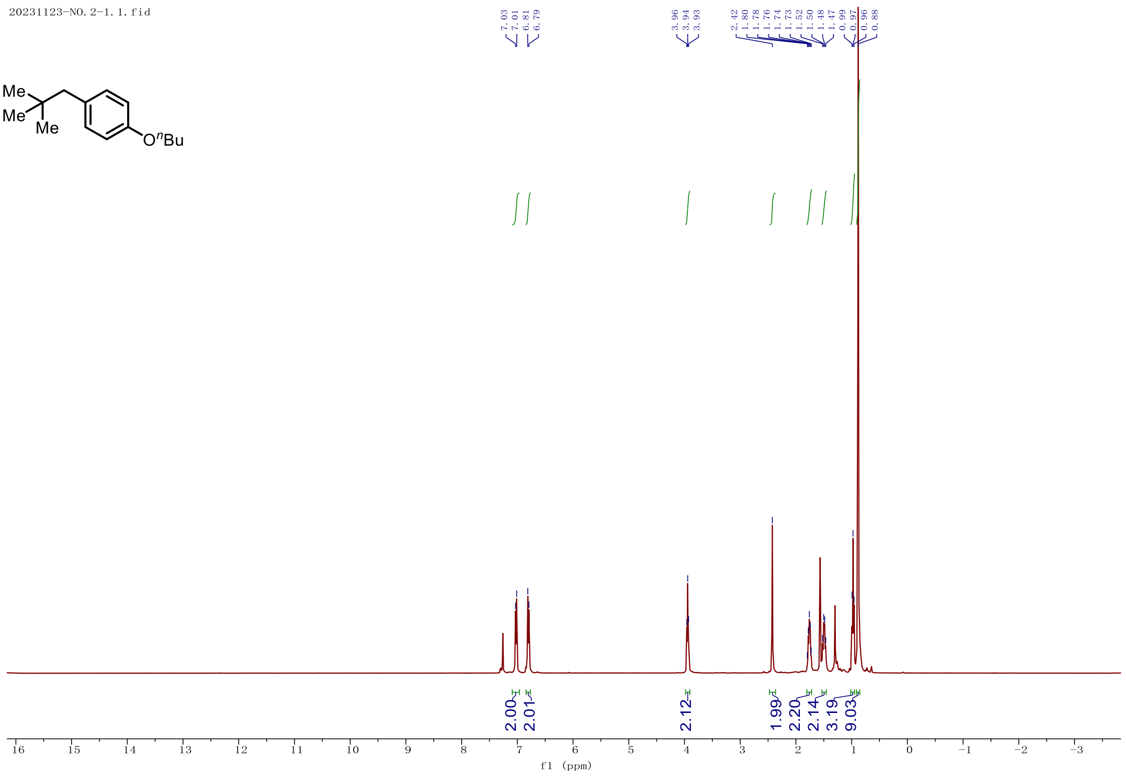

^{13}C NMR of Compound 27 (101 MHz, $CDCl_3$)

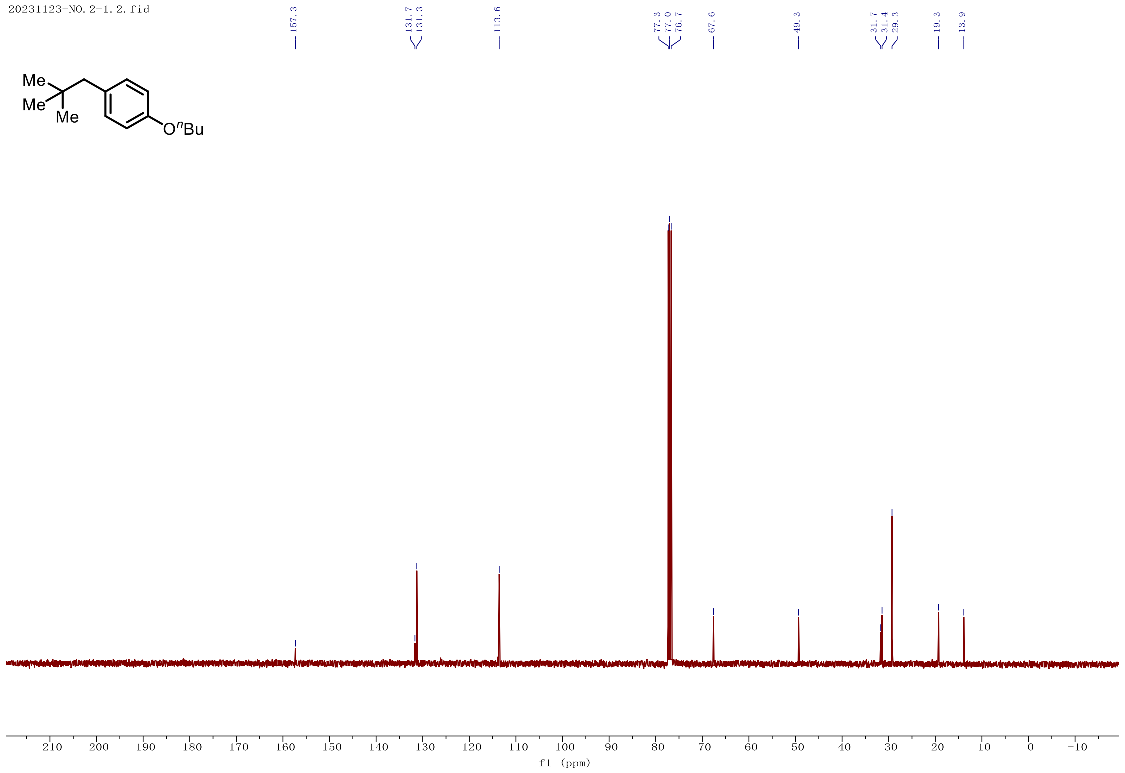

^{1}H NMR of Compound 28 (400 MHz, $CDCl_3$)

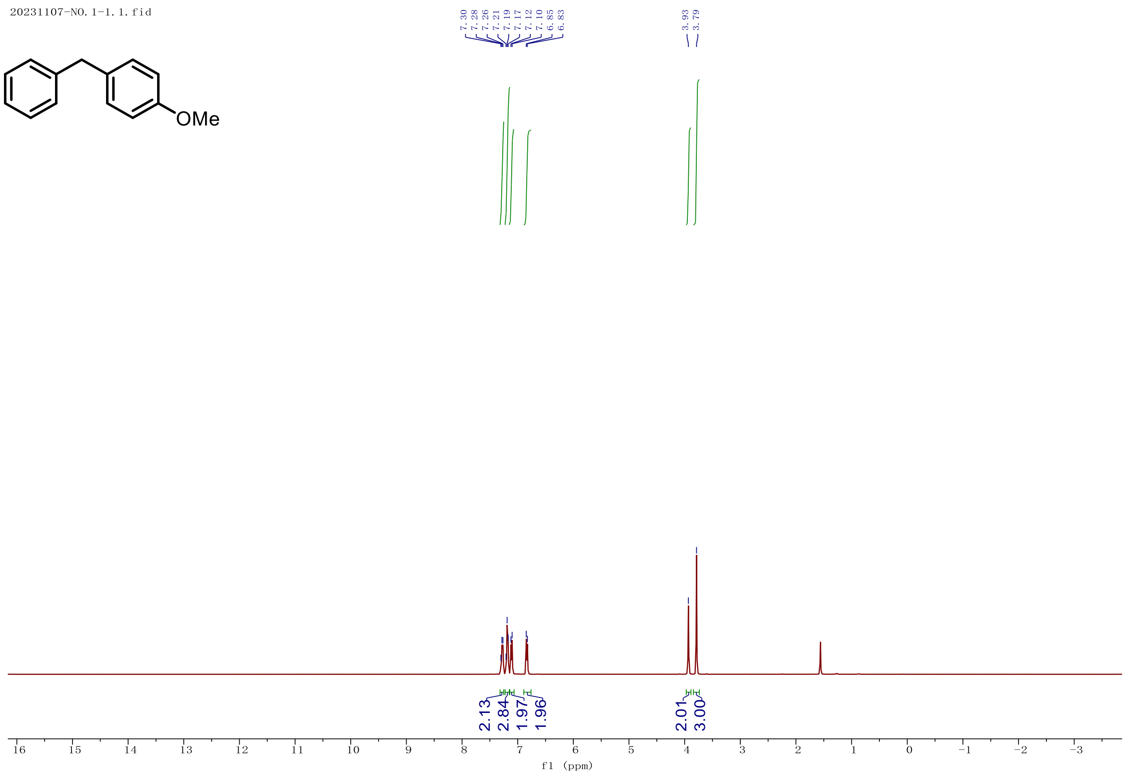

^{13}C NMR of Compound 28 (101 MHz, $CDCl_3$)

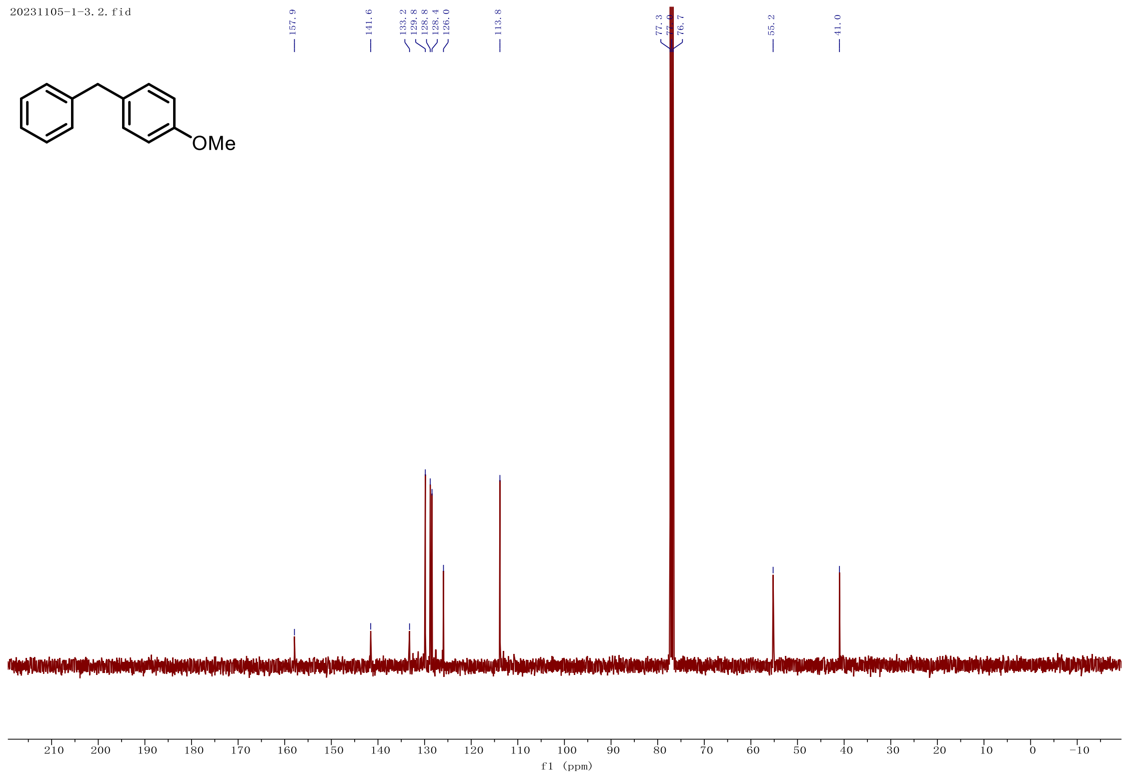

^{1}H NMR of Compound 29 (400 MHz, $CDCl_3$)

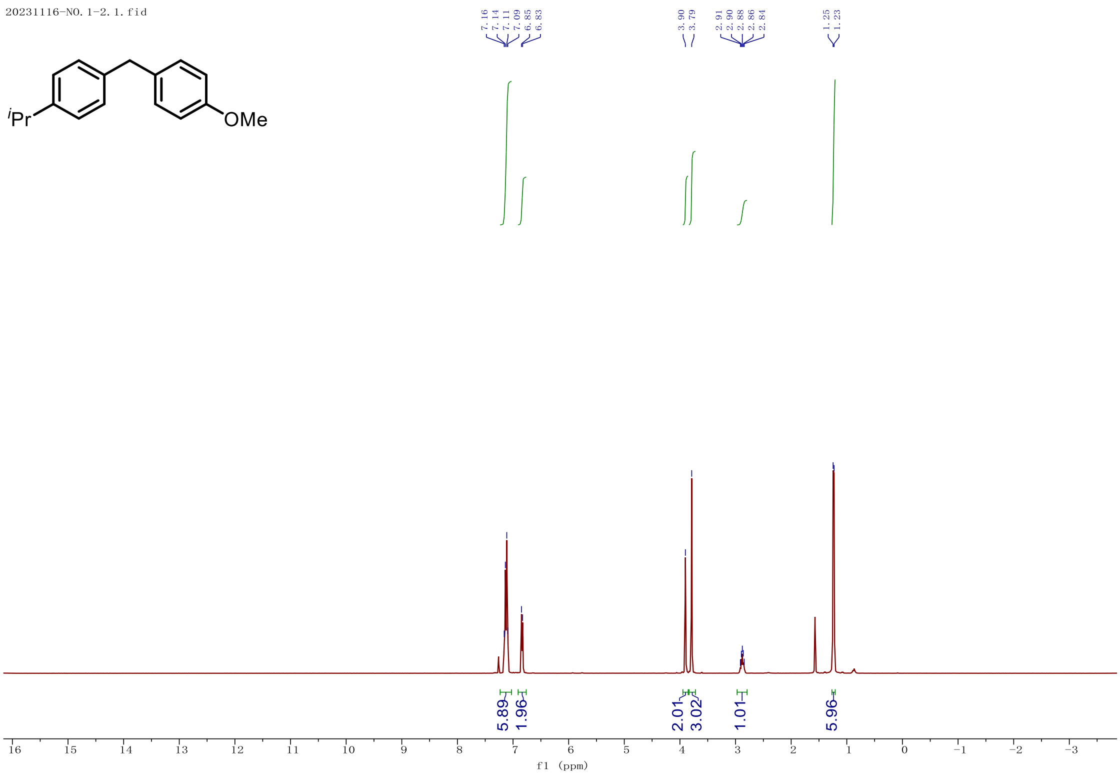

^{13}C NMR of Compound 29 (101 MHz, $CDCl_3$)

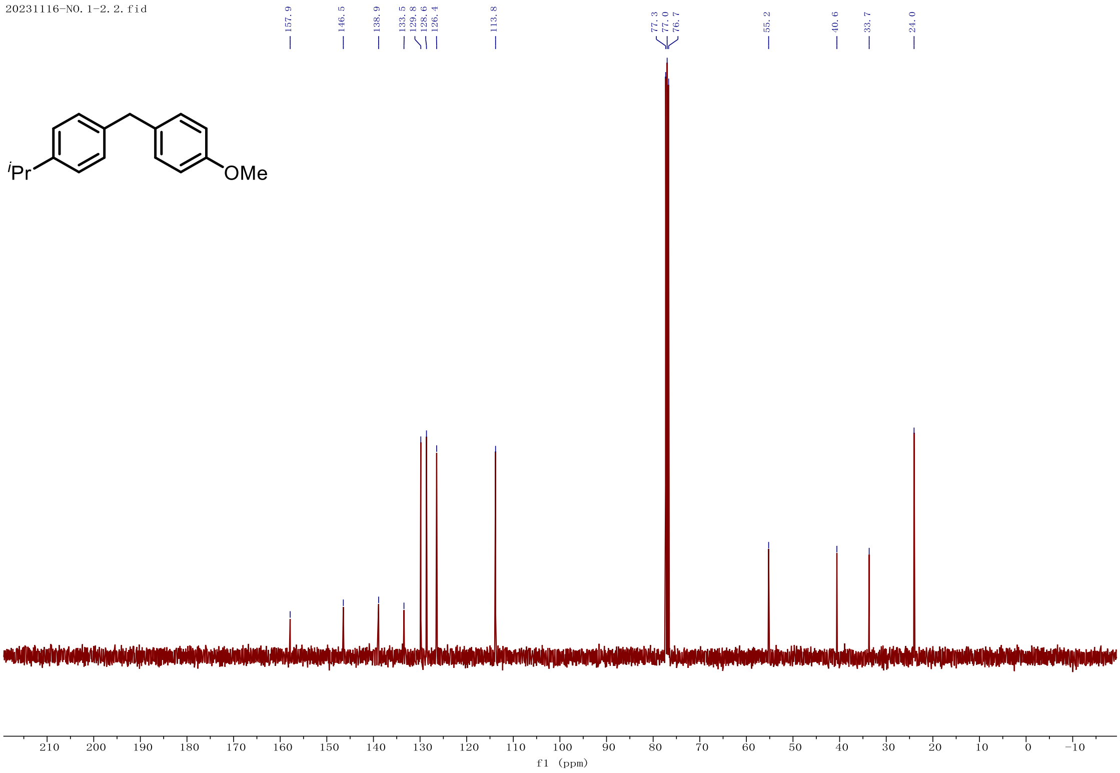

^{1}H NMR of Compound 30 (400 MHz, $CDCl_3$)

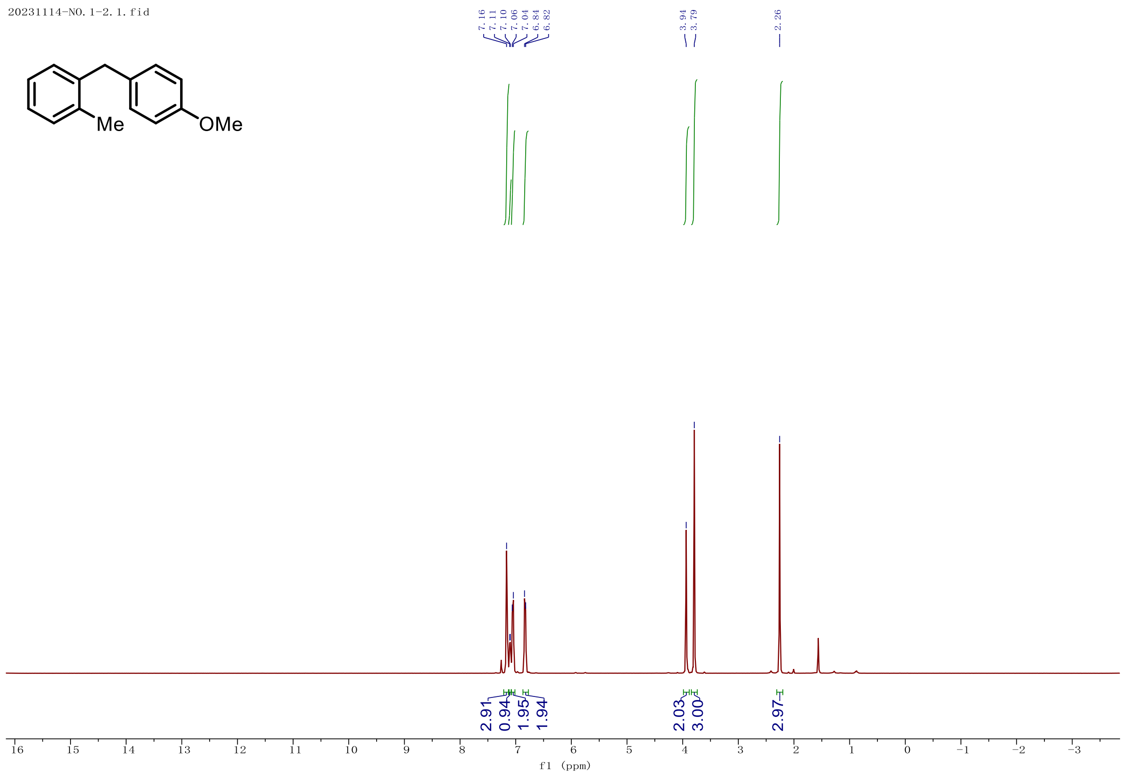

^{13}C NMR of Compound 30 (101 MHz, $CDCl_3$)

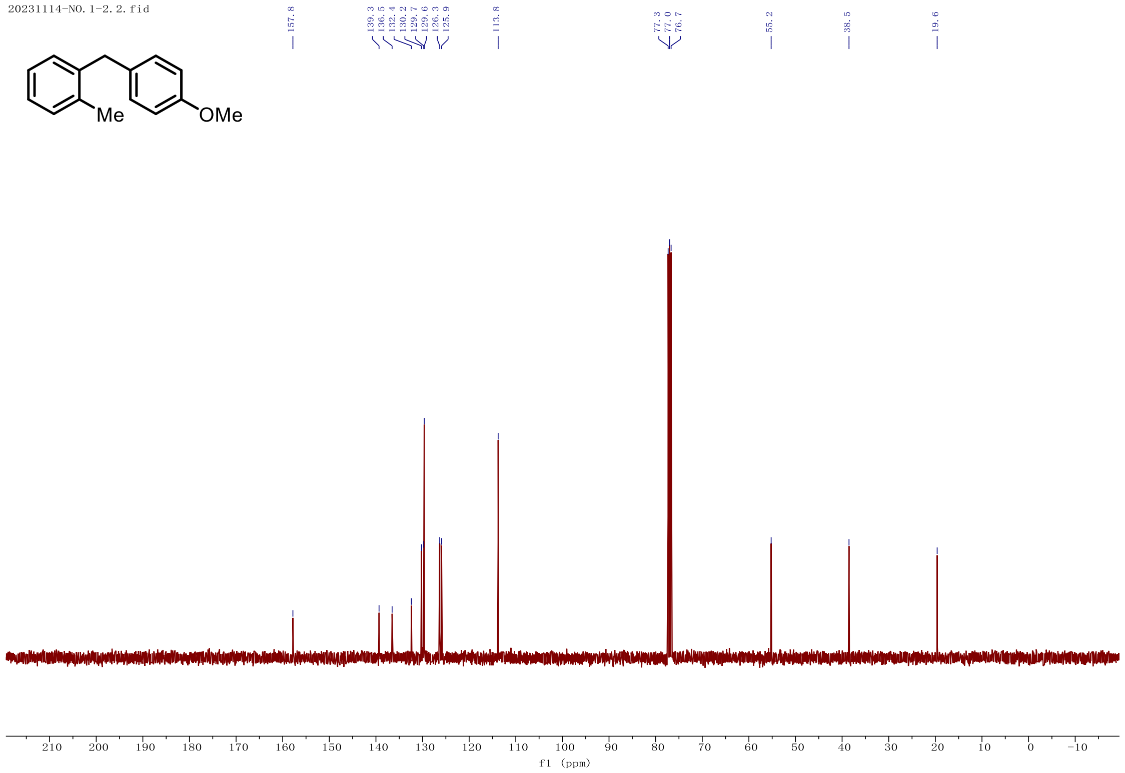

^{1}H NMR of Compound 31 (400 MHz, $CDCl_3$)

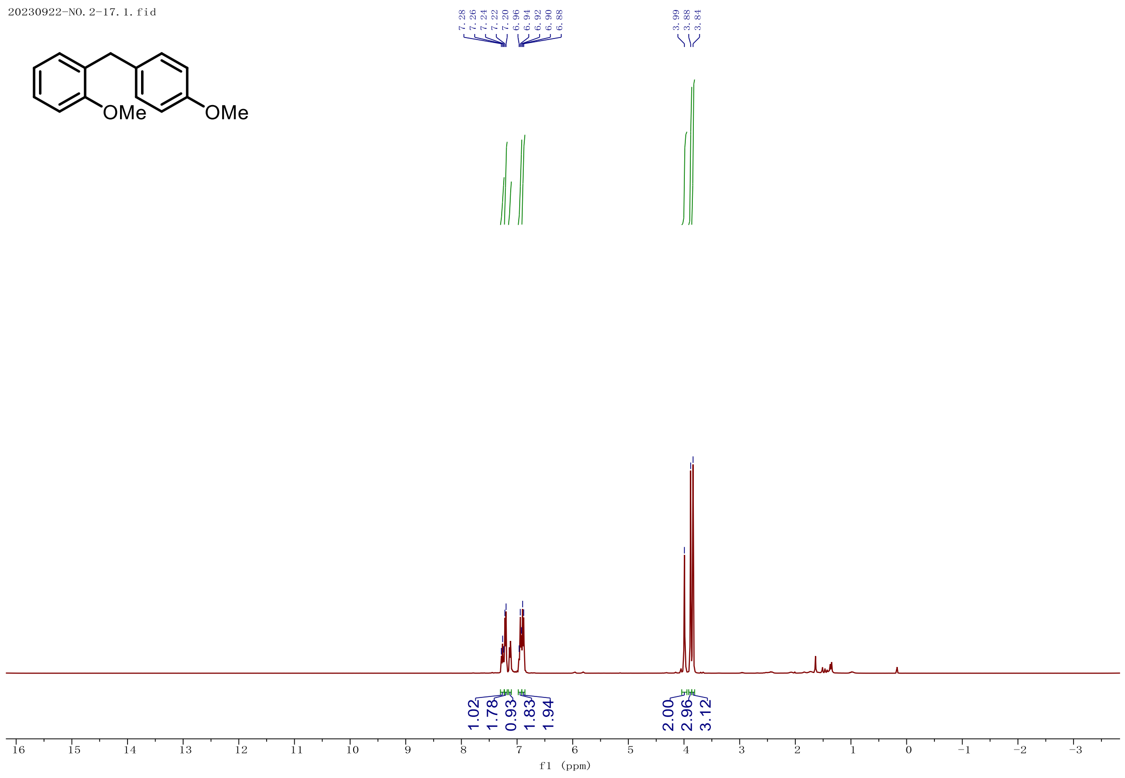

^{13}C NMR of Compound 31 (101 MHz, $CDCl_3$)

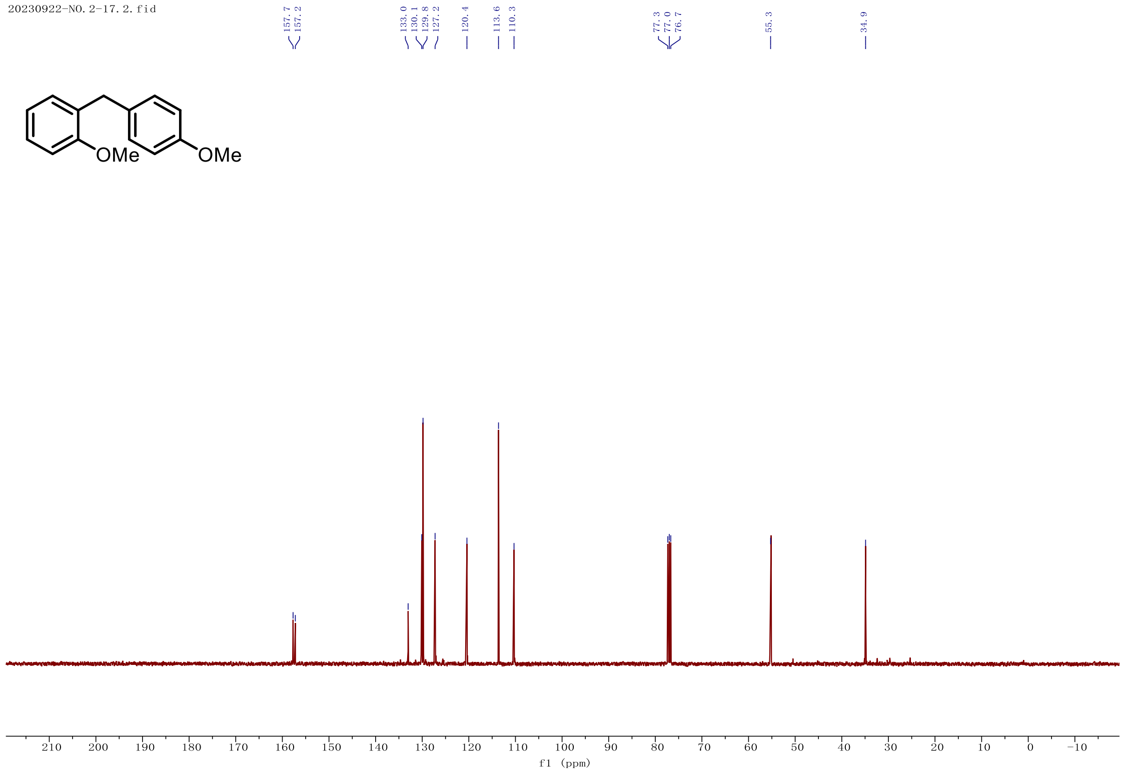

^{1}H NMR of Compound 32 (400 MHz, $CDCl_3$)

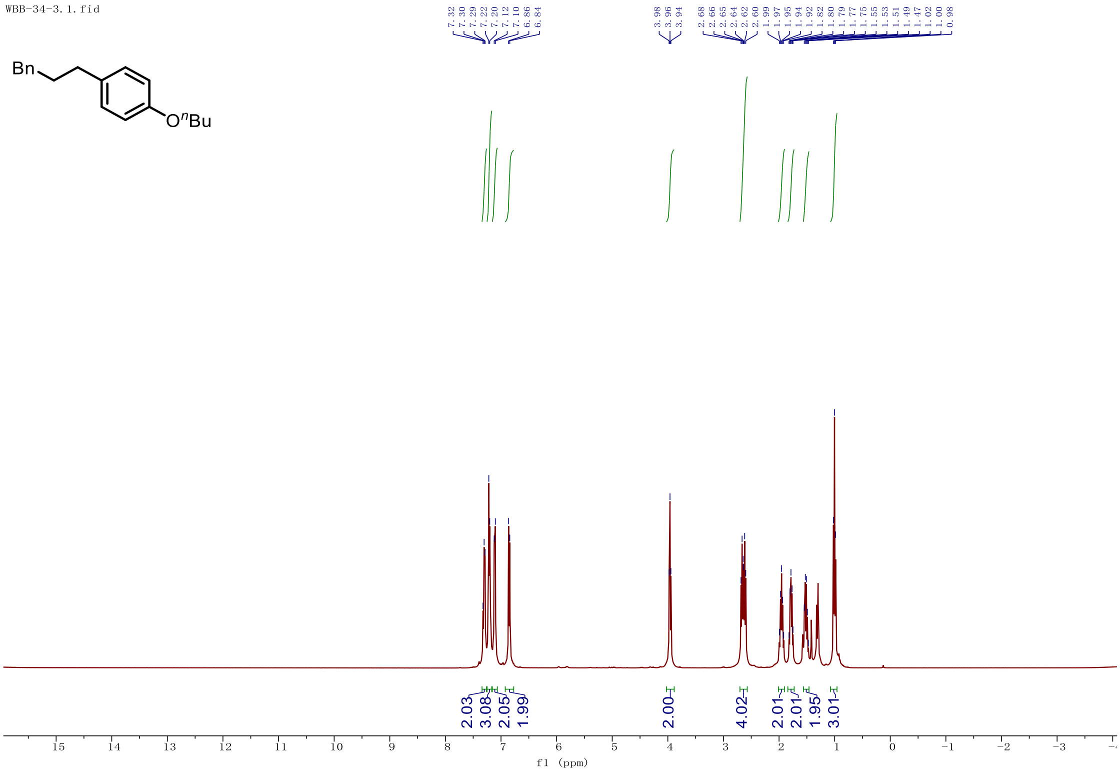

^{13}C NMR of Compound 32 (101 MHz, $CDCl_3$)

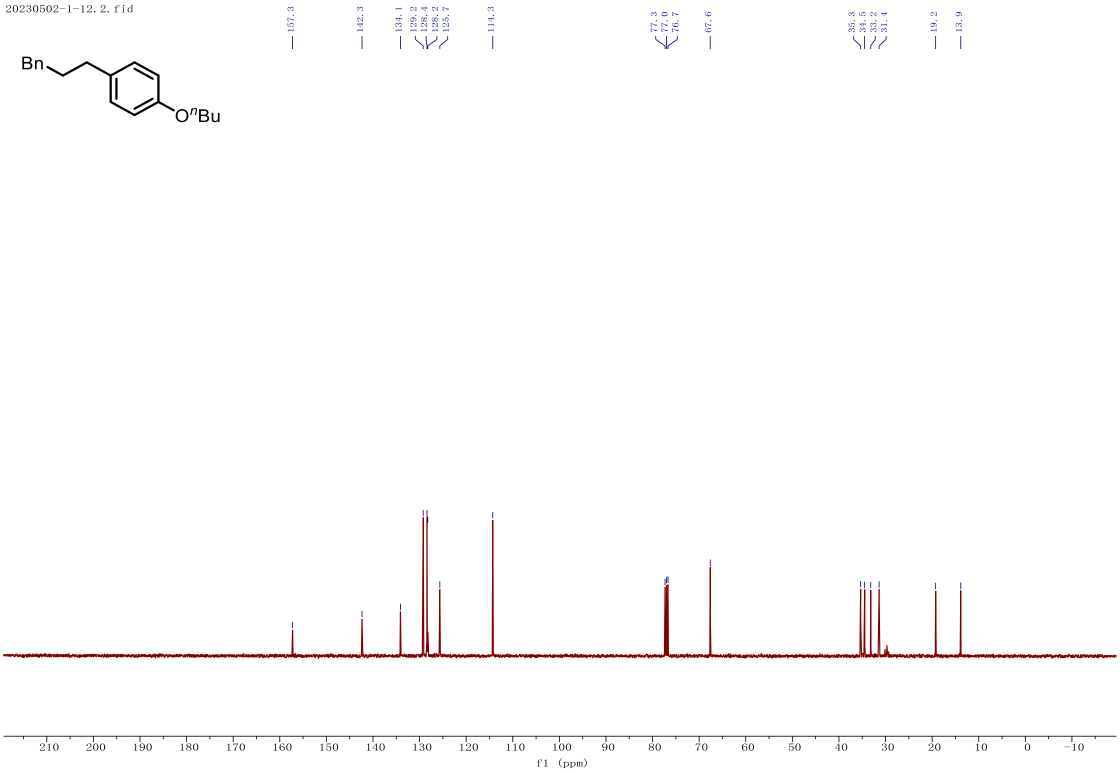

^{1}H NMR of Compound 33 (400 MHz, $CDCl_3$)

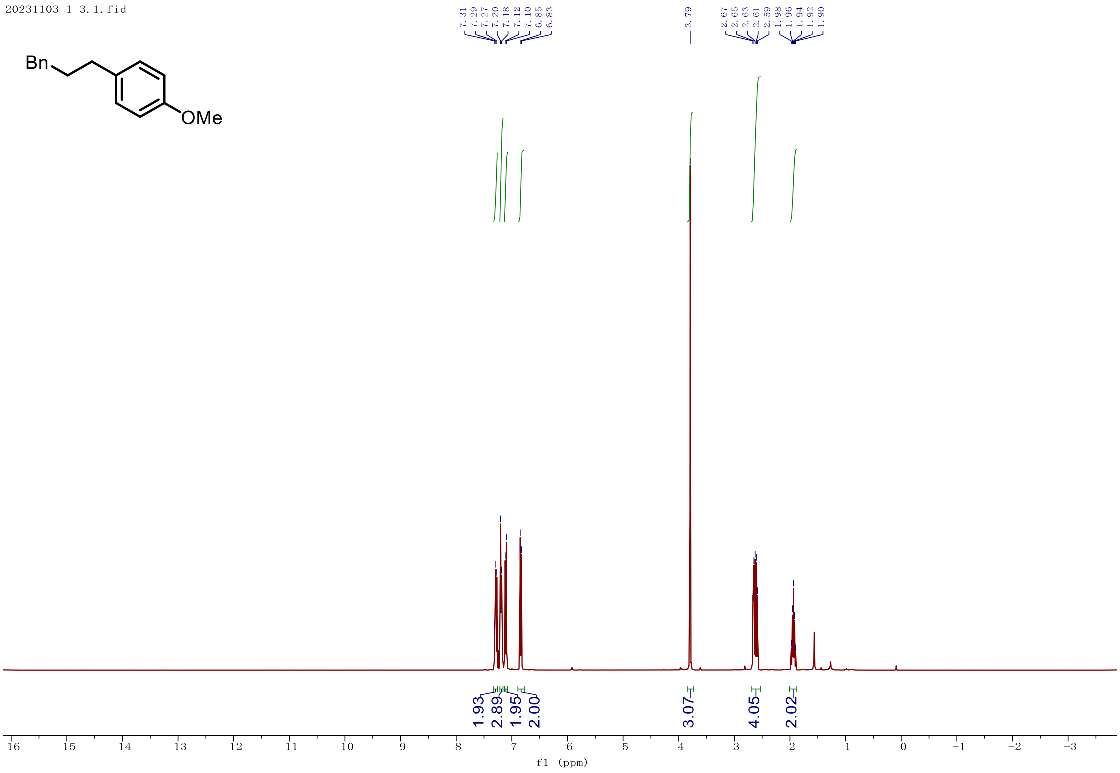

^{13}C NMR of Compound 33 (101 MHz, $CDCl_3$)

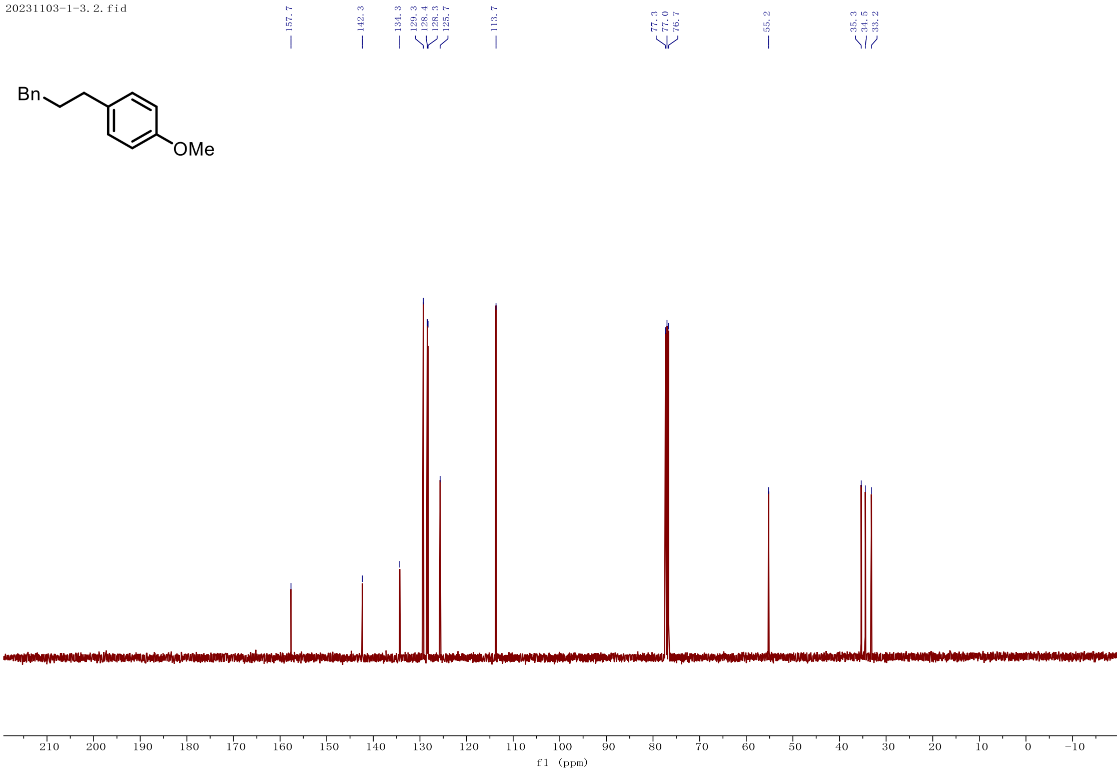

^{1}H NMR of Compound 34 (400 MHz, $CDCl_3$)

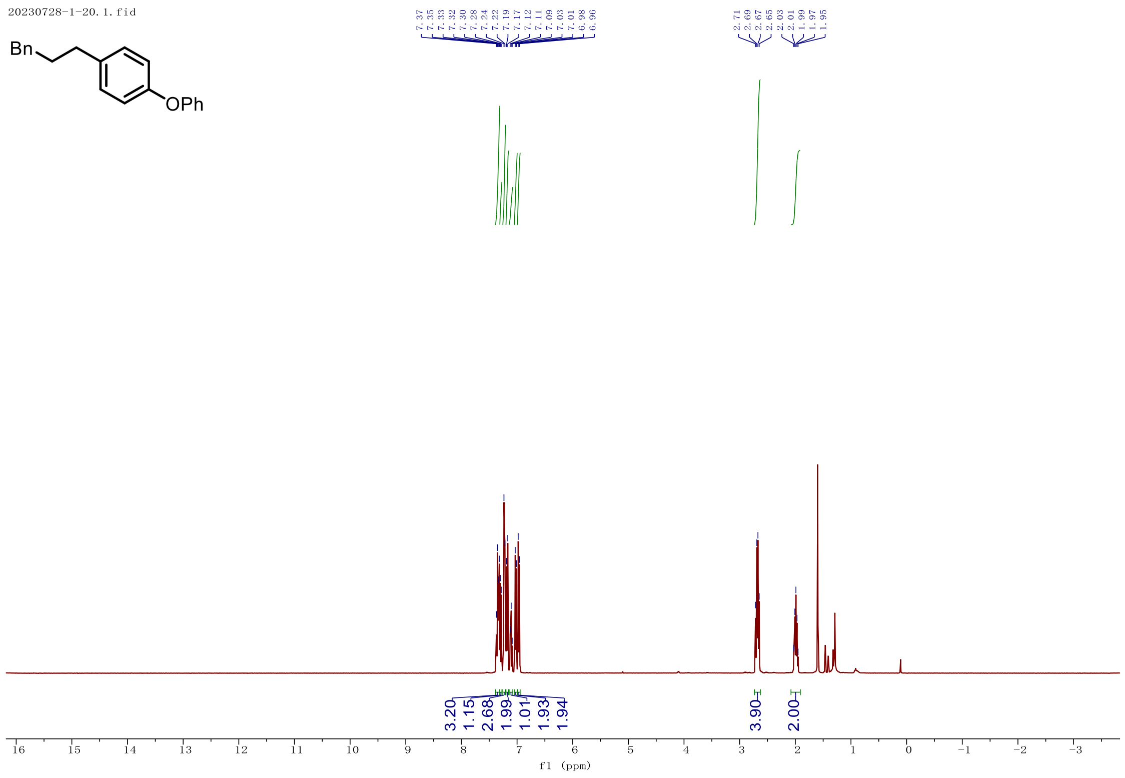

^{13}C NMR of Compound 34 (101 MHz, $CDCl_3$)

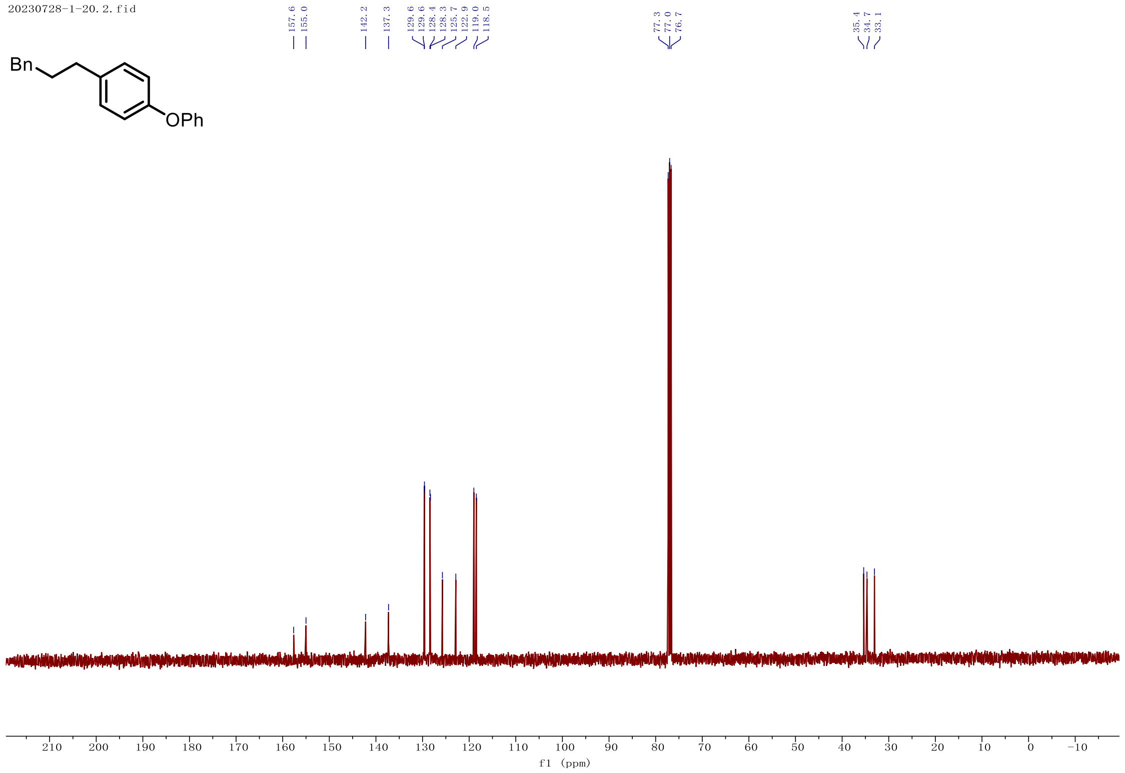

^{1}H NMR of Compound 35 (400 MHz, $CDCl_3$)

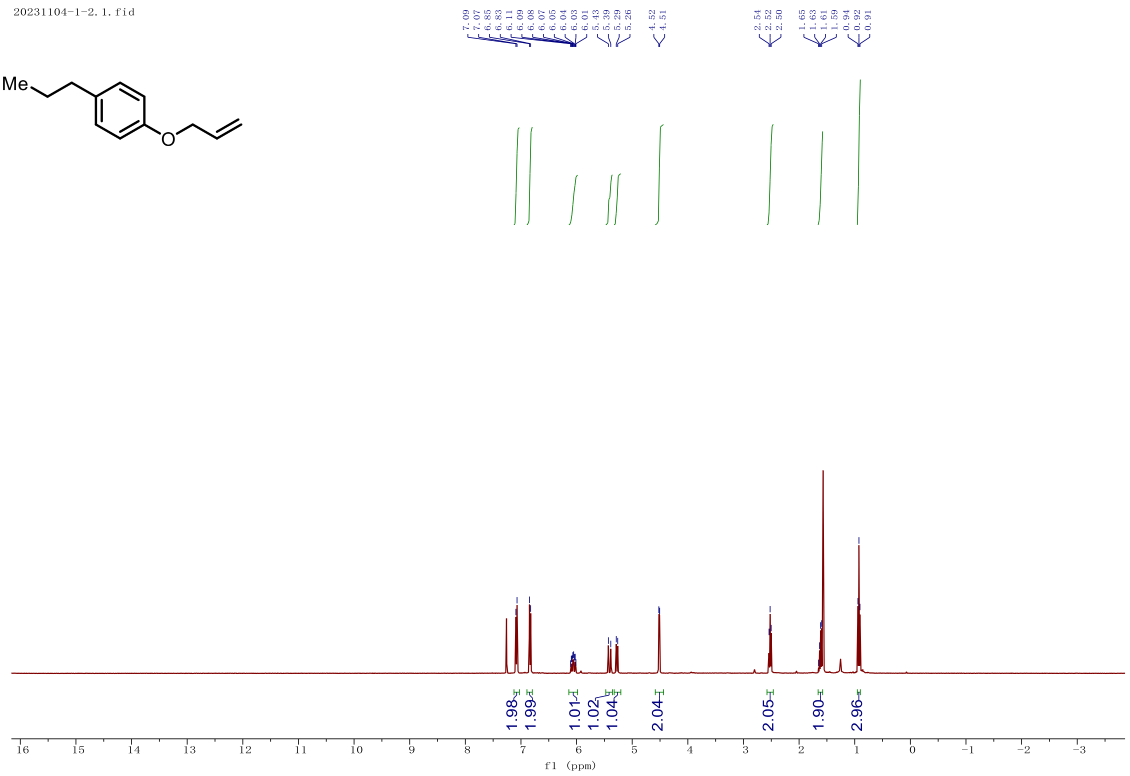

^{13}C NMR of Compound 35 (101 MHz, $CDCl_3$)

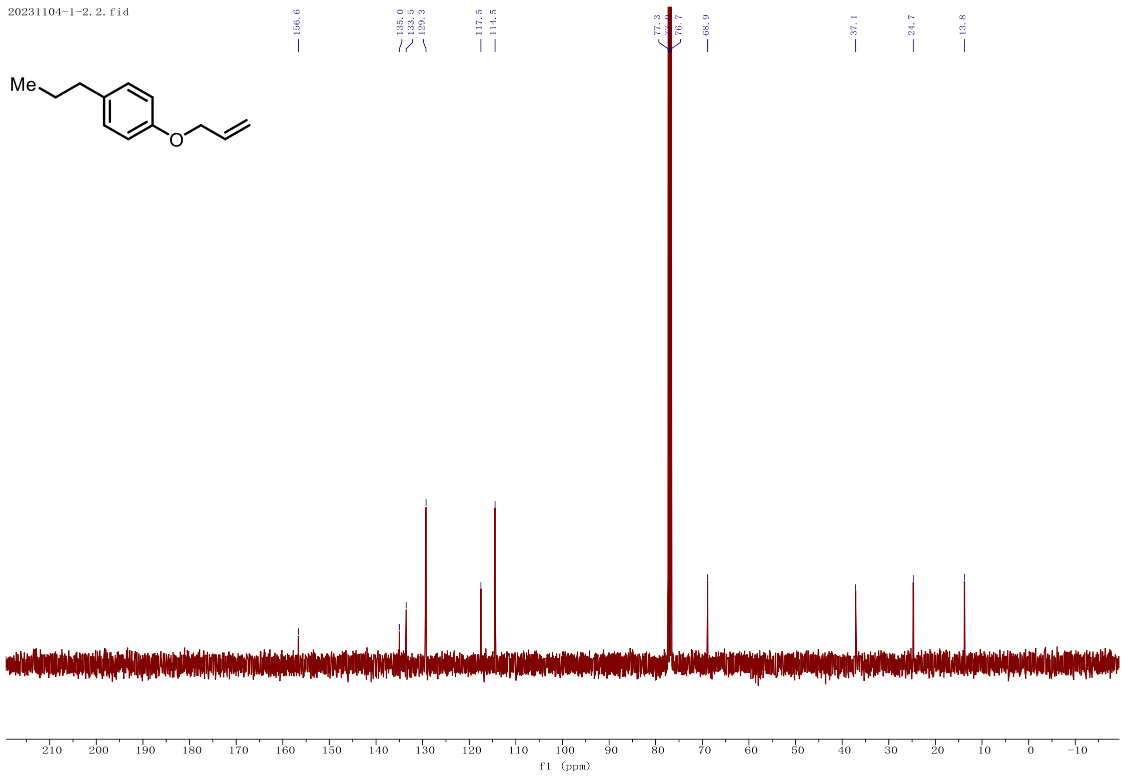

^{1}H NMR of Compound 36 (400 MHz, $CDCl_3$)

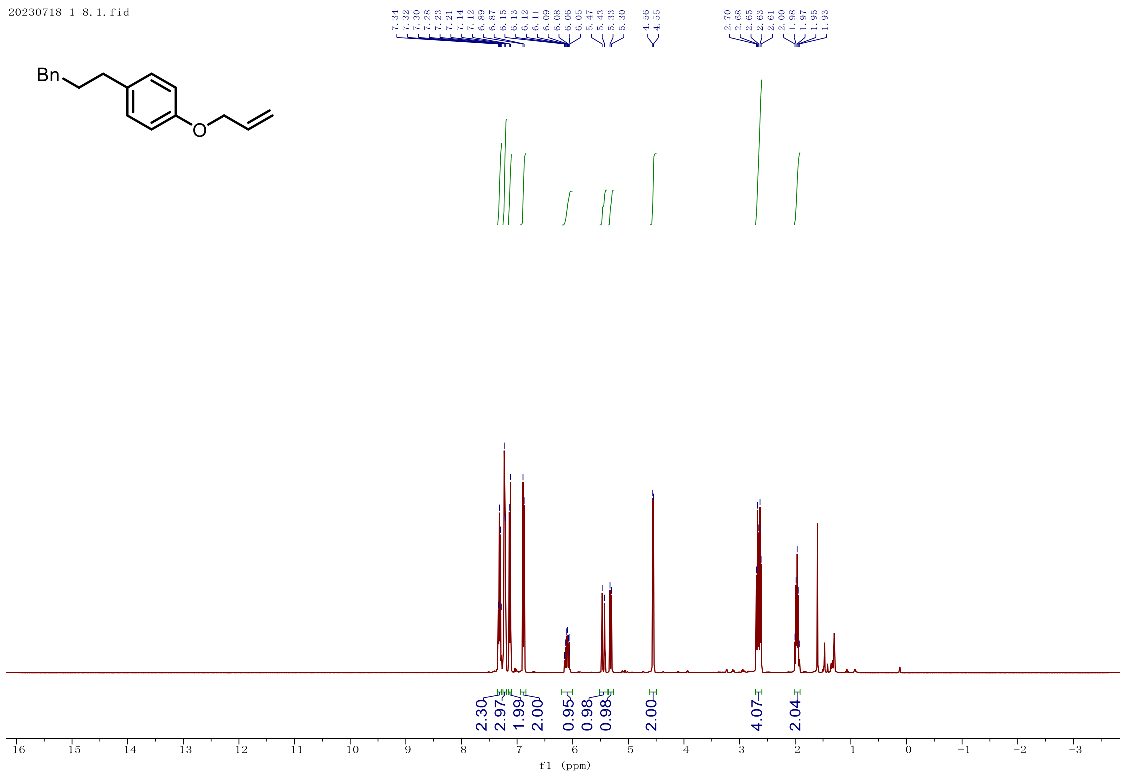

^{13}C NMR of Compound 36 (101 MHz, $CDCl_3$)

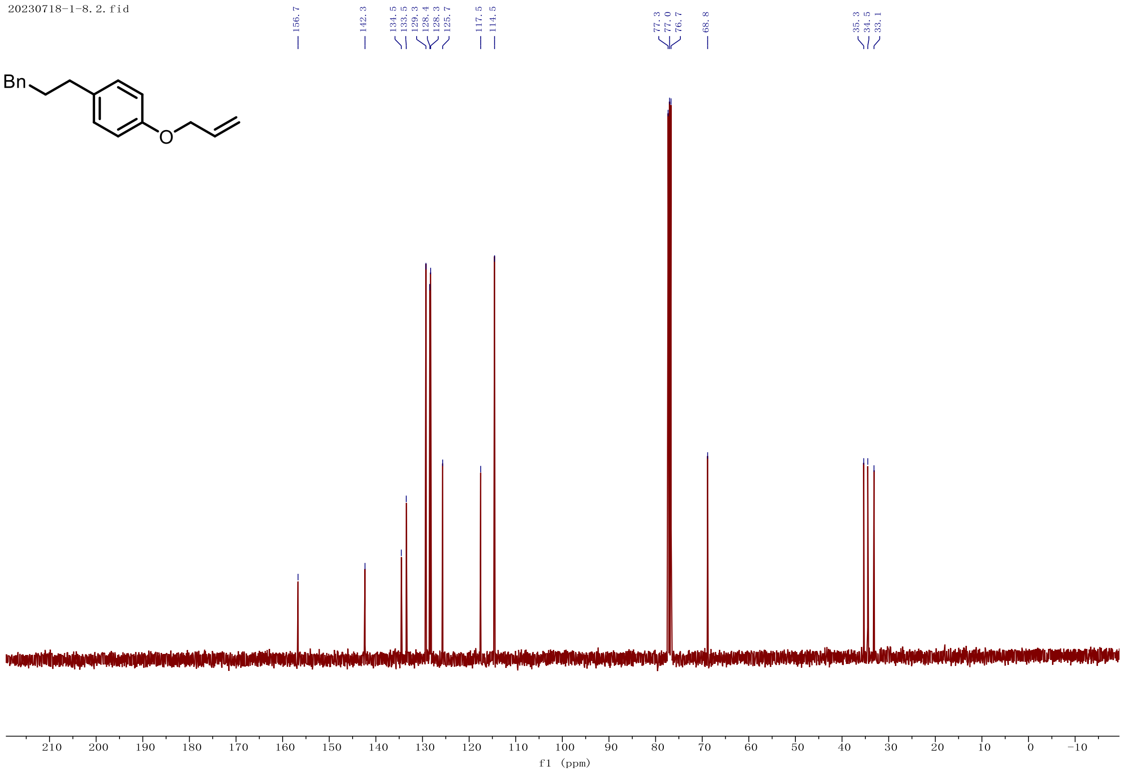

^{1}H NMR of Compound 37 (400 MHz, $CDCl_3$)

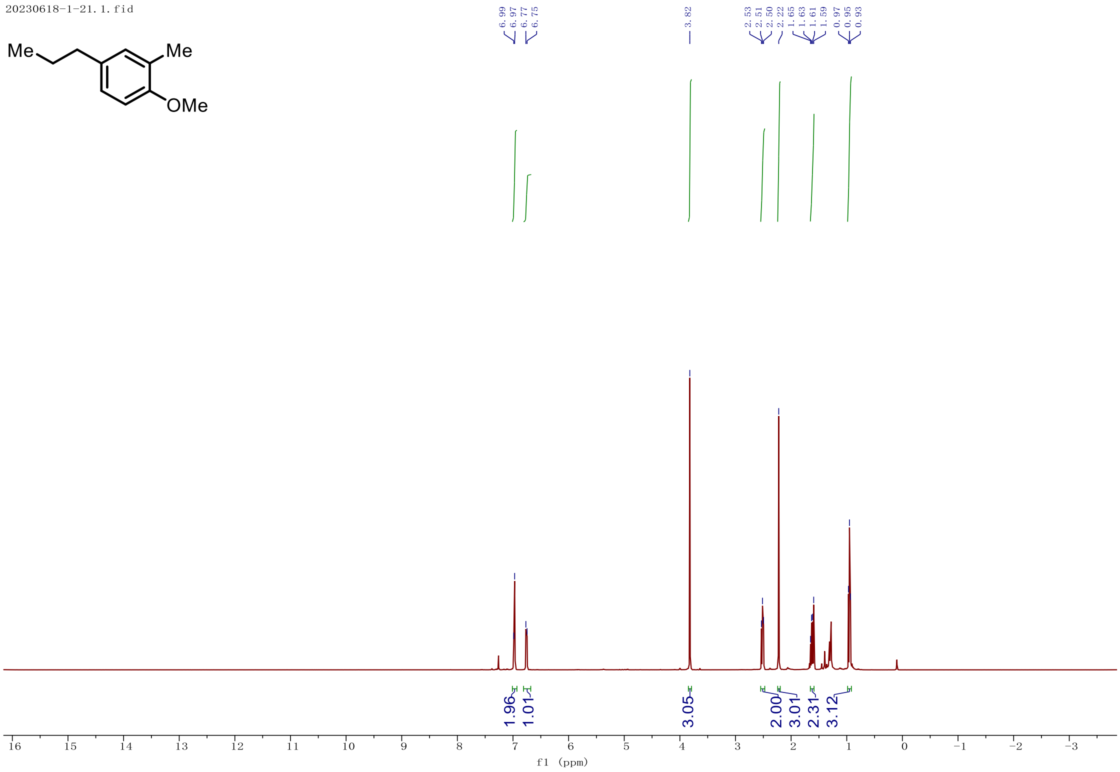

^{13}C NMR of Compound 37 (101 MHz, $CDCl_3$)

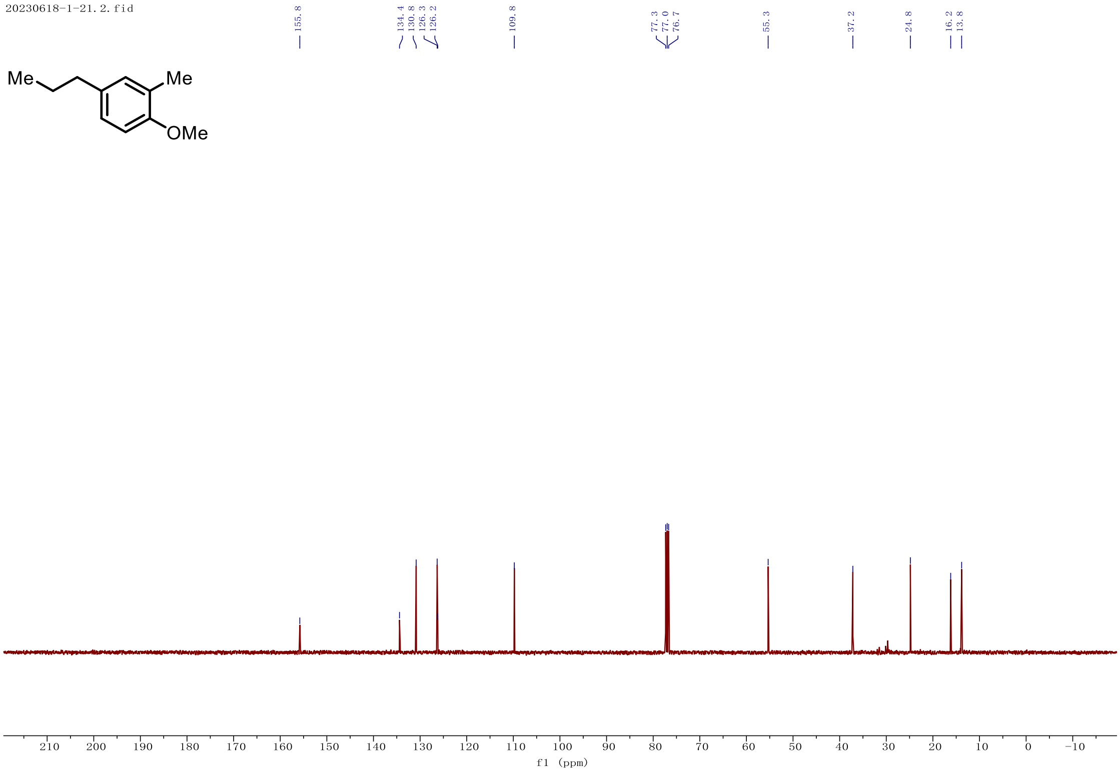

^{1}H NMR of Compound 38 (400 MHz, $CDCl_3$)

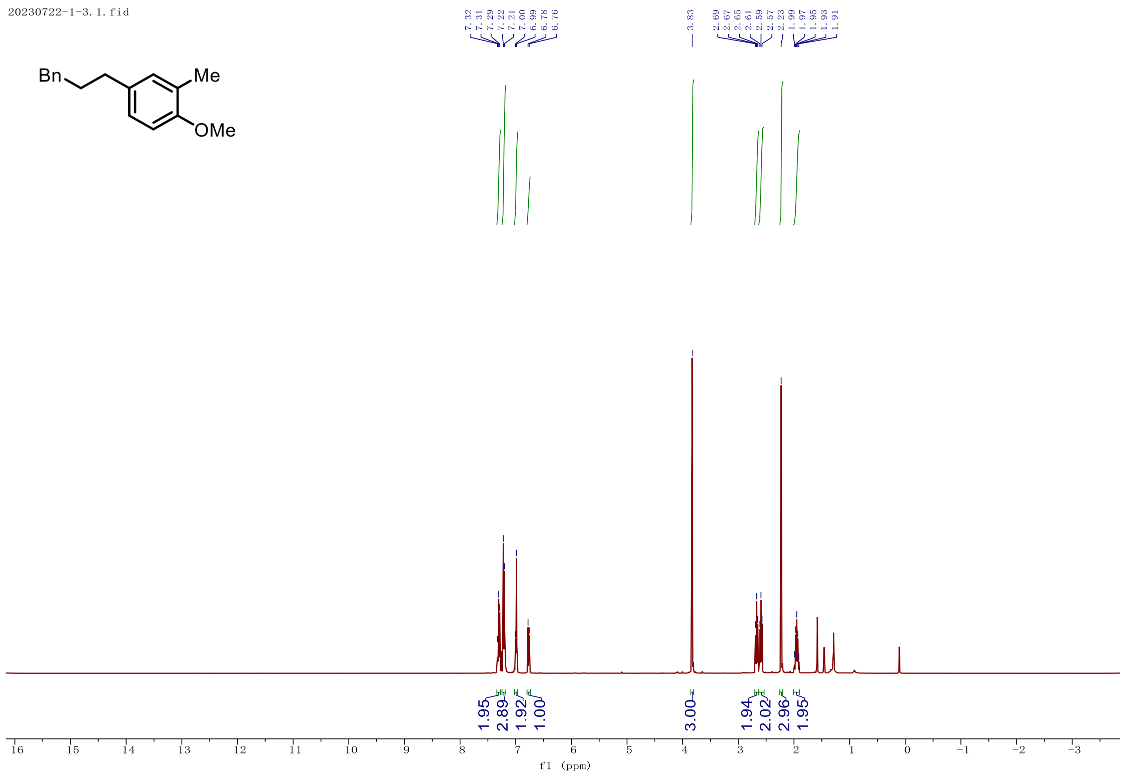

^{13}C NMR of Compound 38 (101 MHz, $CDCl_3$)

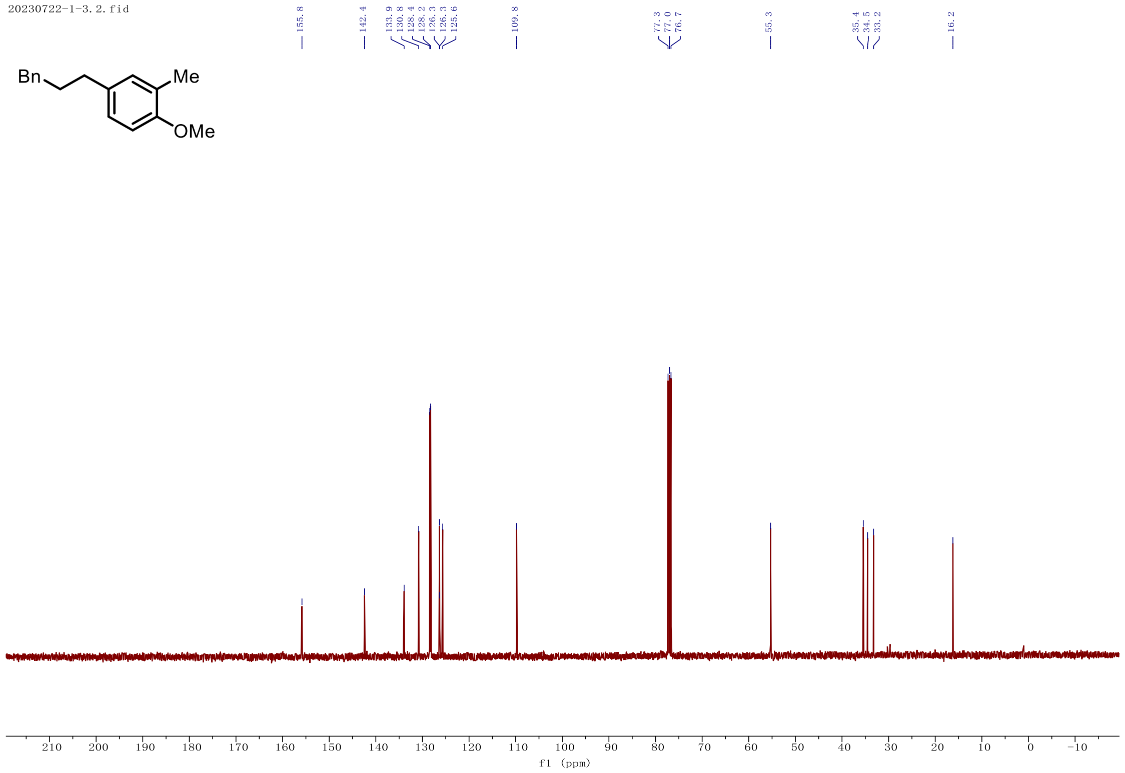

^{1}H NMR of Compound 39 (400 MHz, $CDCl_3$)

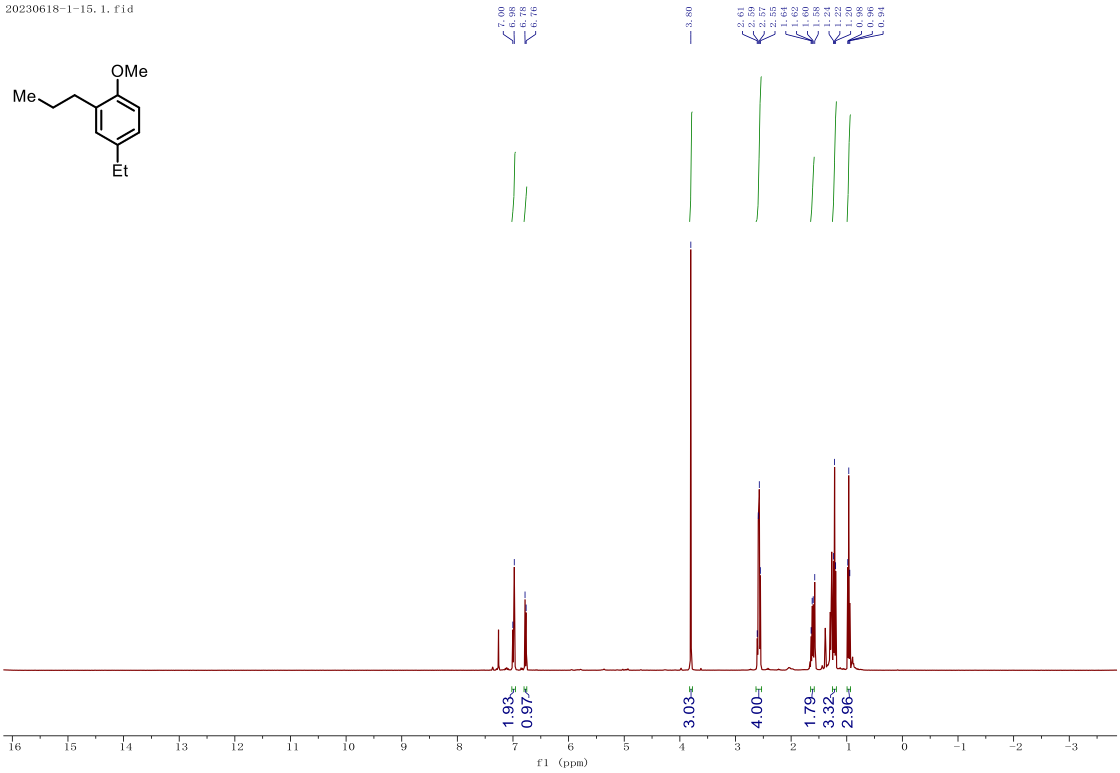

^{13}C NMR of Compound 39 (101 MHz, $CDCl_3$)

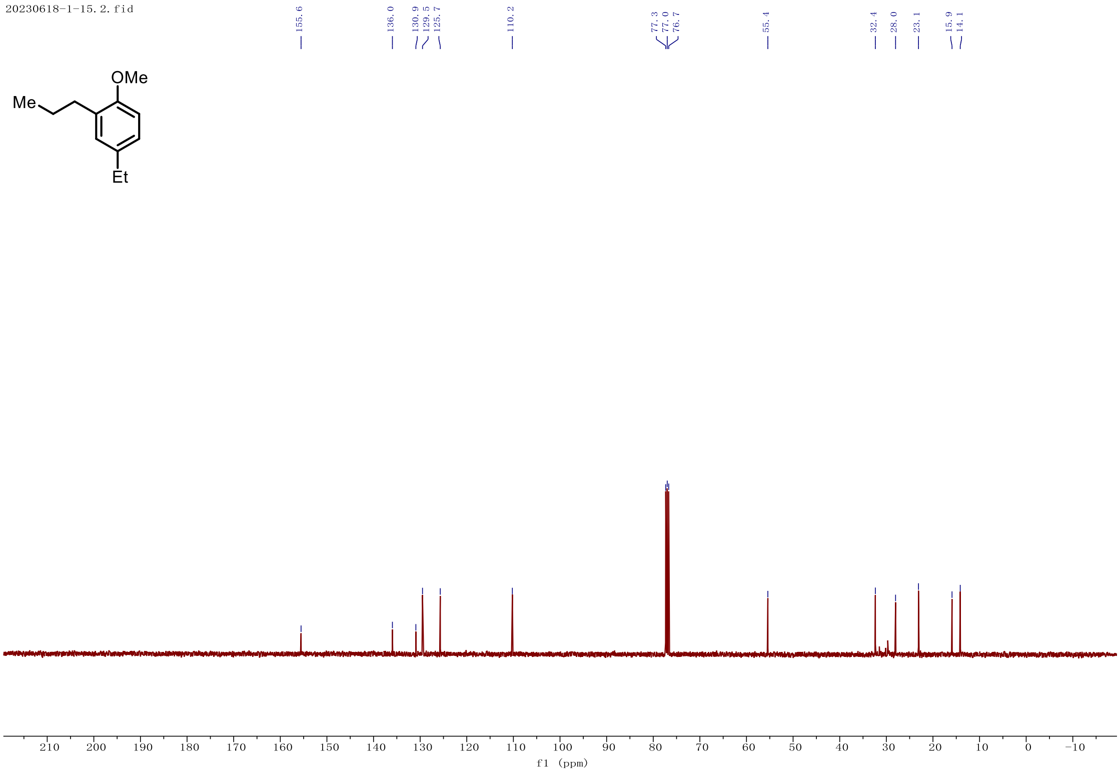

^{1}H NMR of Compound 40 (400 MHz, $CDCl_3$)

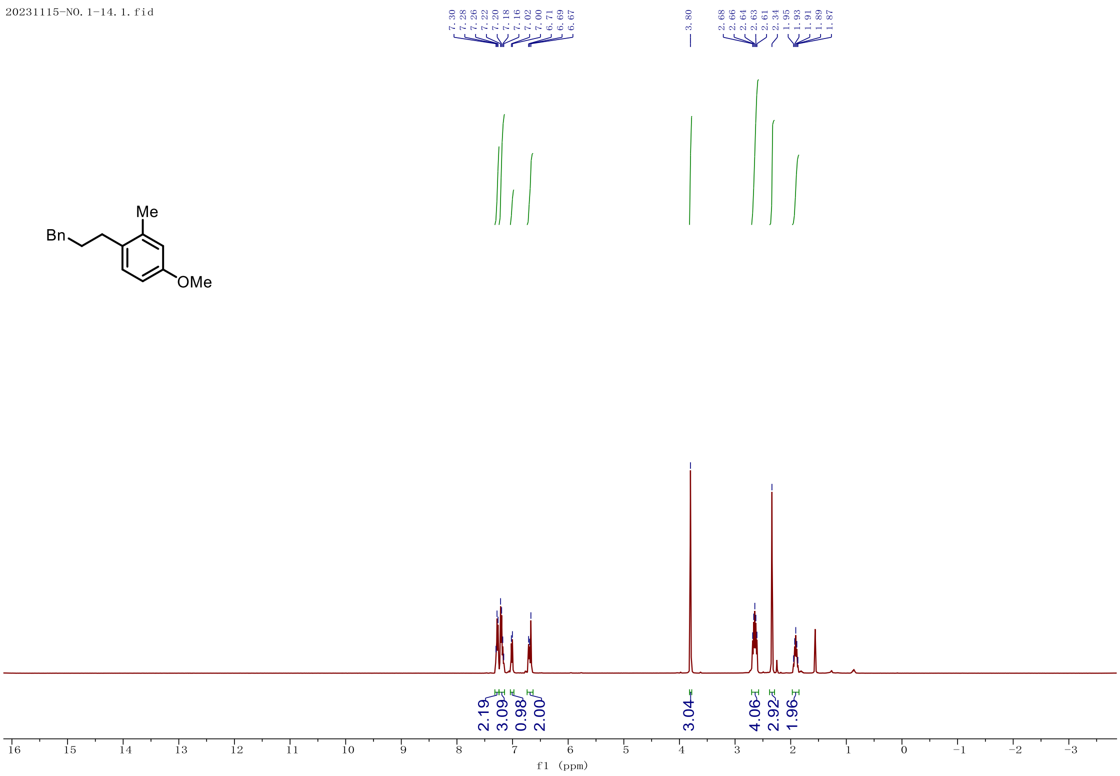

^{13}C NMR of Compound 40 (101 MHz, $CDCl_3$)

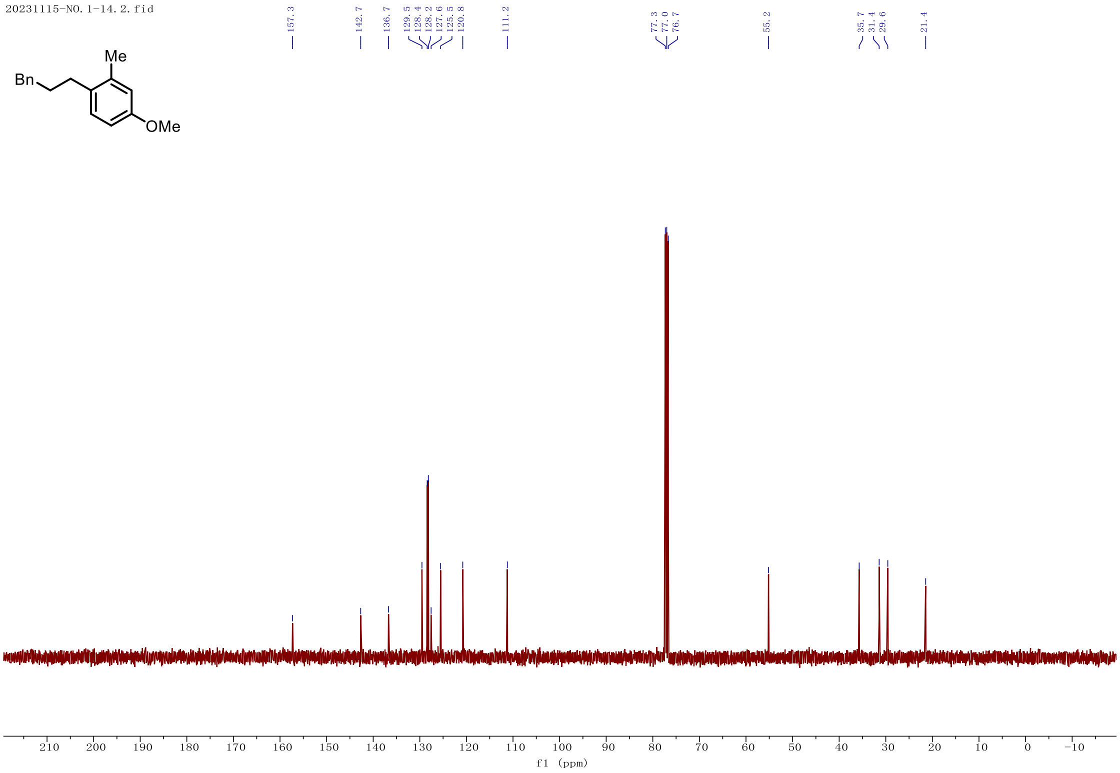

^{1}H NMR of Compound 41 (400 MHz, $CDCl_3$)

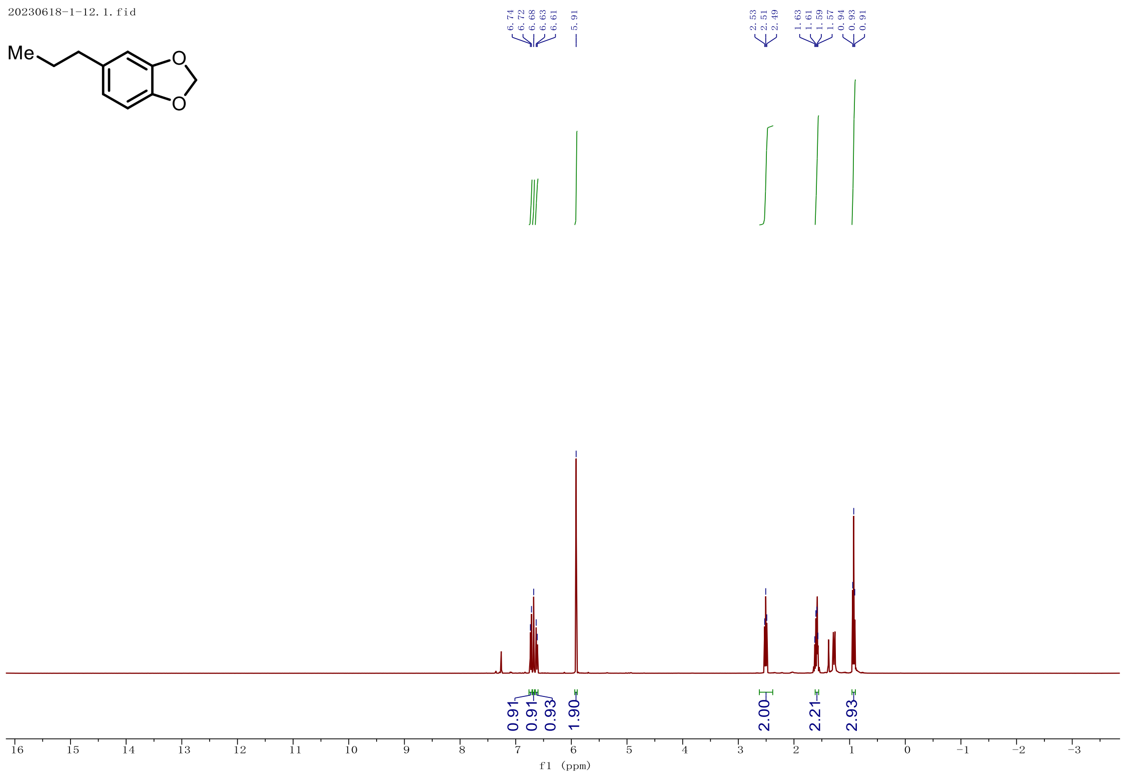

^{13}C NMR of Compound 41 (101 MHz, $CDCl_3$)

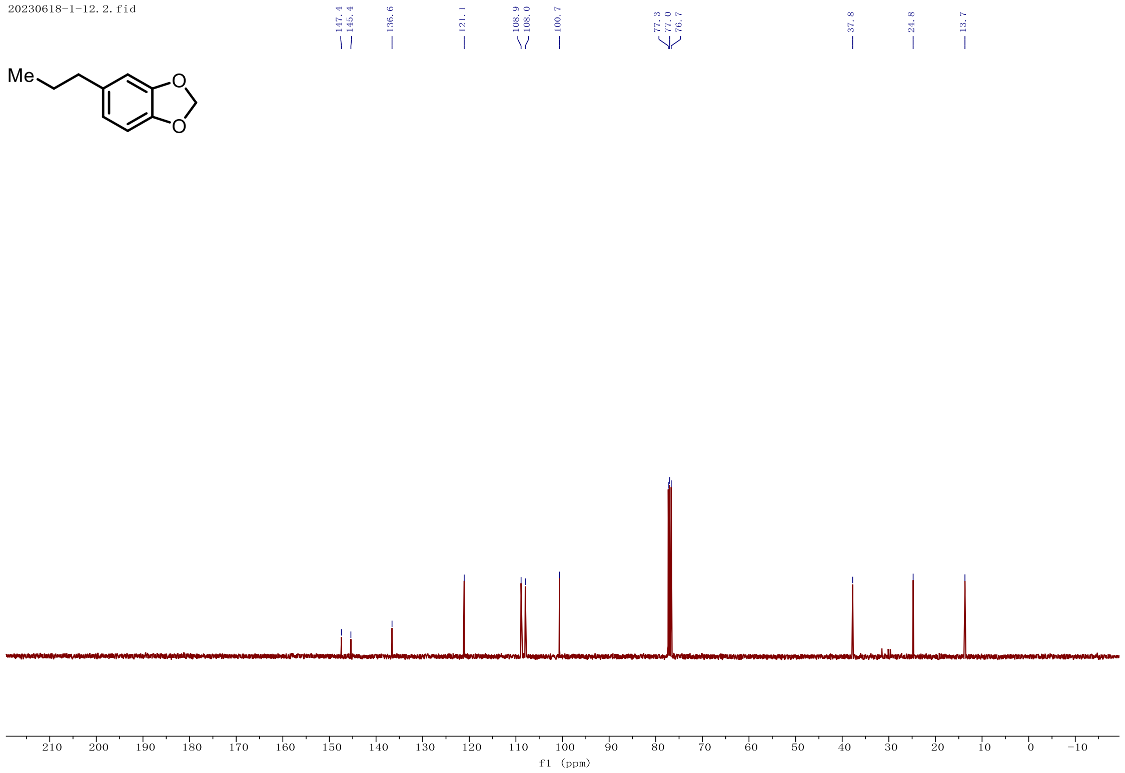

^{1}H NMR of Compound 42 (400 MHz, $CDCl_3$)

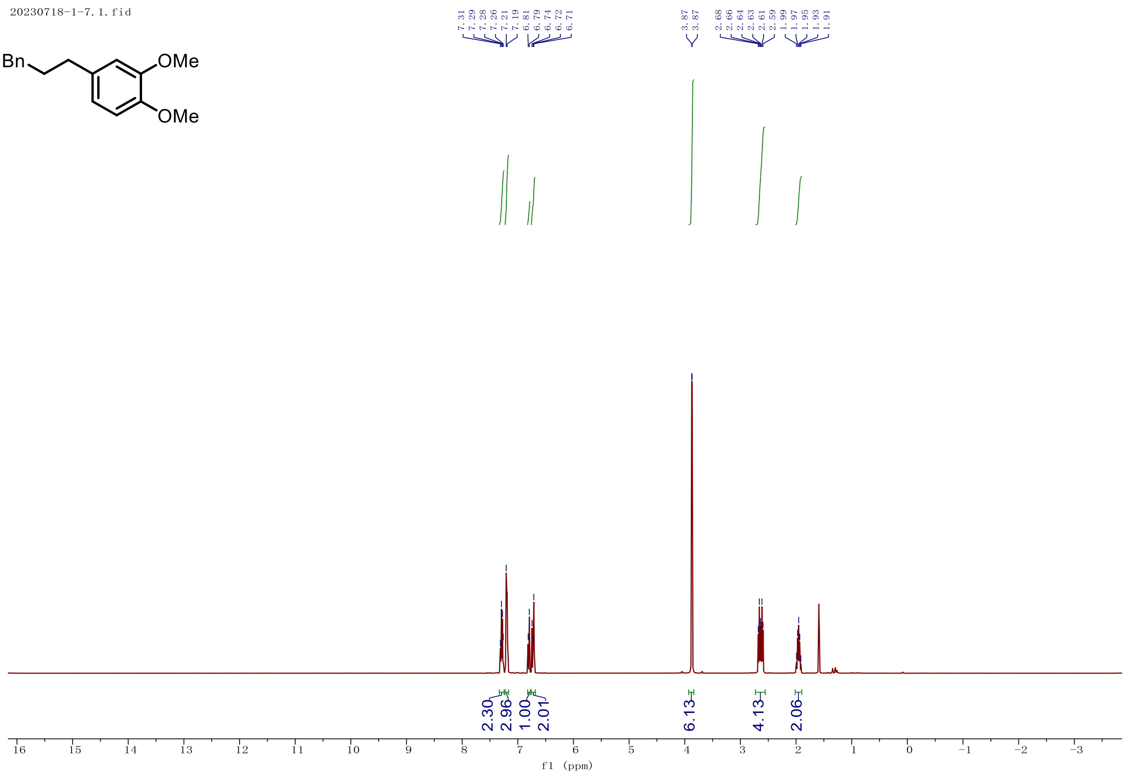

^{13}C NMR of Compound 42 (101 MHz, $CDCl_3$)

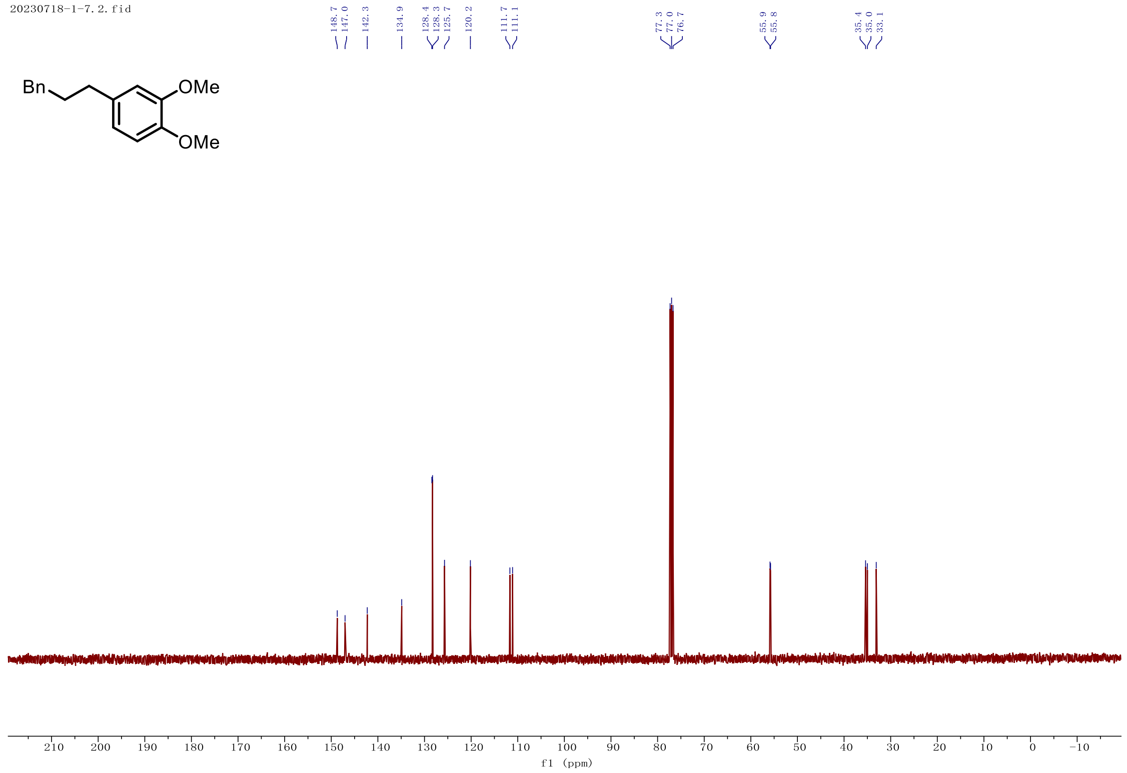

^{1}H NMR of Compound 43 (400 MHz, $CDCl_3$)

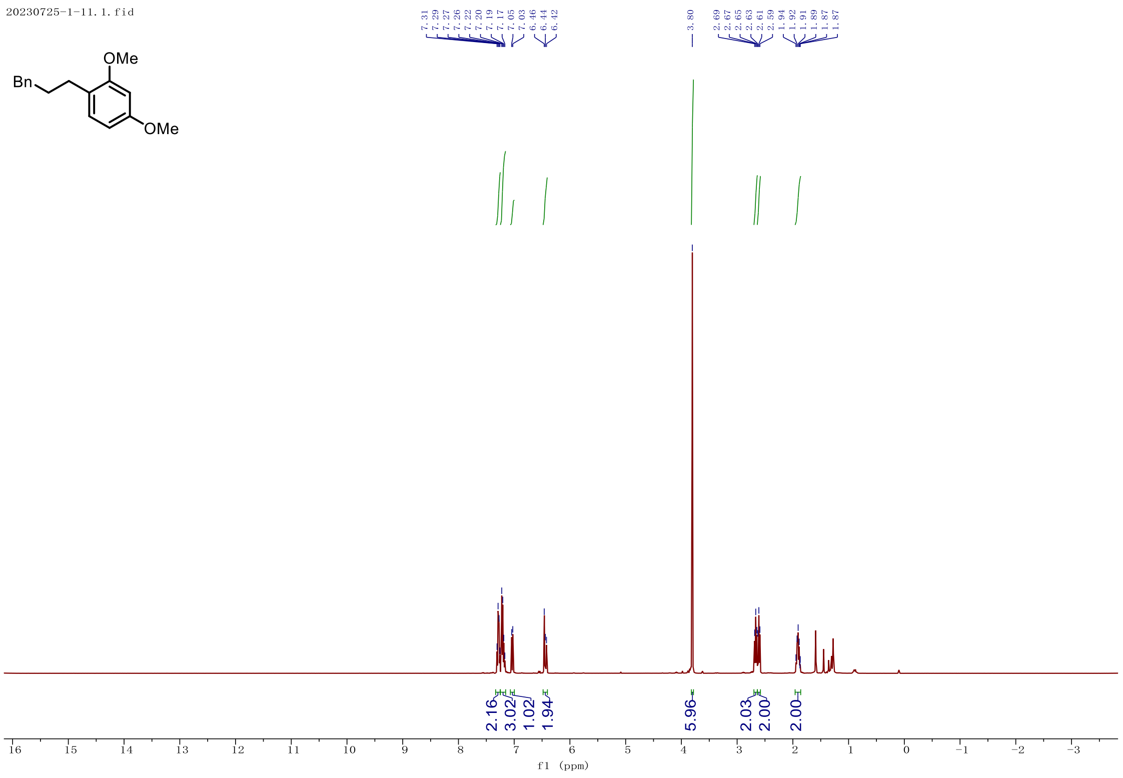

^{13}C NMR of Compound 43 (101 MHz, $CDCl_3$)

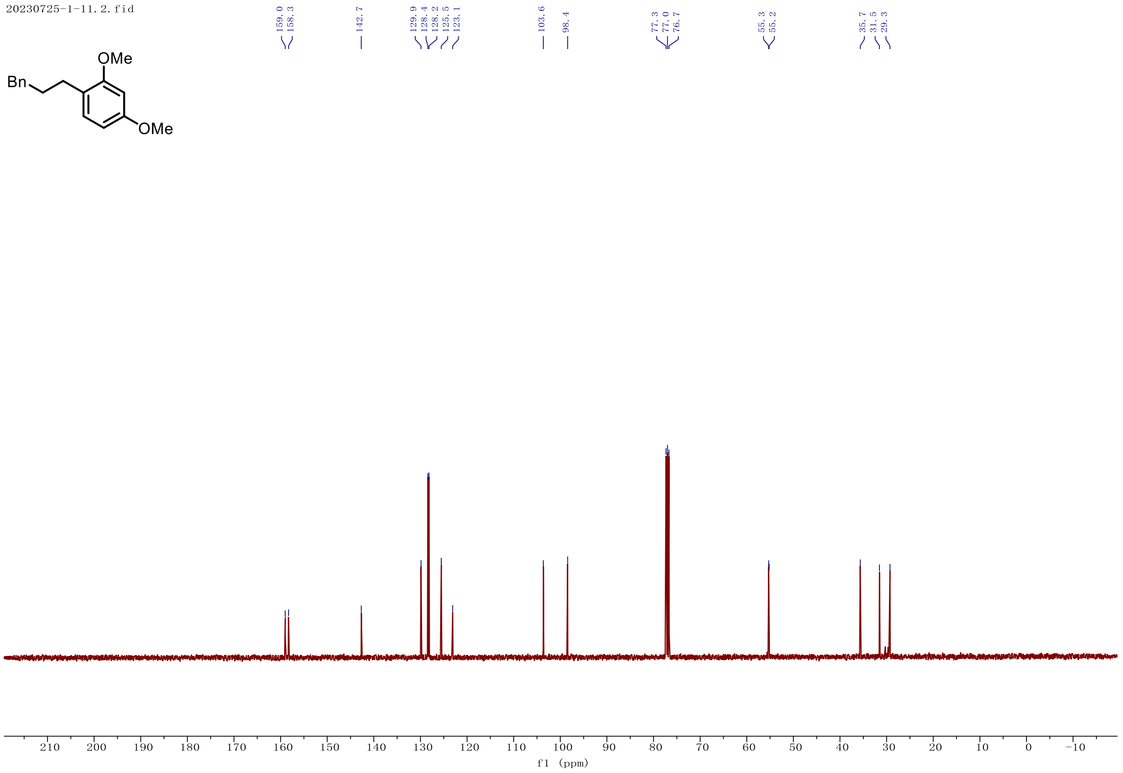

^{1}H NMR of Compound 44 (400 MHz, $CDCl_3$)

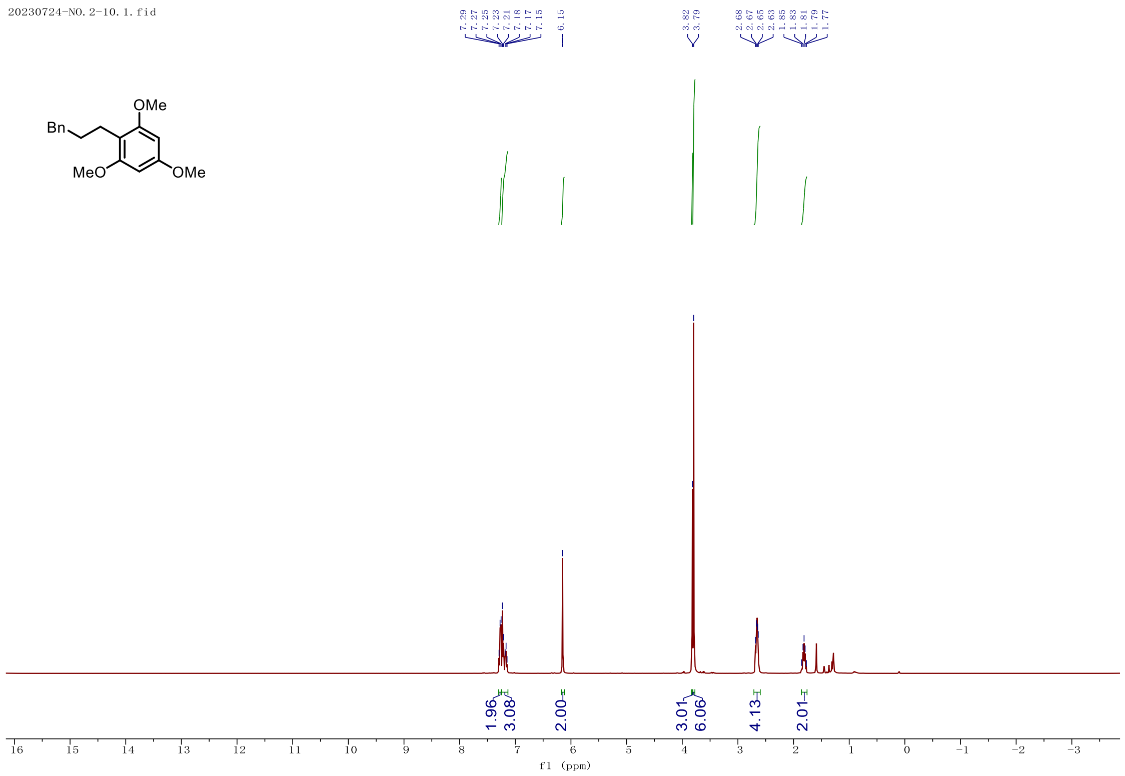

^{13}C NMR of Compound 44 (101 MHz, $CDCl_3$)

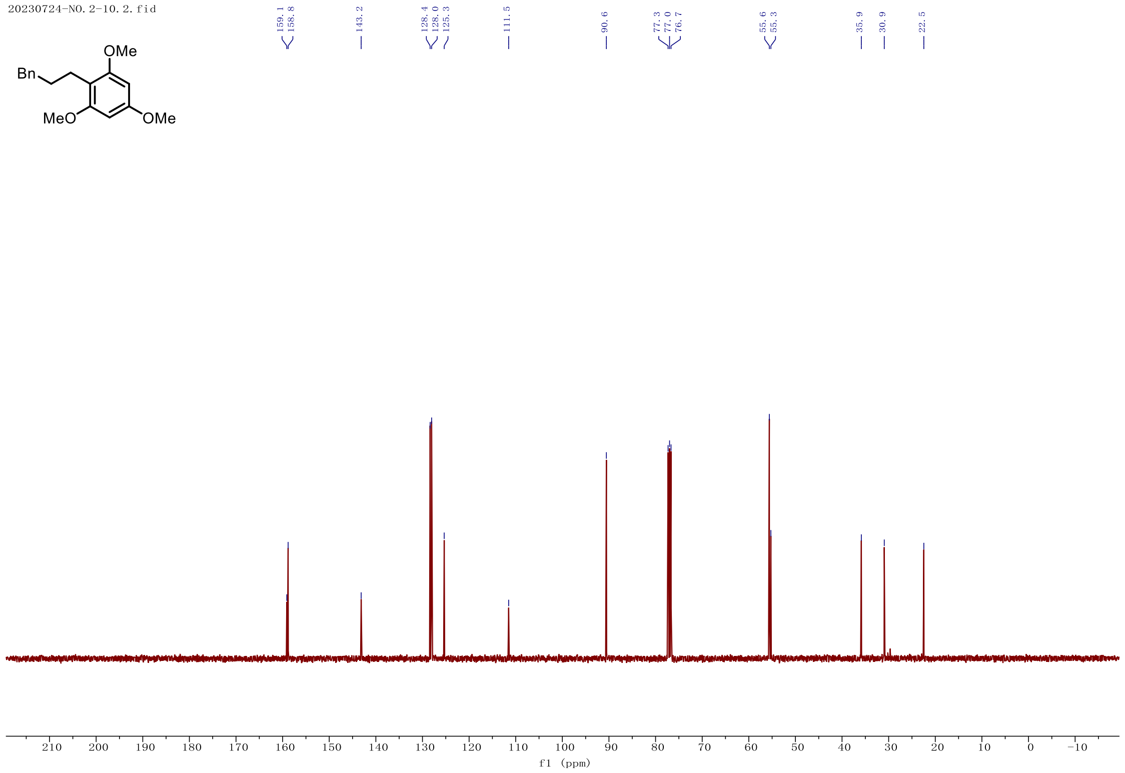

^{1}H NMR of Compound 45 (400 MHz, $CDCl_3$)

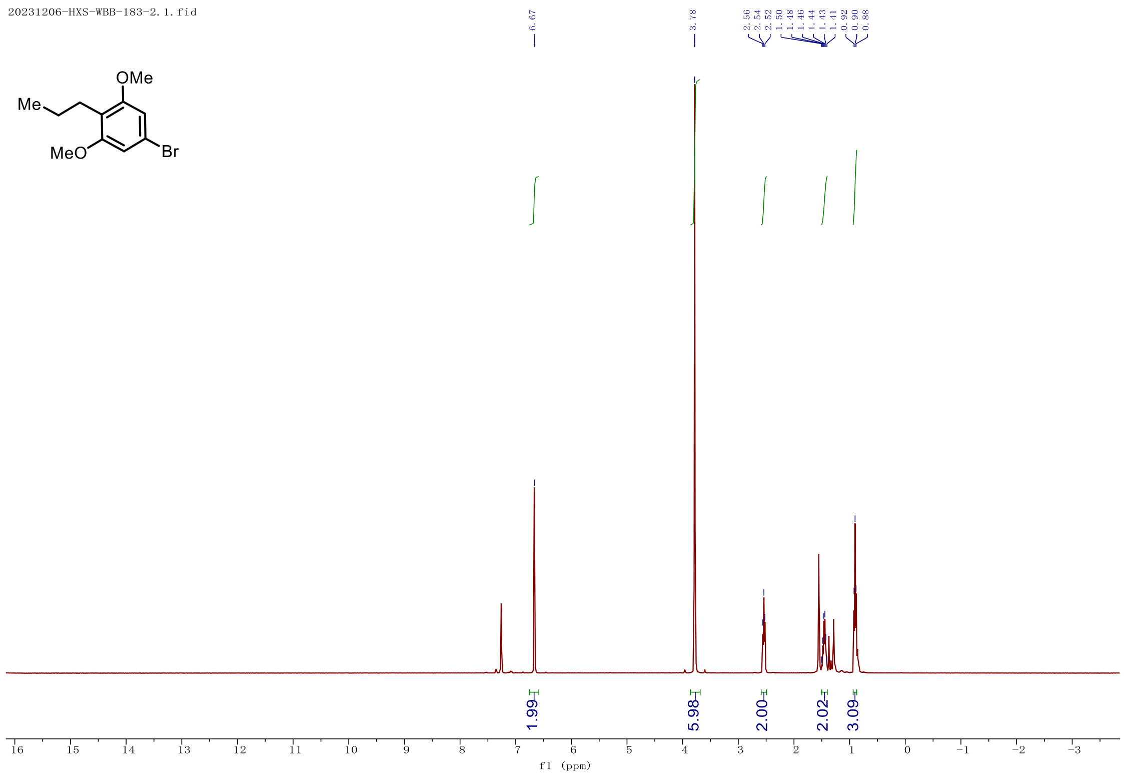

^{13}C NMR of Compound 45 (101 MHz, $CDCl_3$)

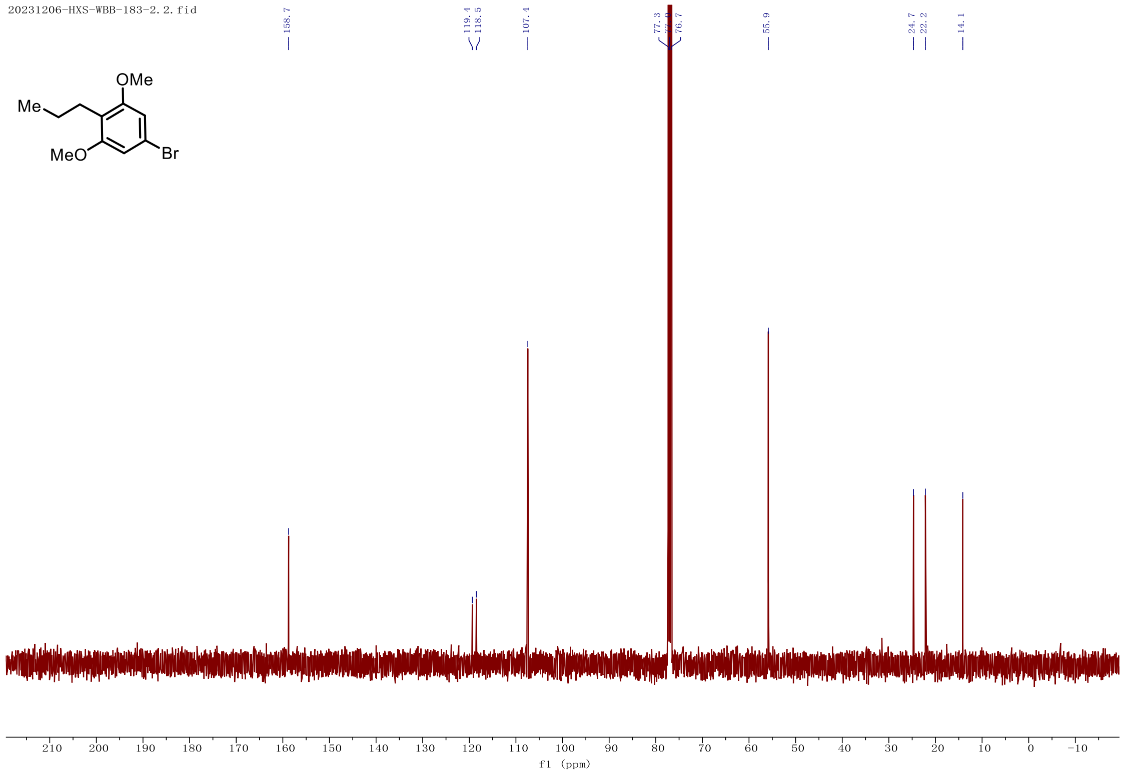

^{1}H NMR of Compound 46 (400 MHz, $CDCl_3$)

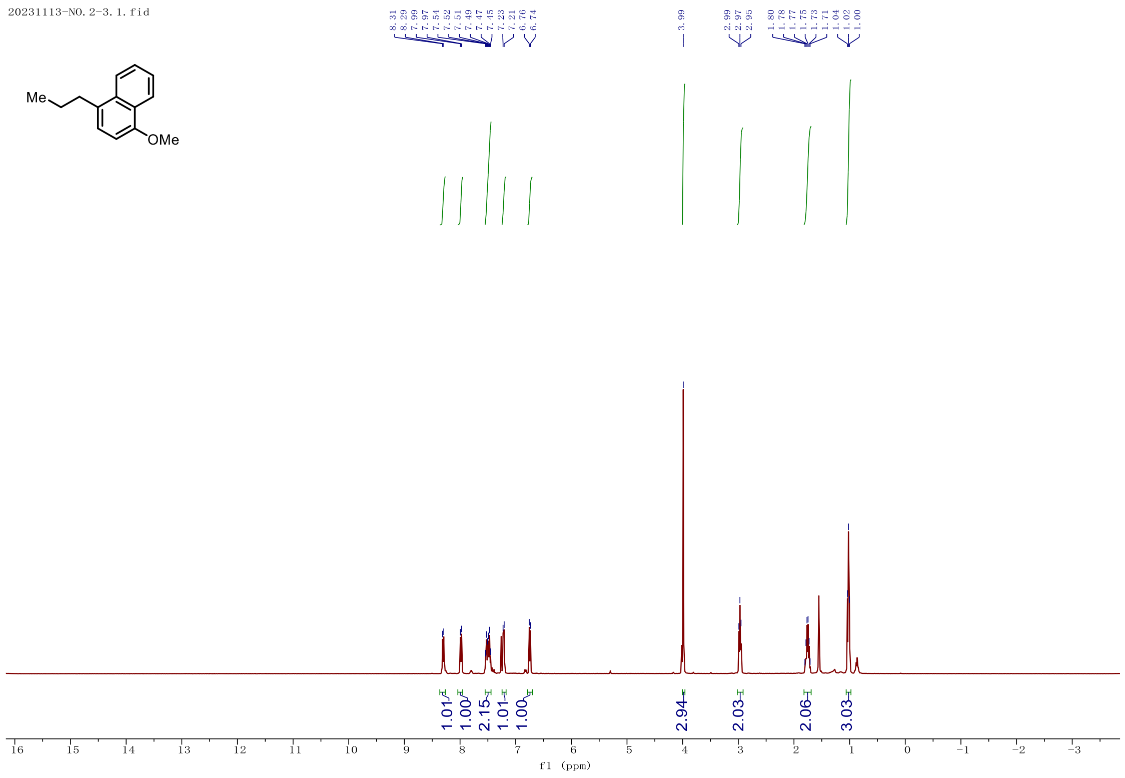

^{13}C NMR of Compound 46 (101 MHz, $CDCl_3$)

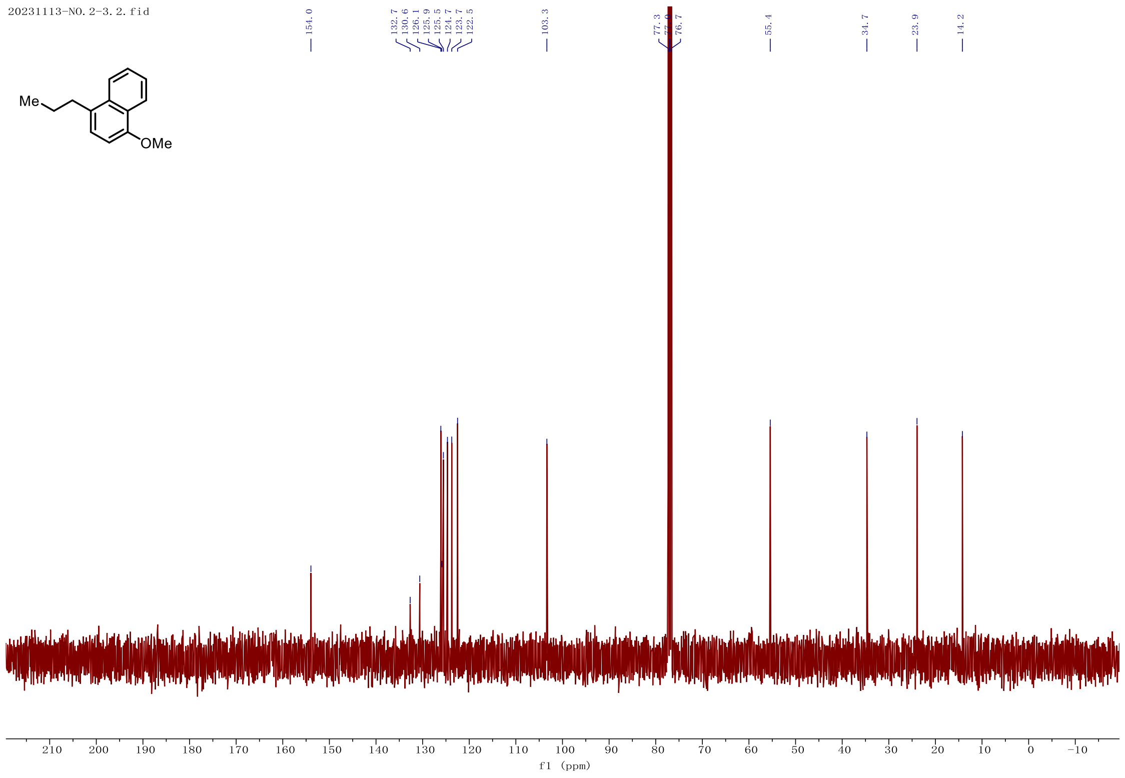

^{1}H NMR of Compound 47 (400 MHz, $CDCl_3$)

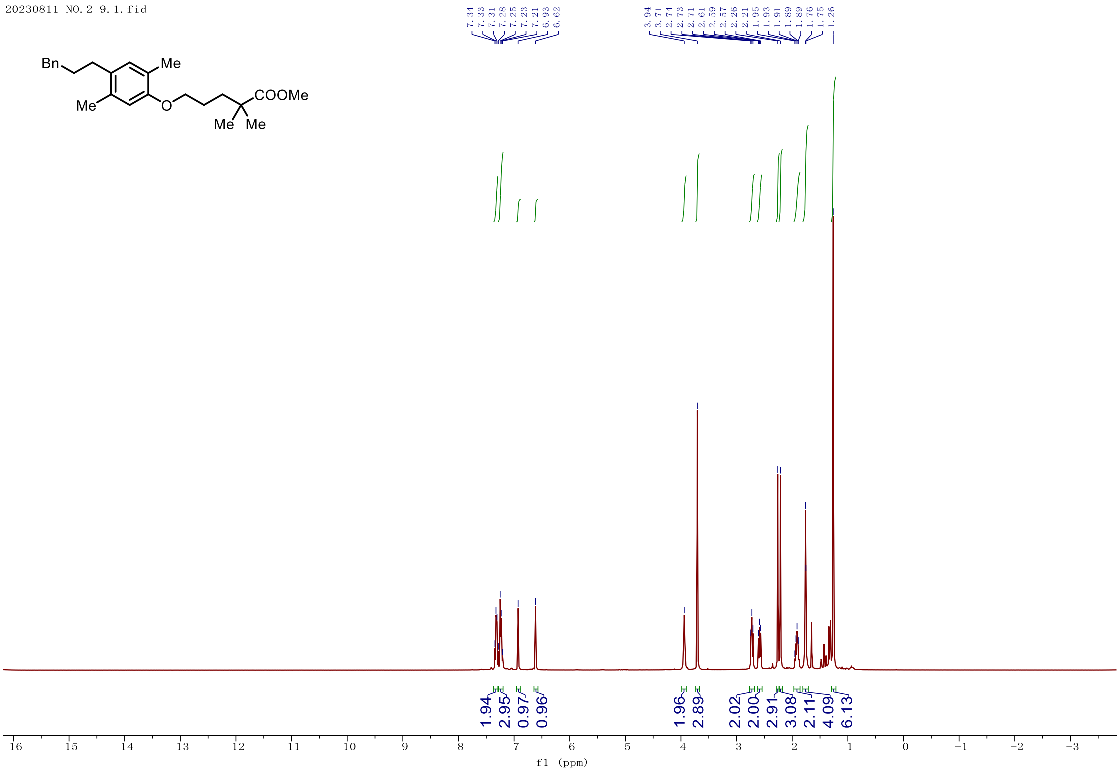

^{13}C NMR of Compound 47 (101 MHz, $CDCl_3$)

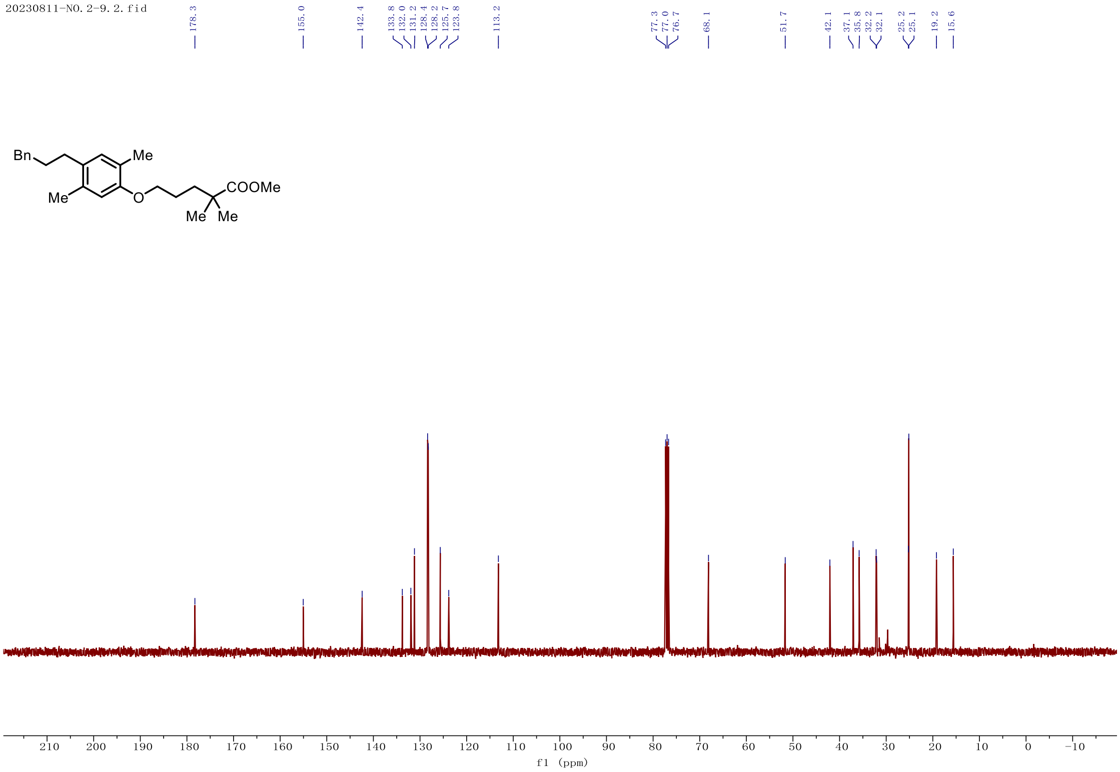

^{1}H NMR of Compound 48 (400 MHz, $CDCl_3$)

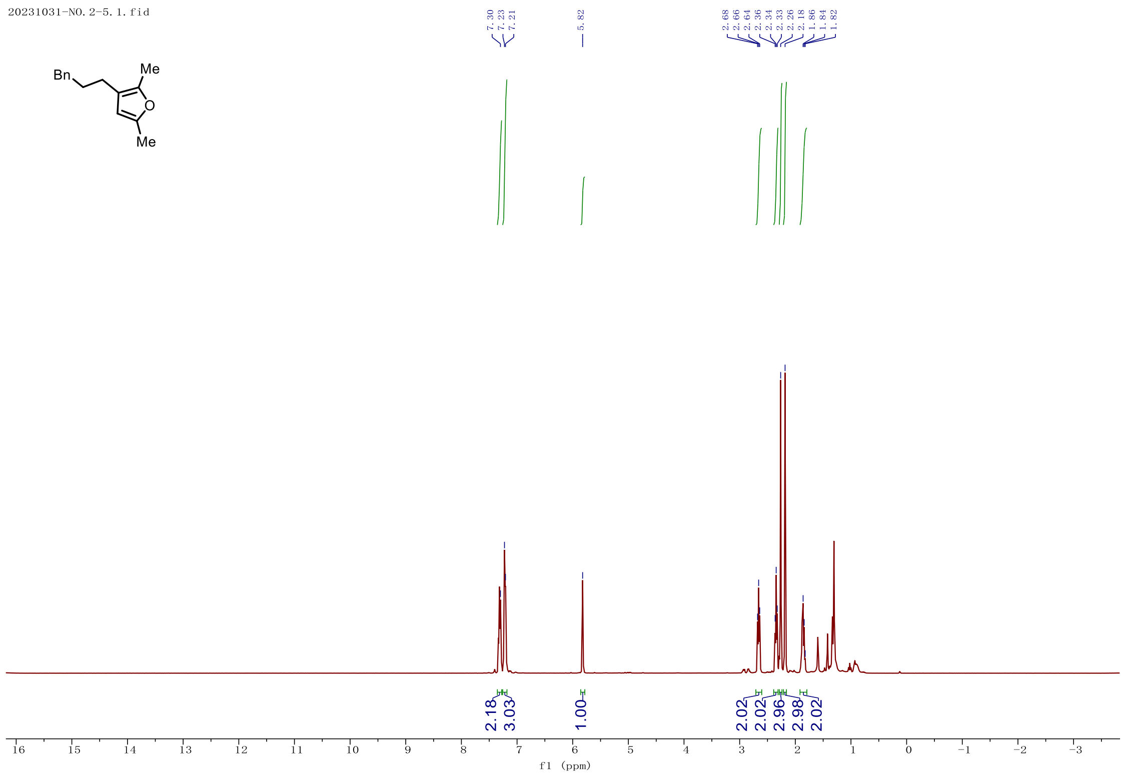

^{13}C NMR of Compound 48 (101 MHz, $CDCl_3$)

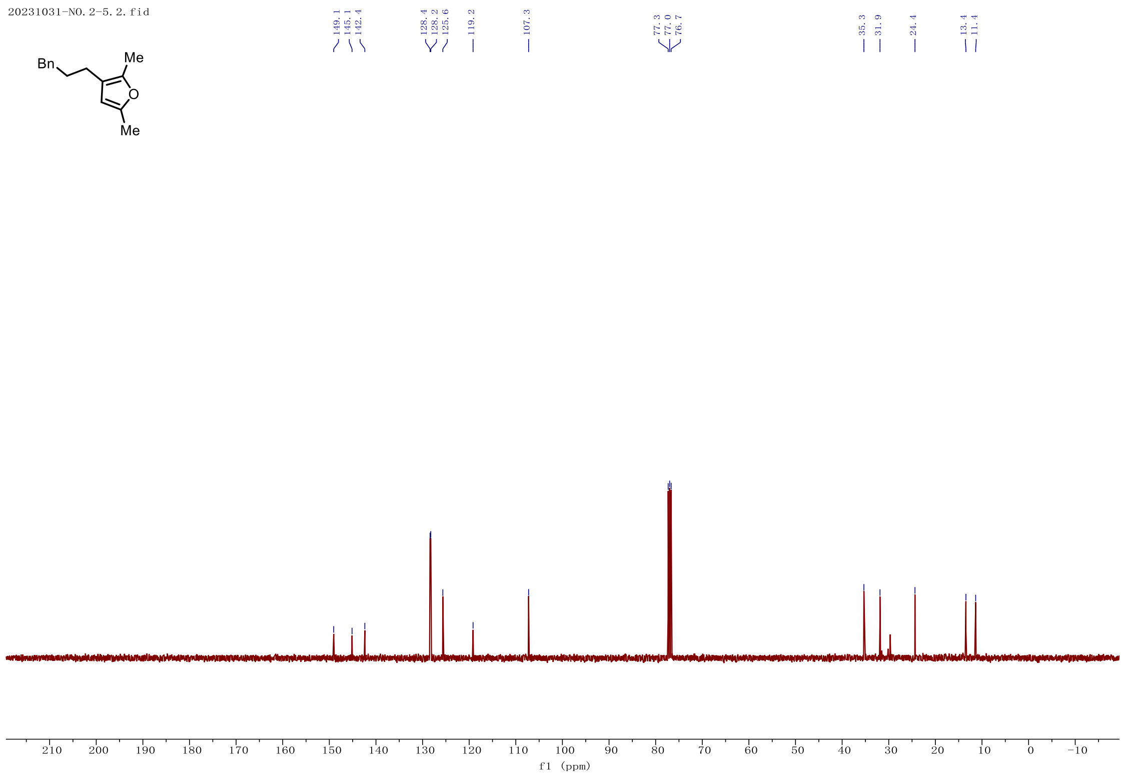

^{1}H NMR of Compound 49 (400 MHz, $CDCl_3$)

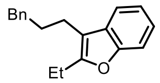

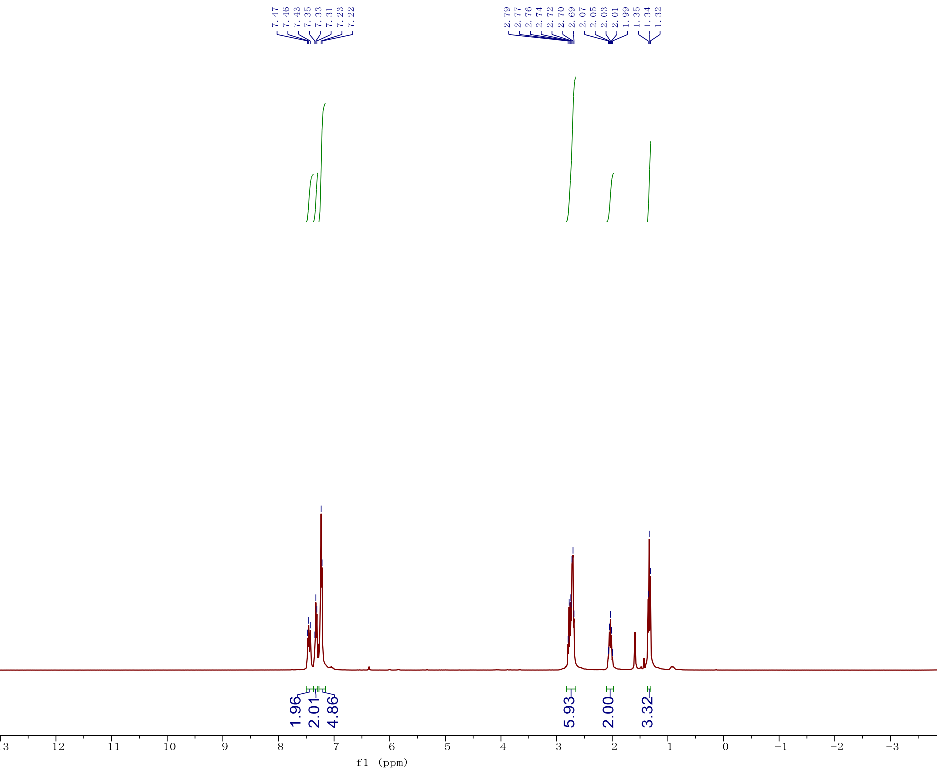

^{13}C NMR of Compound 49 (101 MHz, $CDCl_3$)

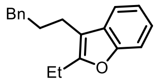

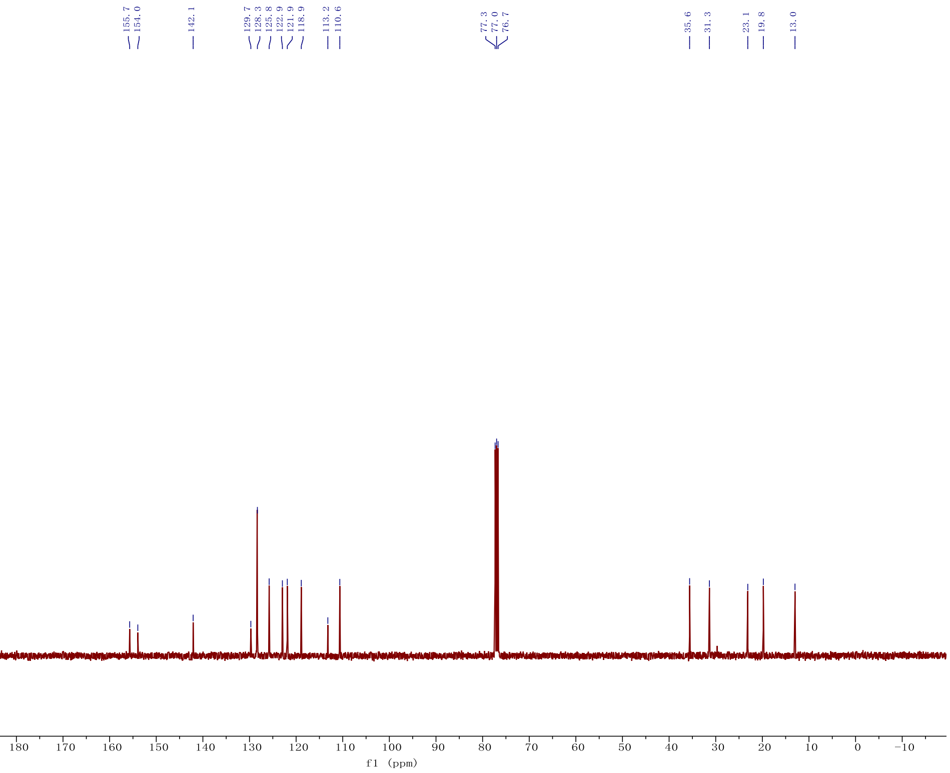

^{1}H NMR of Compound 50 (400 MHz, $CDCl_3$)

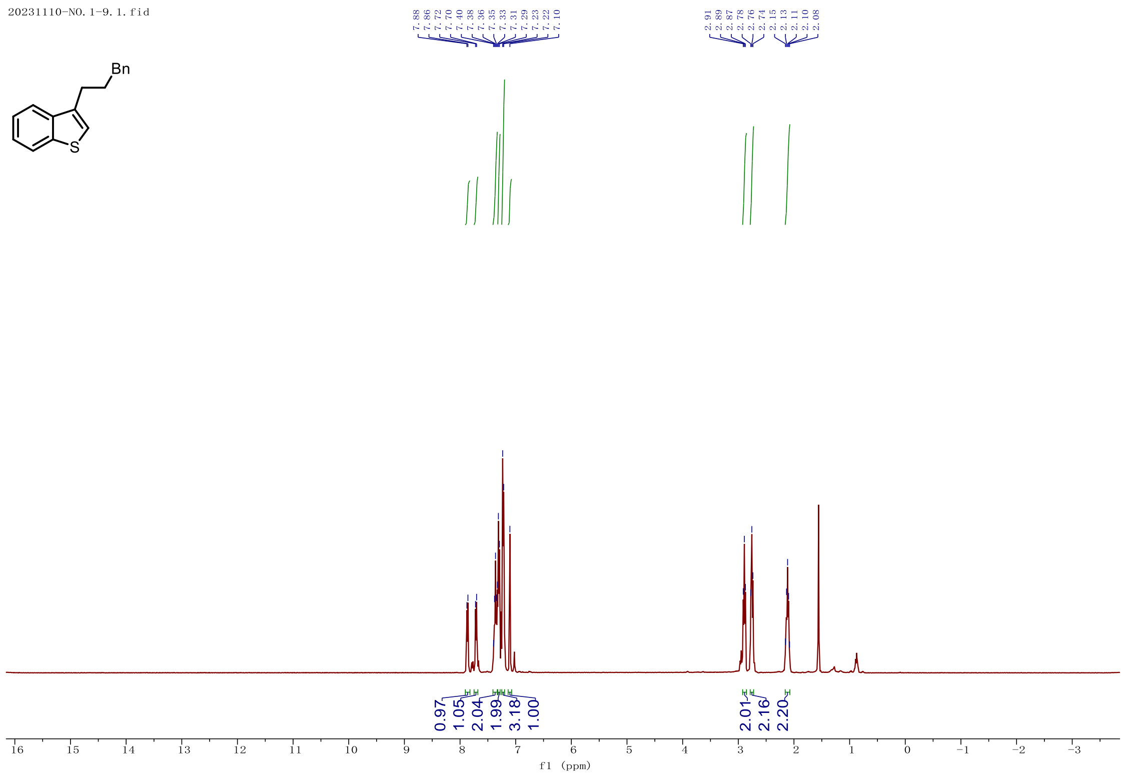

^{13}C NMR of Compound 50 (101 MHz, $CDCl_3$)

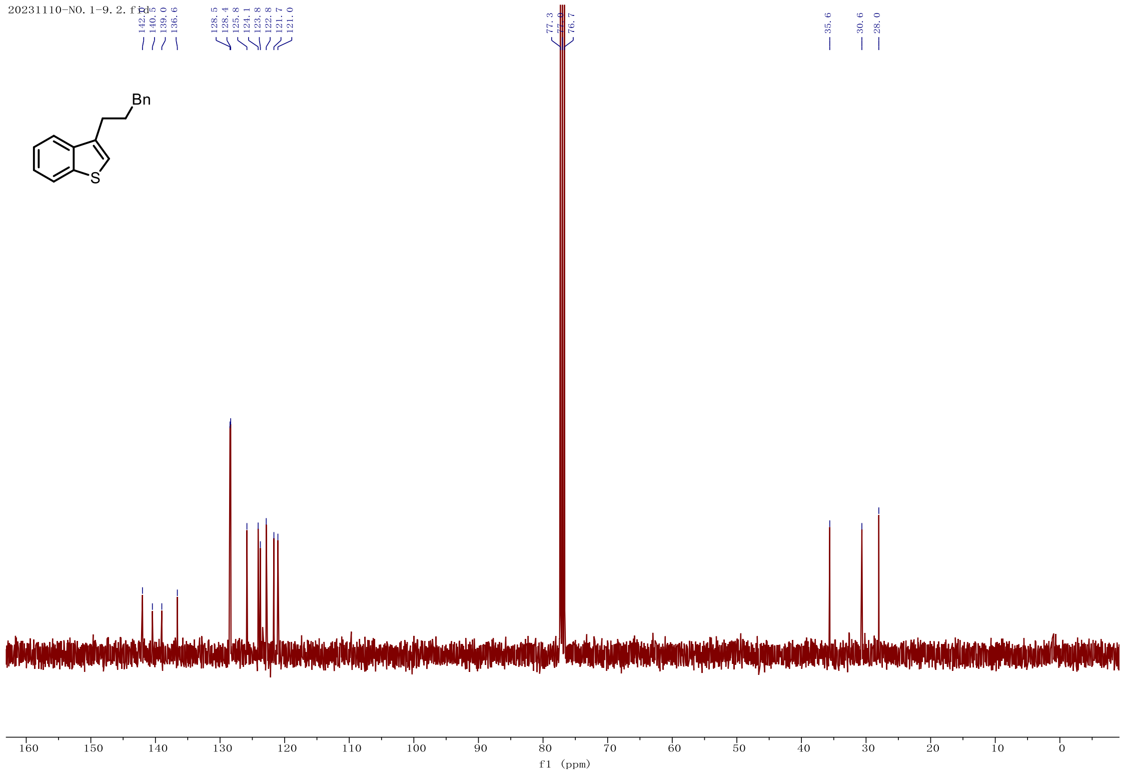

^{1}H NMR of Compound 51 (400 MHz, $CDCl_3$)

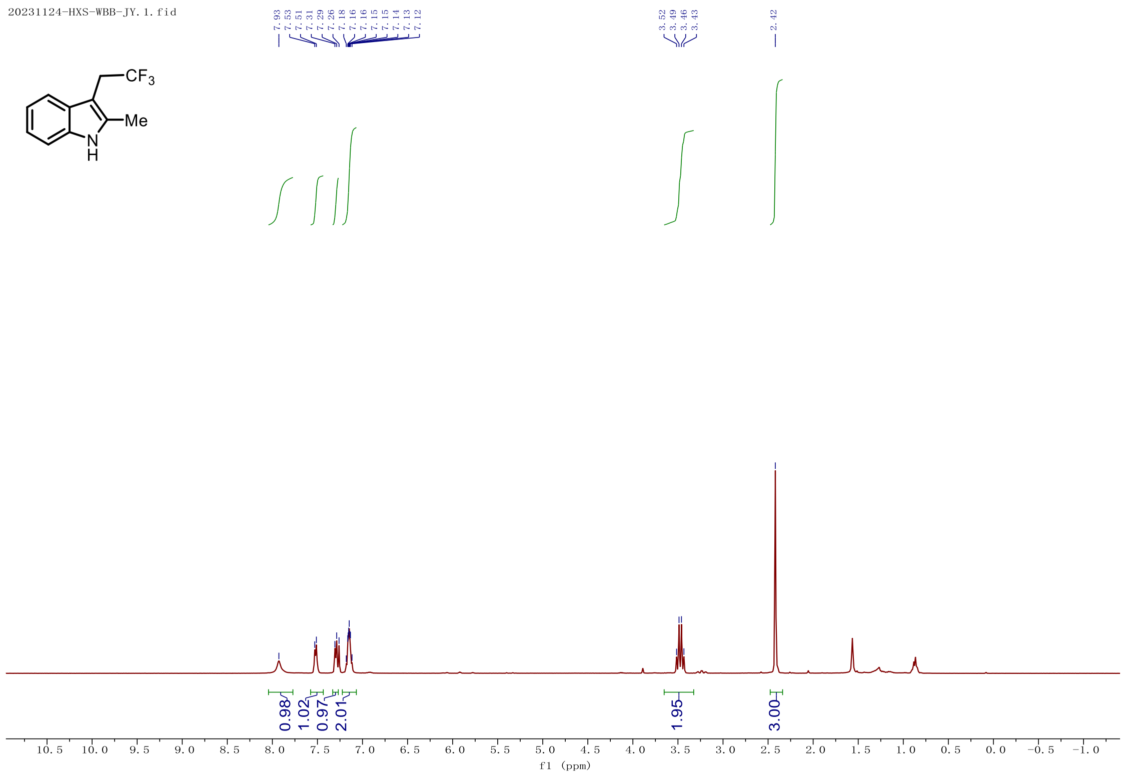

^{13}C NMR of Compound 51 (101 MHz, $CDCl_3$)

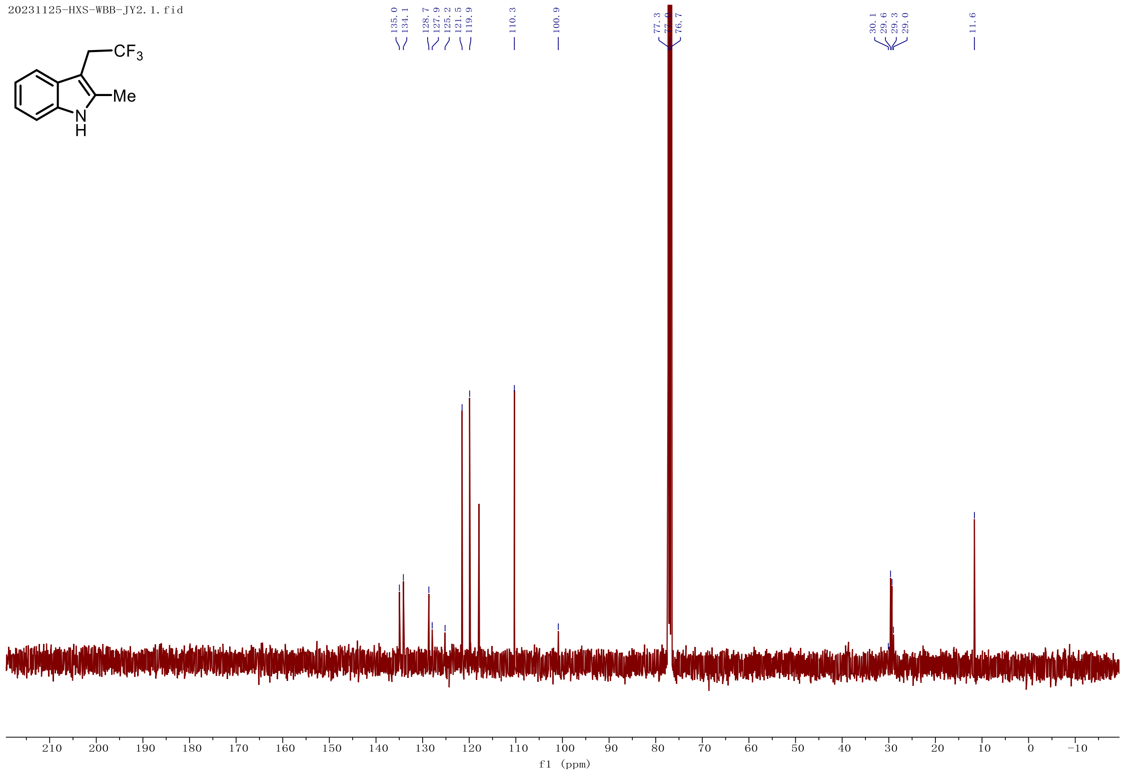

^{19}F NMR of Compound 51 (376 MHz, $CDCl_3$)

20231125-HXS-WBB-JY3.1.fid

-66.00

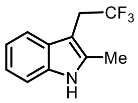

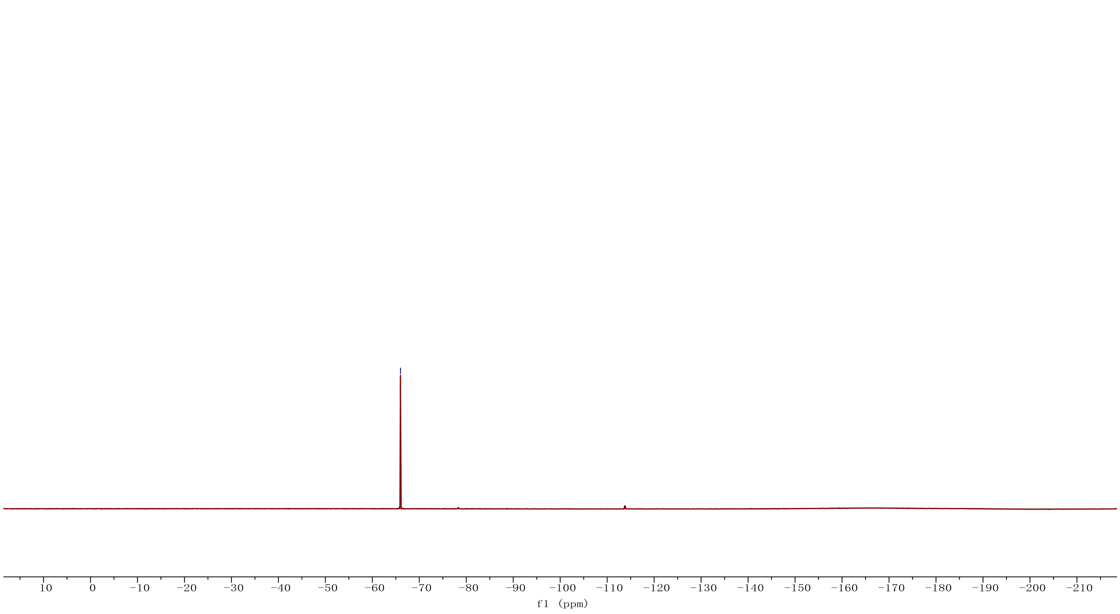

^{1}H NMR of Compound 52 (400 MHz, $CDCl_3$)

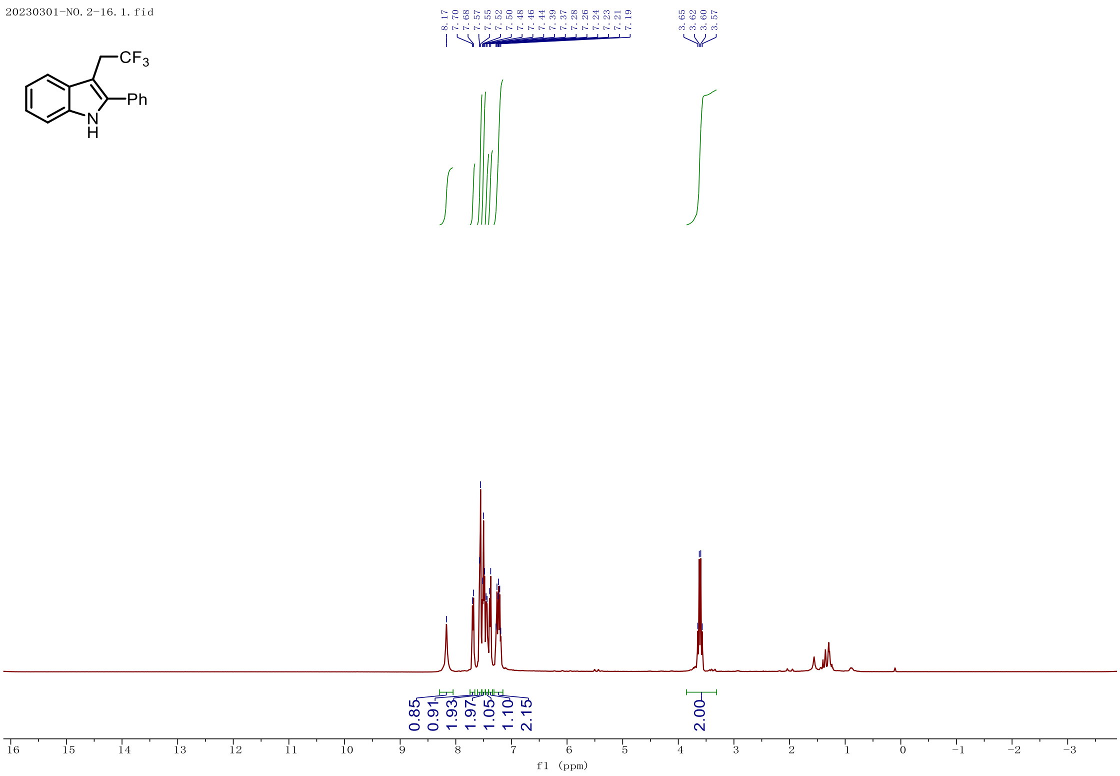

^{13}C NMR of Compound 52 (101 MHz, $CDCl_3$)

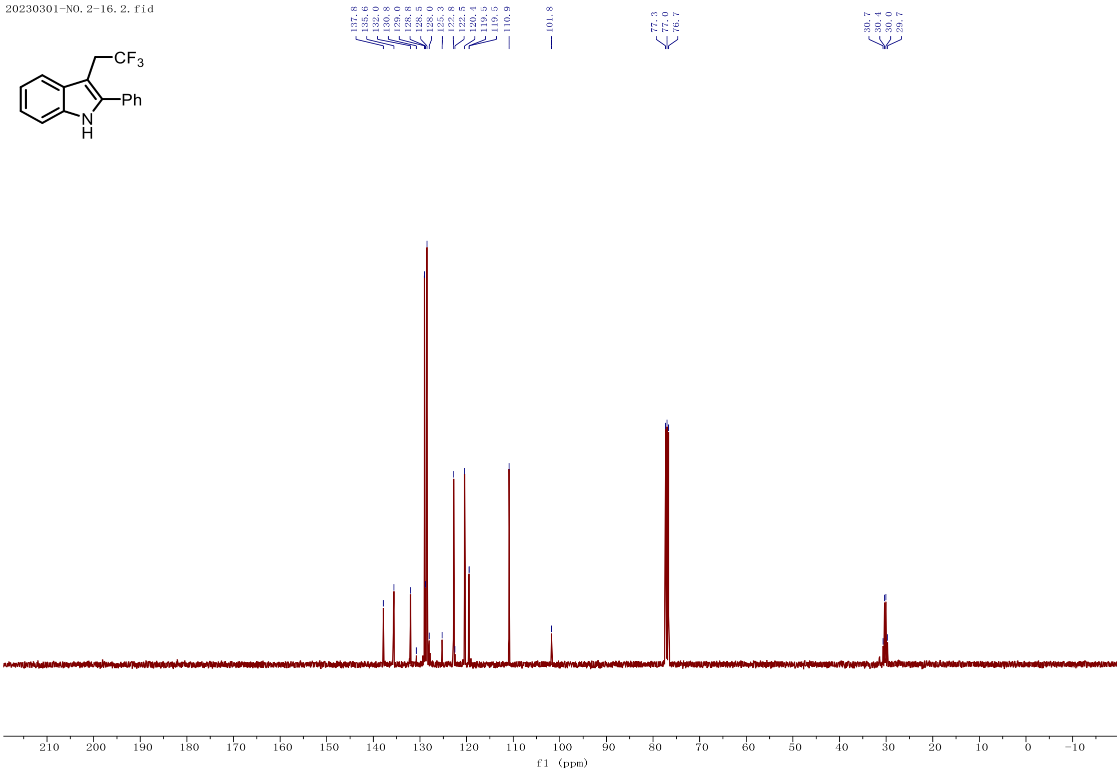

^{19}F NMR of Compound 52 (376 MHz, $CDCl_3$)

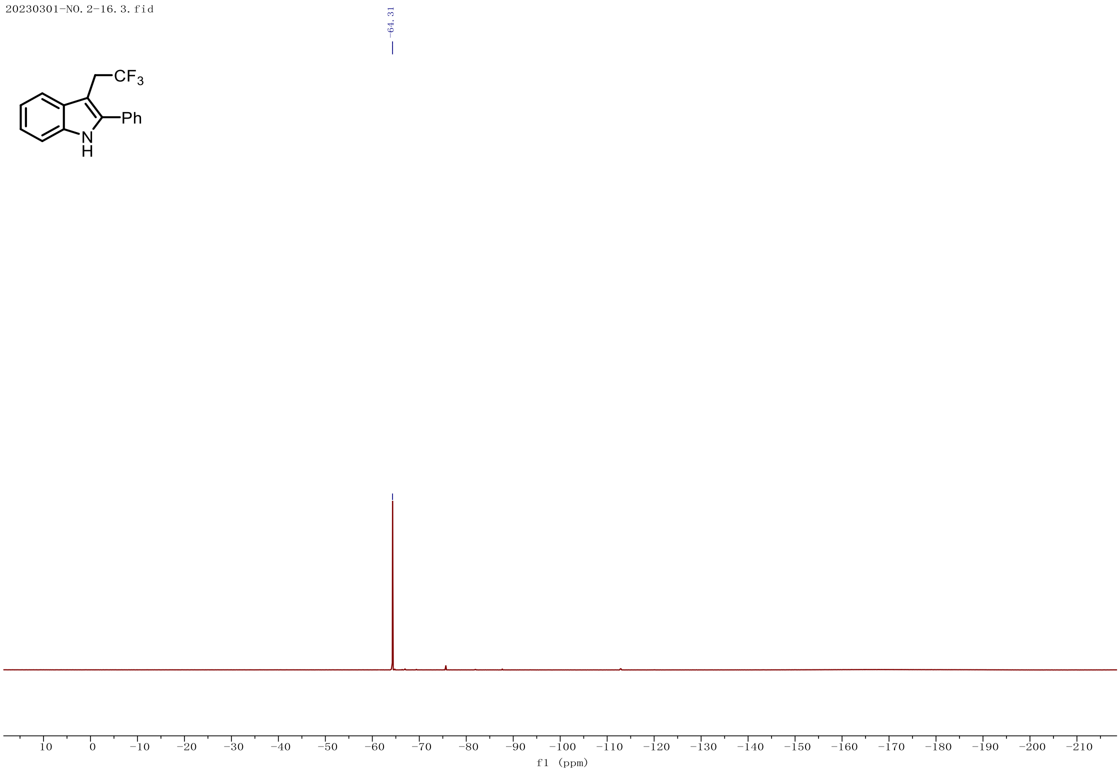

^{1}H NMR of Compound 53 (400 MHz, $CDCl_3$)

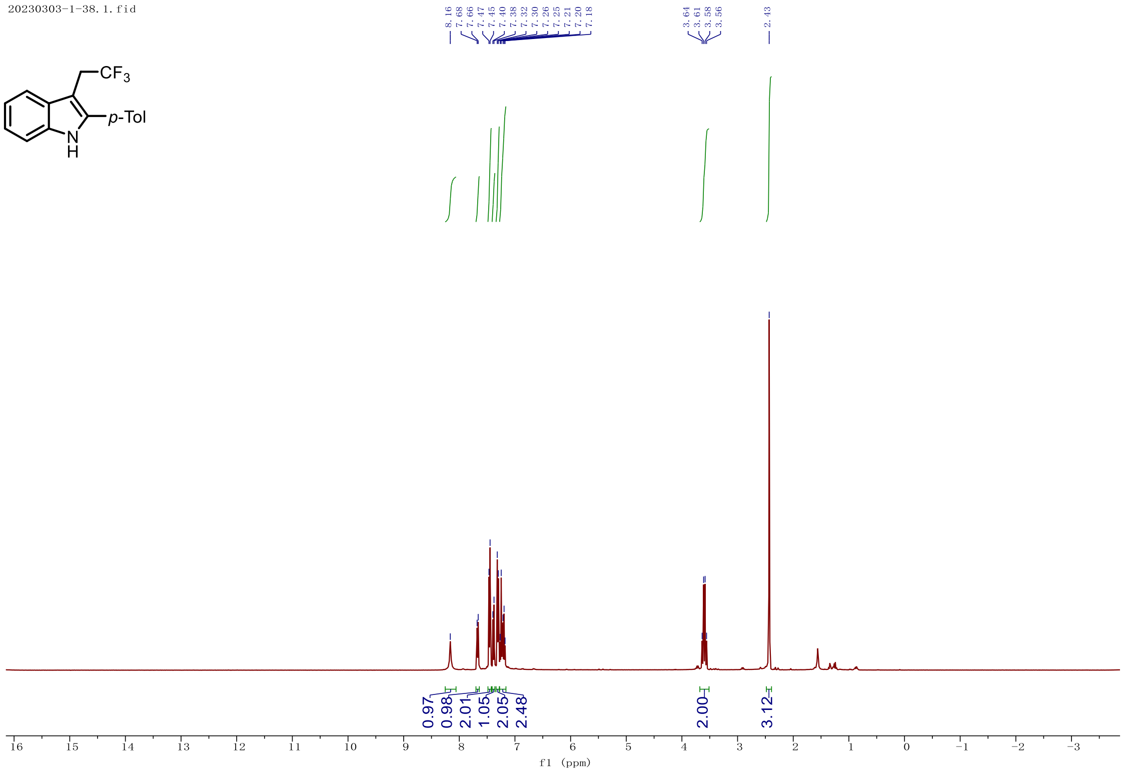

^{13}C NMR of Compound 53 (101 MHz, $CDCl_3$)

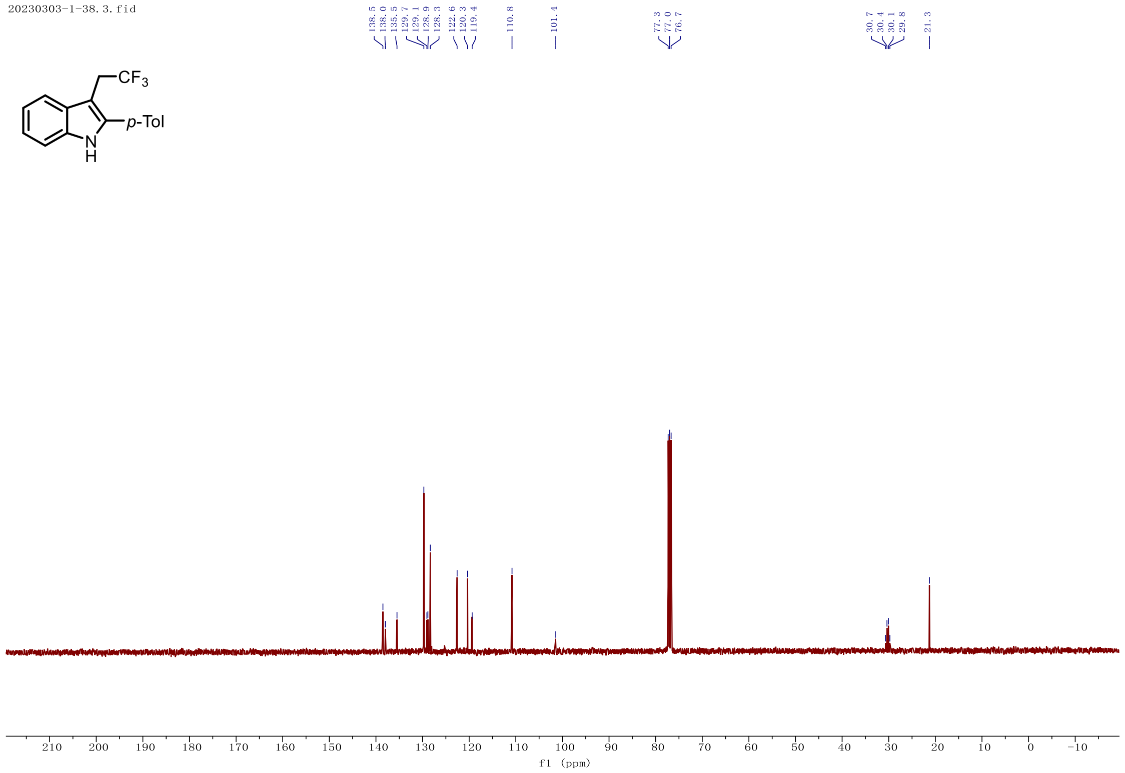

^{19}F NMR of Compound 53 (376 MHz, $CDCl_3$)

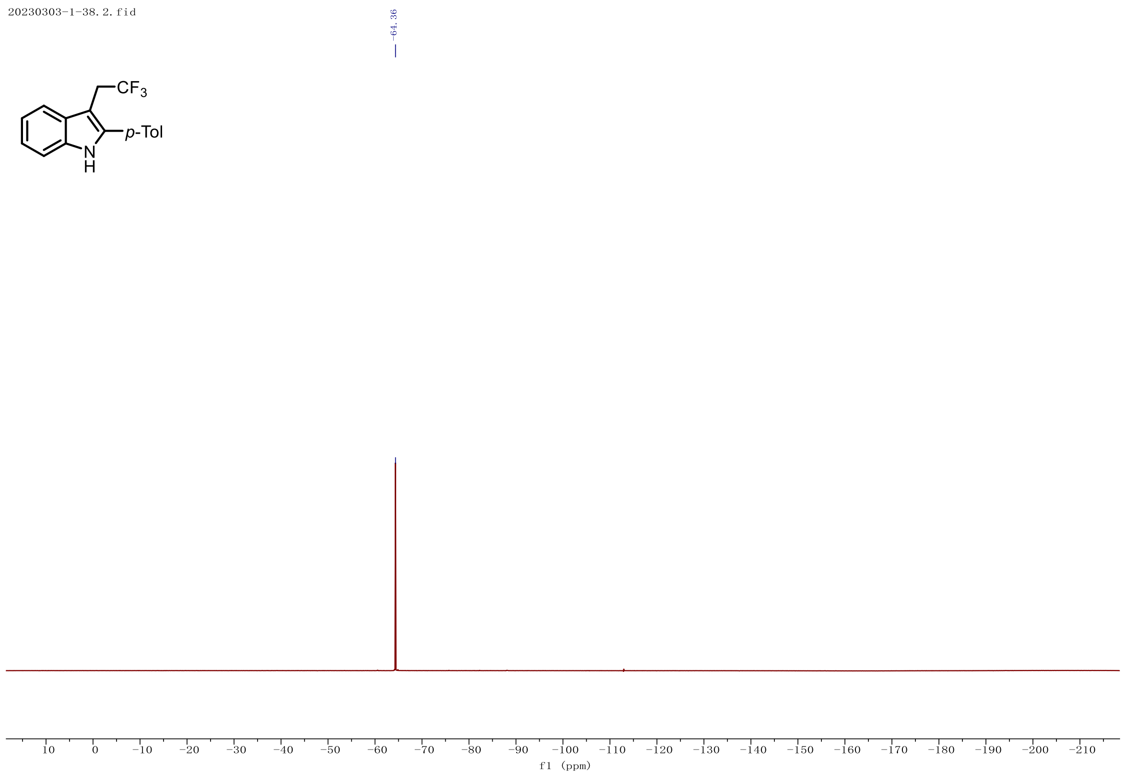

^{1}H NMR of Compound 54 (400 MHz, $CDCl_3$)

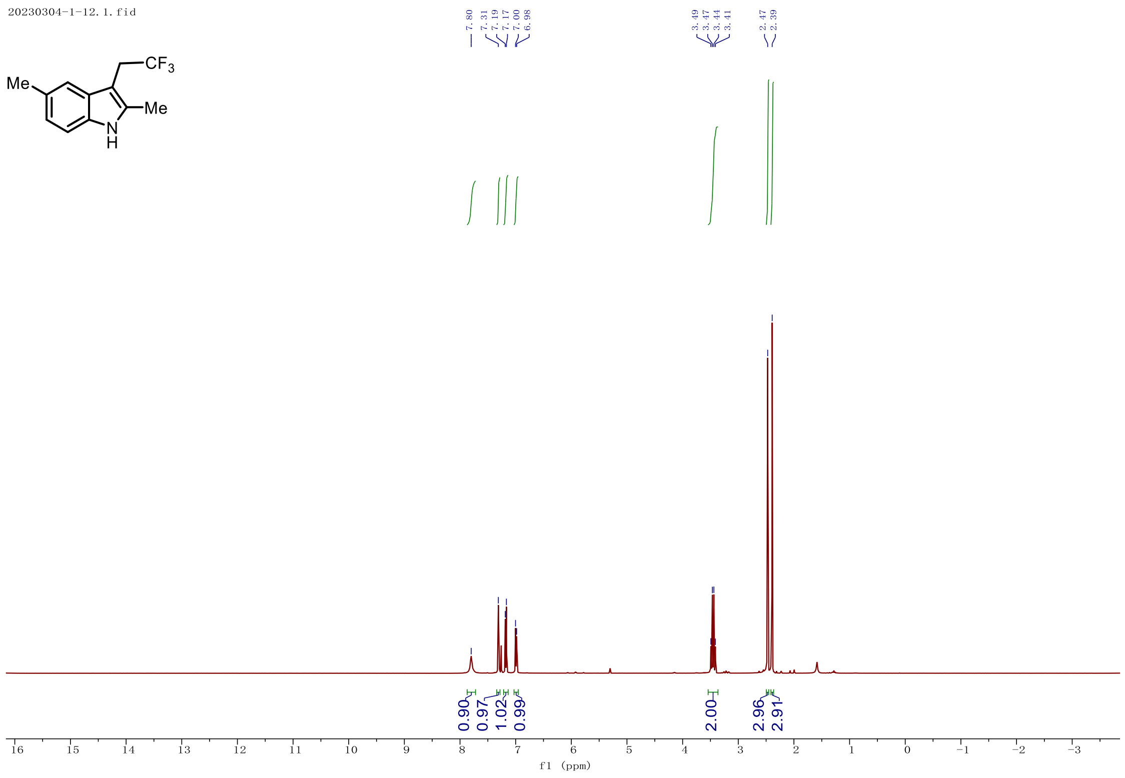

^{13}C NMR of Compound 54 (101 MHz, $CDCl_3$)

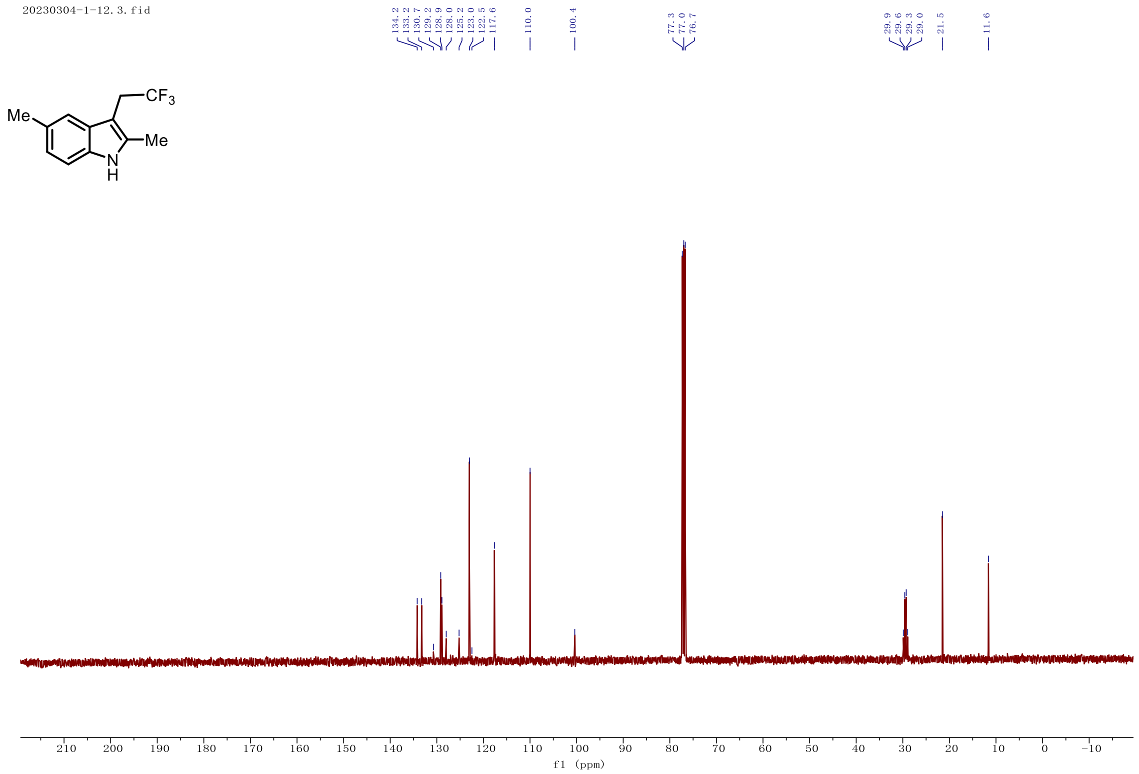

^{19}F NMR of Compound 54 (376 MHz, $CDCl_3$)

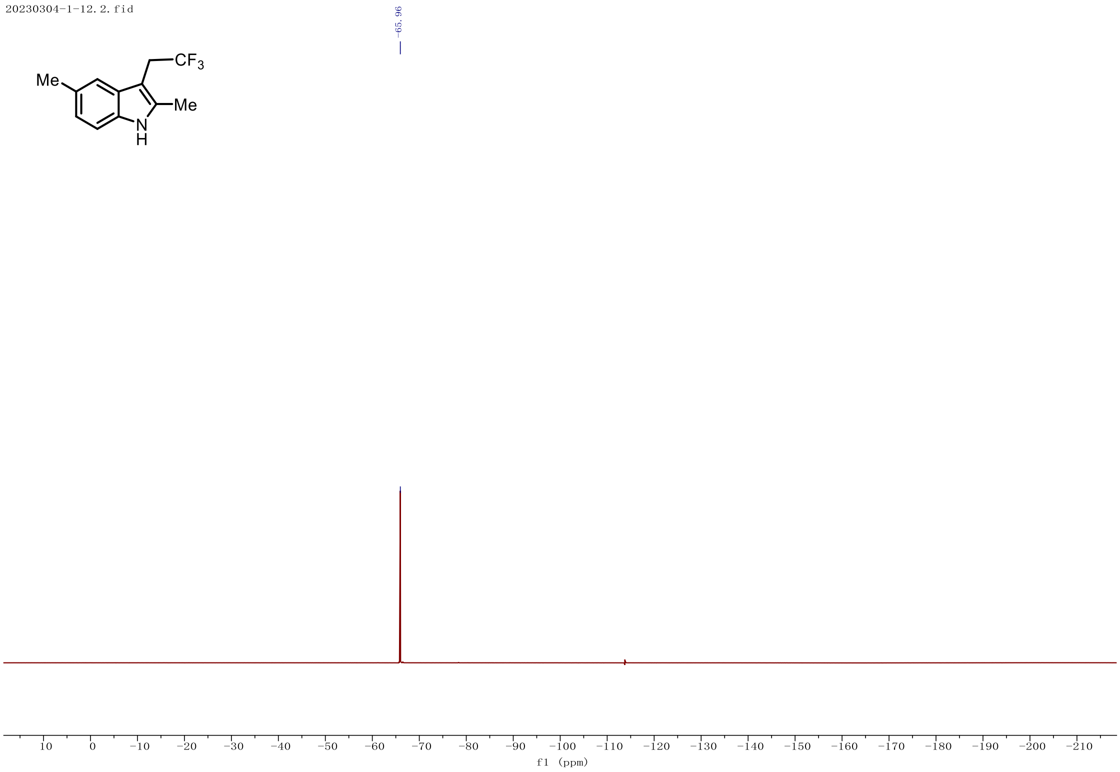

^{1}H NMR of Compound 55 (400 MHz, $CDCl_3$)

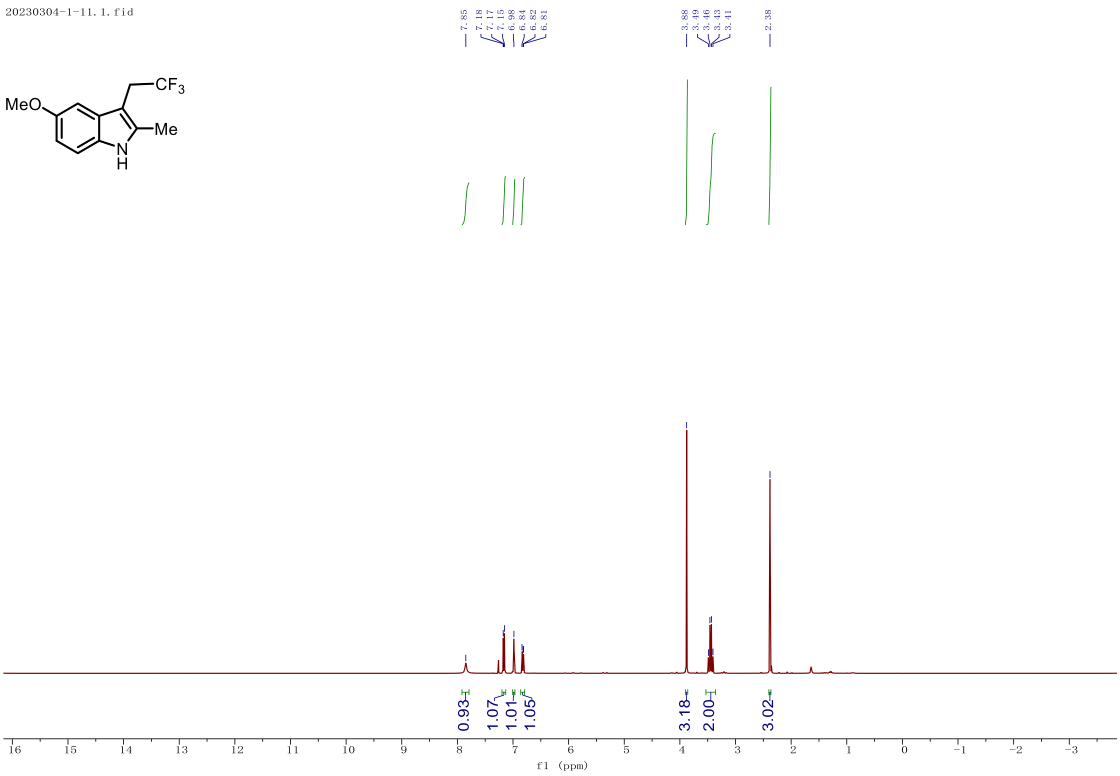

^{13}C NMR of Compound 55 (101 MHz, $CDCl_3$)

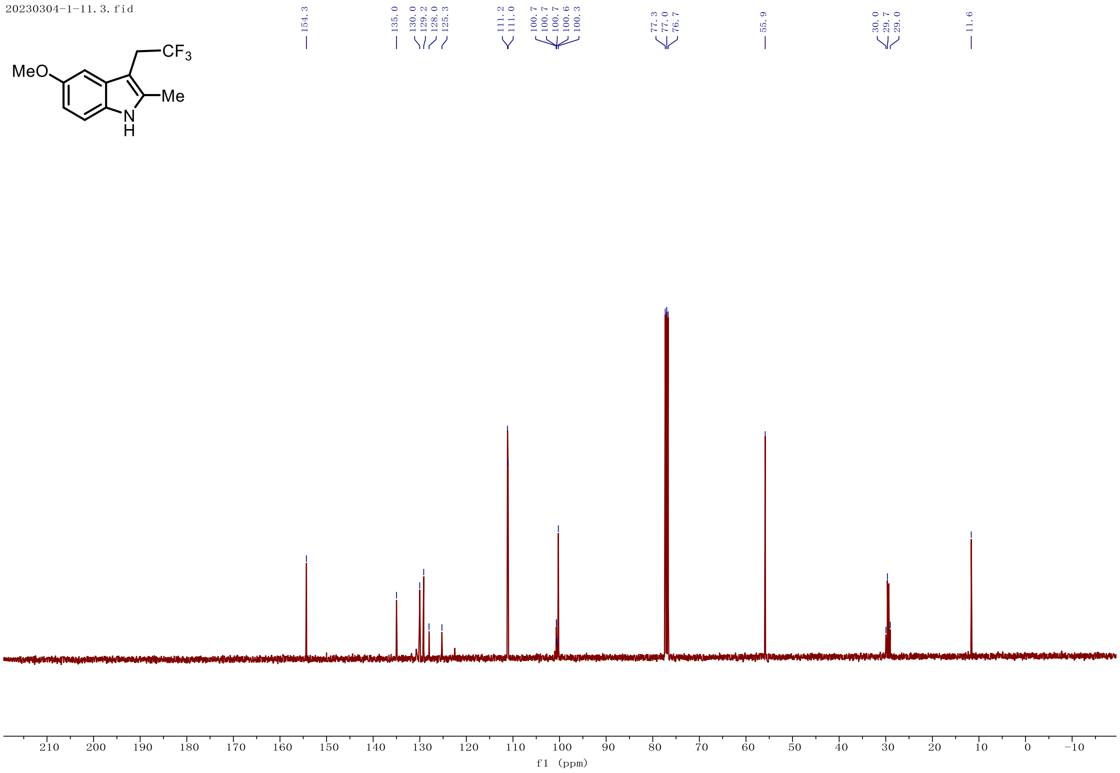

^{19}F NMR of Compound 55 (376 MHz, $CDCl_3$)

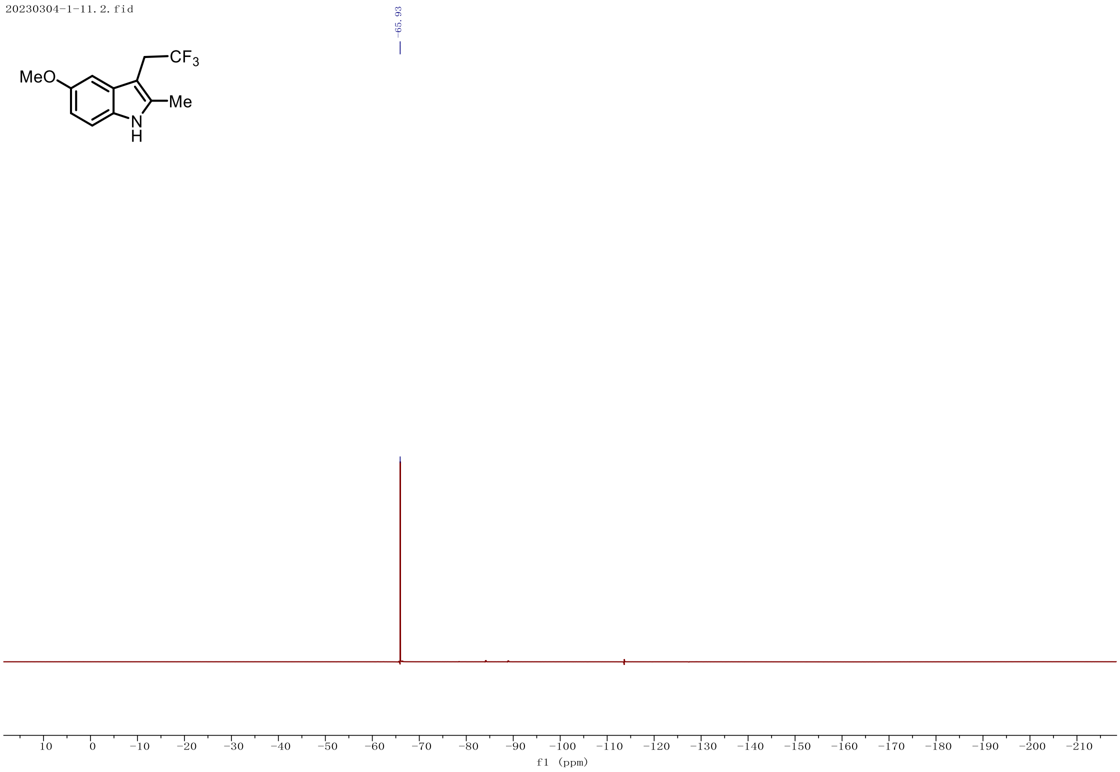

^{1}H NMR of Compound 56 (400 MHz, $CDCl_3$)

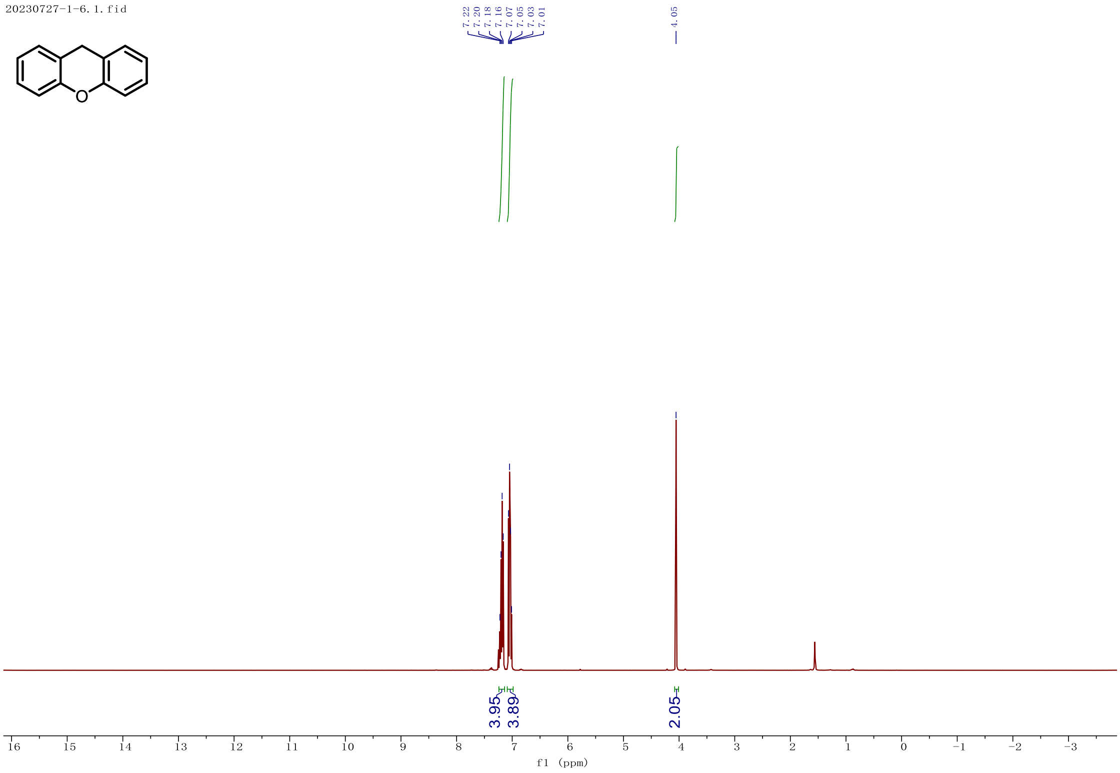

^{13}C NMR of Compound 56 (101 MHz, $CDCl_3$)

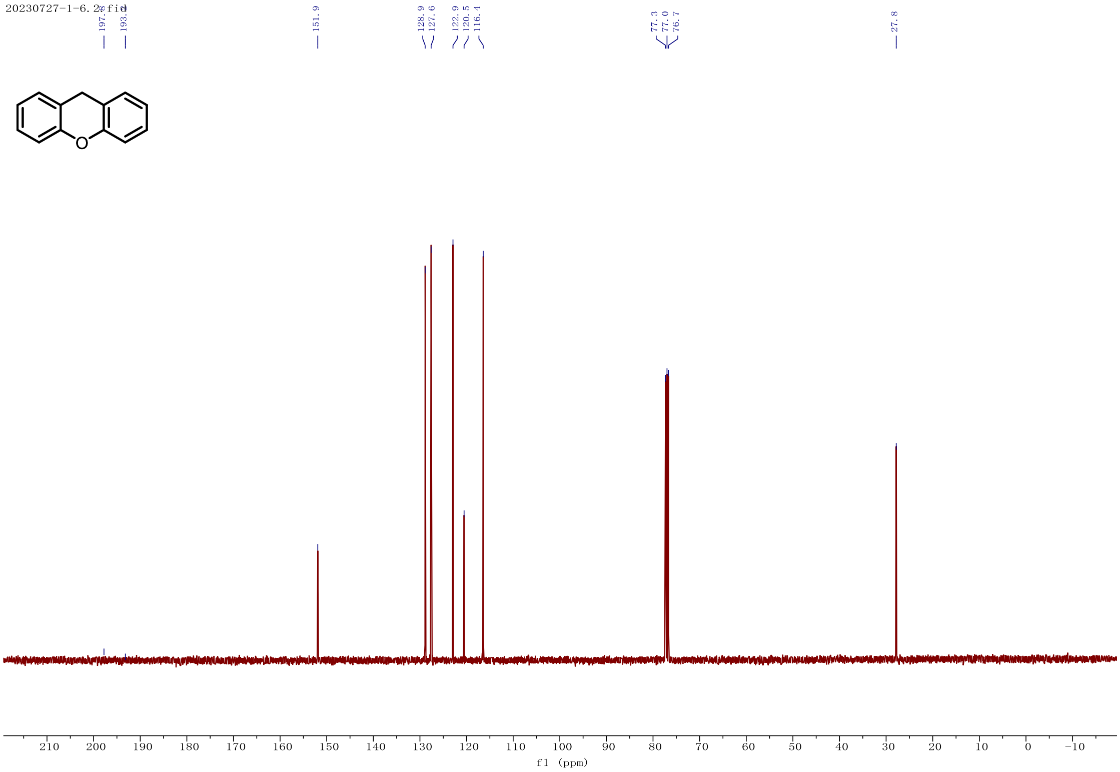

^{1}H NMR of Compound 57 (400 MHz, $CDCl_3$)

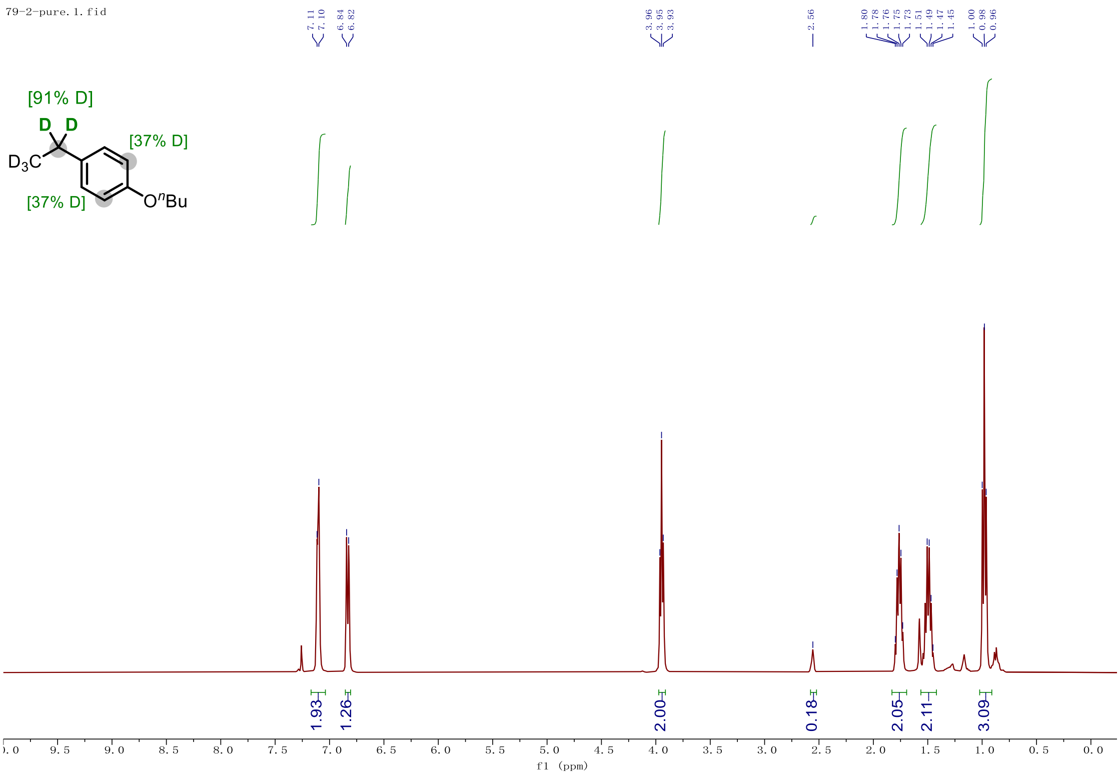

^{13}C NMR of Compound 57 (101 MHz, $CDCl_3$)

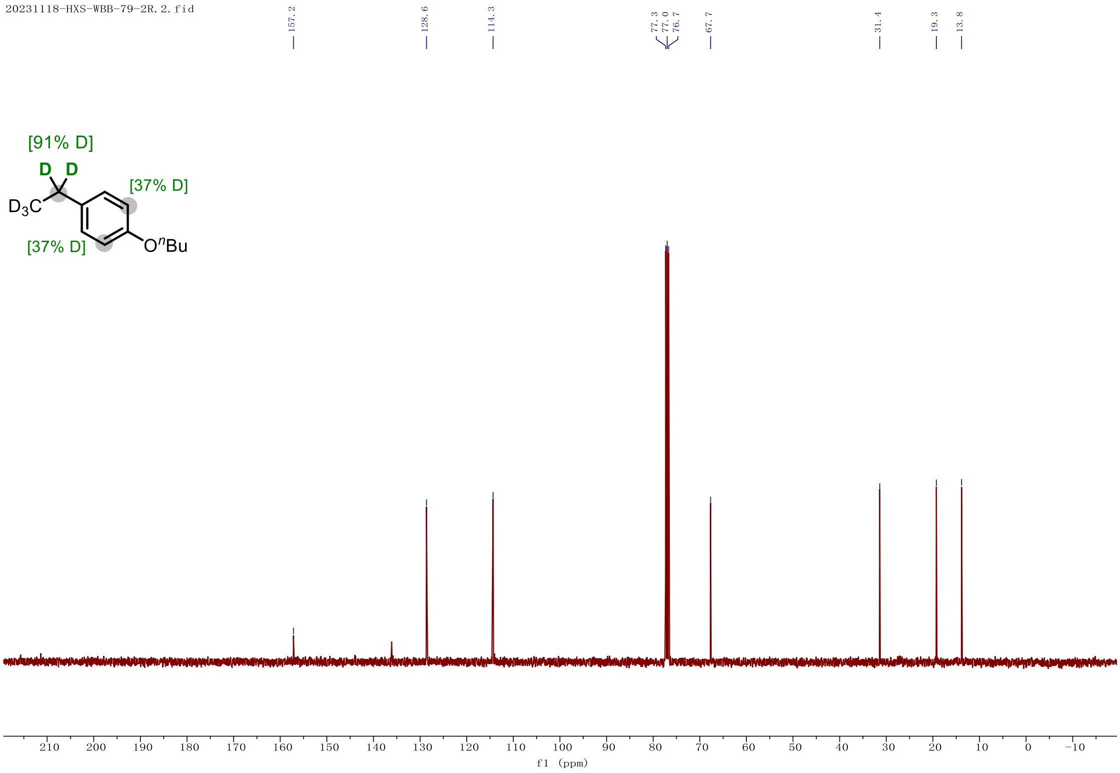

^{1}H NMR of Compound 58 (400 MHz, $CDCl_3$)

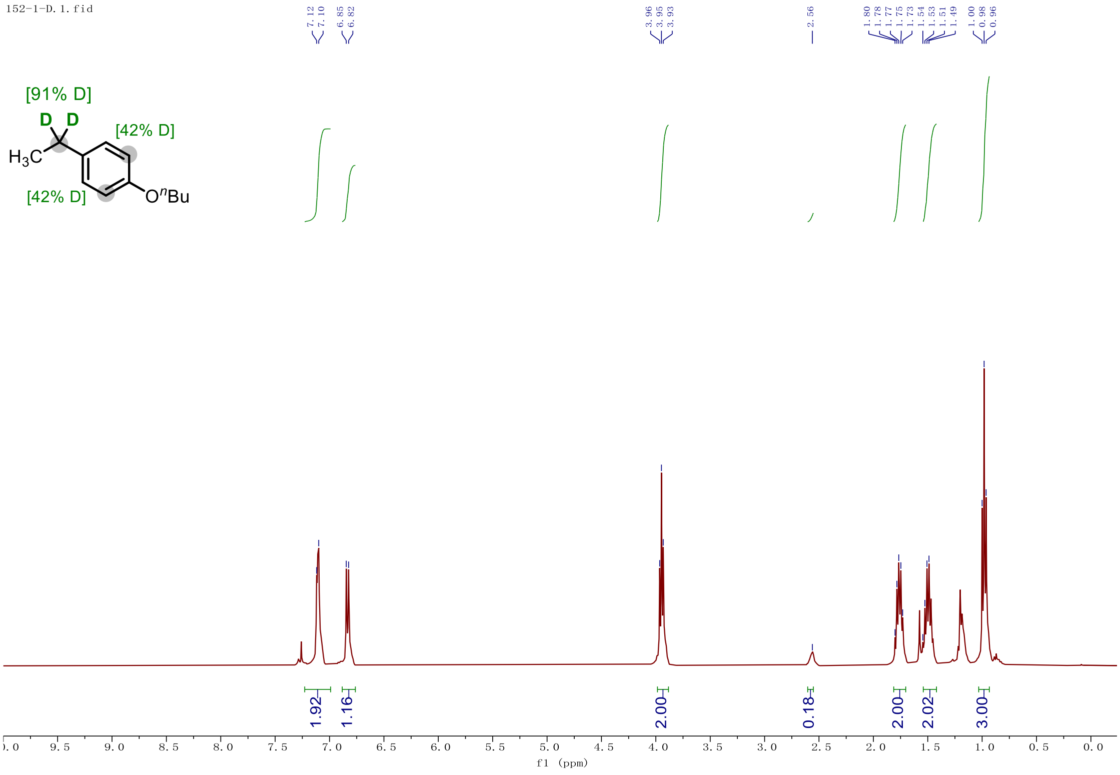

^{13}C NMR of Compound 58 (101 MHz, $CDCl_3$)

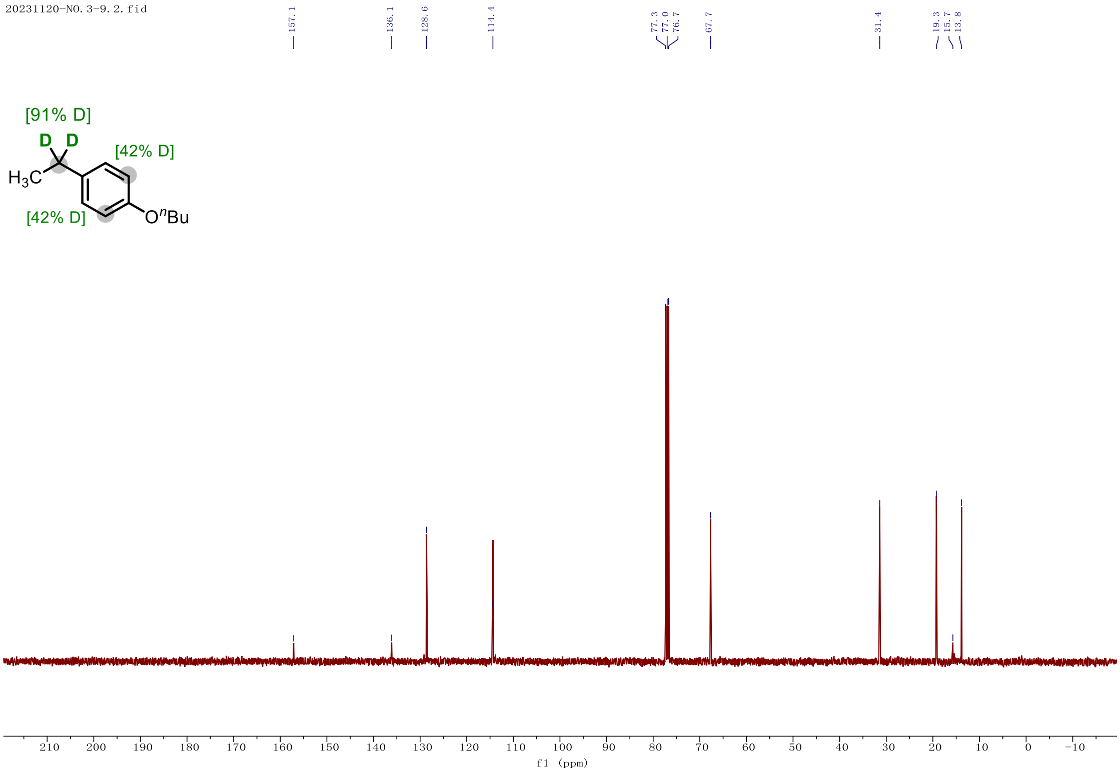

^{1}H NMR of Compound 59 (400 MHz, $CDCl_3$)

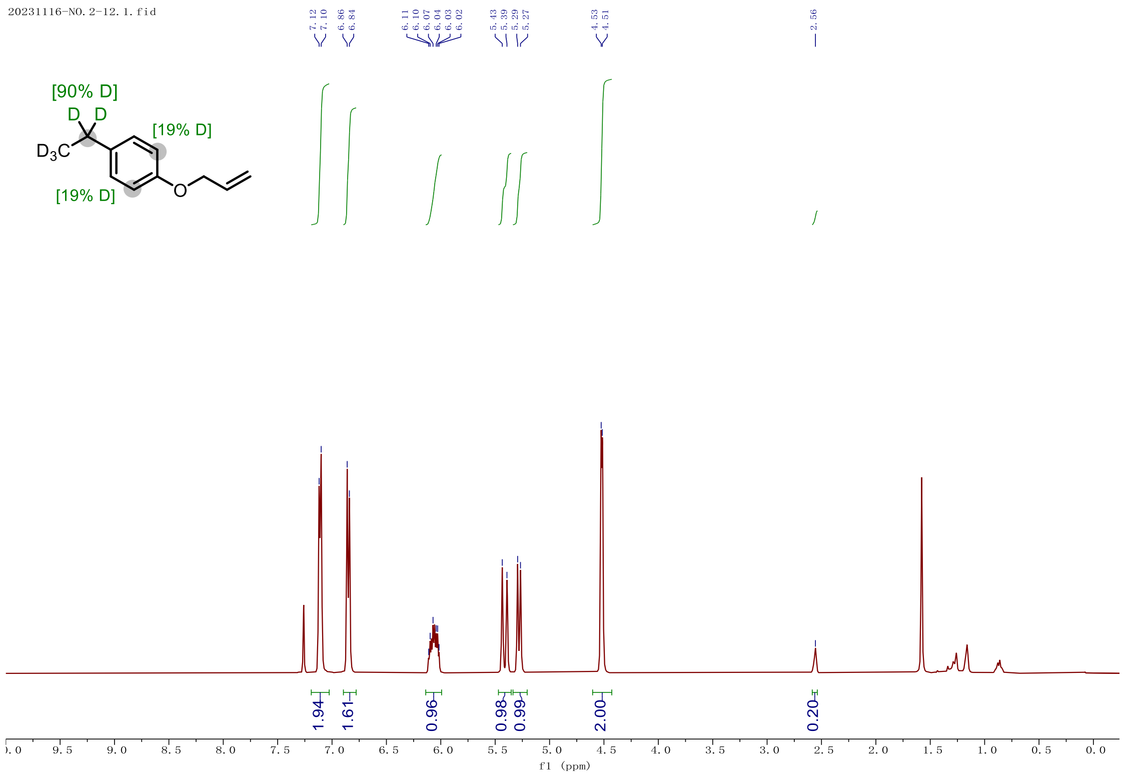

^{13}C NMR of Compound 59 (101 MHz, $CDCl_3$)

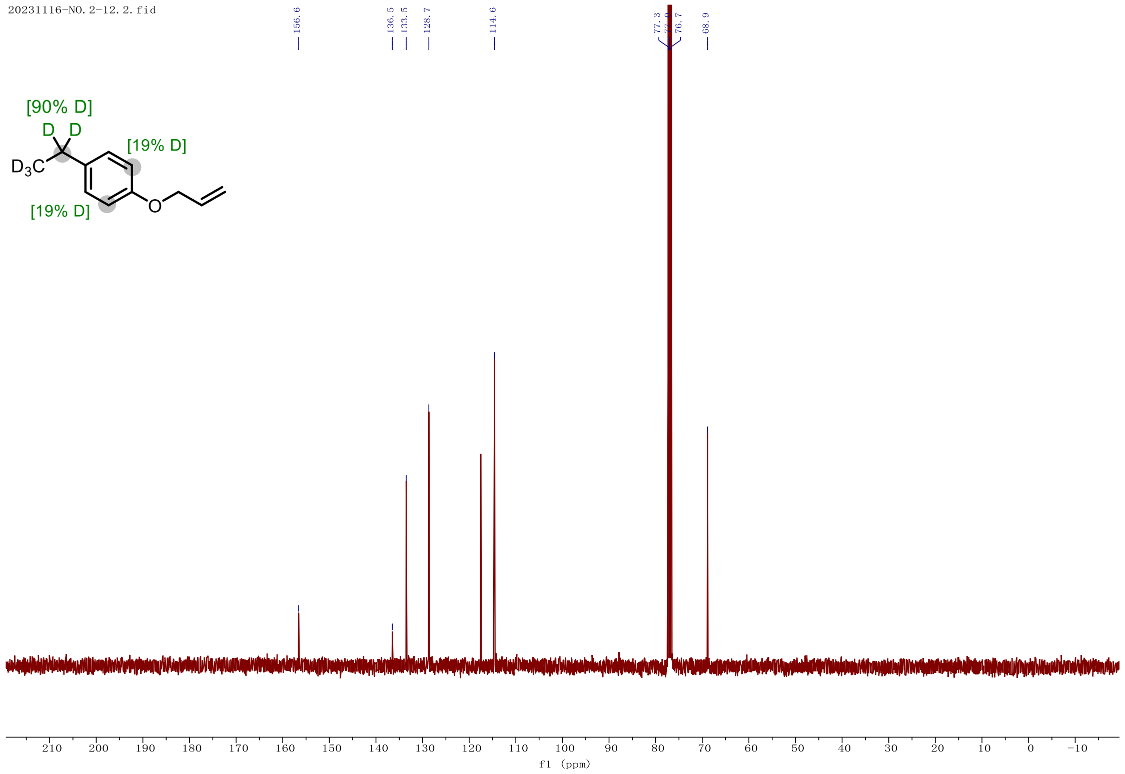

^{1}H NMR of Compound 60 (400 MHz, $CDCl_3$)

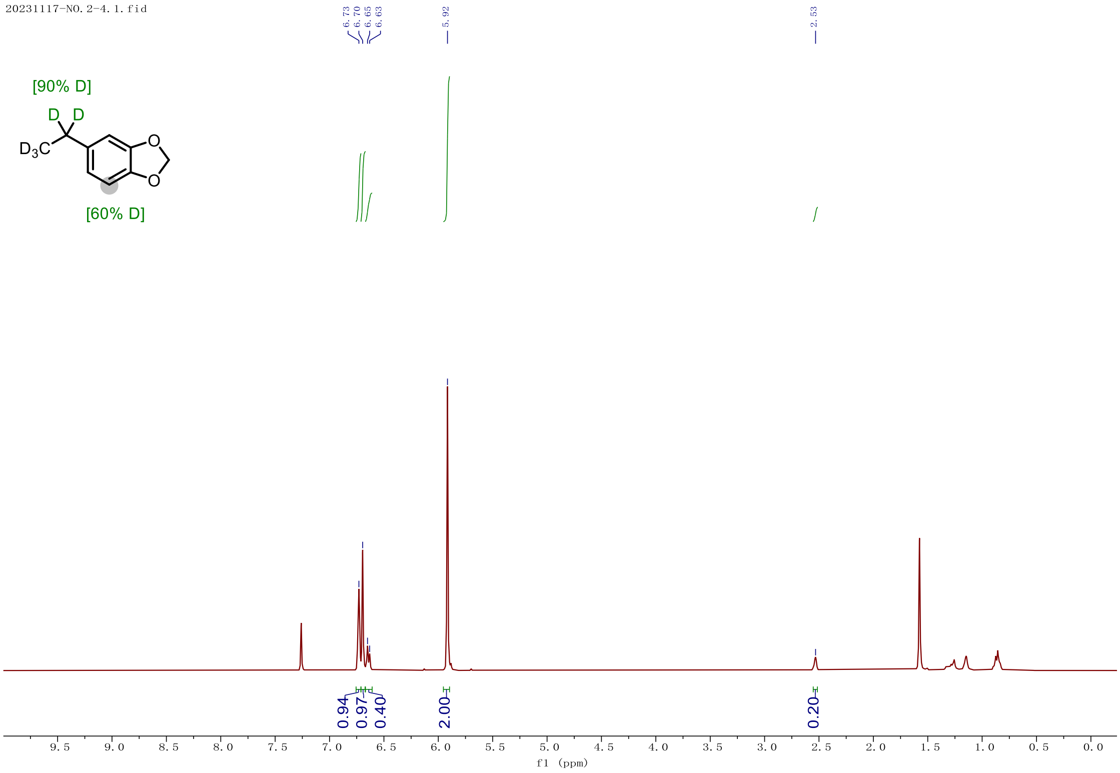

^{13}C NMR of Compound 60 (101 MHz, $CDCl_3$)

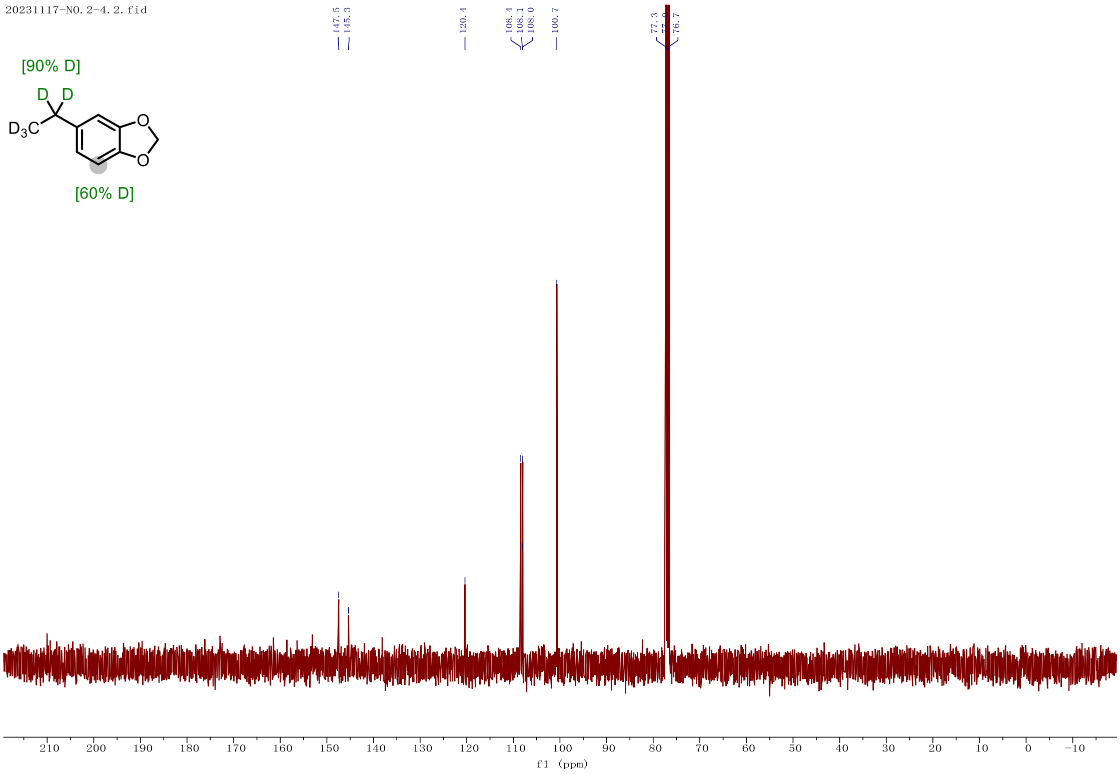

^{1}H NMR of Compound 61 (400 MHz, $CDCl_3$)

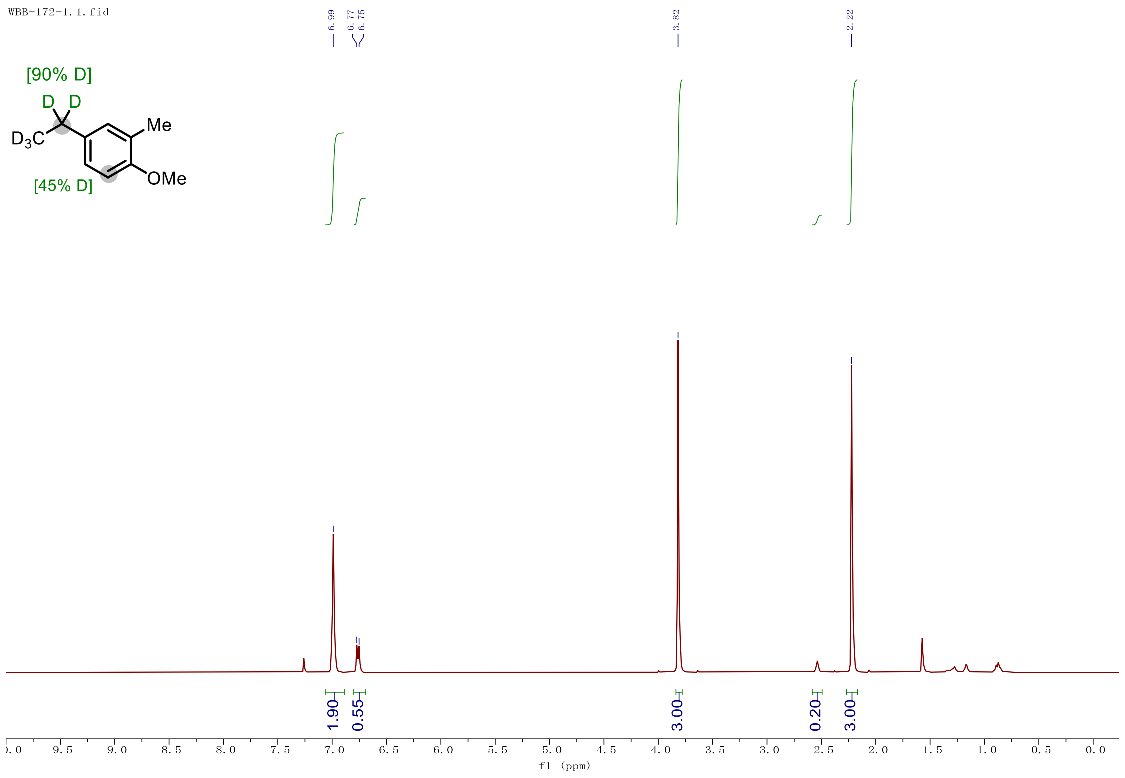

^{13}C NMR of Compound 61 (101 MHz, $CDCl_3$)

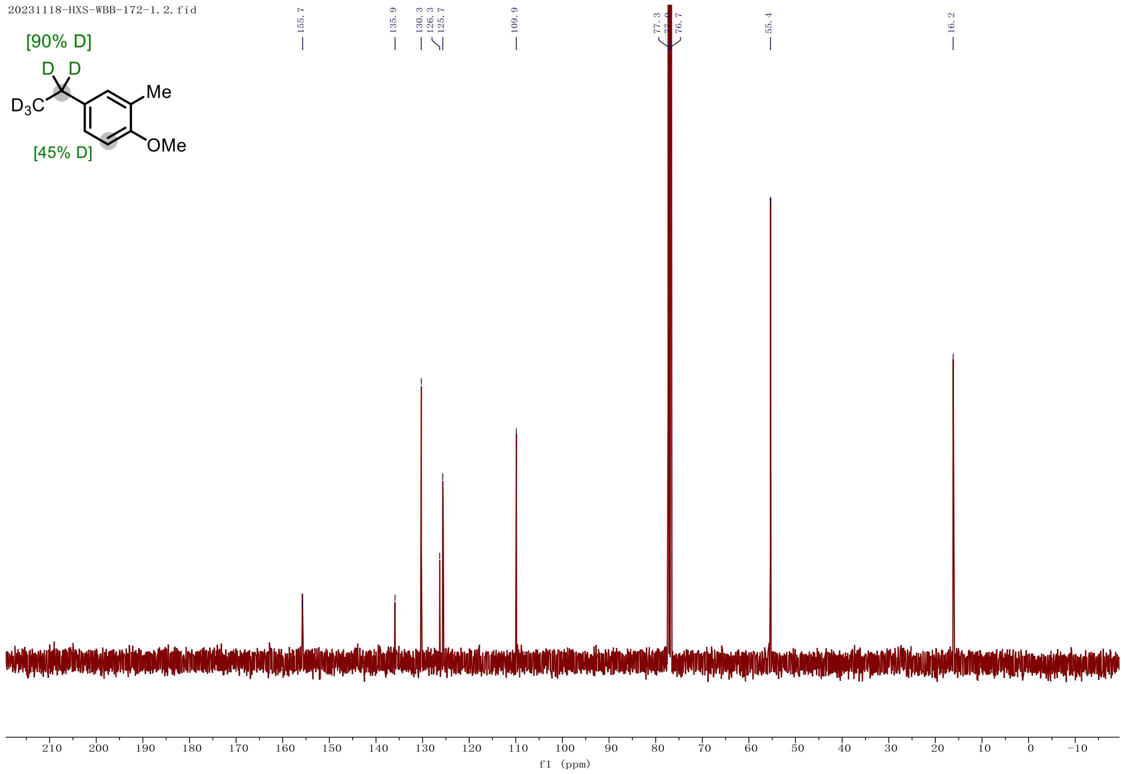

^{1}H NMR of Compound 62 (400 MHz, $CDCl_3$)

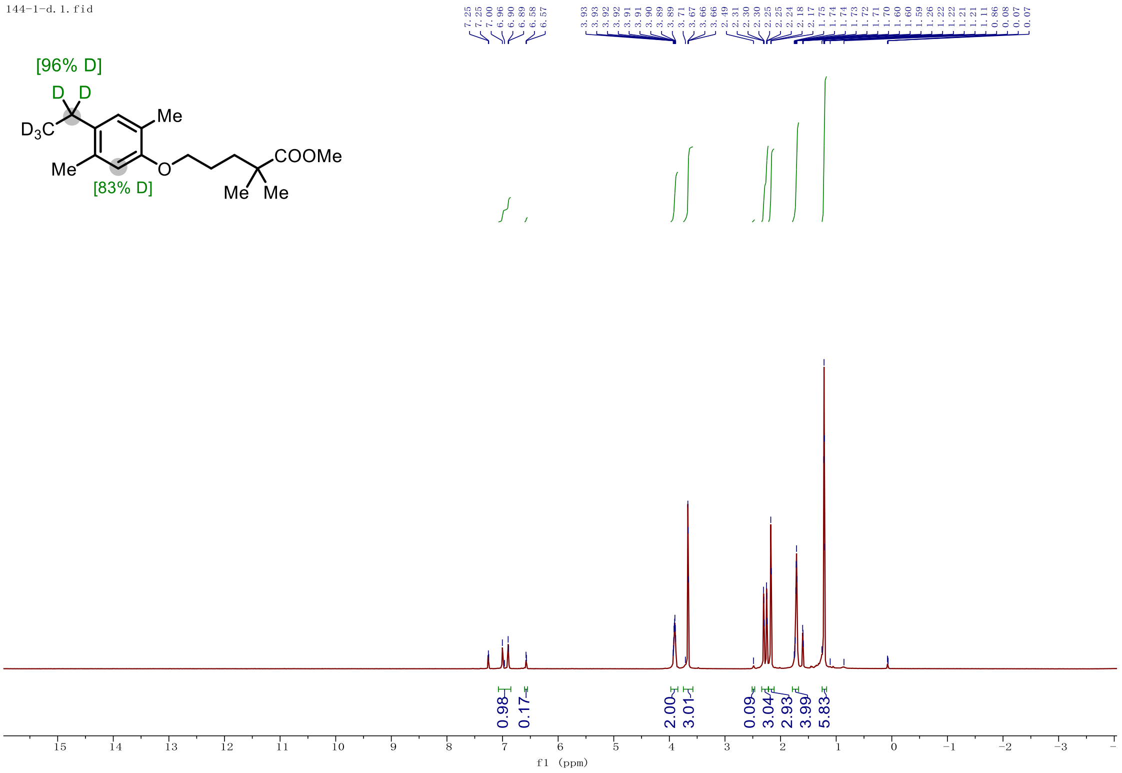

^{13}C NMR of Compound 62 (101 MHz, $CDCl_3$)

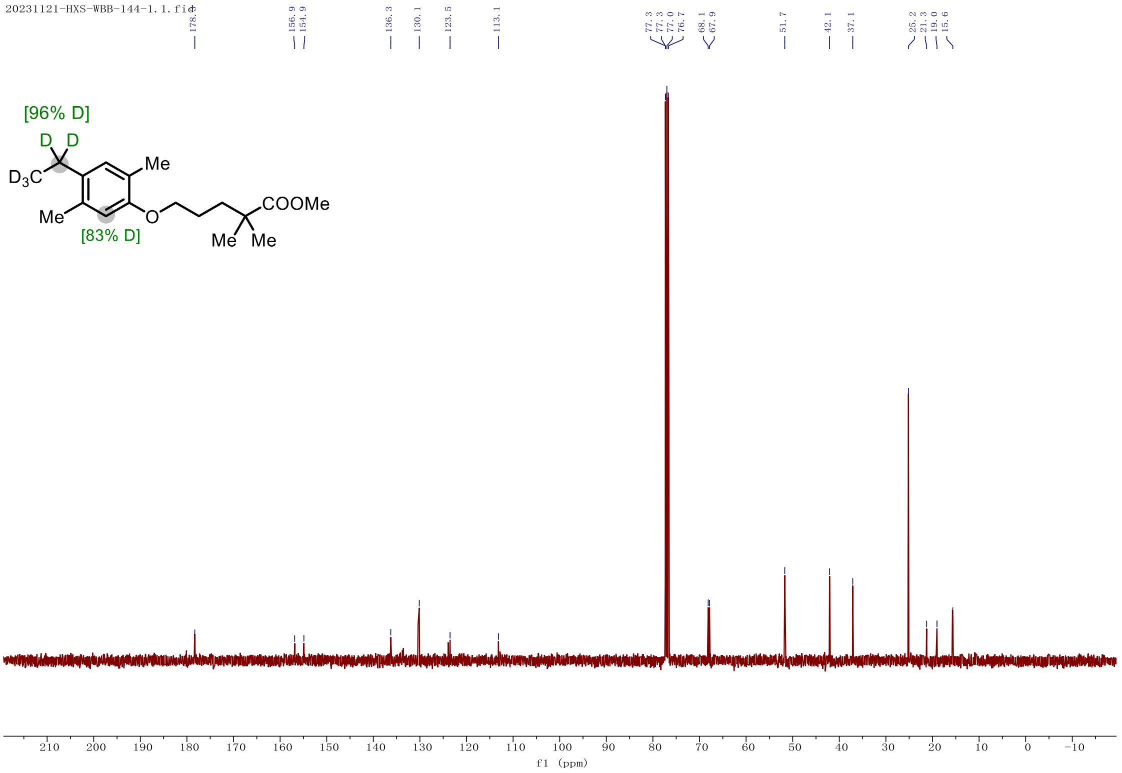

^{1}H NMR of Compound 63 (400 MHz, $CDCl_3$)

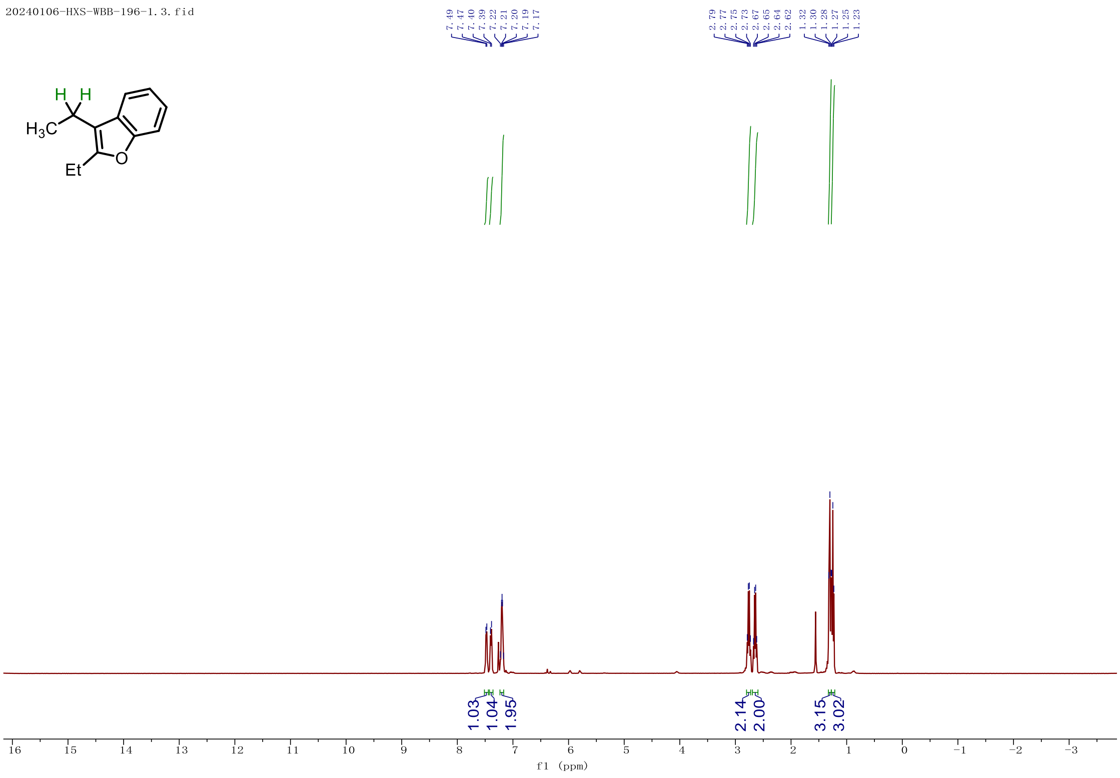

^{13}C NMR of Compound 63 (101 MHz, $CDCl_3$)

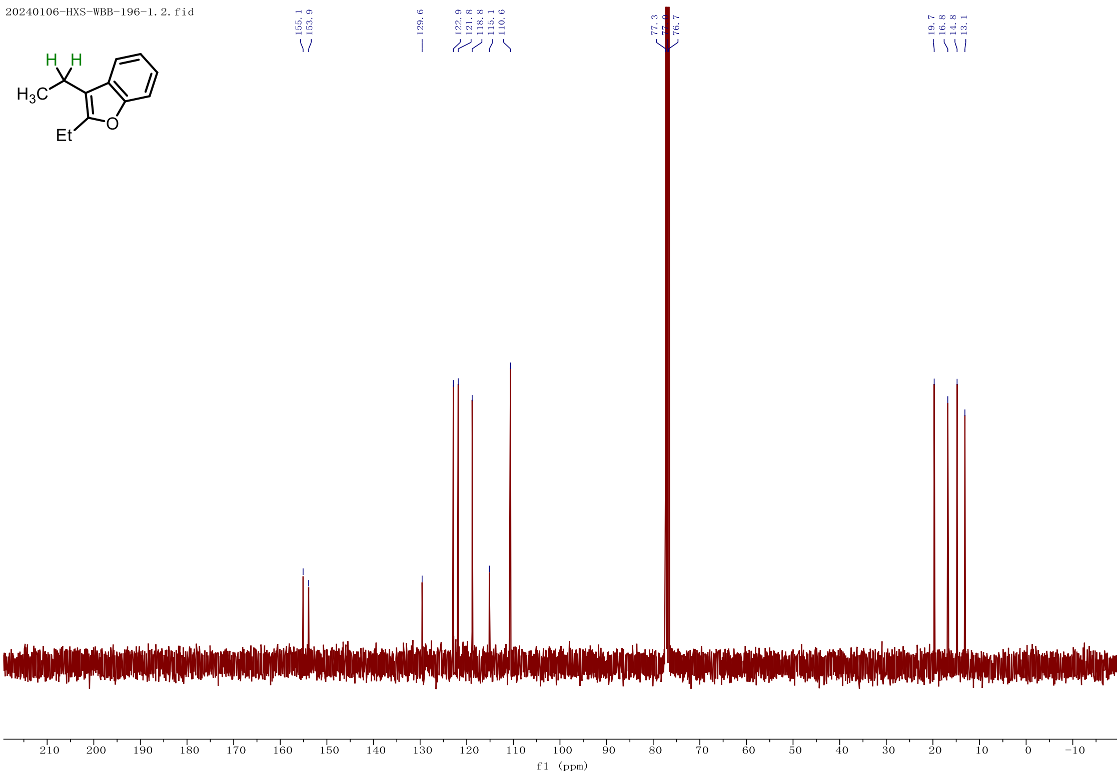

^{1}H NMR of Compound 64 (400 MHz, $CDCl_3$)

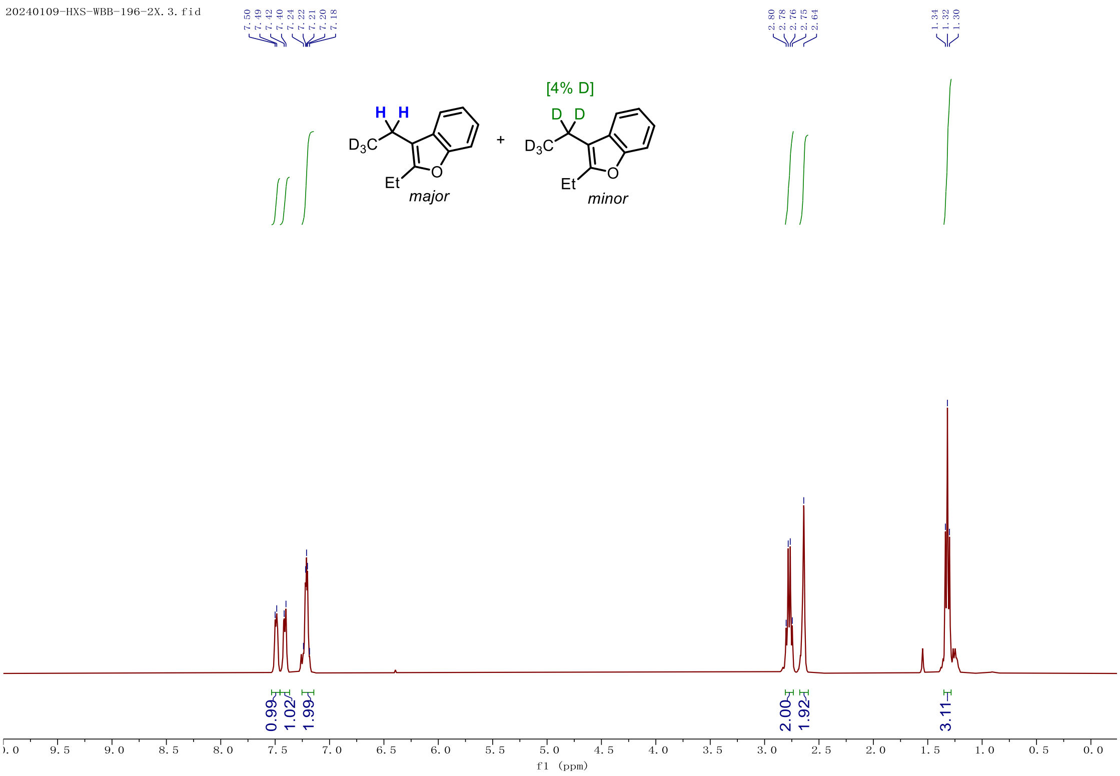

^{13}C NMR of Compound 64 (101 MHz, $CDCl_3$)

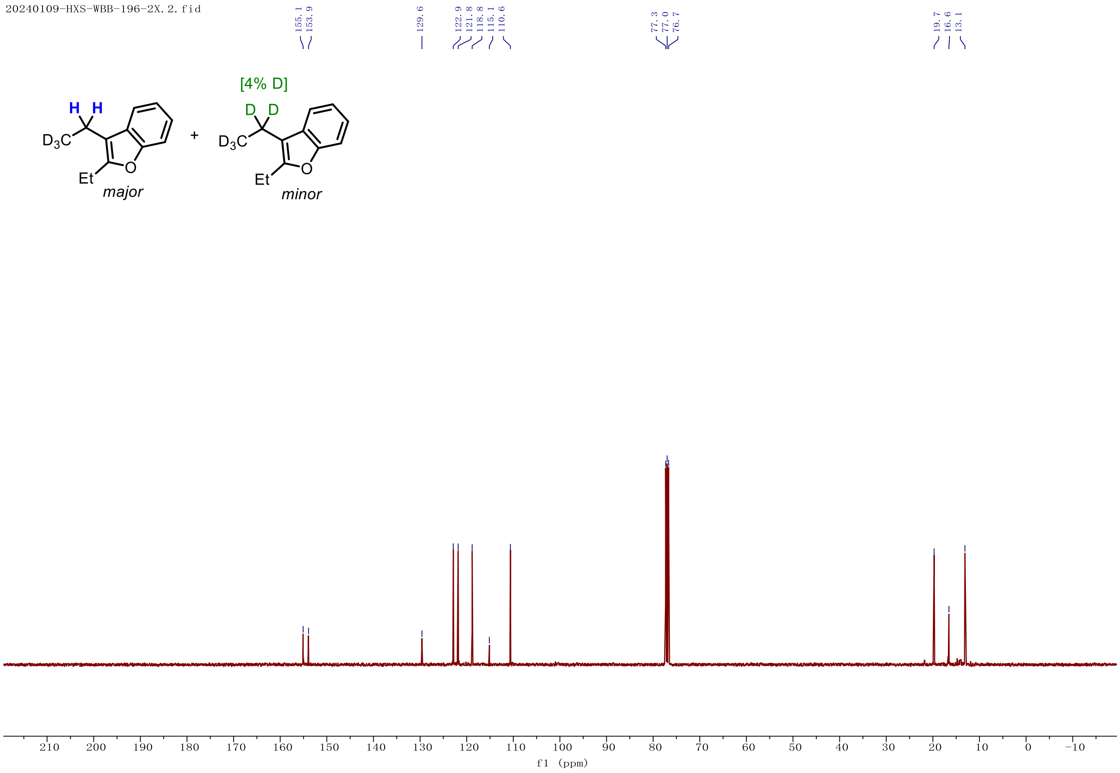

Deuterium NMR of Compound 64 (400 MHz, $CHCl_3$)

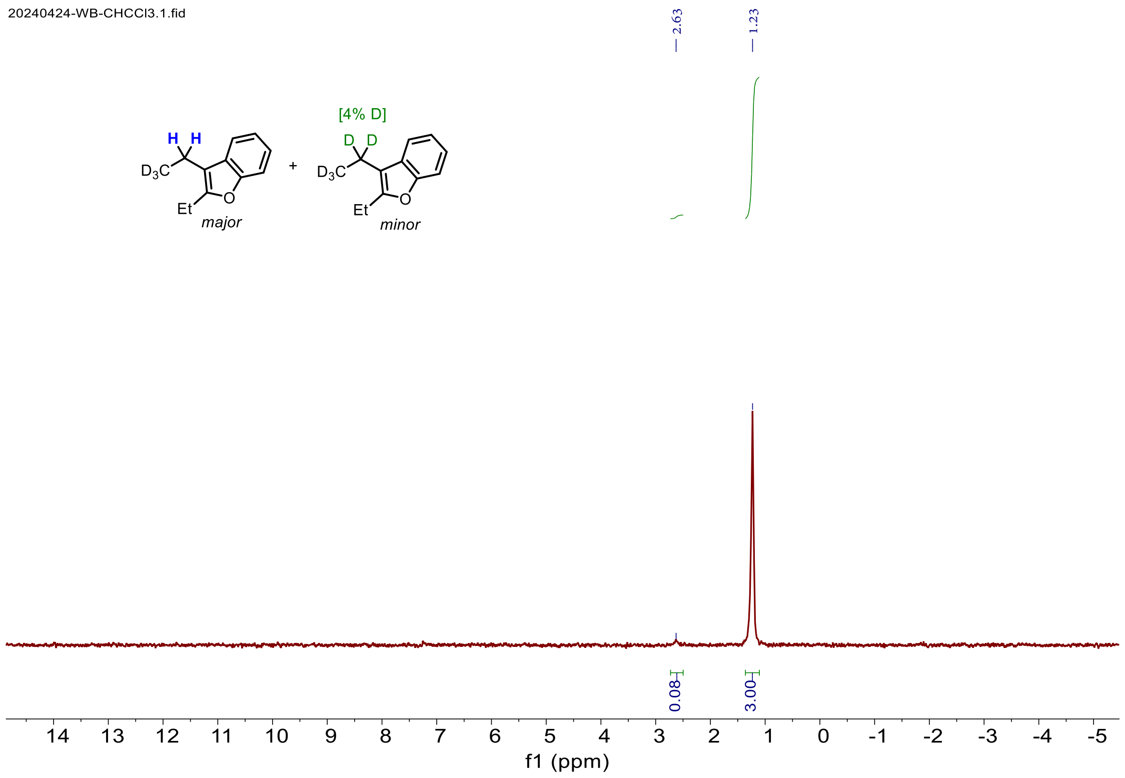

^{1}H NMR of Compound 65 (400 MHz, $CDCl_3$)

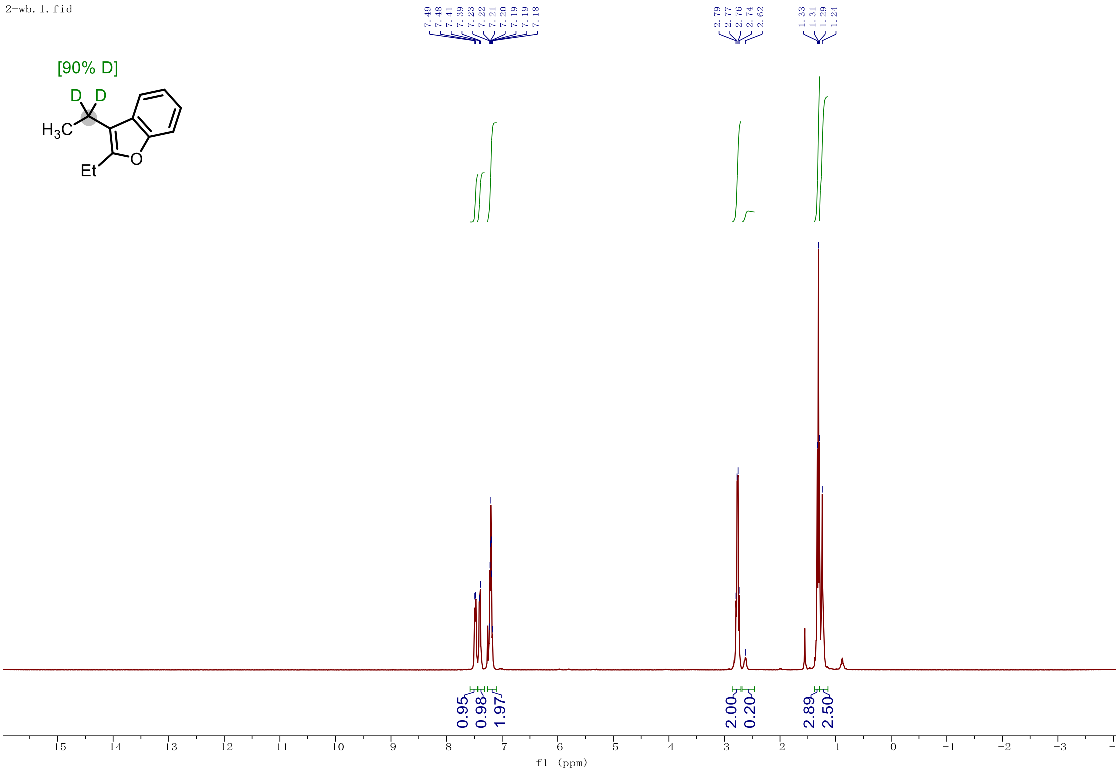

^{13}C NMR of Compound 65 (101 MHz, $CDCl_3$)

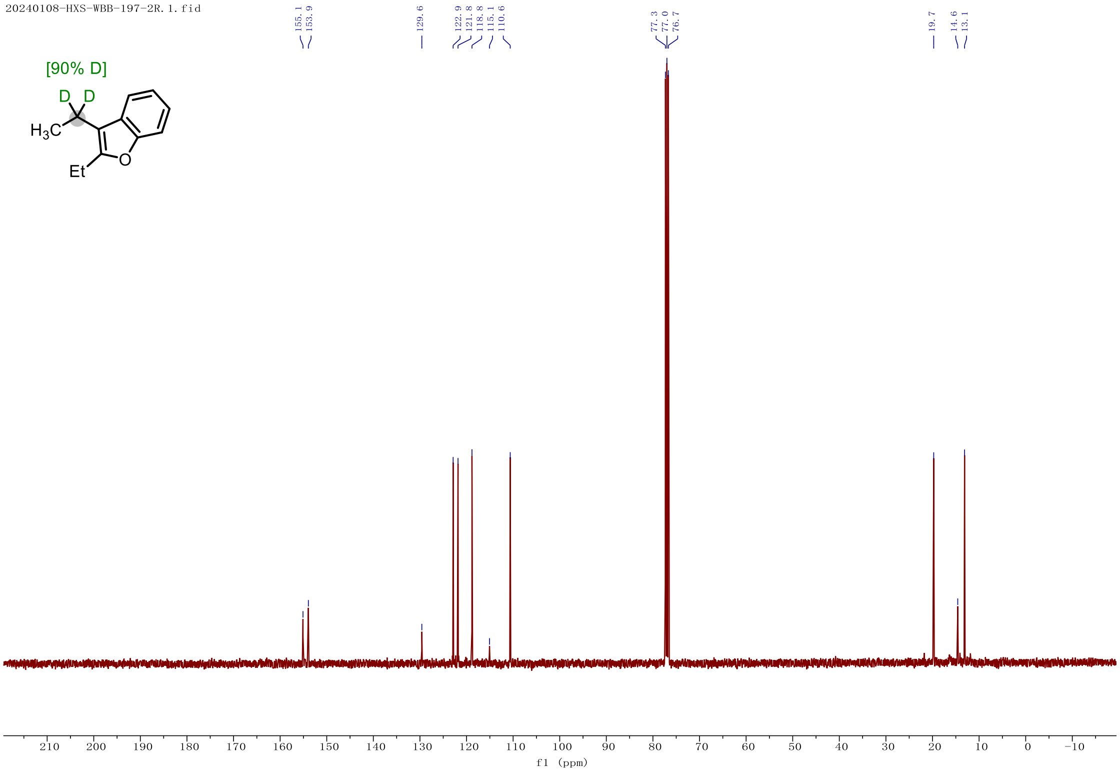

^{1}H NMR of Compound 66 (400 MHz, $CDCl_3$)

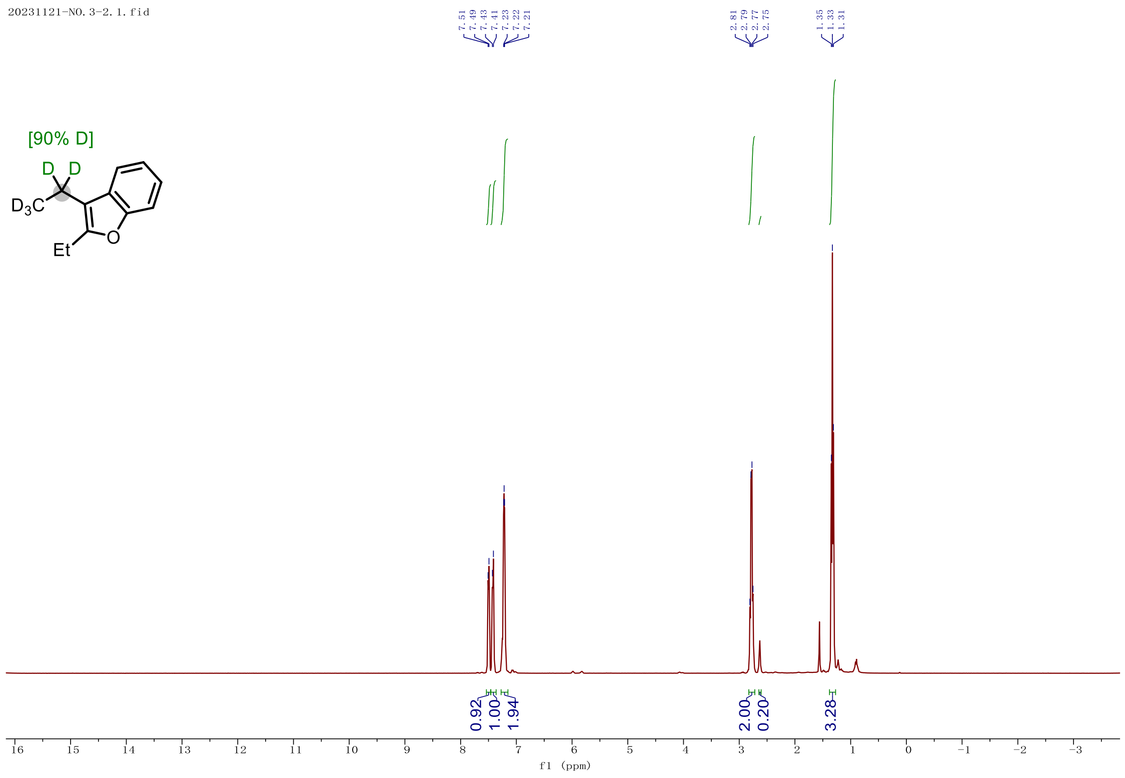

^{13}C NMR of Compound 66 (101 MHz, $CDCl_3$)

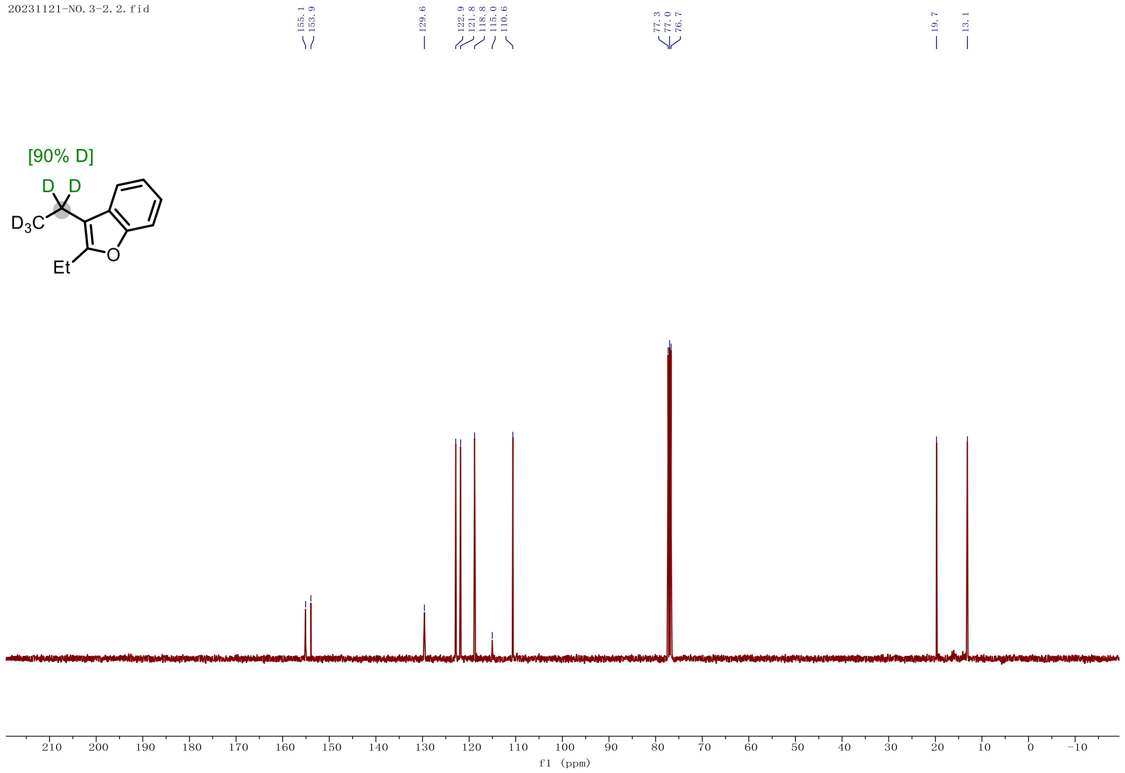

^{1}H NMR of Compound 71 (400 MHz, $CDCl_3$)

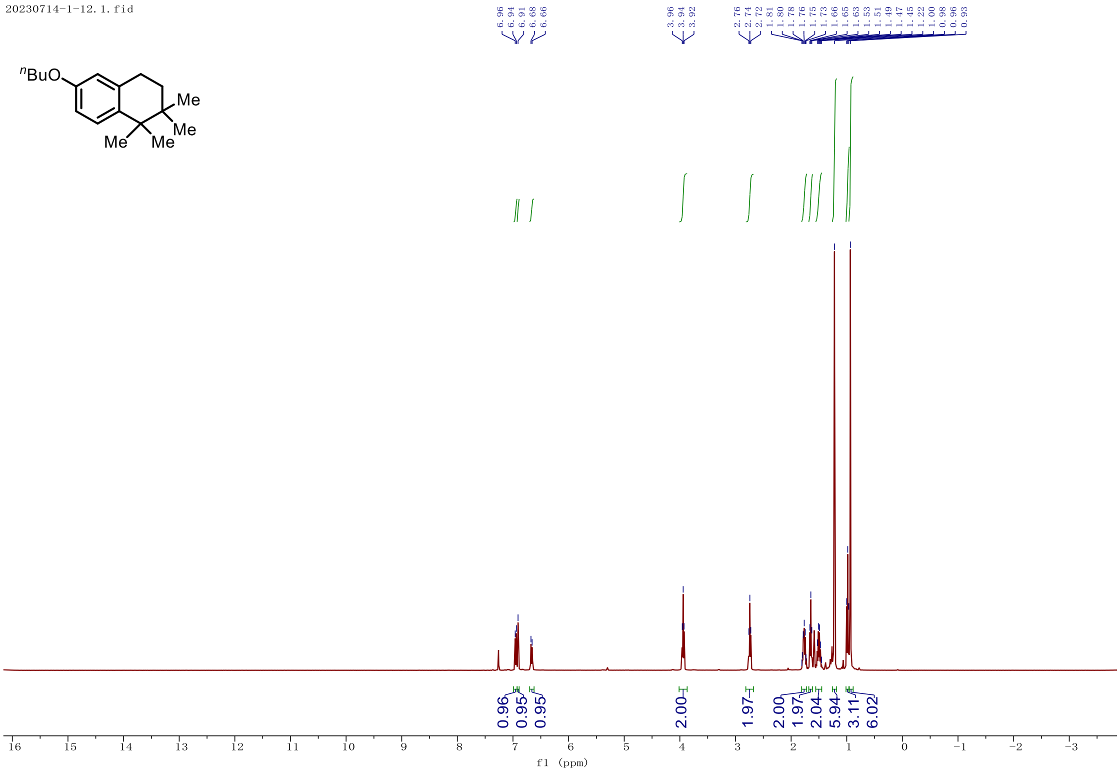

^{13}C NMR of Compound 71 (101 MHz, $CDCl_3$)

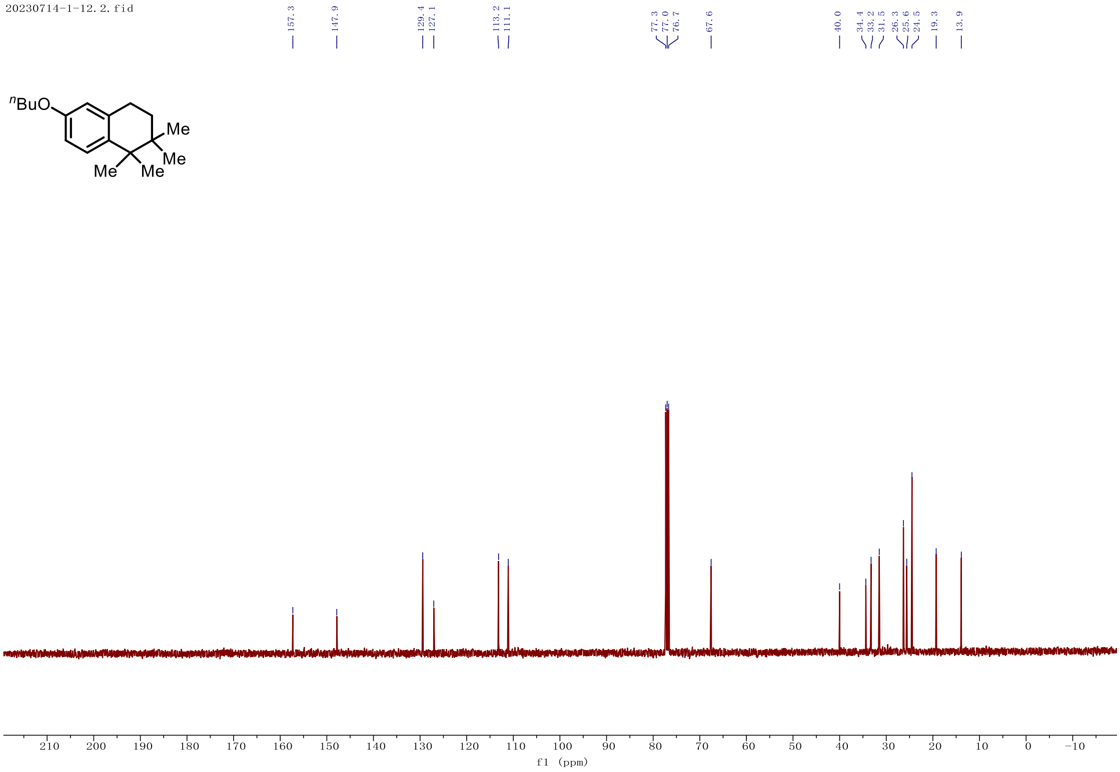

4. Supplementary References

1. Sun, Z.-H. et al. Electrochemical Deoxygenative Hydrogenation and Deuteration of Aldehydes/Ketones by Protic Acids in Water. *Adv. Synth. Catal.* **365**, 476–481 (2023).
2. Zhao, J. et al. Site-Specific Oxidation of (sp^3) C–C (sp^3)/H Bonds by $NaNO_2$/HCl. *Org. Lett.* **23**, 4057–4061(2021).
3. Aoki, Y., Nomoto, S., Hirose, T., Nohira, H. Helical Twisting Power of New Chiral Dopants Derived from 2-Phenylpropanoic Acid for Nematic Liquid Crystals *Mol. Cryst. Liq. Cryst.* **346**, 35–40(2000).
4. Molander, G.-A., Elia, M.-D. Suzuki-Miyaura Cross-coupling Reactions of Benzyl Halides with Potassium Aryltrifluoroborates. *J. Org. Chem.* **71**, 9198–9202(2006).
5. Ghosh, R., Sarkar, A. Bidentate P, N-P ligand for Nickel-Catalyzed Cross-Coupling of Aryl or Benzyl Chlorides with ArMgX. *J. Org. Chem.* **75**, 8283–8286(2010).
6. Wang, X.-X., Xu, B.-B., Song, W.-T., Sun, K.-X., Lu, J.-M. N-heterocyclic Carbene–palladium(II)-1-methylimidazole Complex-catalyzed Suzuki-Miyaura Coupling of Benzyl Sulfonates with Arylboronic acids. *Org. Biomol. Chem.* **13**, 4925–4930(2015).
7. Álvaro, M., Das, D., Cano, M., Garcia, H. Friedel-Crafts Hydroxyalkylation: Reaction of Anisole with Paraformaldehyde Catalyzed by Zeolites in Supercritical CO_2. *J. Catal.* **219**, 464–468(2003).
8. Chatupheeraphat, A. et al. Ligand-controlled Chemoselective C (acyl)–O bond vs C (aryl)–C Bond Activation of Aromatic Esters in Nickel Catalyzed C (sp^2)–C (sp^3) Cross-couplings. *J. Am. Chem. Soc.* **140**, 3724–3735(2018).
9. Peng, L., Li, Z., Yin, G. Photochemical Nickel-catalyzed Reductive Migratory Cross-coupling of Alkyl Bromides with Aryl Bromides. *Org. Lett.* **20**, 1880–1883(2018).
10. Shimizu, R., Okada, Y., Chiba, K. Stepwise radical cation Diels-Alder reaction via multiple pathways. *Beilstein J. Org. Chem.* **14**, 704–708(2018).
11. Si, X.-G. et al. Enantioselective Synthesis of cis-Decalins by Merging the Birch Reduction and Inverse-electron-demand Diels-Alder Reaction. *Angew. Chem. Int. Ed.* **62**, e202303876(2023).

12. Srivastava, V. Continuous-flow Synthesis of Ruthenium Nanoparticles Using a Microreactor for the Selective Hydrogenation Reaction. *Letters in Organic Chemistry.* **20**, 1077–1088(2023).

13. Li, Y. et al. Reaction Scope and MechanisticInsights of Nickel-catalyzed Migratory Suzuki-Miyaura Cross-coupling. *Nat. Commun.* **11**, 417(2020).

14. Moriya, T., Takayama, K., Konakahara, T., Ogiwara, Y., Sakai, N. Indium(III)-catalyzed Reductive Monoalkylation of Electron-rich Benzenes with Aliphatic Carboxylic Acids Leading to Arylalkane Derivatives. *Eur. J. Org. Chem.* **2015**, 2277–2281(2015).

15. Burmaoglu, S. et al. Assessing the Antiangiogenic Effects of Chalcones and Their Derivatives. *Polycycl. Aromat. Comp.* **44**, 51–66(2024).

16. Ding, S., Xu, L., Li, P. Copper-catalyzed Boron-selective C (sp^2)–C (sp^3) Oxidative Cross-Coupling of Arylboronic Acids and Alkyltrifluoroborates Involving a Single-electron Transmetalation Process. *ACS Catal.* **6**, 1329–1333(2016).

17. Tolnai, G.-L. Efficient Direct 2,2,2-Trifluoroethylation of Indoles via C–H Functionalization. *Chem. Commun.* **51**, 4488–4491(2015).

18. Verma, S.-K., Prajapati, A., Saini, M.-K., Basak, A.-K. Lewis Acid Catalyzed Reductive Cyclization of 2-Aryloxybenzaldehydes and 2-(Arylthio)benzaldehydes to Unsubstituted 9H-Xanthenes and Thioxanthenes Indiisopropyl Ether. *Adv. Synth. Catal.* **363**, 532–539(2021).
